# Construction of a high-density genetic map based on specific-locus amplified fragment sequencing and identification of loci controlling anthocyanin pigmentation in Yunnan red radish

**DOI:** 10.1093/hr/uhab031

**Published:** 2022-02-10

**Authors:** Jing Tao, Shikai Li, Qian Wang, Yi Yuan, Jiqiong Ma, Minghui Xu, Yi Yang, Cui Zhang, Lijuan Chen, Yiding Sun

**Affiliations:** 1 College of Agronomy and Biotechnology, Yunnan Agriculture University, 452 Fengyuan Road, Kunming, 650201, China; 2 Engineering Research Center of Vegetable Germplasm Innovation and Support Production Technology, Horticultural Research Institute, Yunnan Academy of Agricultural Sciences; 2238 Beijing Road, Kunming, 650205, China; 3 Key Lab of Agricultural Biotechnology of Yunnan Province, Key Lab of Southwestern Crop Gene Resources and Germplasm Innovation of Ministry of Agriculture, Biotechnology and Germplasm Resources Research Institute, Yunnan Academy of Agricultural Sciences, 2238 Beijing Road, Kunming, 650205, China; 4 College of Plant Protection, Yunnan Agricultural University, 452 Fengyuan Road, Kunming, 650201, China

## Abstract

Radish (*Raphanus sativus* L.) belongs to the family Brassicaceae. The Yunnan red radish variety contains relatively large amounts of anthocyanins, making them important raw materials for producing edible red pigment. However, the genetic mechanism underlying this pigmentation has not been fully characterized. Here, the radish inbred line YAAS-WR1 (white root skin and white root flesh) was crossed with the inbred line YAAS-RR1 (red root skin and red root flesh) to produce F_1_, F_2_, BC_1_P_1_, and BC_1_P_2_ populations. Genetic analyses revealed that the pigmented/non-pigmented and purple/red traits were controlled by two genetic loci. The F_2_ population and the specific-locus amplified fragment sequencing (SLAF-seq) technique were used to construct a high-density genetic map (1230.16 cM), which contained 4032 markers distributed in nine linkage groups, with a mean distance between markers of 0.31 cM. Additionally, two quantitative trait loci (QAC1 and QAC2) considerably affecting radish pigmentation were detected. A bioinformatics analysis of the QAC1 region identified 58 predicted protein-coding genes. Of these, *RsF3′H*, which is related to anthocyanin biosynthesis, was revealed as a likely candidate gene responsible for the purple/red trait. The results were further verified by analyzing gene structure and expression. Regarding QAC2, *RsMYB1.3* was determined to be a likely candidate gene important for the pigmented/non-pigmented trait, with a 4-bp insertion in the first exon that introduced a premature termination codon in the YAAS-WR1 sequence. Assays demonstrated that RsMYB1.3 interacted with RsTT8 and activated *RsTT8* and *RsUFGT* expression*.* These findings may help clarify the complex regulatory mechanism underlying radish anthocyanin synthesis. Furthermore, this study’s results may be relevant for the molecular breeding of radish to improve the anthocyanin content and appearance of the taproots.

## Introduction

Anthocyanins, which are natural water-soluble pigments, are widely found in plants. Anthocyanin synthesis is an important physiological activity because anthocyanins provide plant parts with bright colors that attract pollinators and animals that spread seeds [1]. They are also effective radical scavengers that protect plants against biotic and abiotic stresses [2, 3]. Anthocyanins have recently attracted considerable attention because of their biological activities (e.g. inhibiting cell mutation and proliferation as well as lowering blood pressure) and their anti-inflammatory, antibacterial, and anti-oxidant properties [4–6]. Therefore, vegetables and fruits rich in anthocyanins and industrial products containing natural anthocyanins are increasingly being consumed and used.

The structural and regulatory genes related to anthocyanin synthesis are relatively conserved in various plant species [7]. As a direct precursor for anthocyanin biosynthesis, phenylalanine is converted to 4-coumaryl CoA in reactions catalyzed by phenylalanine ammonia-lyase (PAL) and cinnamate-4-hydroxylase (C4H). Chalcone synthase (CHS) and flavanone-3-hydroxylase (F3H) convert 4-coumaryl CoA and malonyl CoA to dihydroflavonol, which is converted to a colorless leucoanthocyanidin by dihydroflavonol 4-reductase (DFR). The leucoanthocyanidin is further transformed to produce compounds with diverse colors, including blue–purple, brick red, and blue, in reactions catalyzed by anthocyanin synthase (ANS/LDOX) and UDP-glucose:flavonoid-3-*O*-glucosyltransferase (UFGT) [8]. The transcriptional regulatory mechanisms involved in anthocyanin biosynthesis have been extensively studied. Previous research demonstrated that R2R3-MYBs, bHLHs and WD40 form the MYB-bHLH-WD40 (MBW) complex, which affects the synthesis of anthocyanins by regulating the expression of structural genes [9, 10]. Additionally, jasmonate ZIM-domain proteins [11], *SQUAMOSA* promoter-binding protein-like transcription factors [12], NAC family transcription factors [13], ERF family transcription factors involved in the ethylene signaling pathway, R3-MYB transcription factors [14–16], and other factors also help regulate anthocyanin synthesis by interacting with MYB, bHLH, and other transcription factors in plants [17, 18].

Radish (*Raphanus sativus* L.), which belongs to the family Brassicaceae, is an important horticultural crop worldwide. Specifically, it is produced for its seed oil, sprouts, and edible taproots. As the main storage site of secondary metabolites in radish, the taproot is rich in carbohydrates, organic nutrients, and dietary fiber [19, 20]. Additionally, some radish varieties contain large amounts of anthocyanins. Yunnan red radish is a special germplasm resource that is rich in anthocyanidins. Furthermore, it has diverse industrial uses (e.g. production of food products, medicines, and cosmetics) because of its rose color and highly stable red pigment. However, because of years of non-standardized cultivation, the mixed germplasm of the radish varieties currently used for commercial production has affected the quality of the red pigment in radish [21]. To generate highly pure hybrids, the flesh color instability of the commercially cultivated varieties needs to be addressed. This requires a more thorough understanding of the genetic mechanism underlying anthocyanin synthesis in Yunnan red radish.

A previous study indicated that exogenous methyl jasmonate, gibberellin, and UV-A induce anthocyanin production and accumulation in white fleshy radish varieties [22]. These results suggested that structural genes involved in the anthocyanin synthesis pathway are conserved in radish germplasms that differ regarding color. The pigment in red radishes is detectable at 2 or 3 days after germination, implying that anthocyanin synthesis is initiated during the early germination stage and the distribution pattern is fixed [23, 24]. Transcriptional analyses revealed that *RsDFR*, *RsANS*, *RsUFGT*, *RsF3H*, *RsCHS3*, and *RsF3′H* genes are more highly expressed in red fleshy radishes than in other varieties, with *RsUFGT* expression critical for the spatiotemporal accumulation of anthocyanins [18, 25–28], Recent studies confirmed that radish homologs of *AtPAP1* were the key regulatory factors determining the accumulation of anthocyanins in radish because the encoded transcription factor directly regulates the expression of anthocyanin synthesis-related structural genes [29–31]. However, the structural genes and the *AtPAP1* homolog involved in regulating anthocyanin production are differentially expressed among diverse red radish varieties [18, 25–28, 32]. Thus, the genetic mechanisms underlying the coloration of radish plants vary among genotypes. As a special local germplasm resource, a high-quality genetic map should be constructed for Yunnan red radish to enable the mapping of genes associated with specific agronomic traits.

Specific-locus amplified fragment sequencing (SLAF-seq) was recently developed as a high-throughput sequencing technique that decreases the complexity and cost of constructing high-quality reference genome libraries [33]. It has been used for the high-quality genetic linkage mapping of horticultural crops, such as cucumber [34], pea [35], zicaitai [36], and broccoli [37]. In the current study, the radish inbred line YAAS-WR1 (white root skin and white root flesh) was crossed with the inbred line YAAS-RR1 (red root skin and red root flesh) to generate the F_1_, F_2_, BC_1_P_1_, and BC_1_P_2_ populations. The genetic characteristics of Yunnan red radish coloration were explained by multigeneration joint analysis. Then an F_2_ population of 200 individuals was used to fine-map anthocyanin accumulation-related genes. SLAF-seq was used to construct a high-density genetic map spanning 1230.16 cM, with 4032 SLAF markers distributed in nine linkage groups (LGs). The mapping of anthocyanin synthesis-related loci revealed two quantitative trait loci (QTL). Linked markers were developed to predict and structurally analyze the candidate genes potentially applicable for the marker-assisted selection-based breeding of new radish varieties with improved pigment production. The identification and functional analysis of candidate genes for major QTL further revealed the molecular mechanism underlying the regulation of anthocyanin biosynthesis in radish.

## Results

### Phenotypic characterization of genetical population

**Figure 1:**
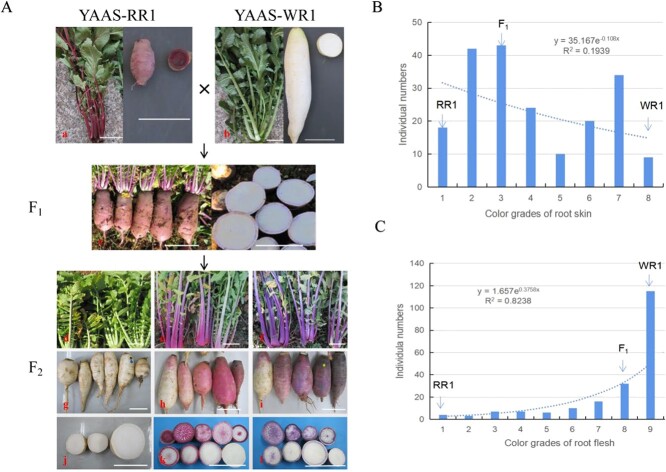
(**a**) Phenotypes of the parental inbred lines and the F_2_ individuals. (**a**, a) Maternal line YAAS-RR1. (**a**, b) Paternal line YAAS-WR1. (**a**, d–**a**, l) Phenotypes of the F_2_ individuals with diverse coloration. (**b**) Frequency distribution of the pigmented-skin trait among F_2_ individuals. (**c**) Frequency distribution of the pigmented-flesh trait among F_2_ individuals.

To determine the inheritance of the pigment accumulation trait, the F_1_, F_2_, BC_1_P_1_, and BC_1_P_2_ populations of YAAS-RR1 and YAAS-WR1 were generated. On the basis of the pigmentation of the plants, the F_2_ population was divided into the following three groups: purple-pigmented (PP), red-pigmented (RP), and non-pigmented (NP). Differences in pigment deposition were observed in the individuals of each group. These results indicated that the genetic basis of pigment accumulation in Yunnan red radish is complex (Fig. 1). When the differences in the pigmented parts and in the extent of the pigmentation were not considered for the F_2_ population, the segregation ratio for the PP:RP:NP individuals was 9:3:4. An examination of the rest of the genetic population indicated that the BC_1_P_1_ population only had PP and NP phenotypes, with a 1:1 segregation ratio, whereas the BC_1_P_2_ population only had PP and RP phenotypes, with a 1:1 segregation ratio (Table 1). However, the segregation was consistent with a 3:1 Mendelian ratio for the pigmented individuals (different shades of red and purple) and NP individuals. These results suggest that the pigmented/non-pigmented (PiN) trait and the PP/PR trait are controlled by two dominant genes. The PiN-related gene, which was designated *RsPi* (pigmented), is dominant homozygous in YAAS-RR1. The PP/PR-related gene, which was designated *RsPP* (purple pigment), is dominant homozygous in YAAS-WR1.

**Table 1:** Segregation of plant colors in six populations

Material	Generation	Observed (PP:RP:NP)	Expected (PP:RP:NP)	χ^2^	*P*
WR1	P_2_	0:0:20	0:0:1		
RR1	P_1_	0:20:0	0:1:0		
RR1 × WR1	F_1_	20:0:0	1:0:0		
(RR1 × WR1) × WR1	BC_1_P_2_	25:0:25	1:0:1	0.02	0.8875
(RR1 × WR1) × RR1	BC_1_P_1_	33:49:0	1:1:0	2.7439	0.0976
RR1 × WR1 Self	F_2_	197:74:87	9:3:4	0.8507	0.6535

Analyses of the root skin and flesh indicated that the pigment contents in these two tissues presented a non-normal distribution among the F_2_ population (Fig. 1, Supplementary Table S1). The anthocyanin content of root skin of F_1_ plants was close to that of YAAS-RR1, whereas the anthocyanin content of root flesh of F_1_ plants was close to that of YAAS-WR1 (Fig. 1). These results imply that the genetic mechanism underlying pigment accumulation differs between these two tissues. However, in the F_2_ population, all individuals with pigmented root flesh also had pigmented root skin, suggesting that the gene controlling the pigment accumulation in the root skin was also involved in regulating the accumulation of pigment in the root flesh. Therefore, we mainly focused on identifying the dominant gene *RsPi* based on the root skin pigment content of F_2_ individuals.

### SLAF sequencing raw data statistics

In this study, DNA was extracted from the leaves of 200 F_2_ individuals in the segregating population and the parental lines for subsequent analyses. An examination of the residual restriction sites in the read inserts indicated that the digestion efficiency was 92.23%, which was normal. The high-throughput sequencing produced ~125.97 Gb of data, with 592.11 M reads with a GC content of 40.36%. Additionally, 94.16% of the total reads were designated as high-quality reads (quality score >30).

After eliminating SLAFs with a low sequencing depth, a total of 1 861 747 high-quality SLAFs distributed throughout nine LGs were obtained. The non-polymorphic and repetitive markers were discarded. On the basis of the genotype encoding rule, seven segregation patterns (ef × eg, hk × hk, lm × ll, nn × np, aa × bb, ab × cc, and cc × ab) were determined according to the remaining 979 435 polymorphic SLAFs. Because YAAS-WR1 and YAAS-RR1 are homozygous lines with aa and bb genotypes, 697 859 SLAFs with the aa × bb segregation pattern were used in this study (Supplementary Fig. S1).

### Map construction

To produce a high-quality map, low-quality SLAFs with a parental sequencing depth <10× or covering <70% of the individuals were discarded and 4032 high-quality SLAFs were retained to construct a genetic map. For the map, the average sequencing depths were 15.54× in the maternal line, 15.66× in the paternal line, and 25.32× in each F_2_ progeny.

**Figure 2:**
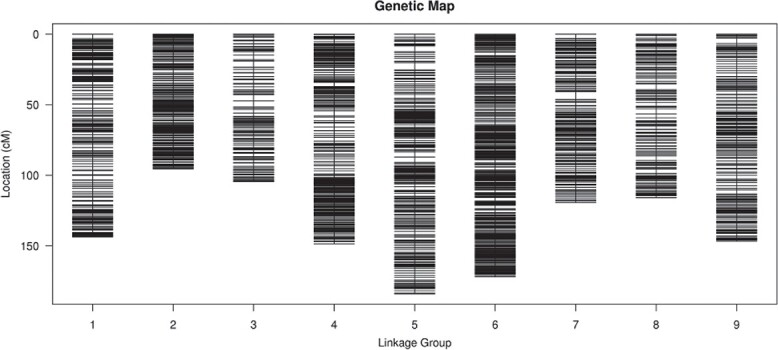
Radish genetic map. The *x*-axis presents the linkage group and the *y*-axis presents the genetic distance.

All selected SLAFs were distributed evenly in nine LGs (Fig. 2). The linear arrangements and the genetic distances of markers in each LG were analyzed using the HighMap software. The average integrity of the mapped markers was 99.78%. The map spanned 1230.16 cM and the average interval between markers was 0.31 cM. The largest gap between markers was 5.52 cM in LG07 (Supplementary Fig. S2 and Table 2). Ninety-one markers exhibited distorted segregation, with 0.17% being singletons and 2.22% missing data. The collinearity between the genetic map and the reference genome was high.

**Table 2:** Basic information regarding the red radish genetic map

Linkage group ID	Total markers	Total distance (cM)	Average distance (cM)	Max. gap (cM)	Gap <5 cM (%)	Total segregation distortion	Singletons (%)	Missing (%)
LG01	404	143.72	0.36	3.26	100.00	63	0.00	0.41
LG02	446	95.47	0.21	2.05	100.00	3	0.01	0.00
LG03	212	104.58	0.50	4.46	100.00	0	0.00	0.38
LG04	562	148.54	0.26	3.56	100.00	7	0.02	0.00
LG05	656	183.98	0.28	5.22	99.85	0	0.00	0.63
LG06	869	171.91	0.20	2.55	100.00	18	0.02	0.04
LG07	317	119.31	0.38	5.52	99.68	0	0.01	0.21
LG08	254	115.94	0.46	3.91	100.00	0	0.03	0.41
LG09	312	146.71	0.47	3.91	100.00	0	0.08	0.14
Total	4032	1230.16	0.31	5.52	99.95	91		

### Analyses of QTL and the pigment accumulation trait of radish

**Figure 3:**
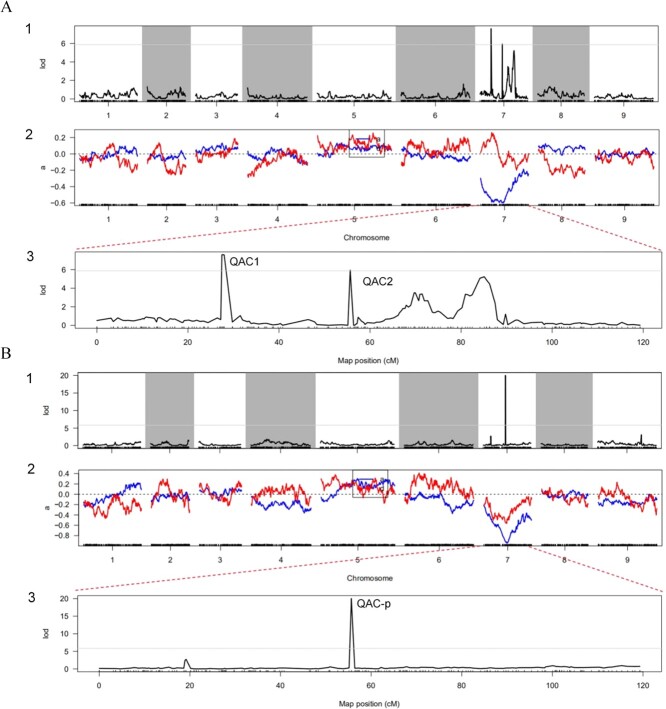
Locations of color-type and root skin anthocyanin content trait on genetic linkage map. (**a**1) LOD scores for the variation in the color-type trait along nine LGs. (**a**2) Phenotypic contribution rate along the nine linkage groups for variation of the color-type trait. (**a**3) LOD scores for the variation in the color-type trait along the seventh LG. (**b**1) LOD scores for variation in the anthocyanin content trait of root skin along nine LGs. (**b**2) Phenotypic contribution rate along the nine LGs for the variation in the anthocyanin content trait of root skin. (**b**3) LOD scores for variation in the anthocyanin content trait of root skin along the seventh LG. In **a**1, **a**3, **b**1 and **b**3, the horizontal ordinate presents the order of the markers in the linkage group; the vertical ordinate presents the LOD values; curves in the plot indicate the genetic coordinate and the LOD score of the detected QTL; the gray line indicates the threshold; the area above the threshold is the associated QTL area. In **a**2 and **b**2, the horizontal ordinate presents the order of the markers in the linkage group; the vertical ordinate presents the contribution rate; ‘a’ (the blue curve) indicates the LOD value corresponding to the marker; ‘d’, (the red curve) indicates the phenotypic contribution rate corresponding to the marker; the gray line indicates the threshold.

The maternal and paternal parents had RP and NP vegetative tissues, respectively. On the basis of the high-density genetic map and the phenotypic characterization, the pigmentation-related QTL were mapped using HighMap with the Kosambi mapping function. First, the NP, RP, and PP groups were assigned values of 1, 2, and 3, respectively. Subsequently, using the values for 200 F_2_ individuals, a joint analysis (LOD ≥ 5.867) detected two QTLs, QAC1 and QAC2, on LG07 (Fig. 3a, Supplementary Table S2). QAC1 (LOD = 7.60) explained 40.853% of the phenotypic variation, and was mapped to a region between Marker748511 and Marker748922, spanning a genetic distance of ~0.50 cM and a physical distance of ~0.29 Mb. QAC2 (LOD = 5.937) explained 39.763% of the phenotypic variation, and was mapped to a region between Marker754365 and Marker754368, spanning a physical distance of 210 bp.

To locate the pigmentation-related QTL more precisely, the location of the QTL related to the synthesis of pigments was determined on the basis of the high-density genetic map and the data for individual anthocyanin contents. More specifically, with the LOD threshold corresponding to the 0.99 confidence level, only one QTL, named QAC-p, which explained 39.414% of the phenotypic variation, was detected on LG07 (Fig. 3b, Supplementary Table S3). However, QAC-p was mapped to a region between Marker754365 and Marker754368, which was just within the scope of QAC2. These results indicated that QAC2 and QAC-p were localized to the same locus.

### Locus analysis and candidate gene prediction

**Figure 4:**
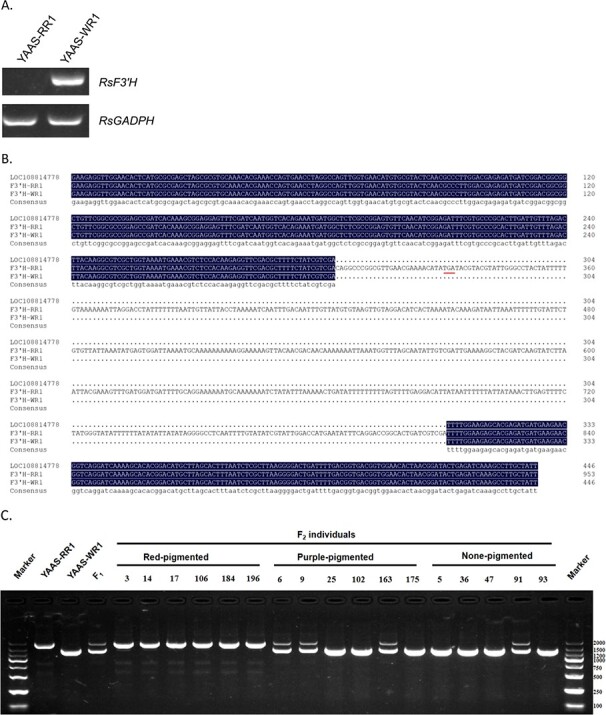
Analysis of *RsF3′H* as a candidate gene. (**a**) Expression patterns of *RsF3′H* based on semiquantitative PCR analysis of the two parental lines. (**b**) Alignment of the second exon of *RsF3′H* sequences from white and red radishes. LOC108814778 is the *RsF3′H* allele in the reference genome. ‘.’ indicates a missing base. The premature termination codon is indicated with a red line. (**c**) Genotypes of the F_2_ individuals for the PCR markers. Numbers represent the different F_2_ individuals used for constructing the genetic map.

An examination of the *R. sativus* L. ‘WK10039’ reference genome (https://www.ncbi.nlm.nih.gov/assembly/GCA_000801105.2) revealed 58 predicted protein-coding genes (Supplementary Table S4) in the QAC1 interval (~0.29 Mb in length with 10 SLAF markers). All but six of these genes were identified and annotated following Swiss-Prot and BLASTX analyses. These genes included *RsF3′H*, which encodes a flavonoid 3′-hydroxylase (F3′H), making it an anthocyanin biosynthesis-related gene. Semiquantitative PCR results confirmed that *RsF3′H* was expressed in YAAS-WR1, but not in YAAS-RR1 (Fig. 4a).

**Figure 5:**
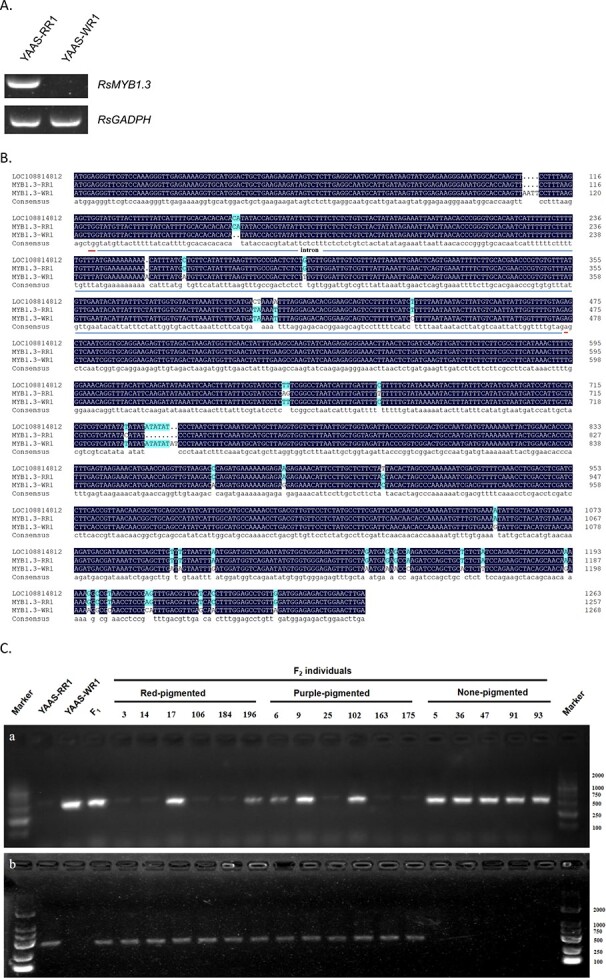
Analysis of *RsMYB1.3* as a candidate gene. (**a**) Expression patterns of *RsMYB1.3* based on the semiquantitative PCR analysis of the two parental lines. (**b**) Alignment of the *RsMYB1.3* sequences from YAAS-RR1 and YAAS-WR1. LOC108814812 is the *RsMYB1.3* allele in the reference genome. ‘.’ represents a missing base. The premature termination codon is indicated with a red line. (**c**) Genotypes of the F_2_ individuals for the PCR markers. Numbers represent the different F_2_ individuals used for constructing the genetic map “a” in the figure above indicates the use of RsMYB1.3-a F/R primer pair and “b” in the figure below indicates the use of RsMYB1.3-b F/R primer pair.

Only one predicted protein-coding gene (Supplementary Table S5) was identified in the QAC2 interval (~280 bp in length with two SLAF markers). The results of the Swiss-Prot and BLASTX analyses indicated that this gene was not related to anthocyanin biosynthesis. Considering that SLAF markers cannot completely cover the entire radish genome, we analyzed the genes within 100 Mb upstream and downstream of the physical locations of the mapped markers. Among these genes, one (annotation number 37239) was predicted to belong to the R2R3 MYB transcription factor family. An analysis of homology indicated that this transcription factor gene is a homolog of *AtPAP1*, which is involved in regulating the anthocyanin biosynthesis pathway. Because the radish genome includes four *AtPAP1* homologs, this gene was named *RsMYB1.3* on the basis of its location in the radish genome. Semiquantitative PCR results proved that the gene was expressed in YAAS-RR1, but not in YAAS-WR1 (Fig. 5a). Accordingly, *RsMYB1.3* might be a suitable candidate gene for *RsPi*.

### Analysis of the candidate genes

To confirm the linkage between the candidate genes (*RsF3′H* and *RsMYB1.3*) and trait segregation, the differences in these two genes between the parents were investigated. First, we compared their genomic structures. As previously described by Masukawa *et al*. [41], a 500-bp fragment was amplified using the P1/P2 primer pair for both YAAS-RR1 and YAAS-WR1. When the RsF3′H-E2-F/RsF3′H-gDNA-R primer pair was used for the PCR amplification, ~1360- and 1867-bp amplified products were obtained for YAAS-WR1 and YAAS-RR1, respectively. The results of sequencing analysis revealed that an insertion of a 507-bp fragment existed in the second exon of *RsF3′H* in YAAS-RR1, which introduced a premature termination codon. Amplified electrophoretic bands indicated that the inserted fragment was heterozygous in the F_1_ plants, suggesting it may be useful as a marker to distinguish genotypes (*F3′H/F3′H*, *f3′h/f3′h*, and *F3′H/f3′h*) (Fig. 4b). It was subsequently used for screening the *RsF3′H* genotypes of 200 F_2_ individuals with phenotypic data. The results revealed a 1:2:1 Mendelian ratio for the *F3′H* genotype in the F_2_ generation. All of the red individuals had a genotype consistent with that of YAAS-RR1 (*f3′h/f3′h*). The purple population comprised the *F3′H/F3′H* and *F3′H/f3′h* genotypes, whereas the non-pigmented population included the *F3′H/F3′H*, *f3′h/f3′h*, and *F3′H/f3′h* genotypes. However, obvious differences were observed in the pigment contents of individuals with the *RsF3′H* genotype, which indicated that *RsF3′H* genotypes were not related to pigmentation grades (Fig. 4c).

An examination of the *RsMYB1.3* homologs in the two parental lines detected 33 SNP differences and 4 InDel differences. By comparing the coding sequence with the gDNA sequence, we detected a 4-bp insertion in the first exon that introduced a premature termination codon in the *RsMYB1.3* homolog of YAAS-WR1 (Fig. 5b). Two pairs of PCR primers, RsMYB1.3-a F/R and RsMYB1.3-b F/R, were designed on the basis of the sequence differences between these two haplotypes. A 511-bp target fragment was generated from the *MYB1.3*-YAAS-WR1 homolog using the RsMYB1.3-a F/R primer pair, whereas a 467-bp target fragment was obtained from the *MYB1.3*-YAAS-RR1 homolog using the RsMYB1.3-b F/R primer pair. In contrast, both fragments were amplified in the F_1_ plants, indicating that *RSMYB1.3* in the F_1_ plants was heterozygous. These two primer pairs were used for genotyping the 200 F_2_ individuals. The results proved that all of the NP individuals had a genotype consistent with that of YAAS-WR1 (*myb1.3*/*myb1.3*), whereas the purple or red individuals had *MYB1.3*/*MYB1.3* or *MYB1.3*/*myb1.3* genotypes, which revealed a 1:2:1 Mendelian ratio for the *RsMYB1.3* genotype in the F_2_ generation (Fig. 5c).

A natural population was screened for the presence of the 4-bp insertion in the first exon of *RsMYB1.3-*WR1. Specifically, we analyzed 68 radish accessions of varying root skin and root flesh colors collected from various regions. The PCR using the RsMYB1.3-a F/R primer pair did not amplify the target fragment for radish accessions containing red or purple pigment. However, a 511-bp fragment was detected for all varieties lacking anthocyanins in the vegetative tissue (Supplementary Table S7). Therefore, the 4-bp insertion appears to be widely distributed in radish varieties that do not produce anthocyanins. These findings suggested that *RsMYB1.3* is the key gene for determining whether radish can synthesize and accumulate anthocyanins.

The above results imply that *RsF3′H* is the *RsPP* gene controlling the purple trait, whereas *RsMYB1.3* is the *RsPi* gene controlling coloration.

### RsMYB1.3 interacts with RsTT8 and promotes the expression of *RsTT8* and *RsUFGT*

**Figure 6:**
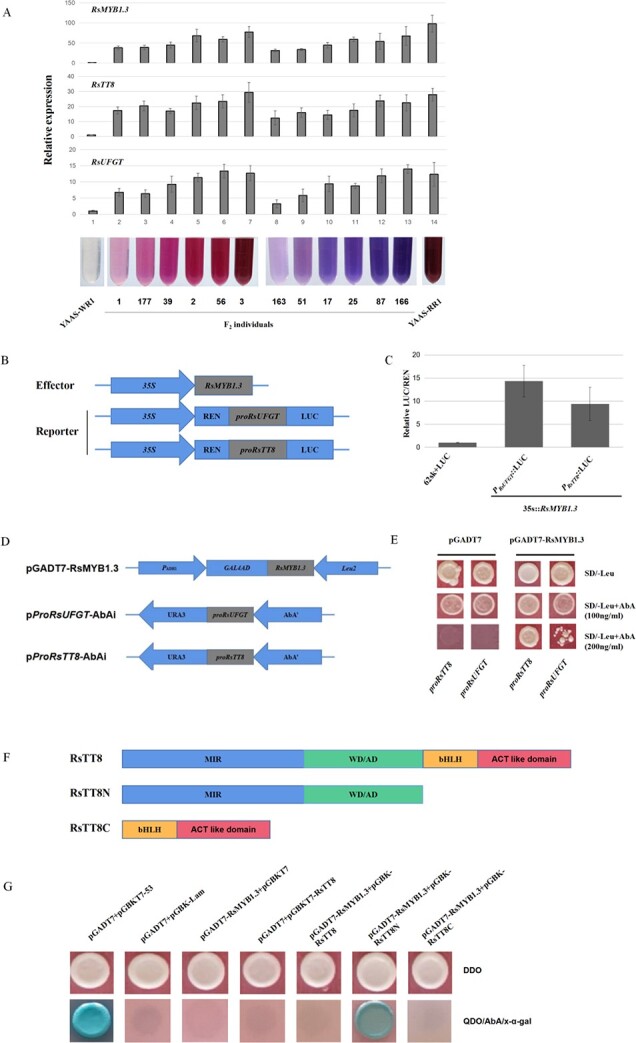
RsMYB1.3 promotes the expression of *RsTT8* and *RsUFGT* and interacts with RsTT8. (**a**) Relative expressions of *RsMYB1.3*, *RsTT8* and *RsUFGT* in the flesh of taproots with different anthocyanin contents as determined by qPCR analysis. Numbers represent the different F_2_ individuals used to construct the genetic population. (**b**) Schematic of the effector/reporter constructs for dual-luciferase assays. (**c**) *In vivo* interaction between RsMYB1.3 and the *RsTT8* and *RsUFGT* promoters according to dual-luciferase assays. (**d**) Construct details for the Y1H assay. (**e**) Y1H assay results for the binding between RsMYB1.3 and anthocyanin synthesis-related gene promoters (*RsUFGT* and *RsTT8*); the empty pGADT7 vector was used as the negative control. (**f**) Details of the *RsTT8* constructs used for Y2H assays. (**g**) Y2H assay results for binding between RsMYB1.3 and RsTT8 fragments.

The transcription of *RsMYB1.3* and the genes related to anthocyanin synthesis in the parents and F_2_ individuals with differing pigmentation was analyzed by qPCR. The results indicated that the *RsMYB1.3* expression level was significantly higher in pigmented individuals than in YAAS-WR1. In the red and purple populations, the *RsMYB1.3* expression level increased as anthocyanin content increased (Fig. 6a). The *RsTT8* and *RsUFGT* expression trends were consistent with that of *RsMYB1.3*.

To assess the ability of RsMYB1.3 to activate the *RsTT8* and *RsUFGT* promoters, transient dual-luciferase assays were conducted using the constructs presented in Fig. 6b. The assay results demonstrated that, compared with the negative control, RsMYB1.3 increased the activities of the *RsUFGT* and *RsTT8* promoters by 14.3- and 9.4-fold, respectively (Fig. 6c). The binding of RsMYB1.3 to *RsUFGT* and *RsTT8* was further confirmed by a yeast one-hybrid (Y1H) assay (Fig. 6d and e).

The expression of genes related to anthocyanin biosynthesis is regulated by the MBW complex. To investigate the interaction between RsMYB1.3 and RsTT8, the ability of RsMYB1.3 to bind to different regions of RsTT8 was evaluated using a yeast two-hybrid (Y2H) system (Fig. 6f). Yeast cell colonies carrying RsMYB1.3 and the RsTT8 N-terminal region (including the MIR and WD/AD domains) grew on QDO/aureobasidin A (AbA)/X-α-gal medium. In contrast, cells carrying RsMYB1.3/RsTT8 or RsMYB1.3/RsTT8C were unable to grow on QDO/AbA/X-α-gal medium (Fig. 6g). Accordingly, the RsTT8 N-terminal region is essential for the interaction between RsTT8 and RsMYB1.3, but the C-terminal region of RsTT8 appears to inhibit the interaction.

Thus, these results suggested that *RsMYB1.3* could affect anthocyanin synthesis in radish by regulating the expression of *RsTT8* and *RsUFGT*.

## Discussion

The SLAF-seq strategy, which is based on high-throughput sequencing technologies, was developed as a simplified genome sequencing method [33]. The advantages of using SLAF-seq technology rather than other available methods to construct genetic maps and for mapping QTL include the detection of more markers, a higher map quality, a faster protocol, and greater data utility. In this study, we used SLAF-seq technology for a simplified genome sequencing of 200 F_2_ plants. A total of 4557 polymorphic markers covering 70% of the progeny were used to construct a genetic map. Linkage and collinearity analyses confirmed that the sequence of most markers in each LG was consistent with the genomic sequence, reflecting good collinearity and the accuracy of the calculated genetic recombination rate. Sufficient molecular markers and population sizes as well as highly efficient genotyping approaches may enhance QTL mapping resolution and ultimately improve map-based cloning. To date, several radish genetic maps have been constructed [42–44]. However, the marker density in the present linkage map is significantly higher than that of most of the available radish genetic maps. Although a high-density radish genetic map was recently established by genome resequencing, the map is not applicable to the genetic analysis of anthocyanin accumulation-related traits of radish root flesh because both parents had white root flesh [31].

The coloration of the fleshy taproot, including the skin and flesh, is an important process influencing the appearance and nutritional quality of the radish, with implications for production and consumption. Possible inheritance patterns for radish taproot color have been examined in numerous studies. Early reports suggested that the root exterior color might be controlled by a single locus comprising multiple alleles, the combination of which determines the pigmentation of the root exterior [45, 46]. In subsequent investigations involving hybridizations between red and white turnip lines, all of the F_1_ hybrids had a purple root, whereas the F_2_ population segregated into three color groups—purple, red, and white—with a 9:3:(3 + 1) ratio [47, 48]. These findings suggest that root coloration is controlled by two genetic loci. In the current study, regardless of the distribution of anthocyanins and the anthocyanin contents, the purple, red, and white coloration of the F_2_ population derived from a cross between YAAS-RR1 and YAAS-WR1 segregated in a 9:3:(3 + 1) ratio. The segregation was consistent with a 3:1 Mendelian segregation ratio for pigmented individuals and NP individuals. Accordingly, the PiN and PP/PR traits are each controlled by one locus. Additionally, the PiN-related gene is dominant homozygous in YAAS-RR1 and the PP/PR-related gene is dominant homozygous in YAAS-WR1. Phenotypic analyses demonstrated that PiN has an epistatic effect on PP/PR.

Earlier research proved that the proportions of pelargonidin and cyanidin affect radish root color, with pelargonidin and cyanidin mainly associated with red and purple pigmentation, respectively [49]. In the anthocyanin synthesis pathway of plants, the difference between pelargonidin and cyanidin is due to the presence of a hydroxyl group (anthocyanin) or a non-hydroxyl group (aspartate) at the 3′ position of the flavonoid B-ring. Additionally, F3′H catalyzes the 3′ hydroxylation of dihydrokaempferol, a precursor of pelargonidin, to produce dihydroquercetin, leading to the biosynthesis of cyanidin. Consequently, a genetic mutation resulting in a lack of F3′H may be responsible for the red or purple coloration of radish. A recent study determined that the insertion of a Gypsy/Ty3 retrotransposon in the first exon of the *F3*′*H* homologous gene in radish results in a loss-of-function mutation [41]. However, we did not detect Gypsy/Ty3 in YAAS-RR1 and YAAS-WR1. Instead, a 507-bp fragment inserted into the second exon of *RsF3′H* in YAAS-RR1 was identified in the current study. The premature termination codon introduced by this insertion results in a lack of functional *RsF3′H*. Considering the expression of *RsF3*′*H* and the tight linkage between the marker and purple/red root color phenotype, it is highly convincing that *RsF3*′*H* is the *RsPP* related to the PP/PR trait. At least two explanations exist for the loss of F3′H functions in different radish varieties, which may be related to the convergent evolution of the radish varieties.

The MYB transcription factors, especially the R2R3-MYB members, are important regulators of the anthocyanin biosynthesis pathway. In *Arabidopsis thaliana*, the R2R3-MYB transcription factor genes, including *AtMYB75* (*AtPAP1*), *AtMYB90* (*AtPAP2*), *AtMYB113*, and *AtMYB114*, encode proteins with highly conserved amino acid sequences and are involved in regulating anthocyanin contents [50]. The color of plant organs may change because of natural mutations to R2R3-MYB genes (e.g. SNPs, InDels, and transposon insertions) as well as epigenetic changes to the MYB gene promoter (e.g. methylation) [51, 52]. So far, the R3R3-MYBs, which are responsible for anthocyanin synthesis, have been found in crops such as apple [53–55], eggplant [56], cauliflower [57, 58], and Chinese cabbage [59]. The following four *AtPAP1* homologs were identified in the ‘WK10039’ radish genome: *RsMYB1.1* (Gene4623, LOC108832642), *RsMYB1.2* (Gene4694, LOC108840410), *RsMYB1.3* (Gene37656, LOC108814812), and *RsMYB1.4* (Gene38063, LOC108816293). With the exception of *RsMYB1.1*, these genes are involved in anthocyanin synthesis. For example, *RsMYB1.2*, which is located on chromosome 2, contributes to the anthocyanin accumulation in radish inbred lines cx16Q-25-2 [28] and NAU-067 [60]. Additionally, *RsMYB1.3* is crucial for the accumulation of anthocyanins in red radish skin [31, 49]. The *RsMYB1.4* gene was first identified in the radish variety ‘Bordeaux’ [29]. A subsequent transcriptome sequencing analysis proved that this gene is closely related to anthocyanin synthesis in ‘Xinlimei’ radish [28]. A recent report suggested that a transposon insertion inducing the methylation of the *RsMYB1.4* promoter inhibits anthocyanin accumulation in ‘Xinlimei’ radish [61]. Thus, more than one MYB transcription factor regulates anthocyanin synthesis in radish. The results of the current study imply that the anthocyanin contents of YAAS-RR1 root skin and flesh are primarily regulated by the same genetic locus (QAC2). Several analyses (i.e. gene structure, expression, and function as well as genetic linkage) confirmed *RsMYB1.3* as the candidate gene for QAC2. In radish inbred line YAAS-WR1, a 4-bp insertion in the first exon of *RsMYB1.3* results in a premature termination codon that prevents the production of a functional RsMYB1.3. Because the *RsMYB1.3* and *RsMYB1.4* sequences are very similar, the expression levels of these two genes could not be determined by qPCR. However, the *RsMYB1.3* coding sequence in YAAS-RR1 seedlings was isolated by reverse transcription PCR, but *RsMYB1.4* was not amplified. Thus, *RsMYB1.3* rather than *RsMYB1.4* is involved in anthocyanin synthesis during the early developmental stage of YAAS-RR1. The expression of *RsMYB1.3* and the tight linkage between the marker and the pigmented/non-pigmented root phenotype provide convincing evidence that *RsMYB1.3* is the PiN-related gene.

Although the homologues of *RsMYB1.3* in some radish varieties are related to red coloration, their functions have not been thoroughly investigated [31, 49]. In this study, we observed that *RsMYB1.3*, *RsTT8*, and *RsUFGT* were similarly expressed in different individuals in the F_2_ population. Additionally, their expression levels increased as the anthocyanin content increased. The Y1H and dual-luciferase assays demonstrated that RsMYB1.3 can bind directly to the promoters of the anthocyanin biosynthesis-related genes *RsTT8* and *RsUFGT* to promote expression. The confirmed interaction between RsMYB1.3 and RsTT8 proved that RsMYB1.3 is an important component of the MBW complex responsible for anthocyanin synthesis in radish (Fig. 6). The diversity of the MYB transcription factors in different radish varieties [28, 31, 61] may reflect differences in the mechanisms underlying anthocyanin accumulation among radish varieties. The comprehensive regulatory effects of *RsMYB1.2*, *RsMYB1.3*, and *RsMYB1.4* on anthocyanin synthesis in radish, including the possible mutual regulation among MYBs, will need to be more precisely characterized in future investigations.

Radish root coloration appears to be largely determined by genetic factors [45, 46, 62], while environmental factors such as light and biotic/abiotic stresses can also affect anthocyanin synthesis in plants. In this study, we observed that all individuals of the F_2_ population with pigmented root flesh had anthocyanins in their root skin, whereas some individuals with pigmented root skin had no anthocyanins in their root flesh. This result indicated that the accumulation of anthocyanins in different tissues was controlled by different mechanisms. Therefore, although *RsMYB1.3* controls anthocyanin synthesis in radish vegetative tissues, it may be differentially regulated or modified in diverse tissues, resulting in inconsistent coloration of different tissues. Hence, the molecular mechanism underlying anthocyanin accumulation in the fleshy roots of Yunnan red radish will need to be more thoroughly investigated, especially regarding the regulation of *RsMYB1.3* expression and the modification of the encoded protein.

## Materials and methods

### Plant materials

Two radish inbred lines were used in this study: YAAS-RR1, with vegetative tissues that accumulated pigment (red taproot skin and flesh, red veins, and red stem), was derived from the Yunnan red radish landrace, whereas YAAS-WR1, with vegetative tissues lacking pigment (white taproot skin and flesh, green veins, and green stem), was derived from Japanese commercial germplasm. The YAAS-RR1 (P_1_) and YAAS-WR1 (P_2_) lines were crossed to produce the F_1_, BC_1_P_1_, BC_1_P_2_, and F_2_, populations. Two F_2_ populations produced from the same hybridization were grown in 2018 and 2019 for an investigation of the agronomic traits of each individual. A total of 200 F_2_ individuals grown in 2018 were used for genetic map construction and trait association analysis. All plants were grown in the greenhouse of the Horticultural Research Institute, Yunnan Academy of Agricultural Sciences. Seeds were sown in the ridges of hilled rows (0.2 m width and 0.3 m height; 0.3 m separation of rows and columns).

### Phenotyping and DNA extraction

The goodness of fit of the segregation ratios in the BC_1_P_1,_ BC_1_P_2_ and F_2_ populations was evaluated by χ^2^ tests in the genetic analysis. Regarding the F_2_ population, the pigmented population was further segregated into red and purple populations. The non-pigmented F_2_ population, red F_2_ population, and purple F_2_ population were scored as first, second, and third grades, respectively.

At 100 days after sowing, anthocyanins of 0.5 g finely ground tissues (root skin, petiole, and root flesh) were extracted from the YAAS-WR1, YAAS-RR1, and 200 F_2_ individuals (the population used for constructing the genetic map) according to Chu’s method [38]. Total anthocyanin contents were calculated on the basis of the absorbances of the extracts at 530 and 657 nm. The following formula was used for quantifying the anthocyanin content: anthocyanin content (*Q*) = (*A*530–0.25 × *A*657) × *M*^−1^, where *A*530 and *A*657 are the absorbances at 530 nm and 657 nm, respectively, and *M* is the sample weight. Each sample was analyzed in triplicate, with three biological replicates.

Young radish leaves were frozen with liquid nitrogen and then ground into a fine powder. The cetyltrimethylammonium bromide (CTAB) method [39] was used to extract genomic DNA from leaf powder (100 mg) of each plant. The DNA concentrations and quality were evaluated using an NP80 ultraviolet spectrophotometer (Implen, Germany) and by 1% agarose gel electrophoresis.

### Construction of SLAF library and sequencing

In this study, an improved SLAF-seq strategy was used. First, for the *in silico* prediction of the number of markers produced by different enzymes, marker-discovery experiments were designed by analyzing the ‘WK10039’ radish reference genome (https://www.ncbi.nlm.nih.gov/assembly/GCA_000801105.2). The genomic DNA of the samples included in the SLAF pilot experiment was digested with Hpy166II and HaeIII (New England Biolabs, Beverly, MA, USA). Dual-index sequencing adapters were ligated to the digested fragments using T4 ligase (New England Biolabs). PCR amplifications were performed using appropriate concentrations of the prepared DNA samples. Agencourt AMPure XP beads (Beckman Coulter, High Wycombe, UK) were used to enrich the PCR products, which were 364–464 bp long (with sequencing adapters). Finally, diluted gel-purified products were sequenced using the Illumina HiSeq 2500 system (Illumina, Inc., San Diego, CA, USA) to generate 125-bp paired-end reads. The sequencing was performed by Biomarker Technologies Corporation (Beijing, China).

### Sequence data grouping and genotyping

SLAF marker identification and genotyping were performed using procedures described by Sun *et al*. [27]. Briefly, low-quality reads (quality score <20e) were eliminated, after which the raw reads were assigned to each progeny according to the duplex barcode sequences. After the barcodes and the terminal 5-bp fragments were trimmed from each high-quality read, the clean reads for each sample were mapped onto the ‘WK10039’ genome sequence using the SOAP software. Sequences mapped to the same position were considered to belong to the same SLAF locus. The single-nucleotide polymorphisms (SNPs) between parents at each SLAF locus were then detected, and SLAF loci with more than three SNPs were eliminated. The alleles at each SLAF locus were then defined on the basis of the parental reads, with a sequence depth >20-fold. For each offspring, the reads with a sequence depth >5-fold were used to define alleles. One SLAF locus can contain up to four genotypes in diploid species. Thus, only SLAF loci with two to four alleles should be identified as polymorphic and considered as potential markers; those with more than four alleles were designated as repetitive markers and discarded. All polymorphic SLAF loci were genotyped. The SNP loci of the parents and progeny should be consistent. The polymorphic markers were analyzed on the basis of the F_2_ population type (aa × bb).

### Linkage map construction and candidate gene identification

A newly developed HighMap strategy was used to correctly order the SLAF markers and fix genotyping errors within LGs in this study. All selected SLAF markers were assigned to one of nine LGs on the basis of their position on the chromosome. The recombination percentage was converted into a genetic distance (cM) by using the Kosambi mapping function. The map quality was evaluated on the basis of haplotype diagrams and heat maps, which were prepared by Biomarker Technologies Corporation (Beijing, China) using Draw_haplotype-map. The R/qtl software was used for analyzing QTL. Automatic cofactor selection (reverse elimination, *P* < .05) was applied as a marker of significant correlations for detecting cofactors. At the *P* < .05 level, the LOD significance threshold was determined on the basis of 1000 permutations. The location of each QTL was determined on the basis of the LOD peak location of each QTL and the surrounding area. At the highest probability peak, the percentage of the phenotypic variation explained by a QTL (R2) was estimated. The annotated *R. sativus* L. genome (http://brassicadb.org/brad/) was referred to for the annotation of candidate genes. The functions of the predicted genes were determined by screening the Swiss-Prot database using the BLASTX algorithm.

To amplify candidate genes by PCR, Primer3web was used to design specific primers based on the DNA sequences extracted from the radish genome [40]. PCR amplification was performed using Q5 High-Fidelity DNA Polymerase (New England Biolabs). The two-step cycle procedure recommended by the manufacturer was used in this study. The PCR products were sequenced using the ABI 3730 instrument (Applied Biosystems, CA, USA). The primers used in this study are listed in Supplementary Table S6.

### RNA extraction and quantitative real-time PCR analysis of candidate genes

The MiniBEST Plant RNA Extraction Kit (Takara, Tokyo, Japan) was used to extract total RNA from tissues collected from YAAS-WR1, YAAS-RR1, and various F_2_ individuals of different coloration traits. The poly(A)^+^ RNA was used as the template to synthesize cDNA with the GoScript Reverse Transcription System (Promega, Madison, USA). Details regarding the specific primers were designed using Primer3web [40].

To investigate the potential regulatory roles of candidate genes related to pigment content, we analyzed gene expression in the root flesh of the parental lines and various F_2_ individuals (different degrees of red/purple deposition) in a quantitative real-time PCR (qPCR) assay. The actin gene of radish was used as the internal reference to normalize the gene expression data. To examine the expression of candidate genes, a PCR mix was prepared by combining 1 μl of cDNA (template), SYBR Green PCR Master Mix (TsingKe Biological Technology Co., Beijing, China), and gene-specific primers. qPCR was performed using the StepOnePlus Real-Time PCR System (Applied Biosystems, CA, USA). Relative gene expression levels were calculated in accordance with the 2^−ΔΔCt^ method. The means of three biological replicates were analyzed, with *t*-tests used to assess the significance of any differences.

### Marker development and segregation analysis

Insertion/deletion (InDel) markers were developed on the basis of the differences among the candidate genes in YAAS-WR1 and YAAS-RR1. Primers for the InDel markers were designed using Primer3.0 (https://primer3.ut.ee/) and genome resequencing data. Polymorphic PCR bands were used to detect recombination in the two parental lines, the F_1_ population, 200 F_2_ individuals, and 68 cultivars. PCR was performed in a 50-μl solution containing 100 ng DNA, 25 μl 2× PCR mix (TsingKe Biological Technology Co.), 1 μl of each primer (10 μM), and double-distilled water. PCR amplification was completed in the Biometra instrument (Bio-Rad Laboratories, USA) using the following program: 95°C for 5 min; 35 cycles of 95°C for 30 s, an appropriate annealing temperature for 30 s, and 72°C for an appropriate time; and 72°C for 5 min. The amplified fragments were visualized with ethidium bromide in a 1.5% agarose gel.

### Yeast two-hybrid assay

The pGADT7 vector, which contains the GAL4 activation domain, and the pGBKT7 vector, which contains the GAL4 DNA-binding domain, were used for the Y2H assay. The full-length RsMYB1.3 coding sequence (CDS) was cloned into pGADT7 vector to produce the pGADT7-RsMYB1.3 recombinant plasmid. The full-length *RsTT8* CDS, the N-terminal fragment (including MIA and WD/AD) of the *RsTT8* CDS and the C-terminal fragment (including bHLH and ACT-like) of the *RsTT8* CDS were cloned into separate pGBKT7 vectors to produce pGBKT7-RsTT8, pGBKT7-RsTT8N and pGBKT7-RsTT8C recombinant plasmids. Next, PGADT7-RSMYB1.3 was paired with recombinant pGBKT7 plasmids containing different *RsTT8* fragments for the cotransformation of Y2HGold yeast cells, which were screened on SD medium lacking Trp and Leu (DDO). Transformants were then screened on SD medium lacking Trp, Leu, His, and Ade, but supplemented with 125 mg/ml AbA (Coolaber, Beijing, China) and 0.02 mg/ml X-α-gal (QDO/AbA/X-α-Gal) (Coolaber). After 3 days of incubation at 30°C, colony growth was observed and scored. The primers used for the Y2H assay are listed in Supplementary Table S6.

### Dual-luciferase assay

The full-length *RsMYB1.3* CDS was cloned into the pGreenII 62-SK vector. The *RsTT8* and *RsUFGT* promoter regions containing MYB-binding elements were cloned into separate pGreenII 0800-LUC vectors. The resulting recombinant plasmids were inserted into *Agrobacterium tumefaciens* strain GV3101 cells, which were then cultured. Culture medium containing cells carrying *RsMYB1.3* was mixed with culture medium containing cells carrying the promoter fragments in a 10:1 ratio. The mixture was infiltrated into the leaves of 6-week-old *Nicotiana benthamiana* plants using needleless syringes. After 3 days, the Dual Luciferase Reporter Gene Assay Kit (Yeasen, Shanghai, China) was used to analyze the activities of luciferase (LUC) and Renilla-luc (REN). The primers used for the dual-luciferase assay are listed in Supplementary Table S6.

### Yeast one-hybrid assay

The *RsTT8* and *RsUFGT* promoter regions containing MYB-binding elements were cloned into separate pAbAi vectors to generate bait constructs. The bait constructs were linearized by BstB1 digestion and then inserted into Y1HGold yeast cells. The transformed cells were used to determine the minimal inhibitory concentrations of AbA. The pGADT7-RsMYB1.3 plasmid was introduced into the yeast Y1HGold cells containing the bait constructs. The cells were then screened on medium lacking Leu (SD/−Leu), but supplemented with the optimal AbA concentration. After 3–5 days of incubation at 30°C, colony growth was observed and scored. The primers used for the Y1H assay are listed in Supplementary Table S6.

## Supplementary Material

Web_Material_uhab031

## Data Availability

The raw genome resequencing data are available in the NCBI database (accession number PRJNA748175). The markers in the genetic map presented herein are listed in Supplementary Tables S2 and S3. The raw sequences of the 200 F_2_ individuals analyzed in this study are available from Y.S.
