# Supplementary material for: Construction of a high-density genetic map based on specific-locus amplified fragment sequencing and identification of loci controlling anthocyanin pigmentation in Yunnan red radish
Source: Hortic Res. 2022 Feb 10;9:uhab031. doi: 10.1093/hr/uhab031 (PMC8829420; doi:10.1093/hr/uhab031)
Supplement: Web_Material_uhab031 [file web_material_uhab031.zip › supplementary figures.docx]

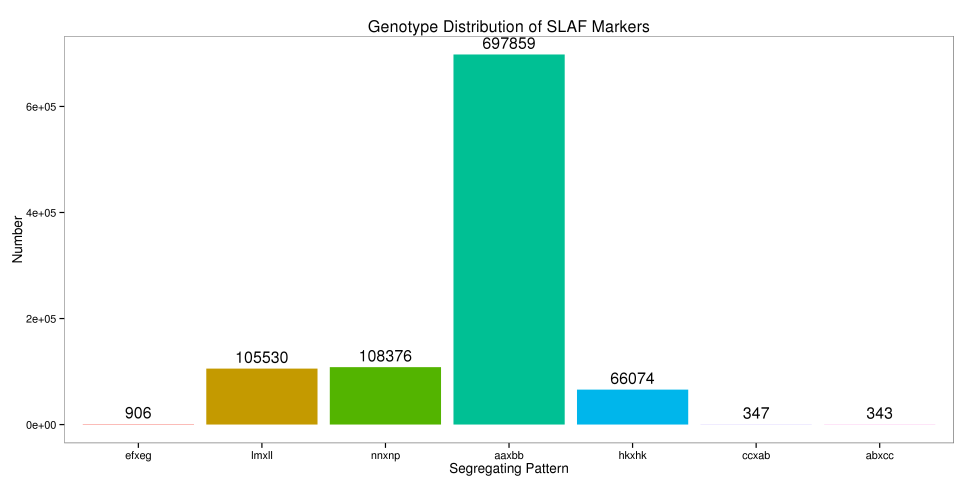


Figure S1. Number of SLAF markers for seven segregation patterns.


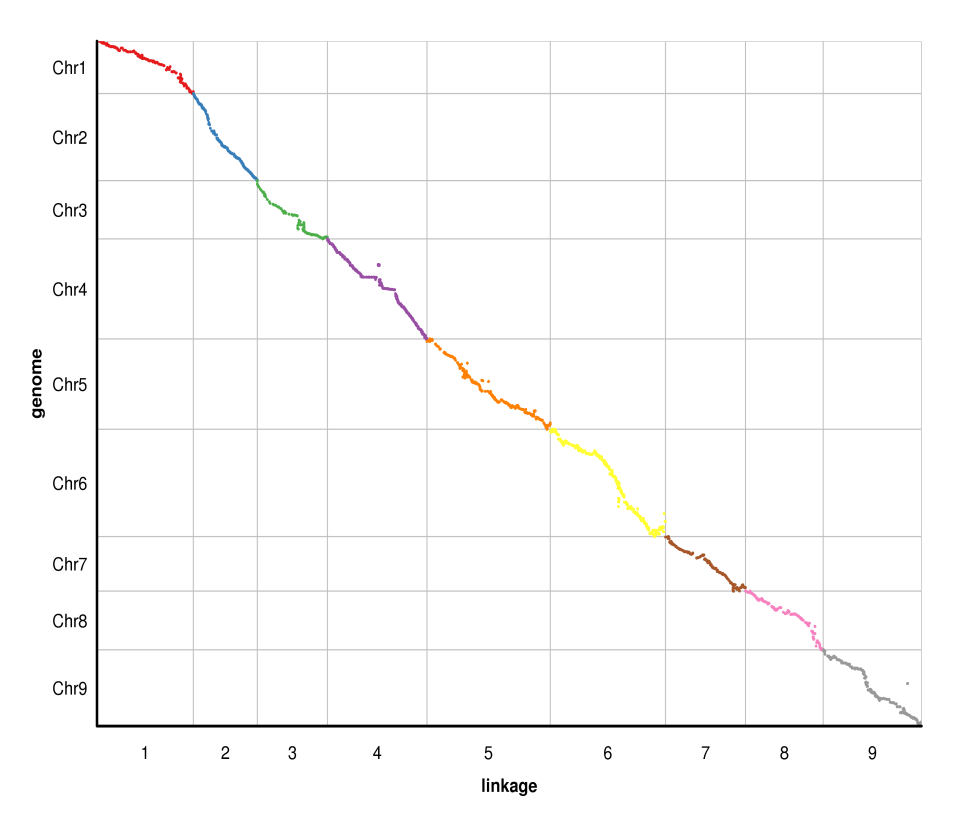


Figure S2. Collinearity mapping of nine linkage groups in radish to the *Raphanus sativus* L. reference genome sequence.
