# Supplementary material for: Construction of a high-density genetic map based on specific-locus amplified fragment sequencing and identification of loci controlling anthocyanin pigmentation in Yunnan red radish
Source: Hortic Res. 2022 Feb 10;9:uhab031. doi: 10.1093/hr/uhab031 (PMC8829420; doi:10.1093/hr/uhab031)
Supplement: Web_Material_uhab031 [file web_material_uhab031.zip › supplementary tables.docx]

Table S1. Phenotypic datasets of pigment content of radish root skin/flesh

| Number | Test1 of root skin  (A530-0.25*A657)*g-1Fw | Test2 of root skin  (A530-0.25*A657)*g-1Fw | Test3 of root skin  (A530-0.25*A657)*g-1Fw | Mean value | Standard deviation （±） | Color grades# |
| --- | --- | --- | --- | --- | --- | --- |
| 1 | 4.66 | 4.21 | 4.68 | 4.52 | 0.22 | 3 |
| 2 | 4.85 | 4.95 | 4.67 | 4.82 | 0.12 | 3 |
| 3 | 5.48 | 5.85 | 5.21 | 5.51 | 0.26 | 2 |
| 4 | 5.99 | 5.78 | 5.96 | 5.91 | 0.09 | 2 |
| 5 | 0.00 | 0.00 | 0.00 | 0.00 | 0.00 | 8 |
| 6 | 5.61 | 5.45 | 5.23 | 5.43 | 0.16 | 2 |
| 7 | 5.63 | 5.85 | 5.42 | 5.63 | 0.18 | 2 |
| 8 | 1.78 | 1.65 | 1.98 | 1.8 | 0.14 | 6 |
| 9 | 5.46 | 5.32 | 5.88 | 5.55 | 0.24 | 2 |
| 10 | 4.78 | 4.65 | 4.78 | 4.74 | 0.06 | 3 |
| 11 | 3.65 | 3.85 | 3.42 | 3.64 | 0.18 | 4 |
| 12 | 5.81 | 5.88 | 5.69 | 5.79 | 0.08 | 2 |
| 13 | 3.52 | 3.46 | 3.62 | 3.53 | 0.07 | 4 |
| 14 | 4.16 | 4.19 | 4.27 | 4.21 | 0.05 | 3 |
| 15 | 0.45 | 0.52 | 0.43 | 0.47 | 0.04 | 7 |
| 16 | 4.23 | 4.36 | 4.21 | 4.27 | 0.07 | 3 |
| 17 | 6.78 | 6.67 | 6.75 | 6.73 | 0.05 | 1 |
| 18 | 4.85 | 4.85 | 4.92 | 4.87 | 0.03 | 3 |
| 19 | 4.62 | 4.55 | 4.71 | 4.63 | 0.07 | 3 |
| 20 | 3.45 | 3.52 | 3.32 | 3.43 | 0.08 | 4 |
| 21 | 4.13 | 4.16 | 4.08 | 4.12 | 0.03 | 3 |
| 22 | 3.25 | 3.29 | 3.34 | 3.29 | 0.04 | 4 |
| 23 | 4.32 | 4.42 | 4.35 | 4.36 | 0.04 | 3 |
| 24 | 1.11 | 1.17 | 1.26 | 1.18 | 0.06 | 6 |
| 25 | 5.22 | 5.34 | 5.26 | 5.27 | 0.05 | 2 |
| 26 | 4.36 | 4.45 | 4.26 | 4.36 | 0.08 | 3 |
| 27 | 2.78 | 2.65 | 2.70 | 2.71 | 0.05 | 5 |
| 28 | 0.55 | 0.49 | 0.48 | 0.51 | 0.03 | 7 |
| 29 | 0.25 | 0.24 | 0.27 | 0.25 | 0.01 | 7 |
| 30 | 5.46 | 5.48 | 5.39 | 5.44 | 0.04 | 2 |
| 31 | 1.89 | 1.95 | 1.88 | 1.91 | 0.03 | 6 |
| 32 | 5.79 | 5.68 | 5.46 | 5.64 | 0.14 | 2 |
| 33 | 6.65 | 6.63 | 6.75 | 6.68 | 0.05 | 1 |
| 34 | 0.16 | 0.18 | 0.13 | 0.16 | 0.02 | 7 |
| 35 | 1.52 | 1.56 | 1.59 | 1.56 | 0.03 | 6 |
| 36 | 0.00 | 0.00 | 0.00 | 0.00 | 0.00 | 8 |
| 37 | 4.35 | 4.23 | 4.26 | 4.28 | 0.05 | 3 |
| 38 | 4.15 | 4.32 | 4.29 | 4.25 | 0.07 | 3 |
| 39 | 6.21 | 6.15 | 6.23 | 6.2 | 0.03 | 1 |
| 40 | 3.65 | 3.68 | 3.36 | 3.56 | 0.14 | 4 |
| 41 | 0.35 | 0.52 | 0.39 | 0.42 | 0.07 | 7 |
| 42 | 5.31 | 5.21 | 5.55 | 5.36 | 0.14 | 2 |
| 43 | 5.26 | 5.13 | 5.42 | 5.27 | 0.12 | 2 |
| 44 | 1.32 | 1.46 | 1.20 | 1.33 | 0.11 | 6 |
| 45 | 5.55 | 5.12 | 5.48 | 5.38 | 0.19 | 2 |
| 46 | 6.23 | 6.21 | 6.33 | 6.26 | 0.05 | 1 |
| 47 | 0.00 | 0.00 | 0.00 | 0.00 | 0.00 | 8 |
| 48 | 5.63 | 5.42 | 5.58 | 5.54 | 0.09 | 2 |
| 49 | 6.69 | 6.42 | 6.88 | 6.66 | 0.19 | 1 |
| 50 | 4.11 | 4.32 | 4.61 | 4.35 | 0.2 | 3 |
| 51 | 5.82 | 5.46 | 5.75 | 5.68 | 0.16 | 2 |
| 52 | 0.38 | 0.46 | 0.52 | 0.45 | 0.06 | 7 |
| 53 | 3.22 | 3.51 | 3.46 | 3.40 | 0.13 | 4 |
| 54 | 3.44 | 3.68 | 3.51 | 3.54 | 0.10 | 4 |
| 55 | 4.03 | 4.21 | 4.15 | 4.13 | 0.07 | 3 |
| 56 | 6.33 | 6.12 | 6.49 | 6.31 | 0.15 | 1 |
| 57 | 6.91 | 6.68 | 7.06 | 6.88 | 0.16 | 1 |
| 58 | 2.65 | 2.45 | 2.36 | 2.49 | 0.12 | 5 |
| 59 | 5.91 | 5.46 | 5.78 | 5.72 | 0.19 | 2 |
| 60 | 6.51 | 6.24 | 6.85 | 6.53 | 0.25 | 1 |
| 61 | 6.43 | 6.54 | 6.53 | 6.5 | 0.05 | 1 |
| 62 | 0.15 | 0.18 | 0.32 | 0.22 | 0.07 | 7 |
| 63 | 1.52 | 1.58 | 1.46 | 1.52 | 0.05 | 6 |
| 64 | 5.29 | 5.61 | 5.24 | 5.38 | 0.16 | 2 |
| 65 | 5.46 | 5.50 | 5.39 | 5.45 | 0.05 | 2 |
| 66 | 5.46 | 5.88 | 0.52 | 3.95 | 2.43 | 4 |
| 67 | 4.61 | 4.63 | 4.81 | 4.68 | 0.09 | 3 |
| 68 | 0.26 | 0.29 | 0.33 | 0.29 | 0.03 | 7 |
| 69 | 5.19 | 5.20 | 5.46 | 5.28 | 0.12 | 2 |
| 70 | 5.36 | 5.26 | 5.16 | 5.26 | 0.08 | 2 |
| 71 | 1.42 | 1.64 | 1.52 | 1.53 | 0.09 | 6 |
| 72 | 4.25 | 4.16 | 4.35 | 4.25 | 0.08 | 3 |
| 73 | 4.22 | 4.38 | 4.51 | 4.37 | 0.12 | 3 |
| 74 | 4.61 | 4.51 | 4.33 | 4.48 | 0.12 | 3 |
| 75 | 0.28 | 0.33 | 0.46 | 0.36 | 0.08 | 7 |
| 76 | 4.52 | 4.16 | 4.33 | 4.34 | 0.15 | 3 |
| 77 | 0.34 | 0.32 | 0.28 | 0.31 | 0.02 | 7 |
| 78 | 5.38 | 5.68 | 5.46 | 5.51 | 0.13 | 2 |
| 79 | 0.36 | 0.38 | 0.42 | 0.39 | 0.02 | 7 |
| 80 | 0.35 | 0.44 | 0.56 | 0.45 | 0.09 | 7 |
| 81 | 0.46 | 0.65 | 0.54 | 0.55 | 0.08 | 7 |
| 82 | 4.61 | 4.66 | 4.75 | 4.67 | 0.06 | 3 |
| 83 | 2.11 | 2.28 | 2.31 | 2.23 | 0.09 | 5 |
| 84 | 6.23 | 6.05 | 6.46 | 6.25 | 0.17 | 1 |
| 85 | 3.21 | 3.26 | 3.54 | 3.34 | 0.15 | 4 |
| 86 | 5.75 | 5.57 | 5.64 | 5.65 | 0.07 | 2 |
| 87 | 6.68 | 6.61 | 6.77 | 6.69 | 0.07 | 1 |
| 88 | 5.33 | 5.56 | 5.43 | 5.44 | 0.09 | 2 |
| 89 | 5.46 | 5.58 | 5.78 | 5.61 | 0.13 | 2 |
| 90 | 1.49 | 1.35 | 1.48 | 1.44 | 0.06 | 6 |
| 91 | 0.00 | 0.00 | 0.00 | 0.00 | 0.00 | 8 |
| 92 | 0.15 | 0.12 | 0.09 | 0.12 | 0.02 | 7 |
| 93 | 0.00 | 0.00 | 0.00 | 0.00 | 0.00 | 8 |
| 94 | 4.91 | 4.78 | 4.99 | 4.89 | 0.09 | 3 |
| 95 | 2.45 | 2.65 | 2.53 | 2.54 | 0.08 | 5 |
| 96 | 4.16 | 4.26 | 4.35 | 4.26 | 0.08 | 3 |
| 97 | 0.36 | 0.48 | 0.29 | 0.38 | 0.08 | 7 |
| 98 | 1.56 | 1.68 | 1.57 | 1.6 | 0.05 | 6 |
| 99 | 0.00 | 0.00 | 0.00 | 0.00 | 0.00 | 8 |
| 100 | 1.23 | 1.25 | 1.36 | 1.28 | 0.06 | 6 |
| 101 | 0.20 | 0.11 | 0.25 | 0.19 | 0.06 | 7 |
| 102 | 4.16 | 4.26 | 4.28 | 4.23 | 0.05 | 3 |
| 103 | 4.15 | 4.10 | 4.09 | 4.11 | 0.03 | 3 |
| 104 | 0.62 | 0.59 | 0.72 | 0.64 | 0.06 | 7 |
| 105 | 5.13 | 5.18 | 5.26 | 5.19 | 0.05 | 2 |
| 106 | 6.66 | 6.45 | 6.39 | 6.5 | 0.12 | 1 |
| 107 | 5.11 | 5.05 | 5.24 | 5.13 | 0.08 | 2 |
| 108 | 0.00 | 0.00 | 0.00 | 0.00 | 0.00 | 8 |
| 109 | 4.22 | 4.52 | 4.32 | 4.35 | 0.12 | 3 |
| 110 | 6.45 | 6.52 | 6.35 | 6.44 | 0.07 | 1 |
| 111 | 1.20 | 1.25 | 1.36 | 1.27 | 0.07 | 6 |
| 112 | 2.85 | 2.86 | 2.36 | 2.69 | 0.23 | 5 |
| 113 | 2.06 | 2.15 | 2.26 | 2.16 | 0.08 | 5 |
| 114 | 5.62 | 5.64 | 5.58 | 5.61 | 0.02 | 2 |
| 115 | 5.46 | 5.48 | 5.59 | 5.51 | 0.06 | 2 |
| 116 | 3.19 | 3.25 | 3.09 | 3.18 | 0.07 | 4 |
| 117 | 3.58 | 3.68 | 3.46 | 3.57 | 0.09 | 4 |
| 118 | 1.69 | 1.75 | 1.68 | 1.71 | 0.03 | 6 |
| 119 | 5.95 | 5.88 | 5.75 | 5.86 | 0.08 | 2 |
| 120 | 5.85 | 5.89 | 5.76 | 5.83 | 0.05 | 2 |
| 121 | 4.85 | 4.98 | 4.59 | 4.81 | 0.16 | 3 |
| 122 | 0.12 | 0.16 | 0.22 | 0.17 | 0.04 | 7 |
| 123 | 3.52 | 3.59 | 3.67 | 3.59 | 0.06 | 4 |
| 124 | 1.43 | 1.46 | 1.68 | 1.52 | 0.11 | 6 |
| 125 | 2.65 | 2.75 | 2.95 | 2.78 | 0.12 | 5 |
| 126 | 1.66 | 1.68 | 1.75 | 1.7 | 0.04 | 6 |
| 127 | 4.68 | 4.61 | 4.76 | 4.68 | 0.06 | 3 |
| 128 | 4.75 | 4.52 | 4.69 | 4.65 | 0.1 | 3 |
| 129 | 4.88 | 4.69 | 4.78 | 4.78 | 0.08 | 3 |
| 130 | 0.00 | 0.00 | 0.00 | 0.00 | 0.00 | 8 |
| 131 | 1.28 | 1.36 | 1.38 | 1.34 | 0.04 | 6 |
| 132 | 3.45 | 3.58 | 3.64 | 3.56 | 0.08 | 4 |
| 133 | 3.16 | 3.26 | 3.33 | 3.25 | 0.07 | 4 |
| 134 | 3.36 | 3.34 | 3.57 | 3.42 | 0.1 | 4 |
| 135 | 3.38 | 3.39 | 3.67 | 3.48 | 0.13 | 4 |
| 136 | 0.45 | 0.68 | 0.55 | 0.56 | 0.09 | 7 |
| 137 | 0.51 | 0.46 | 0.65 | 0.54 | 0.08 | 7 |
| 138 | 0.56 | 0.59 | 0.65 | 0.6 | 0.04 | 7 |
| 139 | 1.06 | 1.25 | 1.18 | 1.16 | 0.08 | 6 |
| 140 | 5.44 | / | / | 5.44 | 0.00 | 2 |
| 141 | 3.05 | 3.08 | 3.15 | 3.09 | 0.04 | 4 |
| 142 | 5.44 | 5.65 | 5.58 | 5.56 | 0.09 | 2 |
| 143 | 5.13 | 5.23 | 5.15 | 5.17 | 0.04 | 2 |
| 144 | 0.32 | 0.26 | 0.45 | 0.34 | 0.08 | 7 |
| 145 | 6.48 | 6.77 | 6.54 | 6.6 | 0.12 | 1 |
| 146 | 1.78 | 1.75 | 1.69 | 1.74 | 0.04 | 6 |
| 147 | 2.72 | 2.85 | 2.99 | 2.85 | 0.11 | 5 |
| 148 | 4.33 | 4.36 | 4.58 | 4.42 | 0.11 | 3 |
| 149 | 3.75 | 3.85 | 3.9 | 3.83 | 0.06 | 4 |
| 150 | 6.25 | 6.38 | 6.42 | 6.35 | 0.07 | 1 |
| 151 | 0.12 | 0.19 | 0.23 | 0.18 | 0.05 | 7 |
| 152 | 0.16 | 0.15 | 0.21 | 0.17 | 0.03 | 7 |
| 153 | 0.54 | 0.56 | 0.61 | 0.57 | 0.03 | 7 |
| 154 | 4.95 | 4.98 | 4.75 | 4.89 | 0.10 | 3 |
| 155 | 4.16 | 4.25 | 4.16 | 4.19 | 0.04 | 3 |
| 156 | 0.26 | 0.28 | 0.35 | 0.30 | 0.04 | 7 |
| 157 | 0.23 | 0.31 | 0.29 | 0.28 | 0.03 | 7 |
| 158 | 4.61 | 4.75 | 4.61 | 4.66 | 0.07 | 3 |
| 159 | 0.00 | 0.00 | 0.00 | 0.00 | 0.00 | 8 |
| 160 | 4.56 | 4.36 | 4.26 | 4.39 | 0.12 | 3 |
| 161 | 5.61 | 5.76 | 5.45 | 5.61 | 0.13 | 2 |
| 162 | 5.22 | 5.33 | 5.46 | 5.34 | 0.10 | 2 |
| 163 | 3.92 | 3.87 | 3.91 | 3.9 | 0.02 | 4 |
| 164 | 4.32 | 4.49 | 4.12 | 4.31 | 0.15 | 3 |
| 165 | 5.31 | 5.19 | 5.23 | 5.24 | 0.05 | 2 |
| 166 | 5.63 | 5.68 | 5.79 | 5.70 | 0.07 | 2 |
| 167 | 5.48 | 5.59 | 5.64 | 5.57 | 0.07 | 2 |
| 168 | 6.77 | 6.85 | 6.69 | 6.77 | 0.07 | 1 |
| 169 | 3.56 | 3.48 | 3.65 | 3.56 | 0.07 | 4 |
| 170 | 0.45 | 0.39 | 0.56 | 0.47 | 0.07 | 7 |
| 171 | 4.16 | 4.19 | 4.26 | 4.2 | 0.04 | 3 |
| 172 | 5.61 | 5.75 | 5.98 | 5.78 | 0.15 | 2 |
| 173 | 3.81 | 3.85 | 3.98 | 3.88 | 0.07 | 4 |
| 174 | 4.51 | 4.59 | 4.68 | 4.59 | 0.07 | 3 |
| 175 | 6.19 | 6.29 | 6.35 | 6.28 | 0.07 | 1 |
| 176 | 3.77 | 3.79 | 3.65 | 3.74 | 0.06 | 4 |
| 177 | 5.88 | 5.98 | 5.64 | 5.83 | 0.14 | 2 |
| 178 | 3.54 | 3.67 | 3.69 | 3.63 | 0.07 | 4 |
| 179 | 0.16 | 0.19 | 0.22 | 0.19 | 0.02 | 7 |
| 180 | 1.52 | 1.69 | 1.58 | 1.60 | 0.07 | 6 |
| 181 | 0.62 | 0.75 | 0.86 | 0.74 | 0.10 | 7 |
| 182 | 3.16 | 3.29 | 3.63 | 3.36 | 0.20 | 4 |
| 183 | 2.16 | 2.26 | 2.34 | 2.25 | 0.07 | 5 |
| 184 | 5.16 | 5.23 | 5.41 | 5.27 | 0.11 | 2 |
| 185 | 0.23 | 0.38 | 0.41 | 0.34 | 0.08 | 7 |
| 186 | 6.29 | 6.18 | 6.38 | 6.28 | 0.08 | 1 |
| 187 | 0.24 | 0.26 | 0.34 | 0.28 | 0.04 | 7 |
| 188 | 5.23 | 5.34 | 5.31 | 5.29 | 0.05 | 2 |
| 189 | 4.22 | 4.29 | 4.38 | 4.30 | 0.07 | 3 |
| 190 | 1.66 | 1.78 | 1.85 | 1.76 | 0.08 | 6 |
| 191 | 4.01 | 4.05 | 4.2 | 4.09 | 0.08 | 3 |
| 192 | 4.46 | 4.59 | 4.36 | 4.47 | 0.09 | 3 |
| 193 | 4.15 | 4.20 | 4.36 | 4.24 | 0.09 | 3 |
| 194 | 0.15 | 0.19 | 0.28 | 0.21 | 0.05 | 7 |
| 195 | 3.22 | 3.46 | 3.05 | 3.24 | 0.17 | 4 |
| 196 | 1.58 | 1.59 | 1.67 | 1.61 | 0.04 | 6 |
| 197 | 2.43 | 2.49 | 2.59 | 2.50 | 0.07 | 5 |
| 198 | 4.12 | 4.26 | 4.31 | 4.23 | 0.08 | 3 |
| 199 | 0.11 | 0.15 | 0.19 | 0.15 | 0.03 | 7 |
| 200 | 4.56 | 4.68 | 4.87 | 4.70 | 0.13 | 3 |
|  |  |  |  |  |  |  |
| Number | Test1 of root flesh  (A530-0.25*A657)*g-1Fw | Test2 of root flesh  (A530-0.25*A657)*g-1Fw | Test3 of root flesh  (A530-0.25*A657)*g-1Fw | Mean value | Standard deviation | Color grades✱ |
| 1 | 2.19 | 2.23 | 2.35 | 2.26 | 0.07 | 7 |
| 2 | 5.43 | 5.53 | 5.41 | 5.46 | 0.05 | 4 |
| 3 | 8.55 | 8.45 | 8.62 | 8.54 | 0.07 | 1 |
| 4 | 0.00 | 0.00 | 0.00 | 0.00 | 0.00 | 9 |
| 5 | 0.00 | 0.00 | 0.00 | 0.00 | 0.00 | 9 |
| 6 | 1.79 | 1.88 | 1.69 | 1.79 | 0.08 | 8 |
| 7 | 6.68 | 6.88 | 6.59 | 6.72 | 0.12 | 3 |
| 8 | 0.00 | 0.00 | 0.00 | 0.00 | 0.00 | 9 |
| 9 | 1.66 | 1.59 | 1.68 | 1.64 | 0.04 | 8 |
| 10 | 1.56 | 1.49 | 1.86 | 1.64 | 0.16 | 8 |
| 11 | 0.00 | 0.00 | 0.00 | 0.00 | 0.00 | 9 |
| 12 | 8.65 | 8.89 | 8.69 | 8.74 | 0.1 | 1 |
| 13 | 0.00 | 0.00 | 0.00 | 0.00 | 0.00 | 9 |
| 14 | 6.51 | 6.21 | 6.33 | 6.35 | 0.12 | 3 |
| 15 | 0.00 | 0.00 | 0.00 | 0.00 | 0.00 | 9 |
| 16 | 1.54 | 1.68 | 1.75 | 1.66 | 0.09 | 8 |
| 17 | 3.56 | 3.59 | 3.66 | 3.6 | 0.04 | 6 |
| 18 | 0.00 | 0.00 | 0.00 | 0.00 | 0.00 | 9 |
| 19 | 4.78 | 4.65 | 4.87 | 4.77 | 0.09 | 5 |
| 20 | 0.00 | 0.00 | 0.00 | 0.00 | 0.00 | 9 |
| 21 | 0.00 | 0.00 | 0.00 | 0.00 | 0.00 | 9 |
| 22 | 0.00 | 0.00 | 0.00 | 0.00 | 0.00 | 9 |
| 23 | 0.00 | 0.00 | 0.00 | 0.00 | 0.00 | 9 |
| 24 | 0.00 | 0.00 | 0.00 | 0.00 | 0.00 | 9 |
| 25 | 4.12 | 4.25 | 4.19 | 4.19 | 0.05 | 5 |
| 26 | 0.00 | 0.00 | 0.00 | 0.00 | 0.00 | 9 |
| 27 | 0.00 | 0.00 | 0.00 | 0.00 | 0.00 | 9 |
| 28 | 0.00 | 0.00 | 0.00 | 0.00 | 0.00 | 9 |
| 29 | 0.00 | 0.00 | 0.00 | 0.00 | 0.00 | 9 |
| 30 | 5.56 | 5.69 | 5.78 | 5.68 | 0.09 | 4 |
| 31 | 0.00 | 0.00 | 0.00 | 0.00 | 0.00 | 9 |
| 32 | 1.21 | 1.05 | 1.33 | 1.2 | 0.11 | 8 |
| 33 | 1.52 | 1.68 | 1.49 | 1.56 | 0.08 | 8 |
| 34 | 0.00 | 0.00 | 0.00 | 0.00 | 0.00 | 9 |
| 35 | 0.00 | 0.00 | 0.00 | 0.00 | 0.00 | 9 |
| 36 | 0.00 | 0.00 | 0.00 | 0.00 | 0.00 | 9 |
| 37 | 1.63 | 1.69 | 1.76 | 1.69 | 0.05 | 8 |
| 38 | 0.00 | 0.00 | 0.00 | 0.00 | 0.00 | 9 |
| 39 | 4.63 | 4.78 | 4.85 | 4.75 | 0.09 | 5 |
| 40 | 0.00 | 0.00 | 0.00 | 0.00 | 0.00 | 9 |
| 41 | 0.00 | 0.00 | 0.00 | 0.00 | 0.00 | 9 |
| 42 | 6.23 | 6.36 | 6.24 | 6.28 | 0.06 | 3 |
| 43 | 6.66 | 6.89 | 6.78 | 6.78 | 0.09 | 3 |
| 44 | 0.00 | 0.00 | 0.00 | 0.00 | 0.00 | 9 |
| 45 | 1.84 | 1.95 | 1.76 | 1.85 | 0.08 | 8 |
| 46 | 3.26 | 3.26 | 3.33 | 3.28 | 0.03 | 6 |
| 47 | 0.00 | 0.00 | 0.00 | 0.00 | 0.00 | 9 |
| 48 | 1.20 | 1.28 | 1.15 | 1.21 | 0.05 | 8 |
| 49 | 2.16 | 2.15 | 2.25 | 2.19 | 0.04 | 7 |
| 50 | 2.49 | 2.68 | 2.31 | 2.49 | 0.15 | 7 |
| 51 | 2.68 | 2.57 | 2.43 | 2.56 | 0.1 | 7 |
| 52 | 0.00 | 0.00 | 0.00 | 0.00 | 0.00 | 9 |
| 53 | 0.00 | 0.00 | 0.00 | 0.00 | 0.00 | 9 |
| 54 | 0.00 | 0.00 | 0.00 | 0.00 | 0.00 | 9 |
| 55 | 1.32 | 1.36 | 1.34 | 1.34 | 0.02 | 8 |
| 56 | 6.32 | 6.21 | 6.25 | 6.26 | 0.05 | 3 |
| 57 | 5.12 | 5.21 | 5.12 | 5.15 | 0.04 | 4 |
| 58 | 1.65 | 1.69 | 1.58 | 1.64 | 0.05 | 8 |
| 59 | 2.46 | 2.35 | 2.33 | 2.38 | 0.06 | 7 |
| 60 | 5.36 | 5.48 | 5.46 | 5.43 | 0.05 | 4 |
| 61 | 4.49 | 4.52 | 4.61 | 4.54 | 0.05 | 5 |
| 62 | 0.00 | 0.00 | 0.00 | 0.00 | 0.00 | 9 |
| 63 | 0.00 | 0.00 | 0.00 | 0.00 | 0.00 | 9 |
| 64 | 1.42 | 1.38 | 1.59 | 1.46 | 0.09 | 8 |
| 65 | 7.68 | 7.76 | 7.95 | 7.8 | 0.11 | 2 |
| 66 | 1.66 | 1.69 | 1.76 | 1.7 | 0.04 | 8 |
| 67 | 1.15 | 1.19 | 1.26 | 1.2 | 0.05 | 8 |
| 68 | 0.00 | 0.00 | 0.00 | 0.00 | 0.00 | 9 |
| 69 | 1.45 | 1.49 | 1.58 | 1.51 | 0.05 | 8 |
| 70 | 1.44 | 1.68 | 1.30 | 1.47 | 0.16 | 8 |
| 71 | 0.00 | 0.00 | 0.00 | 0.00 | 0.00 | 9 |
| 72 | 2.53 | 2.46 | 2.49 | 2.49 | 0.03 | 7 |
| 73 | 0.00 | 0.00 | 0.00 | 0.00 | 0.00 | 9 |
| 74 | 0.00 | 0.00 | 0.00 | 0.00 | 0.00 | 9 |
| 75 | 0.00 | 0.00 | 0.00 | 0.00 | 0.00 | 9 |
| 76 | 1.56 | 1.78 | 1.42 | 1.59 | 0.15 | 8 |
| 77 | 0.00 | 0.00 | 0.00 | 0.00 | 0.00 | 9 |
| 78 | 1.73 | 1.69 | 1.86 | 1.76 | 0.07 | 8 |
| 79 | 0.00 | 0.00 | 0.00 | 0.00 | 0.00 | 9 |
| 80 | 0.00 | 0.00 | 0.00 | 0.00 | 0.00 | 9 |
| 81 | 0.00 | 0.00 | 0.00 | 0.00 | 0.00 | 9 |
| 82 | 0.00 | 0.00 | 0.00 | 0.00 | 0.00 | 9 |
| 83 | 0.00 | 0.00 | 0.00 | 0.00 | 0.00 | 9 |
| 84 | 0.00 | 0.00 | 0.00 | 0.00 | 0.00 | 9 |
| 85 | 0.00 | 0.00 | 0.00 | 0.00 | 0.00 | 9 |
| 86 | 1.85 | 1.93 | 1.58 | 1.79 | 0.15 | 8 |
| 87 | 6.15 | 6.35 | 6.21 | 6.24 | 0.08 | 3 |
| 88 | 2.66 | 2.69 | 2.79 | 2.71 | 0.06 | 7 |
| 89 | 4.06 | 4.02 | 4.15 | 4.08 | 0.05 | 5 |
| 90 | 0.00 | 0.00 | 0.00 | 0.00 | 0.00 | 9 |
| 91 | 0.00 | 0.00 | 0.00 | 0.00 | 0.00 | 9 |
| 92 | 0.00 | 0.00 | 0.00 | 0.00 | 0.00 | 9 |
| 93 | 0.00 | 0.00 | 0.00 | 0.00 | 0.00 | 9 |
| 94 | 2.87 | 2.84 | 2.96 | 2.89 | 0.05 | 7 |
| 95 | 0.00 | 0.00 | 0.00 | 0.00 | 0.00 | 9 |
| 96 | 0.00 | 0.00 | 0.00 | 0.00 | 0.00 | 9 |
| 97 | 0.00 | 0.00 | 0.00 | 0.00 | 0.00 | 9 |
| 98 | 0.00 | 0.00 | 0.00 | 0.00 | 0.00 | 9 |
| 99 | 0.00 | 0.00 | 0.00 | 0.00 | 0.00 | 9 |
| 100 | 0.00 | 0.00 | 0.00 | 0.00 | 0.00 | 9 |
| 101 | 0.00 | 0.00 | 0.00 | 0.00 | 0.00 | 9 |
| 102 | 1.62 | 1.60 | 1.52 | 1.58 | 0.04 | 8 |
| 103 | 2.16 | 2.26 | 2.35 | 2.26 | 0.08 | 7 |
| 104 | 0.00 | 0.00 | 0.00 | 0.00 | 0.00 | 9 |
| 105 | 1.46 | 1.38 | 1.50 | 1.45 | 0.05 | 8 |
| 106 | 8.43 | 8.46 | 8.58 | 8.49 | 0.06 | 1 |
| 107 | 2.46 | 2.49 | 2.68 | 2.54 | 0.1 | 7 |
| 108 | 0.00 | 0.00 | 0.00 | 0.00 | 0.00 | 9 |
| 109 | 1.78 | 1.69 | 1.60 | 1.69 | 0.07 | 8 |
| 110 | 8.21 | 8.19 | 8.3 | 8.23 | 0.05 | 1 |
| 111 | 0.00 | 0.00 | 0.00 | 0.00 | 0.00 | 9 |
| 112 | 0.00 | 0.00 | 0.00 | 0.00 | 0.00 | 9 |
| 113 | 0.00 | 0.00 | 0.00 | 0.00 | 0.00 | 9 |
| 114 | 3.98 | 3.87 | 3.69 | 3.85 | 0.12 | 6 |
| 115 | 3.2 | 3.16 | 3.36 | 3.24 | 0.09 | 6 |
| 116 | 0.00 | 0.00 | 0.00 | 0.00 | 0.00 | 9 |
| 117 | 0.00 | 0.00 | 0.00 | 0.00 | 0.00 | 9 |
| 118 | 0.00 | 0.00 | 0.00 | 0.00 | 0.00 | 9 |
| 119 | 2.96 | 2.87 | 2.99 | 2.94 | 0.05 | 7 |
| 120 | 0.00 | 0.00 | 0.00 | 0.00 | 0.00 | 9 |
| 121 | 3.36 | 3.56 | 3.15 | 3.36 | 0.17 | 6 |
| 122 | 0.00 | 0.00 | 0.00 | 0.00 | 0.00 | 9 |
| 123 | 0.00 | 0.00 | 0.00 | 0.00 | 0.00 | 9 |
| 124 | 0.00 | 0.00 | 0.00 | 0.00 | 0.00 | 9 |
| 125 | 0.00 | 0.00 | 0.00 | 0.00 | 0.00 | 9 |
| 126 | 0.00 | 0.00 | 0.00 | 0.00 | 0.00 | 9 |
| 127 | 0.00 | 0.00 | 0.00 | 0.00 | 0.00 | 9 |
| 128 | 1.58 | 1.56 | 1.82 | 1.65 | 0.12 | 8 |
| 129 | 1.30 | 1.20 | 1.28 | 1.26 | 0.04 | 8 |
| 130 | 0.00 | 0.00 | 0.00 | 0.00 | 0.00 | 9 |
| 131 | 0.00 | 0.00 | 0.00 | 0.00 | 0.00 | 9 |
| 132 | 0.00 | 0.00 | 0.00 | 0.00 | 0.00 | 9 |
| 133 | 0.00 | 0.00 | 0.00 | 0.00 | 0.00 | 9 |
| 134 | 0.00 | 0.00 | 0.00 | 0.00 | 0.00 | 9 |
| 135 | 0.00 | 0.00 | 0.00 | 0.00 | 0.00 | 9 |
| 136 | 0.42 | 0.31 | 0.28 | 0.34 | 0.06 | 9 |
| 137 | 0.26 | 0.15 | 0.2 | 0.2 | 0.04 | 9 |
| 138 | 0.35 | 0.31 | 0.25 | 0.3 | 0.04 | 9 |
| 139 | 0.00 | 0.00 | 0.00 | 0.00 | 0.00 | 9 |
| 140 | 1.84 | 1.92 | 1.66 | 1.81 | 0.11 | 8 |
| 141 | 0.00 | 0.00 | 0.00 | 0.00 | 0.00 | 9 |
| 142 | 2.53 | 2.51 | 2.13 | 2.39 | 0.18 | 7 |
| 143 | 3.14 | 3.25 | 3.08 | 3.16 | 0.07 | 6 |
| 144 | 0.42 | 0.44 | 0.29 | 0.38 | 0.07 | 9 |
| 145 | 4.36 | 4.26 | 4.38 | 4.33 | 0.05 | 5 |
| 146 | 0.00 | 0.00 | 0.00 | 0.00 | 0.00 | 9 |
| 147 | 0.00 | 0.00 | 0.00 | 0.00 | 0.00 | 9 |
| 148 | 5.41 | 5.59 | 5.71 | 5.57 | 0.12 | 4 |
| 149 | 0.00 | 0.00 | 0.00 | 0.00 | 0.00 | 9 |
| 150 | 0.00 | 0.00 | 0.00 | 0.00 | 0.00 | 9 |
| 151 | 0.44 | 0.35 | 0.31 | 0.37 | 0.05 | 9 |
| 152 | 0.00 | 0.00 | 0.00 | 0.00 | 0.00 | 9 |
| 153 | 0.00 | 0.00 | 0.00 | 0.00 | 0.00 | 9 |
| 154 | 3.25 | 3.25 | 3.64 | 3.38 | 0.18 | 6 |
| 155 | 0.00 | 0.00 | 0.00 | 0.00 | 0.00 | 9 |
| 156 | 0.00 | 0.00 | 0.00 | 0.00 | 0.00 | 9 |
| 157 | 0.00 | 0.00 | 0.00 | 0.00 | 0.00 | 9 |
| 158 | 2.58 | 2.13 | 2.46 | 2.39 | 0.19 | 7 |
| 159 | 0.00 | 0.00 | 0.00 | 0.00 | 0.00 | 9 |
| 160 | 1.62 | 1.52 | 1.41 | 1.52 | 0.09 | 8 |
| 161 | 0.00 | 0.00 | 0.00 | 0.00 | 0.00 | 9 |
| 162 | 6.42 | 6.41 | 6.55 | 6.46 | 0.06 | 3 |
| 163 | 2.44 | 2.65 | 2.38 | 2.49 | 0.12 | 7 |
| 164 | 0.00 | 0.00 | 0.00 | 0.00 | 0.00 | 9 |
| 165 | 2.60 | 2.48 | 2.73 | 2.6 | 0.1 | 7 |
| 166 | 7.74 | 7.43 | 7.69 | 7.62 | 0.14 | 2 |
| 167 | 5.06 | 5.09 | 5.15 | 5.1 | 0.04 | 4 |
| 168 | 7.21 | 7.39 | 7.52 | 7.37 | 0.13 | 2 |
| 169 | 0.00 | 0.00 | 0.00 | 0.00 | 0.00 | 9 |
| 170 | 0.00 | 0.00 | 0.00 | 0.00 | 0.00 | 9 |
| 171 | 1.65 | 1.40 | 1.86 | 1.64 | 0.19 | 8 |
| 172 | 1.84 | 1.72 | 1.69 | 1.75 | 0.06 | 8 |
| 173 | 0.00 | 0.00 | 0.00 | 0.00 | 0.00 | 9 |
| 174 | 0.00 | 0.00 | 0.00 | 0.00 | 0.00 | 9 |
| 175 | 3.62 | 3.87 | 3.58 | 3.69 | 0.13 | 6 |
| 176 | 0.00 | 0.00 | 0.00 | 0.00 | 0.00 | 9 |
| 177 | 3.06 | 3.15 | 3.26 | 3.16 | 0.08 | 6 |
| 178 | 0.00 | 0.00 | 0.00 | 0.00 | 0.00 | 9 |
| 179 | 0.18 | 0.19 | 0.07 | 0.15 | 0.05 | 9 |
| 180 | 0.00 | 0.00 | 0.00 | 0.00 | 0.00 | 9 |
| 181 | 0.00 | 0.00 | 0.00 | 0.00 | 0.00 | 9 |
| 182 | 0.00 | 0.00 | 0.00 | 0.00 | 0.00 | 9 |
| 183 | 0.00 | 0.00 | 0.00 | 0.00 | 0.00 | 9 |
| 184 | 1.52 | 1.48 | 1.68 | 1.56 | 0.09 | 8 |
| 185 | 0.00 | 0.00 | 0.00 | 0.00 | 0.00 | 9 |
| 186 | 5.98 | 5.78 | 5.84 | 5.87 | 0.08 | 4 |
| 187 | 0.00 | 0.00 | 0.00 | 0.00 | 0.00 | 9 |
| 188 | 0.00 | 0.00 | 0.00 | 0.00 | 0.00 | 9 |
| 189 | 3.42 | 3.58 | 3.68 | 3.56 | 0.11 | 6 |
| 190 | 0.28 | 0.21 | 0.14 | 0.21 | 0.06 | 9 |
| 191 | 0.00 | 0.00 | 0.00 | 0.00 | 0.00 | 9 |
| 192 | 0.00 | 0.00 | 0.00 | 0.00 | 0.00 | 9 |
| 193 | 1.78 | 1.62 | 1.92 | 1.77 | 0.12 | 8 |
| 194 | 0.00 | 0.00 | 0.00 | 0.00 | 0.00 | 9 |
| 195 | 0.00 | 0.00 | 0.00 | 0.00 | 0.00 | 9 |
| 196 | 2.73 | 2.59 | 2.61 | 2.64 | 0.06 | 7 |
| 197 | 0.00 | 0.00 | 0.00 | 0.00 | 0.00 | 9 |
| 198 | 1.14 | 1.16 | 1.28 | 1.19 | 0.06 | 8 |
| 199 | 0.00 | 0.00 | 0.00 | 0.00 | 0.00 | 9 |
| 200 | 1.45 | 1.68 | 1.50 | 1.54 | 0.1 | 8 |
| #When 6≤Q, 5≤Q<6, 4≤Q<5, 3≤Q<4, 3≤Q<2, 2≤Q<1, 1≤Q<0, Q=0, the anthocyanin content of root skin were scored as first, second, third, forth, fifth, sixth, seventh and eighth grades, respectively.  ✱ When 8≤Q, 7≤Q<8, 6≤Q<7, 5≤Q<6, 4≤Q<5, 3≤Q<4, 2≤Q<3, 1≤Q<2, Q=0, the anthocyanin content of root flesh were scored as first, second, third, forth, fifth, sixth, seventh, eighth and ninth grades, respectively. | | | | | | |

Table S2. QTL analysis of QAC1

| Number | Group | Position | Locus | LOD | Additive | Dominance | expl% |
| --- | --- | --- | --- | --- | --- | --- | --- |
| 1 | 1 | 0 | Marker809 | 0.437715224 | -0.055569622 | -0.017826892 | 0.357265715 |
| 2 | 1 | 0 | Marker803 | 0.437715224 | -0.055569622 | -0.017826892 | 0.357265715 |
| 3 | 1 | 0 | Marker808 | 0.437715224 | -0.055569622 | -0.017826892 | 0.357265715 |
| 4 | 1 | 0 | Marker810 | 0.437715224 | -0.055569622 | -0.017826892 | 0.357265715 |
| 5 | 1 | 0 | Marker806 | 0.437715224 | -0.055569622 | -0.017826892 | 0.357265715 |
| 6 | 1 | 0 | Marker802 | 0.437715224 | -0.055569622 | -0.017826892 | 0.357265715 |
| 7 | 1 | 0 | Marker807 | 0.437715224 | -0.055569622 | -0.017826892 | 0.357265715 |
| 8 | 1 | 3.154 | Marker362 | 0.298042203 | -0.083333333 | -0.032828283 | 0.822305979 |
| 9 | 1 | 3.904 | Marker622 | 0.275712731 | -0.083333333 | -0.033333333 | 0.824033953 |
| 10 | 1 | 4.404 | Marker642 | 0.238340192 | -0.079172962 | -0.041645838 | 0.781393055 |
| 11 | 1 | 4.905 | Marker638 | 0.172984313 | -0.062056738 | -0.075198164 | 0.7116339 |
| 12 | 1 | 4.905 | Marker635 | 0.172984313 | -0.062056738 | -0.075198164 | 0.7116339 |
| 13 | 1 | 4.905 | Marker637 | 0.172984313 | -0.062056738 | -0.075198164 | 0.7116339 |
| 14 | 1 | 5.405 | Marker882 | 0.219811636 | -0.052728955 | -0.057745677 | 0.476228591 |
| 15 | 1 | 5.655 | Marker1245 | 0.27816275 | -0.063829787 | -0.036562694 | 0.518583493 |
| 16 | 1 | 5.655 | Marker1456 | 0.27816275 | -0.063829787 | -0.036562694 | 0.518583493 |
| 17 | 1 | 5.655 | Marker1505 | 0.27816275 | -0.063829787 | -0.036562694 | 0.518583493 |
| 18 | 1 | 5.655 | Marker1508 | 0.27816275 | -0.063829787 | -0.036562694 | 0.518583493 |
| 19 | 1 | 5.655 | Marker860 | 0.27816275 | -0.063829787 | -0.036562694 | 0.518583493 |
| 20 | 1 | 5.655 | Marker1506 | 0.27816275 | -0.063829787 | -0.036562694 | 0.518583493 |
| 21 | 1 | 5.655 | Marker1458 | 0.27816275 | -0.063829787 | -0.036562694 | 0.518583493 |
| 22 | 1 | 5.655 | Marker1457 | 0.27816275 | -0.063829787 | -0.036562694 | 0.518583493 |
| 23 | 1 | 5.655 | Marker1507 | 0.27816275 | -0.063829787 | -0.036562694 | 0.518583493 |
| 24 | 1 | 5.655 | Marker1248 | 0.27816275 | -0.063829787 | -0.036562694 | 0.518583493 |
| 25 | 1 | 6.405 | Marker1898 | 0.22135836 | -0.06079932 | -0.072465986 | 0.674226995 |
| 26 | 1 | 6.405 | Marker1907 | 0.22135836 | -0.06079932 | -0.072465986 | 0.674226995 |
| 27 | 1 | 6.405 | Marker1702 | 0.22135836 | -0.06079932 | -0.072465986 | 0.674226995 |
| 28 | 1 | 6.405 | Marker1539 | 0.22135836 | -0.06079932 | -0.072465986 | 0.674226995 |
| 29 | 1 | 6.405 | Marker1906 | 0.22135836 | -0.06079932 | -0.072465986 | 0.674226995 |
| 30 | 1 | 6.655 | Marker2327 | 0.367951591 | -0.070994355 | -0.052666991 | 0.698431119 |
| 31 | 1 | 6.905 | Marker2331 | 0.342867327 | -0.070763088 | -0.053286532 | 0.698125305 |
| 32 | 1 | 6.905 | Marker2334 | 0.342867327 | -0.070763088 | -0.053286532 | 0.698125305 |
| 33 | 1 | 7.655 | Marker2871 | 0.291952445 | -0.072916667 | -0.094781353 | 1.041479725 |
| 34 | 1 | 7.655 | Marker2879 | 0.291952445 | -0.072916667 | -0.094781353 | 1.041479725 |
| 35 | 1 | 7.655 | Marker2872 | 0.291952445 | -0.072916667 | -0.094781353 | 1.041479725 |
| 36 | 1 | 7.655 | Marker2880 | 0.291952445 | -0.072916667 | -0.094781353 | 1.041479725 |
| 37 | 1 | 7.655 | Marker2878 | 0.291952445 | -0.072916667 | -0.094781353 | 1.041479725 |
| 38 | 1 | 7.905 | Marker2660 | 0.066764286 | -0.061835106 | -0.115860972 | 1.09703213 |
| 39 | 1 | 8.655 | Marker2513 | 0.094462873 | -0.059410431 | -0.074728662 | 0.672374901 |
| 40 | 1 | 8.655 | Marker2608 | 0.094462873 | -0.059410431 | -0.074728662 | 0.672374901 |
| 41 | 1 | 8.655 | Marker3595 | 0.094462873 | -0.059410431 | -0.074728662 | 0.672374901 |
| 42 | 1 | 8.655 | Marker2610 | 0.094462873 | -0.059410431 | -0.074728662 | 0.672374901 |
| 43 | 1 | 8.655 | Marker2609 | 0.094462873 | -0.059410431 | -0.074728662 | 0.672374901 |
| 44 | 1 | 9.155 | Marker2769 | 0.38566095 | -0.081632653 | -0.032127703 | 0.78898314 |
| 45 | 1 | 9.155 | Marker2768 | 0.38566095 | -0.081632653 | -0.032127703 | 0.78898314 |
| 46 | 1 | 9.405 | Marker2520 | 0.246778206 | -0.081632653 | -0.032613045 | 0.790608103 |
| 47 | 1 | 9.405 | Marker2563 | 0.246778206 | -0.081632653 | -0.032613045 | 0.790608103 |
| 48 | 1 | 9.405 | Marker2519 | 0.246778206 | -0.081632653 | -0.032613045 | 0.790608103 |
| 49 | 1 | 9.405 | Marker2517 | 0.246778206 | -0.081632653 | -0.032613045 | 0.790608103 |
| 50 | 1 | 9.405 | Marker2518 | 0.246778206 | -0.081632653 | -0.032613045 | 0.790608103 |
| 51 | 1 | 9.655 | Marker2585 | 0.212963338 | -0.070521542 | -0.053908813 | 0.69772308 |
| 52 | 1 | 9.655 | Marker2586 | 0.212963338 | -0.070521542 | -0.053908813 | 0.69772308 |
| 53 | 1 | 10.155 | Marker3445 | 0.115197157 | -0.059410431 | -0.074728662 | 0.672374901 |
| 54 | 1 | 10.405 | Marker3877 | 0.117759118 | -0.05890538 | -0.075513772 | 0.67164393 |
| 55 | 1 | 10.655 | Marker3878 | 0.210665038 | -0.058376839 | -0.076317038 | 0.670862297 |
| 56 | 1 | 10.655 | Marker3881 | 0.210665038 | -0.058376839 | -0.076317038 | 0.670862297 |
| 57 | 1 | 11.655 | Marker3665 | 0.39912744 | -0.068888889 | -0.051895425 | 0.661740766 |
| 58 | 1 | 11.655 | Marker3661 | 0.39912744 | -0.068888889 | -0.051895425 | 0.661740766 |
| 59 | 1 | 11.655 | Marker3662 | 0.39912744 | -0.068888889 | -0.051895425 | 0.661740766 |
| 60 | 1 | 11.655 | Marker3663 | 0.39912744 | -0.068888889 | -0.051895425 | 0.661740766 |
| 61 | 1 | 12.955 | Marker4612 | 0.609779335 | -0.049206349 | -0.054815842 | 0.419791467 |
| 62 | 1 | 13.205 | Marker5325 | 0.639840047 | -0.049689441 | -0.053952016 | 0.420375662 |
| 63 | 1 | 13.705 | Marker5141 | 0.711822732 | -0.039485359 | -0.033944012 | 0.23091723 |
| 64 | 1 | 13.705 | Marker5140 | 0.711822732 | -0.039485359 | -0.033944012 | 0.23091723 |
| 65 | 1 | 13.705 | Marker5139 | 0.711822732 | -0.039485359 | -0.033944012 | 0.23091723 |
| 66 | 1 | 14.205 | Marker6601 | 0.673889083 | -0.041541003 | -0.036749408 | 0.259382557 |
| 67 | 1 | 14.205 | Marker6600 | 0.673889083 | -0.041541003 | -0.036749408 | 0.259382557 |
| 68 | 1 | 14.455 | Marker4587 | 0.329150701 | -0.052140954 | -0.063781688 | 0.506200488 |
| 69 | 1 | 14.705 | Marker5382 | 0.16944343 | -0.041203236 | -0.0831929 | 0.535014324 |
| 70 | 1 | 14.705 | Marker4982 | 0.16944343 | -0.041203236 | -0.0831929 | 0.535014324 |
| 71 | 1 | 14.705 | Marker4750 | 0.16944343 | -0.041203236 | -0.0831929 | 0.535014324 |
| 72 | 1 | 14.955 | Marker4545 | 0.044315386 | -0.028467909 | -0.10535738 | 0.643088608 |
| 73 | 1 | 14.955 | Marker4977 | 0.044315386 | -0.028467909 | -0.10535738 | 0.643088608 |
| 74 | 1 | 14.955 | Marker4006 | 0.044315386 | -0.028467909 | -0.10535738 | 0.643088608 |
| 75 | 1 | 14.955 | Marker4453 | 0.044315386 | -0.028467909 | -0.10535738 | 0.643088608 |
| 76 | 1 | 14.955 | Marker4546 | 0.044315386 | -0.028467909 | -0.10535738 | 0.643088608 |
| 77 | 1 | 14.955 | Marker4008 | 0.044315386 | -0.028467909 | -0.10535738 | 0.643088608 |
| 78 | 1 | 14.955 | Marker4004 | 0.044315386 | -0.028467909 | -0.10535738 | 0.643088608 |
| 79 | 1 | 14.955 | Marker4007 | 0.044315386 | -0.028467909 | -0.10535738 | 0.643088608 |
| 80 | 1 | 14.955 | Marker4547 | 0.044315386 | -0.028467909 | -0.10535738 | 0.643088608 |
| 81 | 1 | 14.955 | Marker4005 | 0.044315386 | -0.028467909 | -0.10535738 | 0.643088608 |
| 82 | 1 | 15.205 | Marker5619 | 0.041262924 | -0.026849037 | -0.103568614 | 0.614371237 |
| 83 | 1 | 15.455 | Marker5630 | 0.16499617 | -0.039584364 | -0.081314423 | 0.504974252 |
| 84 | 1 | 15.455 | Marker5631 | 0.16499617 | -0.039584364 | -0.081314423 | 0.504974252 |
| 85 | 1 | 15.455 | Marker5460 | 0.16499617 | -0.039584364 | -0.081314423 | 0.504974252 |
| 86 | 1 | 16.205 | Marker6364 | 0.231866327 | -0.063104449 | -0.03866191 | 0.516449568 |
| 87 | 1 | 16.455 | Marker6937 | 0.234638454 | -0.06472332 | -0.040721621 | 0.547654504 |
| 88 | 1 | 16.705 | Marker7103 | 0.231866327 | -0.063104449 | -0.03866191 | 0.516449568 |
| 89 | 1 | 16.705 | Marker7092 | 0.231866327 | -0.063104449 | -0.03866191 | 0.516449568 |
| 90 | 1 | 17.205 | Marker6881 | 0.367864221 | -0.052466151 | -0.01858965 | 0.321720717 |
| 91 | 1 | 17.205 | Marker6882 | 0.367864221 | -0.052466151 | -0.01858965 | 0.321720717 |
| 92 | 1 | 17.705 | Marker7485 | 0.330962181 | -0.060064935 | -0.034715285 | 0.460302522 |
| 93 | 1 | 17.705 | Marker7486 | 0.330962181 | -0.060064935 | -0.034715285 | 0.460302522 |
| 94 | 1 | 17.955 | Marker7668 | 0.293859398 | -0.048172757 | -0.056589147 | 0.418530944 |
| 95 | 1 | 17.955 | Marker7669 | 0.293859398 | -0.048172757 | -0.056589147 | 0.418530944 |
| 96 | 1 | 18.205 | Marker7556 | 0.179469183 | -0.060077519 | -0.07828726 | 0.708530835 |
| 97 | 1 | 18.205 | Marker7557 | 0.179469183 | -0.060077519 | -0.07828726 | 0.708530835 |
| 98 | 1 | 18.205 | Marker7561 | 0.179469183 | -0.060077519 | -0.07828726 | 0.708530835 |
| 99 | 1 | 18.205 | Marker7560 | 0.179469183 | -0.060077519 | -0.07828726 | 0.708530835 |
| 100 | 1 | 18.205 | Marker8102 | 0.179469183 | -0.060077519 | -0.07828726 | 0.708530835 |
| 101 | 1 | 18.205 | Marker8101 | 0.179469183 | -0.060077519 | -0.07828726 | 0.708530835 |
| 102 | 1 | 18.205 | Marker7804 | 0.179469183 | -0.060077519 | -0.07828726 | 0.708530835 |
| 103 | 1 | 18.205 | Marker8105 | 0.179469183 | -0.060077519 | -0.07828726 | 0.708530835 |
| 104 | 1 | 18.205 | Marker7630 | 0.179469183 | -0.060077519 | -0.07828726 | 0.708530835 |
| 105 | 1 | 20.758 | Marker8971 | 0.321193387 | -0.092592593 | -0.062592593 | 1.14745246 |
| 106 | 1 | 21.008 | Marker8966 | 0.32120458 | -0.094339623 | -0.064636652 | 1.196785734 |
| 107 | 1 | 21.258 | Marker8675 | 0.341312164 | -0.094339623 | -0.064339623 | 1.194841657 |
| 108 | 1 | 21.258 | Marker8674 | 0.341312164 | -0.094339623 | -0.064339623 | 1.194841657 |
| 109 | 1 | 21.258 | Marker8680 | 0.341312164 | -0.094339623 | -0.064339623 | 1.194841657 |
| 110 | 1 | 21.258 | Marker8679 | 0.341312164 | -0.094339623 | -0.064339623 | 1.194841657 |
| 111 | 1 | 22.008 | Marker8474 | 0.217813181 | -0.067597125 | -0.052206919 | 0.643860762 |
| 112 | 1 | 22.008 | Marker8468 | 0.217813181 | -0.067597125 | -0.052206919 | 0.643860762 |
| 113 | 1 | 24.309 | Marker8654 | 0.106438837 | -0.080357143 | -0.118630396 | 1.423220291 |
| 114 | 1 | 24.309 | Marker9100 | 0.106438837 | -0.080357143 | -0.118630396 | 1.423220291 |
| 115 | 1 | 24.309 | Marker9101 | 0.106438837 | -0.080357143 | -0.118630396 | 1.423220291 |
| 116 | 1 | 24.309 | Marker9099 | 0.106438837 | -0.080357143 | -0.118630396 | 1.423220291 |
| 117 | 1 | 24.309 | Marker8652 | 0.106438837 | -0.080357143 | -0.118630396 | 1.423220291 |
| 118 | 1 | 24.309 | Marker8653 | 0.106438837 | -0.080357143 | -0.118630396 | 1.423220291 |
| 119 | 1 | 25.059 | Marker9043 | 0.02702452 | -0.058244681 | -0.124153772 | 1.148060284 |
| 120 | 1 | 25.559 | Marker9198 | 0.017476914 | -0.049045599 | -0.146606575 | 1.339760965 |
| 121 | 1 | 26.059 | Marker9375 | 0.004252856 | -0.05 | -0.15 | 1.400457426 |
| 122 | 1 | 26.059 | Marker9372 | 0.004252856 | -0.05 | -0.15 | 1.400457426 |
| 123 | 1 | 26.059 | Marker9620 | 0.004252856 | -0.05 | -0.15 | 1.400457426 |
| 124 | 1 | 26.059 | Marker9786 | 0.004252856 | -0.05 | -0.15 | 1.400457426 |
| 125 | 1 | 26.309 | Marker9661 | 0.072411812 | -0.063414634 | -0.127576357 | 1.26140205 |
| 126 | 1 | 26.309 | Marker9662 | 0.072411812 | -0.063414634 | -0.127576357 | 1.26140205 |
| 127 | 1 | 26.309 | Marker9337 | 0.072411812 | -0.063414634 | -0.127576357 | 1.26140205 |
| 128 | 1 | 26.309 | Marker9658 | 0.072411812 | -0.063414634 | -0.127576357 | 1.26140205 |
| 129 | 1 | 26.559 | Marker9660 | 0.061208392 | -0.077050998 | -0.150221729 | 1.789338417 |
| 130 | 1 | 26.809 | Marker9775 | 0.059386288 | -0.085636856 | -0.167816597 | 2.224616439 |
| 131 | 1 | 27.309 | Marker9551 | 0.064309033 | -0.063602376 | -0.130746207 | 1.304895698 |
| 132 | 1 | 28.109 | Marker10882 | 0.166715998 | -0.042079274 | -0.125914708 | 0.98784622 |
| 133 | 1 | 28.359 | Marker10831 | 0.123000611 | -0.055761099 | -0.148784355 | 1.451006528 |
| 134 | 1 | 28.359 | Marker10965 | 0.123000611 | -0.055761099 | -0.148784355 | 1.451006528 |
| 135 | 1 | 28.359 | Marker10898 | 0.123000611 | -0.055761099 | -0.148784355 | 1.451006528 |
| 136 | 1 | 28.359 | Marker10964 | 0.123000611 | -0.055761099 | -0.148784355 | 1.451006528 |
| 137 | 1 | 29.91 | Marker11648 | 0.502147791 | -0.019047619 | -0.087012987 | 0.417029512 |
| 138 | 1 | 29.91 | Marker11460 | 0.502147791 | -0.019047619 | -0.087012987 | 0.417029512 |
| 139 | 1 | 29.91 | Marker11495 | 0.502147791 | -0.019047619 | -0.087012987 | 0.417029512 |
| 140 | 1 | 29.91 | Marker11649 | 0.502147791 | -0.019047619 | -0.087012987 | 0.417029512 |
| 141 | 1 | 30.16 | Marker11907 | 0.561410064 | -0.006728778 | -0.065269626 | 0.216268199 |
| 142 | 1 | 30.16 | Marker11906 | 0.561410064 | -0.006728778 | -0.065269626 | 0.216268199 |
| 143 | 1 | 30.16 | Marker11909 | 0.561410064 | -0.006728778 | -0.065269626 | 0.216268199 |
| 144 | 1 | 30.16 | Marker11991 | 0.561410064 | -0.006728778 | -0.065269626 | 0.216268199 |
| 145 | 1 | 30.16 | Marker11908 | 0.561410064 | -0.006728778 | -0.065269626 | 0.216268199 |
| 146 | 1 | 30.16 | Marker11910 | 0.561410064 | -0.006728778 | -0.065269626 | 0.216268199 |
| 147 | 1 | 30.66 | Marker12452 | 0.600284253 | 0.01369509 | -0.065886732 | 0.233466343 |
| 148 | 1 | 30.66 | Marker12451 | 0.600284253 | 0.01369509 | -0.065886732 | 0.233466343 |
| 149 | 1 | 30.91 | Marker12663 | 0.656440869 | 0.025530839 | -0.044451934 | 0.167267695 |
| 150 | 1 | 30.91 | Marker12664 | 0.656440869 | 0.025530839 | -0.044451934 | 0.167267695 |
| 151 | 1 | 30.91 | Marker12671 | 0.656440869 | 0.025530839 | -0.044451934 | 0.167267695 |
| 152 | 1 | 30.91 | Marker12662 | 0.656440869 | 0.025530839 | -0.044451934 | 0.167267695 |
| 153 | 1 | 31.16 | Marker12947 | 0.641219015 | 0.026224641 | -0.043325457 | 0.166330654 |
| 154 | 1 | 31.66 | Marker13588 | 0.518813291 | 0.017323208 | -0.060860623 | 0.214095098 |
| 155 | 1 | 31.66 | Marker13814 | 0.518813291 | 0.017323208 | -0.060860623 | 0.214095098 |
| 156 | 1 | 31.66 | Marker13589 | 0.518813291 | 0.017323208 | -0.060860623 | 0.214095098 |
| 157 | 1 | 31.66 | Marker13886 | 0.518813291 | 0.017323208 | -0.060860623 | 0.214095098 |
| 158 | 1 | 31.91 | Marker14478 | 0.437026824 | 0.006056202 | -0.081920981 | 0.334705109 |
| 159 | 1 | 31.91 | Marker14243 | 0.437026824 | 0.006056202 | -0.081920981 | 0.334705109 |
| 160 | 1 | 31.91 | Marker14692 | 0.437026824 | 0.006056202 | -0.081920981 | 0.334705109 |
| 161 | 1 | 31.91 | Marker14477 | 0.437026824 | 0.006056202 | -0.081920981 | 0.334705109 |
| 162 | 1 | 32.16 | Marker14476 | 0.456276047 | 0.007119127 | -0.080588514 | 0.325405264 |
| 163 | 1 | 32.41 | Marker14696 | 0.438460946 | 0.008503401 | -0.082242331 | 0.34085987 |
| 164 | 1 | 32.41 | Marker14695 | 0.438460946 | 0.008503401 | -0.082242331 | 0.34085987 |
| 165 | 1 | 32.41 | Marker14694 | 0.438460946 | 0.008503401 | -0.082242331 | 0.34085987 |
| 166 | 1 | 32.41 | Marker14693 | 0.438460946 | 0.008503401 | -0.082242331 | 0.34085987 |
| 167 | 1 | 32.91 | Marker15247 | 0.529993881 | 0.018139535 | -0.059677996 | 0.210185355 |
| 168 | 1 | 32.91 | Marker15246 | 0.529993881 | 0.018139535 | -0.059677996 | 0.210185355 |
| 169 | 1 | 33.41 | Marker15337 | 0.517816241 | 0.017602496 | -0.056818182 | 0.191763676 |
| 170 | 1 | 33.66 | Marker15336 | 0.517816241 | 0.017602496 | -0.056818182 | 0.191763676 |
| 171 | 1 | 33.66 | Marker15341 | 0.517816241 | 0.017602496 | -0.056818182 | 0.191763676 |
| 172 | 1 | 36.064 | Marker11497 | 0.642710197 | 0.016142558 | -0.012140271 | 0.035593609 |
| 173 | 1 | 36.314 | Marker11496 | 0.65179545 | 0.015734395 | -0.013002936 | 0.035217985 |
| 174 | 1 | 37.565 | Marker11333 | 0.534665564 | 0.011735624 | -0.021383221 | 0.037285084 |
| 175 | 1 | 37.815 | Marker11332 | 0.561264245 | 0.016142558 | -0.012140271 | 0.035593609 |
| 176 | 1 | 37.815 | Marker11330 | 0.561264245 | 0.016142558 | -0.012140271 | 0.035593609 |
| 177 | 1 | 37.815 | Marker11331 | 0.561264245 | 0.016142558 | -0.012140271 | 0.035593609 |
| 178 | 1 | 39.773 | Marker14840 | 0.497351998 | -0.005124777 | -0.014483066 | 0.013389136 |
| 179 | 1 | 39.773 | Marker14839 | 0.497351998 | -0.005124777 | -0.014483066 | 0.013389136 |
| 180 | 1 | 40.023 | Marker14841 | 0.497281122 | -0.005882353 | -0.013143079 | 0.012483239 |
| 181 | 1 | 42.375 | Marker15035 | 0.495171055 | -0.020176217 | -0.021140283 | 0.067649814 |
| 182 | 1 | 43.675 | Marker15837 | 0.196561238 | -0.025297278 | -0.052842649 | 0.210819537 |
| 183 | 1 | 43.675 | Marker15845 | 0.196561238 | -0.025297278 | -0.052842649 | 0.210819537 |
| 184 | 1 | 43.675 | Marker15838 | 0.196561238 | -0.025297278 | -0.052842649 | 0.210819537 |
| 185 | 1 | 45.276 | Marker16801 | 0.22006307 | -0.031407994 | -0.024947687 | 0.140718768 |
| 186 | 1 | 45.526 | Marker16802 | 0.20640451 | -0.033671012 | -0.027601945 | 0.164080761 |
| 187 | 1 | 46.326 | Marker17407 | 0.137754384 | -0.04458042 | -0.050431005 | 0.348434666 |
| 188 | 1 | 49.43 | Marker17261 | 0.136258728 | -0.046474359 | -0.046903912 | 0.350321189 |
| 189 | 1 | 49.43 | Marker17259 | 0.136258728 | -0.046474359 | -0.046903912 | 0.350321189 |
| 190 | 1 | 49.43 | Marker17260 | 0.136258728 | -0.046474359 | -0.046903912 | 0.350321189 |
| 191 | 1 | 49.43 | Marker17253 | 0.136258728 | -0.046474359 | -0.046903912 | 0.350321189 |
| 192 | 1 | 49.43 | Marker17250 | 0.136258728 | -0.046474359 | -0.046903912 | 0.350321189 |
| 193 | 1 | 49.43 | Marker17251 | 0.136258728 | -0.046474359 | -0.046903912 | 0.350321189 |
| 194 | 1 | 49.43 | Marker17252 | 0.136258728 | -0.046474359 | -0.046903912 | 0.350321189 |
| 195 | 1 | 52.584 | Marker17015 | 0.104573397 | -0.019620251 | -0.094437726 | 0.486386743 |
| 196 | 1 | 54.135 | Marker16155 | 0.139815288 | -0.022161659 | -0.057420223 | 0.21926688 |
| 197 | 1 | 55.135 | Marker16686 | 0.138077033 | -0.018282989 | -0.062666822 | 0.232904559 |
| 198 | 1 | 55.135 | Marker15846 | 0.138077033 | -0.018282989 | -0.062666822 | 0.232904559 |
| 199 | 1 | 56.135 | Marker17881 | 0.134353697 | -0.018218623 | -0.059782814 | 0.215094408 |
| 200 | 1 | 57.435 | Marker18781 | 0.117935005 | -0.031796502 | -0.039978997 | 0.192531421 |
| 201 | 1 | 57.435 | Marker18782 | 0.117935005 | -0.031796502 | -0.039978997 | 0.192531421 |
| 202 | 1 | 57.935 | Marker20003 | 0.112648979 | -0.02991453 | -0.039598526 | 0.178099015 |
| 203 | 1 | 57.935 | Marker20306 | 0.112648979 | -0.02991453 | -0.039598526 | 0.178099015 |
| 204 | 1 | 57.935 | Marker18561 | 0.112648979 | -0.02991453 | -0.039598526 | 0.178099015 |
| 205 | 1 | 57.935 | Marker20004 | 0.112648979 | -0.02991453 | -0.039598526 | 0.178099015 |
| 206 | 1 | 57.935 | Marker19793 | 0.112648979 | -0.02991453 | -0.039598526 | 0.178099015 |
| 207 | 1 | 58.185 | Marker19798 | 0.109628633 | -0.028825996 | -0.038085255 | 0.165097078 |
| 208 | 1 | 58.185 | Marker19792 | 0.109628633 | -0.028825996 | -0.038085255 | 0.165097078 |
| 209 | 1 | 59.986 | Marker21347 | 0.340565132 | -0.064814815 | -0.008740048 | 0.46701593 |
| 210 | 1 | 60.986 | Marker23842 | 0.531281502 | -0.056603774 | 0.008816787 | 0.355441984 |
| 211 | 1 | 60.986 | Marker22863 | 0.531281502 | -0.056603774 | 0.008816787 | 0.355441984 |
| 212 | 1 | 60.986 | Marker22310 | 0.531281502 | -0.056603774 | 0.008816787 | 0.355441984 |
| 213 | 1 | 60.986 | Marker21970 | 0.531281502 | -0.056603774 | 0.008816787 | 0.355441984 |
| 214 | 1 | 60.986 | Marker22312 | 0.531281502 | -0.056603774 | 0.008816787 | 0.355441984 |
| 215 | 1 | 60.986 | Marker21971 | 0.531281502 | -0.056603774 | 0.008816787 | 0.355441984 |
| 216 | 1 | 60.986 | Marker21794 | 0.531281502 | -0.056603774 | 0.008816787 | 0.355441984 |
| 217 | 1 | 61.486 | Marker21673 | 0.615978058 | -0.06948875 | 0.032290137 | 0.578679028 |
| 218 | 1 | 61.486 | Marker21674 | 0.615978058 | -0.06948875 | 0.032290137 | 0.578679028 |
| 219 | 1 | 61.736 | Marker22298 | 0.602725691 | -0.067591114 | 0.032383928 | 0.550459043 |
| 220 | 1 | 61.736 | Marker22301 | 0.602725691 | -0.067591114 | 0.032383928 | 0.550459043 |
| 221 | 1 | 61.736 | Marker22299 | 0.602725691 | -0.067591114 | 0.032383928 | 0.550459043 |
| 222 | 1 | 61.736 | Marker22300 | 0.602725691 | -0.067591114 | 0.032383928 | 0.550459043 |
| 223 | 1 | 62.836 | Marker24713 | 0.585746126 | -0.035861713 | 0.002637312 | 0.141667914 |
| 224 | 1 | 62.836 | Marker24714 | 0.585746126 | -0.035861713 | 0.002637312 | 0.141667914 |
| 225 | 1 | 62.836 | Marker24716 | 0.585746126 | -0.035861713 | 0.002637312 | 0.141667914 |
| 226 | 1 | 63.636 | Marker23850 | 0.566865562 | -0.036111111 | -0.000825826 | 0.143560512 |
| 227 | 1 | 63.636 | Marker23844 | 0.566865562 | -0.036111111 | -0.000825826 | 0.143560512 |
| 228 | 1 | 63.636 | Marker23849 | 0.566865562 | -0.036111111 | -0.000825826 | 0.143560512 |
| 229 | 1 | 63.636 | Marker23843 | 0.566865562 | -0.036111111 | -0.000825826 | 0.143560512 |
| 230 | 1 | 63.636 | Marker23845 | 0.566865562 | -0.036111111 | -0.000825826 | 0.143560512 |
| 231 | 1 | 63.636 | Marker24125 | 0.566865562 | -0.036111111 | -0.000825826 | 0.143560512 |
| 232 | 1 | 63.636 | Marker24479 | 0.566865562 | -0.036111111 | -0.000825826 | 0.143560512 |
| 233 | 1 | 63.886 | Marker24408 | 0.420105644 | -0.035130719 | 0.000727867 | 0.135767033 |
| 234 | 1 | 63.886 | Marker24407 | 0.420105644 | -0.035130719 | 0.000727867 | 0.135767033 |
| 235 | 1 | 63.886 | Marker25629 | 0.420105644 | -0.035130719 | 0.000727867 | 0.135767033 |
| 236 | 1 | 63.886 | Marker24409 | 0.420105644 | -0.035130719 | 0.000727867 | 0.135767033 |
| 237 | 1 | 63.886 | Marker25630 | 0.420105644 | -0.035130719 | 0.000727867 | 0.135767033 |
| 238 | 1 | 64.136 | Marker25742 | 0.441867067 | -0.034188034 | 0.002254372 | 0.128708134 |
| 239 | 1 | 64.386 | Marker26309 | 0.480867689 | -0.026847153 | 0.016642749 | 0.092169111 |
| 240 | 1 | 64.886 | Marker26492 | 0.545158302 | -0.019353637 | 0.031236347 | 0.088312163 |
| 241 | 1 | 65.136 | Marker26404 | 0.569478922 | -0.014957265 | 0.039833765 | 0.101920865 |
| 242 | 1 | 65.636 | Marker26369 | 0.586824354 | -0.013888889 | 0.042445483 | 0.109158831 |
| 243 | 1 | 66.636 | Marker23039 | 0.313442511 | -0.037735849 | 0.0463763 | 0.259734884 |
| 244 | 1 | 67.387 | Marker23040 | 0.458694209 | -0.051624738 | 0.068745632 | 0.52023033 |
| 245 | 1 | 67.387 | Marker28011 | 0.458694209 | -0.051624738 | 0.068745632 | 0.52023033 |
| 246 | 1 | 67.637 | Marker28012 | 0.421403041 | -0.04536561 | 0.058664732 | 0.391602124 |
| 247 | 1 | 68.137 | Marker28010 | 0.520078754 | -0.044865762 | 0.081834115 | 0.545626846 |
| 248 | 1 | 68.637 | Marker27235 | 0.465060039 | -0.052021563 | 0.068292984 | 0.521683944 |
| 249 | 1 | 69.137 | Marker27845 | 0.470708118 | -0.028846154 | 0.061243936 | 0.273637425 |
| 250 | 1 | 69.137 | Marker27844 | 0.470708118 | -0.028846154 | 0.061243936 | 0.273637425 |
| 251 | 1 | 69.137 | Marker27846 | 0.470708118 | -0.028846154 | 0.061243936 | 0.273637425 |
| 252 | 1 | 69.387 | Marker26904 | 0.558499511 | -0.019607843 | 0.078606443 | 0.344769076 |
| 253 | 1 | 71.187 | Marker28060 | 0.962705873 | 0.03030303 | 0.070614401 | 0.351313932 |
| 254 | 1 | 71.187 | Marker28059 | 0.962705873 | 0.03030303 | 0.070614401 | 0.351313932 |
| 255 | 1 | 71.187 | Marker28051 | 0.962705873 | 0.03030303 | 0.070614401 | 0.351313932 |
| 256 | 1 | 71.187 | Marker28053 | 0.962705873 | 0.03030303 | 0.070614401 | 0.351313932 |
| 257 | 1 | 71.187 | Marker28061 | 0.962705873 | 0.03030303 | 0.070614401 | 0.351313932 |
| 258 | 1 | 71.187 | Marker28054 | 0.962705873 | 0.03030303 | 0.070614401 | 0.351313932 |
| 259 | 1 | 71.187 | Marker28052 | 0.962705873 | 0.03030303 | 0.070614401 | 0.351313932 |
| 260 | 1 | 71.187 | Marker28049 | 0.962705873 | 0.03030303 | 0.070614401 | 0.351313932 |
| 261 | 1 | 73.239 | Marker28807 | 0.835637881 | 0.015625 | 0.09056969 | 0.434735956 |
| 262 | 1 | 73.489 | Marker28808 | 0.442618518 | 0.005821078 | 0.071062307 | 0.253986785 |
| 263 | 1 | 74.239 | Marker29129 | 0.563856324 | -0.009615385 | 0.096579306 | 0.469326606 |
| 264 | 1 | 75.239 | Marker29323 | 0.68066391 | -0.025562995 | 0.121739318 | 0.798447382 |
| 265 | 1 | 75.989 | Marker29922 | 0.614375177 | -0.03627451 | 0.100507099 | 0.637202877 |
| 266 | 1 | 75.989 | Marker29867 | 0.614375177 | -0.03627451 | 0.100507099 | 0.637202877 |
| 267 | 1 | 75.989 | Marker29921 | 0.614375177 | -0.03627451 | 0.100507099 | 0.637202877 |
| 268 | 1 | 76.489 | Marker30794 | 0.631166867 | -0.029808655 | 0.105022516 | 0.636987325 |
| 269 | 1 | 76.989 | Marker30795 | 0.259980188 | -0.038440545 | 0.071538806 | 0.410405951 |
| 270 | 1 | 78.039 | Marker31625 | 0.273800413 | -0.023927126 | 0.09627217 | 0.516701279 |
| 271 | 1 | 80.441 | Marker31209 | 0.162546578 | -0.00862069 | 0.11137931 | 0.619339704 |
| 272 | 1 | 80.441 | Marker31210 | 0.162546578 | -0.00862069 | 0.11137931 | 0.619339704 |
| 273 | 1 | 80.441 | Marker31204 | 0.162546578 | -0.00862069 | 0.11137931 | 0.619339704 |
| 274 | 1 | 80.441 | Marker31211 | 0.162546578 | -0.00862069 | 0.11137931 | 0.619339704 |
| 275 | 1 | 80.441 | Marker31205 | 0.162546578 | -0.00862069 | 0.11137931 | 0.619339704 |
| 276 | 1 | 80.441 | Marker31208 | 0.162546578 | -0.00862069 | 0.11137931 | 0.619339704 |
| 277 | 1 | 82.592 | Marker31626 | 0.352225917 | -0.029834421 | 0.132470829 | 0.957731597 |
| 278 | 1 | 83.392 | Marker33440 | 0.280323263 | -0.004004157 | 0.089237823 | 0.394563119 |
| 279 | 1 | 83.392 | Marker33443 | 0.280323263 | -0.004004157 | 0.089237823 | 0.394563119 |
| 280 | 1 | 84.192 | Marker32666 | 0.310948158 | -0.01 | 0.094424881 | 0.449796638 |
| 281 | 1 | 84.442 | Marker31620 | 0.470326102 | -0.026129032 | 0.118197998 | 0.759728373 |
| 282 | 1 | 85.192 | Marker33627 | 0.425240877 | -0.01 | 0.092564103 | 0.432639509 |
| 283 | 1 | 85.942 | Marker33884 | 0.337208842 | -0.009615385 | 0.095647773 | 0.460495765 |
| 284 | 1 | 85.942 | Marker33928 | 0.337208842 | -0.009615385 | 0.095647773 | 0.460495765 |
| 285 | 1 | 85.942 | Marker33927 | 0.337208842 | -0.009615385 | 0.095647773 | 0.460495765 |
| 286 | 1 | 85.942 | Marker33926 | 0.337208842 | -0.009615385 | 0.095647773 | 0.460495765 |
| 287 | 1 | 86.442 | Marker34682 | 0.454676587 | -0.009803922 | 0.093644354 | 0.44216457 |
| 288 | 1 | 87.192 | Marker35457 | 0.272423233 | -0.003333333 | 0.048803419 | 0.118602821 |
| 289 | 1 | 88.743 | Marker34149 | 0.047757559 | 0.012103062 | 0.029023317 | 0.058378262 |
| 290 | 1 | 90.043 | Marker34613 | 0.028753274 | 0.021056246 | 0.007829302 | 0.052110974 |
| 291 | 1 | 90.043 | Marker34612 | 0.028753274 | 0.021056246 | 0.007829302 | 0.052110974 |
| 292 | 1 | 91.844 | Marker37426 | 0.033029346 | 0.034198113 | -0.037230458 | 0.194828433 |
| 293 | 1 | 91.844 | Marker37427 | 0.033029346 | 0.034198113 | -0.037230458 | 0.194828433 |
| 294 | 1 | 93.645 | Marker37740 | 0.038566729 | 0.003641457 | -0.028018775 | 0.040059854 |
| 295 | 1 | 95.997 | Marker38600 | 0.069605752 | 0.034313725 | -0.077030812 | 0.417881424 |
| 296 | 1 | 96.797 | Marker38313 | 0.05105672 | 0.004897299 | -0.02743879 | 0.039591307 |
| 297 | 1 | 99.549 | Marker40298 | 0.616124701 | 0.089696644 | -0.165168342 | 2.205955444 |
| 298 | 1 | 99.799 | Marker40300 | 0.610887108 | 0.090684785 | -0.164758859 | 2.218660657 |
| 299 | 1 | 99.799 | Marker40299 | 0.610887108 | 0.090684785 | -0.164758859 | 2.218660657 |
| 300 | 1 | 100.049 | Marker40296 | 0.653680333 | 0.094418999 | -0.168493073 | 2.354424538 |
| 301 | 1 | 103.309 | Marker46916 | 0.378298839 | 0.061831814 | -0.117316893 | 1.087376609 |
| 302 | 1 | 103.309 | Marker46917 | 0.378298839 | 0.061831814 | -0.117316893 | 1.087376609 |
| 303 | 1 | 103.559 | Marker46919 | 0.381761894 | 0.064708113 | -0.120364475 | 1.162231877 |
| 304 | 1 | 105.16 | Marker43313 | 0.394550626 | 0.090778206 | -0.106225309 | 1.446532226 |
| 305 | 1 | 106.21 | Marker45544 | 0.525660604 | 0.078977274 | -0.099994702 | 1.165836322 |
| 306 | 1 | 107.26 | Marker46223 | 0.470283934 | 0.080553102 | -0.076535159 | 0.992052454 |
| 307 | 1 | 108.06 | Marker46215 | 0.388468493 | 0.070320051 | -0.061755852 | 0.724540914 |
| 308 | 1 | 109.11 | Marker41705 | 0.363385869 | 0.069677871 | -0.0580461 | 0.69323639 |
| 309 | 1 | 111.713 | Marker50196 | 0.363976151 | 0.060077519 | -0.058202051 | 0.558078482 |
| 310 | 1 | 111.713 | Marker50195 | 0.363976151 | 0.060077519 | -0.058202051 | 0.558078482 |
| 311 | 1 | 112.763 | Marker50292 | 0.642541391 | 0.094941349 | -0.146729725 | 2.029975219 |
| 312 | 1 | 112.763 | Marker50291 | 0.642541391 | 0.094941349 | -0.146729725 | 2.029975219 |
| 313 | 1 | 113.813 | Marker49625 | 0.606761121 | 0.094925039 | -0.110674631 | 1.577361665 |
| 314 | 1 | 115.113 | Marker49937 | 0.398691103 | 0.05 | -0.07568306 | 0.551138315 |
| 315 | 1 | 115.464 | Marker49934 | 0.312700977 | 0.020071685 | -0.067638259 | 0.267878295 |
| 316 | 1 | 117.816 | Marker51959 | 0.769916798 | 0.046266169 | -0.141236129 | 1.209122369 |
| 317 | 1 | 118.066 | Marker51960 | 0.747823532 | 0.047322078 | -0.140101705 | 1.204051527 |
| 318 | 1 | 118.316 | Marker51967 | 0.815422075 | 0.035883299 | -0.160452451 | 1.403133238 |
| 319 | 1 | 120.969 | Marker61340 | 0.235104081 | 0.014234954 | -0.090194729 | 0.421881322 |
| 320 | 1 | 121.469 | Marker61341 | 0.188233365 | 0.021843736 | -0.07021658 | 0.293289233 |
| 321 | 1 | 121.469 | Marker61345 | 0.188233365 | 0.021843736 | -0.07021658 | 0.293289233 |
| 322 | 1 | 121.469 | Marker61344 | 0.188233365 | 0.021843736 | -0.07021658 | 0.293289233 |
| 323 | 1 | 121.469 | Marker61347 | 0.188233365 | 0.021843736 | -0.07021658 | 0.293289233 |
| 324 | 1 | 122.519 | Marker60529 | 0.338159897 | 0.031221614 | -0.090797609 | 0.509389825 |
| 325 | 1 | 124.119 | Marker68842 | 0.14346031 | 0.00534188 | -0.108956845 | 0.588622993 |
| 326 | 1 | 124.369 | Marker54367 | 0.431810413 | 0.019230769 | -0.131042455 | 0.884513409 |
| 327 | 1 | 124.369 | Marker54366 | 0.431810413 | 0.019230769 | -0.131042455 | 0.884513409 |
| 328 | 1 | 124.369 | Marker64748 | 0.431810413 | 0.019230769 | -0.131042455 | 0.884513409 |
| 329 | 1 | 124.369 | Marker64767 | 0.431810413 | 0.019230769 | -0.131042455 | 0.884513409 |
| 330 | 1 | 124.369 | Marker68123 | 0.431810413 | 0.019230769 | -0.131042455 | 0.884513409 |
| 331 | 1 | 124.619 | Marker59328 | 0.4352048 | 0.021611722 | -0.133556687 | 0.927411347 |
| 332 | 1 | 124.869 | Marker68843 | 0.439314388 | 0.02413273 | -0.136208826 | 0.974715299 |
| 333 | 1 | 125.119 | Marker67910 | 0.514479053 | 0.041958042 | -0.16216317 | 1.480513093 |
| 334 | 1 | 125.119 | Marker68070 | 0.514479053 | 0.041958042 | -0.16216317 | 1.480513093 |
| 335 | 1 | 125.119 | Marker67909 | 0.514479053 | 0.041958042 | -0.16216317 | 1.480513093 |
| 336 | 1 | 125.119 | Marker67962 | 0.514479053 | 0.041958042 | -0.16216317 | 1.480513093 |
| 337 | 1 | 125.119 | Marker68601 | 0.514479053 | 0.041958042 | -0.16216317 | 1.480513093 |
| 338 | 1 | 125.119 | Marker55851 | 0.514479053 | 0.041958042 | -0.16216317 | 1.480513093 |
| 339 | 1 | 125.119 | Marker69756 | 0.514479053 | 0.041958042 | -0.16216317 | 1.480513093 |
| 340 | 1 | 125.119 | Marker60207 | 0.514479053 | 0.041958042 | -0.16216317 | 1.480513093 |
| 341 | 1 | 125.119 | Marker67963 | 0.514479053 | 0.041958042 | -0.16216317 | 1.480513093 |
| 342 | 1 | 125.619 | Marker64259 | 0.498171888 | 0.040441176 | -0.157311095 | 1.390983025 |
| 343 | 1 | 126.119 | Marker62227 | 0.541984246 | 0.027941176 | -0.177941176 | 1.641198302 |
| 344 | 1 | 126.119 | Marker62226 | 0.541984246 | 0.027941176 | -0.177941176 | 1.641198302 |
| 345 | 1 | 126.119 | Marker64951 | 0.541984246 | 0.027941176 | -0.177941176 | 1.641198302 |
| 346 | 1 | 126.119 | Marker62823 | 0.541984246 | 0.027941176 | -0.177941176 | 1.641198302 |
| 347 | 1 | 126.369 | Marker62785 | 0.532132469 | 0.029770445 | -0.176111908 | 1.620318206 |
| 348 | 1 | 126.619 | Marker62786 | 0.693329965 | 0.026829268 | -0.173170732 | 1.552338191 |
| 349 | 1 | 126.869 | Marker62787 | 0.725932207 | 0.016666667 | -0.191666667 | 1.839764293 |
| 350 | 1 | 128.17 | Marker71065 | 0.994660715 | 0.036111111 | -0.194375574 | 1.997343832 |
| 351 | 1 | 128.17 | Marker70952 | 0.994660715 | 0.036111111 | -0.194375574 | 1.997343832 |
| 352 | 1 | 129.22 | Marker72924 | 0.927421072 | 0.017763158 | -0.209569881 | 2.197868019 |
| 353 | 1 | 129.22 | Marker72926 | 0.927421072 | 0.017763158 | -0.209569881 | 2.197868019 |
| 354 | 1 | 129.97 | Marker75362 | 1.06344491 | 0.007702182 | -0.228247862 | 2.577308595 |
| 355 | 1 | 129.97 | Marker75611 | 1.06344491 | 0.007702182 | -0.228247862 | 2.577308595 |
| 356 | 1 | 129.97 | Marker75357 | 1.06344491 | 0.007702182 | -0.228247862 | 2.577308595 |
| 357 | 1 | 129.97 | Marker75363 | 1.06344491 | 0.007702182 | -0.228247862 | 2.577308595 |
| 358 | 1 | 130.22 | Marker73344 | 0.884098039 | 0.017763158 | -0.209569881 | 2.197868019 |
| 359 | 1 | 130.22 | Marker73089 | 0.884098039 | 0.017763158 | -0.209569881 | 2.197868019 |
| 360 | 1 | 130.47 | Marker73193 | 0.945858552 | 0.020608108 | -0.212274775 | 2.265062099 |
| 361 | 1 | 130.47 | Marker74445 | 0.945858552 | 0.020608108 | -0.212274775 | 2.265062099 |
| 362 | 1 | 130.47 | Marker74121 | 0.945858552 | 0.020608108 | -0.212274775 | 2.265062099 |
| 363 | 1 | 130.72 | Marker73188 | 1.084092006 | 0.030921053 | -0.231131137 | 2.731556741 |
| 364 | 1 | 130.97 | Marker72538 | 1.070271195 | 0.045250321 | -0.208540937 | 2.356724023 |
| 365 | 1 | 131.47 | Marker72971 | 1.038673891 | 0.043956044 | -0.204054566 | 2.253419226 |
| 366 | 1 | 131.97 | Marker72627 | 0.943157085 | 0.035428099 | -0.190695068 | 1.922426048 |
| 367 | 1 | 132.77 | Marker74316 | 1.02869809 | 0.045833333 | -0.19702381 | 2.132529504 |
| 368 | 1 | 133.02 | Marker74738 | 1.027391985 | 0.044318182 | -0.198380933 | 2.144450059 |
| 369 | 1 | 133.27 | Marker74739 | 1.112085488 | 0.053769401 | -0.216918752 | 2.62156998 |
| 370 | 1 | 133.52 | Marker75729 | 1.107187543 | 0.066395664 | -0.195524793 | 2.350125361 |
| 371 | 1 | 134.02 | Marker78226 | 1.112409922 | 0.068181818 | -0.199517297 | 2.453361435 |
| 372 | 1 | 134.27 | Marker78227 | 1.138070617 | 0.069444444 | -0.198412698 | 2.450442388 |
| 373 | 1 | 135.27 | Marker77508 | 1.189503034 | 0.088775357 | -0.197387778 | 2.76005839 |
| 374 | 1 | 136.32 | Marker78437 | 0.997700673 | 0.071358325 | -0.165353332 | 1.889542422 |
| 375 | 1 | 136.57 | Marker79178 | 1.002807839 | 0.070575066 | -0.17265702 | 1.998578719 |
| 376 | 1 | 137.37 | Marker80200 | 0.900275451 | 0.059196617 | -0.150105708 | 1.482530409 |
| 377 | 1 | 137.37 | Marker80201 | 0.900275451 | 0.059196617 | -0.150105708 | 1.482530409 |
| 378 | 1 | 138.17 | Marker83441 | 0.827728283 | 0.067389006 | -0.128065539 | 1.294180974 |
| 379 | 1 | 138.67 | Marker82034 | 0.829744084 | 0.068936877 | -0.131436877 | 1.359834928 |
| 380 | 1 | 138.67 | Marker82474 | 0.829744084 | 0.068936877 | -0.131436877 | 1.359834928 |
| 381 | 1 | 138.92 | Marker82473 | 0.829744084 | 0.068936877 | -0.131436877 | 1.359834928 |
| 382 | 1 | 140.471 | Marker84258 | 0.532673251 | 0.057032115 | -0.15227021 | 1.487556621 |
| 383 | 1 | 140.471 | Marker84259 | 0.532673251 | 0.057032115 | -0.15227021 | 1.487556621 |
| 384 | 1 | 140.971 | Marker84324 | 0.490451227 | 0.034262485 | -0.156213705 | 1.325047564 |
| 385 | 1 | 141.221 | Marker83858 | 0.48964493 | 0.032738095 | -0.157738095 | 1.337800527 |
| 386 | 1 | 141.221 | Marker83859 | 0.48964493 | 0.032738095 | -0.157738095 | 1.337800527 |
| 387 | 1 | 141.221 | Marker84256 | 0.48964493 | 0.032738095 | -0.157738095 | 1.337800527 |
| 388 | 1 | 141.721 | Marker85258 | 0.481435532 | 0.019852524 | -0.175043545 | 1.550868496 |
| 389 | 1 | 141.721 | Marker85012 | 0.481435532 | 0.019852524 | -0.175043545 | 1.550868496 |
| 390 | 1 | 141.721 | Marker85011 | 0.481435532 | 0.019852524 | -0.175043545 | 1.550868496 |
| 391 | 1 | 141.721 | Marker84928 | 0.481435532 | 0.019852524 | -0.175043545 | 1.550868496 |
| 392 | 1 | 141.721 | Marker85010 | 0.481435532 | 0.019852524 | -0.175043545 | 1.550868496 |
| 393 | 1 | 141.971 | Marker85829 | 0.596027363 | 0.008296666 | -0.192926494 | 1.843631203 |
| 394 | 1 | 142.221 | Marker85552 | 0.639581064 | 0.005555556 | -0.19040404 | 1.792672924 |
| 395 | 1 | 142.221 | Marker85551 | 0.639581064 | 0.005555556 | -0.19040404 | 1.792672924 |
| 396 | 1 | 142.721 | Marker85557 | 0.558734269 | -0.008865248 | -0.167060678 | 1.390290793 |
| 397 | 1 | 142.721 | Marker85976 | 0.558734269 | -0.008865248 | -0.167060678 | 1.390290793 |
| 398 | 1 | 142.971 | Marker85978 | 0.560748505 | -0.006927264 | -0.165209228 | 1.355912832 |
| 399 | 1 | 142.971 | Marker85977 | 0.560748505 | -0.006927264 | -0.165209228 | 1.355912832 |
| 400 | 1 | 142.971 | Marker85973 | 0.560748505 | -0.006927264 | -0.165209228 | 1.355912832 |
| 401 | 1 | 143.221 | Marker84348 | 0.462379904 | -0.018895349 | -0.143895349 | 1.067287702 |
| 402 | 1 | 143.471 | Marker86020 | 0.465066324 | -0.017565562 | -0.145225136 | 1.08064149 |
| 403 | 1 | 143.471 | Marker86021 | 0.465066324 | -0.017565562 | -0.145225136 | 1.08064149 |
| 404 | 1 | 143.721 | Marker83490 | 0.359788257 | -0.029312016 | -0.12404472 | 0.861426551 |
| 405 | 2 | 0 | Marker86654 | 1.317298841 | -0.072222222 | -0.117573696 | 1.272418953 |
| 406 | 2 | 0 | Marker86655 | 1.317298841 | -0.072222222 | -0.117573696 | 1.272418953 |
| 407 | 2 | 0.25 | Marker86657 | 1.128167142 | -0.072727273 | -0.116963449 | 1.273429371 |
| 408 | 2 | 0.75 | Marker88406 | 1.019453969 | -0.079968944 | -0.094630598 | 1.159896628 |
| 409 | 2 | 0.75 | Marker87144 | 1.019453969 | -0.079968944 | -0.094630598 | 1.159896628 |
| 410 | 2 | 0.75 | Marker88439 | 1.019453969 | -0.079968944 | -0.094630598 | 1.159896628 |
| 411 | 2 | 0.75 | Marker87141 | 1.019453969 | -0.079968944 | -0.094630598 | 1.159896628 |
| 412 | 2 | 0.75 | Marker87804 | 1.019453969 | -0.079968944 | -0.094630598 | 1.159896628 |
| 413 | 2 | 1 | Marker88915 | 0.881573793 | -0.089054157 | -0.074683123 | 1.160304049 |
| 414 | 2 | 1 | Marker88914 | 0.881573793 | -0.089054157 | -0.074683123 | 1.160304049 |
| 415 | 2 | 1 | Marker88917 | 0.881573793 | -0.089054157 | -0.074683123 | 1.160304049 |
| 416 | 2 | 1 | Marker88440 | 0.881573793 | -0.089054157 | -0.074683123 | 1.160304049 |
| 417 | 2 | 1 | Marker88913 | 0.881573793 | -0.089054157 | -0.074683123 | 1.160304049 |
| 418 | 2 | 1.5 | Marker90341 | 0.827372616 | -0.089205397 | -0.074188712 | 1.15957365 |
| 419 | 2 | 1.75 | Marker88918 | 0.774301219 | -0.080268199 | -0.054956387 | 0.866174485 |
| 420 | 2 | 2.001 | Marker90343 | 0.830555417 | -0.071939736 | -0.073579977 | 0.84658882 |
| 421 | 2 | 2.501 | Marker90892 | 0.788880141 | -0.071939736 | -0.073579977 | 0.84658882 |
| 422 | 2 | 2.501 | Marker91210 | 0.788880141 | -0.071939736 | -0.073579977 | 0.84658882 |
| 423 | 2 | 2.501 | Marker90758 | 0.788880141 | -0.071939736 | -0.073579977 | 0.84658882 |
| 424 | 2 | 2.751 | Marker91945 | 0.791831008 | -0.072222222 | -0.07294686 | 0.846440268 |
| 425 | 2 | 3.501 | Marker91946 | 0.695594126 | -0.062878788 | -0.053201369 | 0.580969688 |
| 426 | 2 | 3.501 | Marker91953 | 0.695594126 | -0.062878788 | -0.053201369 | 0.580969688 |
| 427 | 2 | 3.501 | Marker91954 | 0.695594126 | -0.062878788 | -0.053201369 | 0.580969688 |
| 428 | 2 | 3.501 | Marker91952 | 0.695594126 | -0.062878788 | -0.053201369 | 0.580969688 |
| 429 | 2 | 3.751 | Marker93317 | 0.743091372 | -0.072222222 | -0.07294686 | 0.846440268 |
| 430 | 2 | 4.001 | Marker93318 | 0.654681152 | -0.062878788 | -0.053201369 | 0.580969688 |
| 431 | 2 | 4.501 | Marker93481 | 0.664837083 | -0.045177278 | -0.051346853 | 0.359065166 |
| 432 | 2 | 5.251 | Marker93872 | 0.730622656 | -0.043591654 | -0.049293721 | 0.333057078 |
| 433 | 2 | 5.251 | Marker94570 | 0.730622656 | -0.043591654 | -0.049293721 | 0.333057078 |
| 434 | 2 | 5.251 | Marker93871 | 0.730622656 | -0.043591654 | -0.049293721 | 0.333057078 |
| 435 | 2 | 5.501 | Marker93870 | 0.708784035 | -0.046920341 | -0.041644197 | 0.331479567 |
| 436 | 2 | 6.001 | Marker94573 | 0.735978449 | -0.042076503 | -0.047300787 | 0.308974546 |
| 437 | 2 | 6.001 | Marker94572 | 0.735978449 | -0.042076503 | -0.047300787 | 0.308974546 |
| 438 | 2 | 6.251 | Marker94800 | 0.803380517 | -0.033333333 | -0.067391304 | 0.350748753 |
| 439 | 2 | 6.501 | Marker96757 | 0.719602362 | -0.023484848 | -0.047140762 | 0.172504537 |
| 440 | 2 | 6.751 | Marker96762 | 0.735556701 | -0.024806202 | -0.048919677 | 0.188162787 |
| 441 | 2 | 7.001 | Marker96764 | 0.517215859 | -0.014285714 | -0.028320802 | 0.062822514 |
| 442 | 2 | 7.001 | Marker96781 | 0.517215859 | -0.014285714 | -0.028320802 | 0.062822514 |
| 443 | 2 | 7.001 | Marker97169 | 0.517215859 | -0.014285714 | -0.028320802 | 0.062822514 |
| 444 | 2 | 7.001 | Marker96782 | 0.517215859 | -0.014285714 | -0.028320802 | 0.062822514 |
| 445 | 2 | 7.001 | Marker96763 | 0.517215859 | -0.014285714 | -0.028320802 | 0.062822514 |
| 446 | 2 | 7.251 | Marker97642 | 0.581191657 | -0.005246166 | -0.048325262 | 0.118875419 |
| 447 | 2 | 7.251 | Marker97172 | 0.581191657 | -0.005246166 | -0.048325262 | 0.118875419 |
| 448 | 2 | 7.501 | Marker97970 | 0.685282883 | 0.002380952 | -0.0660401 | 0.215817646 |
| 449 | 2 | 8.251 | Marker98563 | 0.596892994 | -0.004844961 | -0.045625103 | 0.105836759 |
| 450 | 2 | 8.501 | Marker99624 | 0.532593439 | -0.013724743 | -0.025535072 | 0.05358213 |
| 451 | 2 | 9.501 | Marker100328 | 0.565256727 | -0.013724743 | -0.025535072 | 0.05358213 |
| 452 | 2 | 9.751 | Marker100329 | 0.568041677 | -0.01319767 | -0.024716619 | 0.049944418 |
| 453 | 2 | 10.251 | Marker99625 | 0.51922511 | -0.01399038 | -0.02400999 | 0.050632249 |
| 454 | 2 | 11.001 | Marker100428 | 0.525870044 | -0.011657559 | -0.022286275 | 0.039967226 |
| 455 | 2 | 11.001 | Marker100697 | 0.525870044 | -0.011657559 | -0.022286275 | 0.039967226 |
| 456 | 2 | 11.251 | Marker100698 | 0.531604558 | -0.010691376 | -0.020709591 | 0.034171821 |
| 457 | 2 | 11.501 | Marker100865 | 0.536379681 | -0.009766306 | -0.019160301 | 0.028974988 |
| 458 | 2 | 11.501 | Marker101223 | 0.536379681 | -0.009766306 | -0.019160301 | 0.028974988 |
| 459 | 2 | 11.501 | Marker100817 | 0.536379681 | -0.009766306 | -0.019160301 | 0.028974988 |
| 460 | 2 | 12.001 | Marker101224 | 0.440571675 | -0.009766306 | -0.019160301 | 0.028974988 |
| 461 | 2 | 12.251 | Marker102421 | 0.415797426 | -0.010295127 | -0.017993074 | 0.027996133 |
| 462 | 2 | 12.251 | Marker101225 | 0.415797426 | -0.010295127 | -0.017993074 | 0.027996133 |
| 463 | 2 | 12.501 | Marker102422 | 0.379281414 | -0.017963028 | 0.000272375 | 0.035496095 |
| 464 | 2 | 12.751 | Marker103054 | 0.378178362 | -0.018888097 | -0.001401758 | 0.039397605 |
| 465 | 2 | 12.751 | Marker103053 | 0.378178362 | -0.018888097 | -0.001401758 | 0.039397605 |
| 466 | 2 | 12.751 | Marker103055 | 0.378178362 | -0.018888097 | -0.001401758 | 0.039397605 |
| 467 | 2 | 13.001 | Marker103056 | 0.377207221 | -0.019854281 | -0.003100542 | 0.043958129 |
| 468 | 2 | 13.001 | Marker104726 | 0.377207221 | -0.019854281 | -0.003100542 | 0.043958129 |
| 469 | 2 | 14.001 | Marker105489 | 0.338732457 | -0.028571429 | 0.018334225 | 0.105464782 |
| 470 | 2 | 14.252 | Marker104739 | 0.213350722 | -0.029829545 | 0.01757214 | 0.11219621 |
| 471 | 2 | 15.503 | Marker105893 | 0.242314839 | -0.035541753 | 0.039494322 | 0.2134813 |
| 472 | 2 | 15.503 | Marker105490 | 0.242314839 | -0.035541753 | 0.039494322 | 0.2134813 |
| 473 | 2 | 15.503 | Marker105894 | 0.242314839 | -0.035541753 | 0.039494322 | 0.2134813 |
| 474 | 2 | 16.003 | Marker107618 | 0.292780745 | -0.02708248 | 0.022906828 | 0.105488179 |
| 475 | 2 | 16.753 | Marker108556 | 0.253021388 | -0.052556818 | -0.027627043 | 0.344277171 |
| 476 | 2 | 17.753 | Marker109936 | 0.138370275 | -0.049005682 | 0.014021004 | 0.27268378 |
| 477 | 2 | 17.753 | Marker109036 | 0.138370275 | -0.049005682 | 0.014021004 | 0.27268378 |
| 478 | 2 | 17.753 | Marker108842 | 0.138370275 | -0.049005682 | 0.014021004 | 0.27268378 |
| 479 | 2 | 17.753 | Marker109038 | 0.138370275 | -0.049005682 | 0.014021004 | 0.27268378 |
| 480 | 2 | 17.753 | Marker108848 | 0.138370275 | -0.049005682 | 0.014021004 | 0.27268378 |
| 481 | 2 | 18.003 | Marker110302 | 0.124139619 | -0.056818182 | 0.034090909 | 0.409066757 |
| 482 | 2 | 18.253 | Marker111285 | 0.124731926 | -0.056818182 | 0.035135841 | 0.412532132 |
| 483 | 2 | 18.503 | Marker110305 | 0.117781086 | -0.064280868 | 0.055295668 | 0.599186428 |
| 484 | 2 | 19.253 | Marker114254 | 0.085045923 | -0.045454545 | 0.05799373 | 0.388669525 |
| 485 | 2 | 20.753 | Marker115703 | 0.053985693 | -0.035714286 | 0.074175824 | 0.407332561 |
| 486 | 2 | 20.753 | Marker115675 | 0.053985693 | -0.035714286 | 0.074175824 | 0.407332561 |
| 487 | 2 | 21.003 | Marker116061 | 0.055592313 | -0.035714286 | 0.075396825 | 0.416276017 |
| 488 | 2 | 21.003 | Marker115704 | 0.055592313 | -0.035714286 | 0.075396825 | 0.416276017 |
| 489 | 2 | 21.003 | Marker116750 | 0.055592313 | -0.035714286 | 0.075396825 | 0.416276017 |
| 490 | 2 | 21.003 | Marker119677 | 0.055592313 | -0.035714286 | 0.075396825 | 0.416276017 |
| 491 | 2 | 21.003 | Marker115944 | 0.055592313 | -0.035714286 | 0.075396825 | 0.416276017 |
| 492 | 2 | 21.253 | Marker117997 | 0.056539065 | -0.036585366 | 0.073304744 | 0.407854519 |
| 493 | 2 | 21.503 | Marker121338 | 0.056998659 | -0.036585366 | 0.074525745 | 0.41669093 |
| 494 | 2 | 22.003 | Marker122978 | 0.064714571 | -0.034113478 | 0.064515516 | 0.329657827 |
| 495 | 2 | 22.605 | Marker121340 | 0.050082724 | -0.0375 | 0.073611111 | 0.417389971 |
| 496 | 2 | 22.956 | Marker129959 | 0.059648467 | -0.025641026 | 0.095238095 | 0.516010398 |
| 497 | 2 | 23.206 | Marker127547 | 0.070278713 | -0.018288084 | 0.078117144 | 0.335683811 |
| 498 | 2 | 23.206 | Marker127500 | 0.070278713 | -0.018288084 | 0.078117144 | 0.335683811 |
| 499 | 2 | 23.206 | Marker127499 | 0.070278713 | -0.018288084 | 0.078117144 | 0.335683811 |
| 500 | 2 | 23.706 | Marker129962 | 0.040151492 | -0.029789165 | 0.107596186 | 0.663759396 |
| 501 | 2 | 24.206 | Marker130319 | 0.052068556 | -0.025641026 | 0.096581197 | 0.528676426 |
| 502 | 2 | 24.456 | Marker130320 | 0.055180809 | -0.025641026 | 0.095238095 | 0.516010398 |
| 503 | 2 | 25.31 | Marker134850 | 0.02267325 | -0.038148023 | 0.072459803 | 0.414467561 |
| 504 | 2 | 27.361 | Marker138849 | 0.116650845 | -0.012820513 | 0.116211745 | 0.682617452 |
| 505 | 2 | 28.111 | Marker138850 | 0.225264395 | -0.012820513 | 0.11761427 | 0.69878765 |
| 506 | 2 | 28.861 | Marker138853 | 0.255030521 | -0.020283199 | 0.137499317 | 0.974266684 |
| 507 | 2 | 29.963 | Marker141755 | 0.107577315 | -0.026545595 | 0.096717293 | 0.535003549 |
| 508 | 2 | 30.963 | Marker138854 | 0.222908616 | -0.012820513 | 0.119047619 | 0.71551407 |
| 509 | 2 | 31.463 | Marker140657 | 0.125669737 | -0.012931724 | 0.118463933 | 0.708969255 |
| 510 | 2 | 32.713 | Marker144186 | 0.244941764 | -0.017037303 | 0.081893179 | 0.360735926 |
| 511 | 2 | 32.713 | Marker144185 | 0.244941764 | -0.017037303 | 0.081893179 | 0.360735926 |
| 512 | 2 | 32.713 | Marker143435 | 0.244941764 | -0.017037303 | 0.081893179 | 0.360735926 |
| 513 | 2 | 32.713 | Marker144184 | 0.244941764 | -0.017037303 | 0.081893179 | 0.360735926 |
| 514 | 2 | 32.963 | Marker144187 | 0.04820619 | -0.030147059 | 0.056270654 | 0.253334195 |
| 515 | 2 | 34.714 | Marker144190 | 0.019366083 | -0.078947368 | -0.001169591 | 0.685979754 |
| 516 | 2 | 34.964 | Marker148203 | 0.086134297 | -0.070772199 | 0.013674592 | 0.558544104 |
| 517 | 2 | 35.964 | Marker148284 | 0.06127966 | -0.076923077 | -8.88E-16 | 0.651027732 |
| 518 | 2 | 35.964 | Marker148204 | 0.06127966 | -0.076923077 | -8.88E-16 | 0.651027732 |
| 519 | 2 | 36.214 | Marker148206 | 0.057086982 | -0.076923077 | 0.000854701 | 0.650943515 |
| 520 | 2 | 36.214 | Marker150077 | 0.057086982 | -0.076923077 | 0.000854701 | 0.650943515 |
| 521 | 2 | 36.214 | Marker148285 | 0.057086982 | -0.076923077 | 0.000854701 | 0.650943515 |
| 522 | 2 | 36.714 | Marker150798 | 0.071397388 | -0.076923077 | 0.000854701 | 0.650943515 |
| 523 | 2 | 36.714 | Marker150215 | 0.071397388 | -0.076923077 | 0.000854701 | 0.650943515 |
| 524 | 2 | 36.714 | Marker150078 | 0.071397388 | -0.076923077 | 0.000854701 | 0.650943515 |
| 525 | 2 | 37.714 | Marker150799 | 0.030316354 | -0.084642577 | -0.045220124 | 0.896285888 |
| 526 | 2 | 37.964 | Marker151035 | 0.022193595 | -0.076953748 | -0.064246026 | 0.864527 |
| 527 | 2 | 37.964 | Marker151735 | 0.022193595 | -0.076953748 | -0.064246026 | 0.864527 |
| 528 | 2 | 37.964 | Marker151029 | 0.022193595 | -0.076953748 | -0.064246026 | 0.864527 |
| 529 | 2 | 38.964 | Marker151936 | 0.061179617 | -0.073144573 | -0.027161919 | 0.628727981 |
| 530 | 2 | 39.464 | Marker153693 | 0.056810137 | -0.062818336 | -0.043530009 | 0.532793921 |
| 531 | 2 | 39.464 | Marker152522 | 0.056810137 | -0.062818336 | -0.043530009 | 0.532793921 |
| 532 | 2 | 39.714 | Marker153101 | 0.056413257 | -0.063074353 | -0.042737051 | 0.532889126 |
| 533 | 2 | 39.964 | Marker154273 | 0.044727222 | -0.055509868 | -0.060279605 | 0.524673532 |
| 534 | 2 | 39.964 | Marker153695 | 0.044727222 | -0.055509868 | -0.060279605 | 0.524673532 |
| 535 | 2 | 39.964 | Marker153694 | 0.044727222 | -0.055509868 | -0.060279605 | 0.524673532 |
| 536 | 2 | 39.964 | Marker153697 | 0.044727222 | -0.055509868 | -0.060279605 | 0.524673532 |
| 537 | 2 | 39.964 | Marker153696 | 0.044727222 | -0.055509868 | -0.060279605 | 0.524673532 |
| 538 | 2 | 40.464 | Marker154463 | 0.042695216 | -0.05475382 | -0.061903803 | 0.525385748 |
| 539 | 2 | 41.214 | Marker154469 | 0.080284143 | -0.046160483 | -0.081122009 | 0.566431146 |
| 540 | 2 | 41.464 | Marker155506 | 0.052276343 | -0.045614035 | -0.081977671 | 0.567799019 |
| 541 | 2 | 41.464 | Marker155524 | 0.052276343 | -0.045614035 | -0.081977671 | 0.567799019 |
| 542 | 2 | 41.464 | Marker155507 | 0.052276343 | -0.045614035 | -0.081977671 | 0.567799019 |
| 543 | 2 | 41.464 | Marker155508 | 0.052276343 | -0.045614035 | -0.081977671 | 0.567799019 |
| 544 | 2 | 41.464 | Marker155526 | 0.052276343 | -0.045614035 | -0.081977671 | 0.567799019 |
| 545 | 2 | 41.464 | Marker155315 | 0.052276343 | -0.045614035 | -0.081977671 | 0.567799019 |
| 546 | 2 | 41.464 | Marker155862 | 0.052276343 | -0.045614035 | -0.081977671 | 0.567799019 |
| 547 | 2 | 41.714 | Marker155797 | 0.043012867 | -0.061261261 | -0.107927928 | 1.000534386 |
| 548 | 2 | 41.964 | Marker156201 | 0.042193623 | -0.06069629 | -0.108690919 | 1.001092662 |
| 549 | 2 | 41.964 | Marker156114 | 0.042193623 | -0.06069629 | -0.108690919 | 1.001092662 |
| 550 | 2 | 41.964 | Marker156294 | 0.042193623 | -0.06069629 | -0.108690919 | 1.001092662 |
| 551 | 2 | 41.964 | Marker155863 | 0.042193623 | -0.06069629 | -0.108690919 | 1.001092662 |
| 552 | 2 | 41.964 | Marker156116 | 0.042193623 | -0.06069629 | -0.108690919 | 1.001092662 |
| 553 | 2 | 41.964 | Marker158069 | 0.042193623 | -0.06069629 | -0.108690919 | 1.001092662 |
| 554 | 2 | 41.964 | Marker156295 | 0.042193623 | -0.06069629 | -0.108690919 | 1.001092662 |
| 555 | 2 | 41.964 | Marker156238 | 0.042193623 | -0.06069629 | -0.108690919 | 1.001092662 |
| 556 | 2 | 41.964 | Marker156199 | 0.042193623 | -0.06069629 | -0.108690919 | 1.001092662 |
| 557 | 2 | 41.964 | Marker156198 | 0.042193623 | -0.06069629 | -0.108690919 | 1.001092662 |
| 558 | 2 | 41.964 | Marker156115 | 0.042193623 | -0.06069629 | -0.108690919 | 1.001092662 |
| 559 | 2 | 41.964 | Marker157013 | 0.042193623 | -0.06069629 | -0.108690919 | 1.001092662 |
| 560 | 2 | 41.964 | Marker156202 | 0.042193623 | -0.06069629 | -0.108690919 | 1.001092662 |
| 561 | 2 | 42.214 | Marker157014 | 0.046983302 | -0.063323917 | -0.111512684 | 1.068496785 |
| 562 | 2 | 42.964 | Marker158070 | 0.051202695 | -0.085249042 | -0.117555704 | 1.50070692 |
| 563 | 2 | 42.964 | Marker159935 | 0.051202695 | -0.085249042 | -0.117555704 | 1.50070692 |
| 564 | 2 | 42.964 | Marker159933 | 0.051202695 | -0.085249042 | -0.117555704 | 1.50070692 |
| 565 | 2 | 43.214 | Marker158457 | 0.044013951 | -0.082246039 | -0.114362334 | 1.407648318 |
| 566 | 2 | 43.214 | Marker158456 | 0.044013951 | -0.082246039 | -0.114362334 | 1.407648318 |
| 567 | 2 | 43.214 | Marker158199 | 0.044013951 | -0.082246039 | -0.114362334 | 1.407648318 |
| 568 | 2 | 44.214 | Marker161066 | 0.100821494 | -0.091101695 | -0.158898305 | 2.187112788 |
| 569 | 2 | 44.964 | Marker161149 | 0.018842268 | -0.086924744 | -0.135026288 | 1.753613681 |
| 570 | 2 | 45.214 | Marker161927 | 0.022353476 | -0.083748754 | -0.141929979 | 1.788716385 |
| 571 | 2 | 46.215 | Marker161928 | 0.116927568 | -0.065922921 | -0.178895006 | 2.080941492 |
| 572 | 2 | 46.715 | Marker162723 | 0.133837843 | -0.05496368 | -0.193025224 | 2.192665983 |
| 573 | 2 | 46.965 | Marker161929 | 0.14485394 | -0.065677966 | -0.213733799 | 2.757335782 |
| 574 | 2 | 47.215 | Marker161930 | 0.130976118 | -0.066666667 | -0.213036304 | 2.757326702 |
| 575 | 2 | 47.465 | Marker162015 | 0.120350645 | -0.055952381 | -0.192226891 | 2.189803157 |
| 576 | 2 | 47.715 | Marker162729 | 0.128377175 | -0.059313725 | -0.195397868 | 2.294687763 |
| 577 | 2 | 47.715 | Marker162730 | 0.128377175 | -0.059313725 | -0.195397868 | 2.294687763 |
| 578 | 2 | 47.715 | Marker162728 | 0.128377175 | -0.059313725 | -0.195397868 | 2.294687763 |
| 579 | 2 | 47.715 | Marker162727 | 0.128377175 | -0.059313725 | -0.195397868 | 2.294687763 |
| 580 | 2 | 48.215 | Marker162824 | 0.191045733 | -0.064141196 | -0.208131305 | 2.61735219 |
| 581 | 2 | 48.715 | Marker162933 | 0.163681686 | -0.056908665 | -0.191464744 | 2.187486447 |
| 582 | 2 | 49.215 | Marker162934 | 0.178829227 | -0.05496368 | -0.193025224 | 2.192665983 |
| 583 | 2 | 49.215 | Marker162935 | 0.178829227 | -0.05496368 | -0.193025224 | 2.192665983 |
| 584 | 2 | 49.465 | Marker163060 | 0.206349888 | -0.047619048 | -0.210364146 | 2.454261463 |
| 585 | 2 | 49.965 | Marker163381 | 0.484864552 | -0.039285714 | -0.228501401 | 2.765954008 |
| 586 | 2 | 50.215 | Marker163863 | 0.441291963 | -0.046489104 | -0.211208538 | 2.459786554 |
| 587 | 2 | 50.715 | Marker163866 | 0.458701459 | -0.064655172 | -0.214471041 | 2.757953957 |
| 588 | 2 | 50.715 | Marker163867 | 0.458701459 | -0.064655172 | -0.214471041 | 2.757953957 |
| 589 | 2 | 50.715 | Marker164461 | 0.458701459 | -0.064655172 | -0.214471041 | 2.757953957 |
| 590 | 2 | 50.965 | Marker164462 | 0.550544046 | -0.05720339 | -0.232012296 | 3.043912372 |
| 591 | 2 | 50.965 | Marker164560 | 0.550544046 | -0.05720339 | -0.232012296 | 3.043912372 |
| 592 | 2 | 50.965 | Marker164559 | 0.550544046 | -0.05720339 | -0.232012296 | 3.043912372 |
| 593 | 2 | 50.965 | Marker164808 | 0.550544046 | -0.05720339 | -0.232012296 | 3.043912372 |
| 594 | 2 | 51.215 | Marker165786 | 0.551593886 | -0.056034483 | -0.232800469 | 3.047034325 |
| 595 | 2 | 51.215 | Marker165444 | 0.551593886 | -0.056034483 | -0.232800469 | 3.047034325 |
| 596 | 2 | 51.215 | Marker165060 | 0.551593886 | -0.056034483 | -0.232800469 | 3.047034325 |
| 597 | 2 | 51.215 | Marker165016 | 0.551593886 | -0.056034483 | -0.232800469 | 3.047034325 |
| 598 | 2 | 51.215 | Marker166446 | 0.551593886 | -0.056034483 | -0.232800469 | 3.047034325 |
| 599 | 2 | 51.215 | Marker165700 | 0.551593886 | -0.056034483 | -0.232800469 | 3.047034325 |
| 600 | 2 | 51.465 | Marker166447 | 0.550544046 | -0.05720339 | -0.232012296 | 3.043912372 |
| 601 | 2 | 51.465 | Marker167034 | 0.550544046 | -0.05720339 | -0.232012296 | 3.043912372 |
| 602 | 2 | 51.715 | Marker167035 | 0.548512641 | -0.060774818 | -0.23520299 | 3.165796195 |
| 603 | 2 | 51.715 | Marker167036 | 0.548512641 | -0.060774818 | -0.23520299 | 3.165796195 |
| 604 | 2 | 51.715 | Marker167255 | 0.548512641 | -0.060774818 | -0.23520299 | 3.165796195 |
| 605 | 2 | 51.965 | Marker167256 | 0.550544046 | -0.05720339 | -0.232012296 | 3.043912372 |
| 606 | 2 | 51.965 | Marker167990 | 0.550544046 | -0.05720339 | -0.232012296 | 3.043912372 |
| 607 | 2 | 51.965 | Marker167346 | 0.550544046 | -0.05720339 | -0.232012296 | 3.043912372 |
| 608 | 2 | 52.215 | Marker167601 | 0.546315858 | -0.056404167 | -0.230762988 | 3.004889175 |
| 609 | 2 | 52.715 | Marker168781 | 0.510018676 | -0.040311498 | -0.205607689 | 2.282645327 |
| 610 | 2 | 52.965 | Marker168864 | 0.575252857 | -0.030521901 | -0.224910001 | 2.614323922 |
| 611 | 2 | 52.965 | Marker168789 | 0.575252857 | -0.030521901 | -0.224910001 | 2.614323922 |
| 612 | 2 | 52.965 | Marker168788 | 0.575252857 | -0.030521901 | -0.224910001 | 2.614323922 |
| 613 | 2 | 53.215 | Marker168865 | 0.577085266 | -0.02916074 | -0.225890428 | 2.626715026 |
| 614 | 2 | 53.215 | Marker168875 | 0.577085266 | -0.02916074 | -0.225890428 | 2.626715026 |
| 615 | 2 | 53.215 | Marker168876 | 0.577085266 | -0.02916074 | -0.225890428 | 2.626715026 |
| 616 | 2 | 53.465 | Marker168877 | 0.650658524 | -0.039473684 | -0.246388029 | 3.188631116 |
| 617 | 2 | 53.965 | Marker169425 | 0.560495803 | -0.049743914 | -0.226799858 | 2.834337123 |
| 618 | 2 | 54.215 | Marker169490 | 0.560771586 | -0.049455535 | -0.22699053 | 2.835362268 |
| 619 | 2 | 54.215 | Marker169489 | 0.560771586 | -0.049455535 | -0.22699053 | 2.835362268 |
| 620 | 2 | 54.215 | Marker169486 | 0.560771586 | -0.049455535 | -0.22699053 | 2.835362268 |
| 621 | 2 | 54.215 | Marker169487 | 0.560771586 | -0.049455535 | -0.22699053 | 2.835362268 |
| 622 | 2 | 54.715 | Marker169834 | 0.468605319 | -0.056062581 | -0.20500968 | 2.443393567 |
| 623 | 2 | 54.715 | Marker169833 | 0.468605319 | -0.056062581 | -0.20500968 | 2.443393567 |
| 624 | 2 | 55.215 | Marker170869 | 0.574714201 | -0.04872712 | -0.226357122 | 2.812946937 |
| 625 | 2 | 56.215 | Marker171167 | 0.525260278 | -0.049455535 | -0.22699053 | 2.835362268 |
| 626 | 2 | 56.715 | Marker171265 | 0.496324854 | -0.036297641 | -0.203931645 | 2.21327799 |
| 627 | 2 | 57.215 | Marker171291 | 0.465057025 | -0.050399885 | -0.193582702 | 2.14884825 |
| 628 | 2 | 57.465 | Marker171673 | 0.444140529 | -0.045941124 | -0.184585192 | 1.931137211 |
| 629 | 2 | 58.215 | Marker171674 | 0.804921631 | -0.039113429 | -0.242160852 | 3.083015238 |
| 630 | 2 | 58.465 | Marker172173 | 0.854833374 | -0.045937653 | -0.256592869 | 3.506748958 |
| 631 | 2 | 59.465 | Marker172307 | 0.8600591 | -0.015254237 | -0.235766171 | 2.778545198 |
| 632 | 2 | 60.715 | Marker172308 | 0.732714501 | -0.036437247 | -0.243836934 | 3.099941845 |
| 633 | 2 | 60.715 | Marker172318 | 0.732714501 | -0.036437247 | -0.243836934 | 3.099941845 |
| 634 | 2 | 60.715 | Marker172454 | 0.732714501 | -0.036437247 | -0.243836934 | 3.099941845 |
| 635 | 2 | 60.965 | Marker172547 | 0.732714501 | -0.036437247 | -0.243836934 | 3.099941845 |
| 636 | 2 | 60.965 | Marker172787 | 0.732714501 | -0.036437247 | -0.243836934 | 3.099941845 |
| 637 | 2 | 60.965 | Marker172549 | 0.732714501 | -0.036437247 | -0.243836934 | 3.099941845 |
| 638 | 2 | 60.965 | Marker172458 | 0.732714501 | -0.036437247 | -0.243836934 | 3.099941845 |
| 639 | 2 | 60.965 | Marker172457 | 0.732714501 | -0.036437247 | -0.243836934 | 3.099941845 |
| 640 | 2 | 60.965 | Marker172548 | 0.732714501 | -0.036437247 | -0.243836934 | 3.099941845 |
| 641 | 2 | 61.215 | Marker173305 | 0.70394941 | -0.021052632 | -0.218947368 | 2.425703926 |
| 642 | 2 | 61.465 | Marker174618 | 0.595346054 | -0.028571429 | -0.201131542 | 2.09906615 |
| 643 | 2 | 62.716 | Marker174664 | 0.423813473 | -0.054784689 | -0.21090357 | 2.549035572 |
| 644 | 2 | 62.966 | Marker174665 | 0.462095658 | -0.071498771 | -0.236866737 | 3.365502663 |
| 645 | 2 | 63.216 | Marker175491 | 0.449135242 | -0.067942584 | -0.233676849 | 3.234846084 |
| 646 | 2 | 63.216 | Marker175492 | 0.449135242 | -0.067942584 | -0.233676849 | 3.234846084 |
| 647 | 2 | 63.216 | Marker175223 | 0.449135242 | -0.067942584 | -0.233676849 | 3.234846084 |
| 648 | 2 | 63.216 | Marker175222 | 0.449135242 | -0.067942584 | -0.233676849 | 3.234846084 |
| 649 | 2 | 63.216 | Marker175297 | 0.449135242 | -0.067942584 | -0.233676849 | 3.234846084 |
| 650 | 2 | 63.216 | Marker175457 | 0.449135242 | -0.067942584 | -0.233676849 | 3.234846084 |
| 651 | 2 | 63.466 | Marker176241 | 0.604624199 | -0.069078947 | -0.232913899 | 3.234778494 |
| 652 | 2 | 64.216 | Marker176242 | 0.581206063 | -0.063596491 | -0.226403509 | 3.00390864 |
| 653 | 2 | 64.216 | Marker176509 | 0.581206063 | -0.063596491 | -0.226403509 | 3.00390864 |
| 654 | 2 | 64.216 | Marker176508 | 0.581206063 | -0.063596491 | -0.226403509 | 3.00390864 |
| 655 | 2 | 64.216 | Marker177361 | 0.581206063 | -0.063596491 | -0.226403509 | 3.00390864 |
| 656 | 2 | 64.466 | Marker176507 | 0.548366272 | -0.063319438 | -0.226580494 | 3.003906743 |
| 657 | 2 | 64.966 | Marker176577 | 0.591606608 | -0.060547711 | -0.223758769 | 2.901886839 |
| 658 | 2 | 65.466 | Marker177579 | 0.603193465 | -0.043534483 | -0.221869558 | 2.658347341 |
| 659 | 2 | 65.466 | Marker177585 | 0.603193465 | -0.043534483 | -0.221869558 | 2.658347341 |
| 660 | 2 | 65.716 | Marker177733 | 0.607120023 | -0.04079058 | -0.219537941 | 2.580751934 |
| 661 | 2 | 65.716 | Marker177986 | 0.607120023 | -0.04079058 | -0.219537941 | 2.580751934 |
| 662 | 2 | 66.216 | Marker178102 | 0.668522673 | -0.032169891 | -0.238362713 | 2.93508375 |
| 663 | 2 | 66.216 | Marker177987 | 0.668522673 | -0.032169891 | -0.238362713 | 2.93508375 |
| 664 | 2 | 66.216 | Marker178282 | 0.668522673 | -0.032169891 | -0.238362713 | 2.93508375 |
| 665 | 2 | 66.216 | Marker178103 | 0.668522673 | -0.032169891 | -0.238362713 | 2.93508375 |
| 666 | 2 | 66.216 | Marker178283 | 0.668522673 | -0.032169891 | -0.238362713 | 2.93508375 |
| 667 | 2 | 66.716 | Marker178384 | 0.673485907 | -0.034219983 | -0.237149094 | 2.922364079 |
| 668 | 2 | 67.216 | Marker178729 | 0.670363788 | -0.034756098 | -0.236839431 | 2.91939371 |
| 669 | 2 | 67.466 | Marker178310 | 0.669136958 | -0.033484911 | -0.237573676 | 2.926554002 |
| 670 | 2 | 67.716 | Marker178314 | 0.676563438 | -0.034756098 | -0.236839431 | 2.91939371 |
| 671 | 2 | 67.966 | Marker178868 | 0.681753118 | -0.032142857 | -0.234774436 | 2.850787798 |
| 672 | 2 | 68.466 | Marker179214 | 0.702563879 | -0.035985606 | -0.236158168 | 2.913530371 |
| 673 | 2 | 68.466 | Marker178913 | 0.702563879 | -0.035985606 | -0.236158168 | 2.913530371 |
| 674 | 2 | 68.466 | Marker178817 | 0.702563879 | -0.035985606 | -0.236158168 | 2.913530371 |
| 675 | 2 | 68.466 | Marker178874 | 0.702563879 | -0.035985606 | -0.236158168 | 2.913530371 |
| 676 | 2 | 68.466 | Marker178873 | 0.702563879 | -0.035985606 | -0.236158168 | 2.913530371 |
| 677 | 2 | 68.716 | Marker179421 | 0.982943526 | -0.026422764 | -0.255589431 | 3.316779535 |
| 678 | 2 | 68.716 | Marker179770 | 0.982943526 | -0.026422764 | -0.255589431 | 3.316779535 |
| 679 | 2 | 68.966 | Marker179771 | 0.984353068 | -0.027788884 | -0.254881205 | 3.307668486 |
| 680 | 2 | 69.216 | Marker179777 | 0.858772113 | -0.034756098 | -0.236839431 | 2.91939371 |
| 681 | 2 | 69.216 | Marker179850 | 0.858772113 | -0.034756098 | -0.236839431 | 2.91939371 |
| 682 | 2 | 69.216 | Marker179772 | 0.858772113 | -0.034756098 | -0.236839431 | 2.91939371 |
| 683 | 2 | 69.216 | Marker180101 | 0.858772113 | -0.034756098 | -0.236839431 | 2.91939371 |
| 684 | 2 | 69.216 | Marker179849 | 0.858772113 | -0.034756098 | -0.236839431 | 2.91939371 |
| 685 | 2 | 69.466 | Marker180102 | 0.976700657 | -0.032142857 | -0.234774436 | 2.850787798 |
| 686 | 2 | 69.966 | Marker181018 | 1.020352343 | -0.044047619 | -0.257205514 | 3.502750945 |
| 687 | 2 | 70.466 | Marker182059 | 0.950801314 | -0.030880671 | -0.232185095 | 2.781607383 |
| 688 | 2 | 71.466 | Marker182530 | 0.670453523 | -0.036854553 | -0.214553036 | 2.438296296 |
| 689 | 2 | 71.716 | Marker182533 | 0.610631524 | -0.046317829 | -0.19489939 | 2.129348222 |
| 690 | 2 | 71.716 | Marker182542 | 0.610631524 | -0.046317829 | -0.19489939 | 2.129348222 |
| 691 | 2 | 71.716 | Marker182532 | 0.610631524 | -0.046317829 | -0.19489939 | 2.129348222 |
| 692 | 2 | 71.716 | Marker182531 | 0.610631524 | -0.046317829 | -0.19489939 | 2.129348222 |
| 693 | 2 | 71.966 | Marker182543 | 0.612259338 | -0.047274114 | -0.194286276 | 2.127693996 |
| 694 | 2 | 71.966 | Marker184013 | 0.612259338 | -0.047274114 | -0.194286276 | 2.127693996 |
| 695 | 2 | 72.966 | Marker184630 | 0.59078693 | -0.040183926 | -0.175990656 | 1.720892911 |
| 696 | 2 | 72.966 | Marker185231 | 0.59078693 | -0.040183926 | -0.175990656 | 1.720892911 |
| 697 | 2 | 73.216 | Marker185230 | 0.672207147 | -0.037861046 | -0.17389174 | 1.663769574 |
| 698 | 2 | 73.216 | Marker185714 | 0.672207147 | -0.037861046 | -0.17389174 | 1.663769574 |
| 699 | 2 | 73.216 | Marker185233 | 0.672207147 | -0.037861046 | -0.17389174 | 1.663769574 |
| 700 | 2 | 73.466 | Marker186663 | 0.617432336 | -0.045238095 | -0.155764411 | 1.436813843 |
| 701 | 2 | 73.466 | Marker185733 | 0.617432336 | -0.045238095 | -0.155764411 | 1.436813843 |
| 702 | 2 | 74.716 | Marker189047 | 0.642984127 | -0.049925484 | -0.131892697 | 1.145770797 |
| 703 | 2 | 74.716 | Marker187069 | 0.642984127 | -0.049925484 | -0.131892697 | 1.145770797 |
| 704 | 2 | 74.716 | Marker187201 | 0.642984127 | -0.049925484 | -0.131892697 | 1.145770797 |
| 705 | 2 | 74.716 | Marker187205 | 0.642984127 | -0.049925484 | -0.131892697 | 1.145770797 |
| 706 | 2 | 74.716 | Marker187742 | 0.642984127 | -0.049925484 | -0.131892697 | 1.145770797 |
| 707 | 2 | 74.716 | Marker187197 | 0.642984127 | -0.049925484 | -0.131892697 | 1.145770797 |
| 708 | 2 | 74.716 | Marker187068 | 0.642984127 | -0.049925484 | -0.131892697 | 1.145770797 |
| 709 | 2 | 74.716 | Marker187161 | 0.642984127 | -0.049925484 | -0.131892697 | 1.145770797 |
| 710 | 2 | 74.716 | Marker187200 | 0.642984127 | -0.049925484 | -0.131892697 | 1.145770797 |
| 711 | 2 | 74.716 | Marker187193 | 0.642984127 | -0.049925484 | -0.131892697 | 1.145770797 |
| 712 | 2 | 74.716 | Marker187199 | 0.642984127 | -0.049925484 | -0.131892697 | 1.145770797 |
| 713 | 2 | 74.716 | Marker187741 | 0.642984127 | -0.049925484 | -0.131892697 | 1.145770797 |
| 714 | 2 | 74.716 | Marker187743 | 0.642984127 | -0.049925484 | -0.131892697 | 1.145770797 |
| 715 | 2 | 74.716 | Marker187198 | 0.642984127 | -0.049925484 | -0.131892697 | 1.145770797 |
| 716 | 2 | 75.216 | Marker189443 | 0.603841298 | -0.049925484 | -0.131892697 | 1.145770797 |
| 717 | 2 | 75.216 | Marker189312 | 0.603841298 | -0.049925484 | -0.131892697 | 1.145770797 |
| 718 | 2 | 75.216 | Marker189573 | 0.603841298 | -0.049925484 | -0.131892697 | 1.145770797 |
| 719 | 2 | 75.216 | Marker189442 | 0.603841298 | -0.049925484 | -0.131892697 | 1.145770797 |
| 720 | 2 | 75.216 | Marker189130 | 0.603841298 | -0.049925484 | -0.131892697 | 1.145770797 |
| 721 | 2 | 75.216 | Marker189311 | 0.603841298 | -0.049925484 | -0.131892697 | 1.145770797 |
| 722 | 2 | 75.216 | Marker189313 | 0.603841298 | -0.049925484 | -0.131892697 | 1.145770797 |
| 723 | 2 | 75.216 | Marker189048 | 0.603841298 | -0.049925484 | -0.131892697 | 1.145770797 |
| 724 | 2 | 75.716 | Marker190359 | 0.603841298 | -0.049925484 | -0.131892697 | 1.145770797 |
| 725 | 2 | 76.717 | Marker190508 | 0.353591403 | -0.025534852 | -0.082172658 | 0.409196298 |
| 726 | 2 | 77.217 | Marker190716 | 0.114637961 | -0.032430506 | -0.064670943 | 0.326193425 |
| 727 | 2 | 77.467 | Marker191295 | 0.113980008 | -0.031884058 | -0.065583692 | 0.328189347 |
| 728 | 2 | 77.467 | Marker191296 | 0.113980008 | -0.031884058 | -0.065583692 | 0.328189347 |
| 729 | 2 | 77.717 | Marker191300 | 0.11342267 | -0.031319086 | -0.066507001 | 0.330271527 |
| 730 | 2 | 78.217 | Marker191953 | 0.204879378 | -0.031364637 | -0.106701968 | 0.676881152 |
| 731 | 2 | 78.967 | Marker191954 | 0.711712539 | -0.022988506 | -0.165656183 | 1.420958423 |
| 732 | 2 | 79.217 | Marker192138 | 0.690021299 | -0.021873161 | -0.163143274 | 1.374191784 |
| 733 | 2 | 80.217 | Marker192221 | 0.699236926 | -0.003039514 | -0.167173252 | 1.382738897 |
| 734 | 2 | 80.217 | Marker192220 | 0.699236926 | -0.003039514 | -0.167173252 | 1.382738897 |
| 735 | 2 | 80.468 | Marker192332 | 0.771977622 | -0.005911518 | -0.167538991 | 1.392498215 |
| 736 | 2 | 80.468 | Marker192939 | 0.771977622 | -0.005911518 | -0.167538991 | 1.392498215 |
| 737 | 2 | 80.468 | Marker192328 | 0.771977622 | -0.005911518 | -0.167538991 | 1.392498215 |
| 738 | 2 | 80.468 | Marker192333 | 0.771977622 | -0.005911518 | -0.167538991 | 1.392498215 |
| 739 | 2 | 80.468 | Marker192322 | 0.771977622 | -0.005911518 | -0.167538991 | 1.392498215 |
| 740 | 2 | 80.968 | Marker193460 | 0.766721632 | -0.003359684 | -0.169647563 | 1.424253834 |
| 741 | 2 | 80.968 | Marker193254 | 0.766721632 | -0.003359684 | -0.169647563 | 1.424253834 |
| 742 | 2 | 81.218 | Marker193465 | 0.831047154 | 0.004270186 | -0.188023047 | 1.747231848 |
| 743 | 2 | 81.218 | Marker193690 | 0.831047154 | 0.004270186 | -0.188023047 | 1.747231848 |
| 744 | 2 | 81.468 | Marker193691 | 0.82295482 | 0.002579365 | -0.189384921 | 1.771927635 |
| 745 | 2 | 81.718 | Marker193694 | 0.901285295 | 0.013131313 | -0.209923982 | 2.191230779 |
| 746 | 2 | 81.718 | Marker193724 | 0.901285295 | 0.013131313 | -0.209923982 | 2.191230779 |
| 747 | 2 | 81.718 | Marker193695 | 0.901285295 | 0.013131313 | -0.209923982 | 2.191230779 |
| 748 | 2 | 81.718 | Marker193693 | 0.901285295 | 0.013131313 | -0.209923982 | 2.191230779 |
| 749 | 2 | 81.718 | Marker193692 | 0.901285295 | 0.013131313 | -0.209923982 | 2.191230779 |
| 750 | 2 | 81.718 | Marker194076 | 0.901285295 | 0.013131313 | -0.209923982 | 2.191230779 |
| 751 | 2 | 82.218 | Marker194923 | 1.276221972 | 0.011257764 | -0.248328702 | 3.055661726 |
| 752 | 2 | 82.218 | Marker194915 | 1.276221972 | 0.011257764 | -0.248328702 | 3.055661726 |
| 753 | 2 | 82.218 | Marker194372 | 1.276221972 | 0.011257764 | -0.248328702 | 3.055661726 |
| 754 | 2 | 82.218 | Marker195440 | 1.276221972 | 0.011257764 | -0.248328702 | 3.055661726 |
| 755 | 2 | 82.218 | Marker194922 | 1.276221972 | 0.011257764 | -0.248328702 | 3.055661726 |
| 756 | 2 | 82.468 | Marker195148 | 1.191786757 | 0.023746201 | -0.22587386 | 2.572958182 |
| 757 | 2 | 82.468 | Marker195146 | 1.191786757 | 0.023746201 | -0.22587386 | 2.572958182 |
| 758 | 2 | 82.468 | Marker195093 | 1.191786757 | 0.023746201 | -0.22587386 | 2.572958182 |
| 759 | 2 | 83.218 | Marker196395 | 1.056794161 | 0.025531915 | -0.227099664 | 2.609278507 |
| 760 | 2 | 83.218 | Marker196941 | 1.056794161 | 0.025531915 | -0.227099664 | 2.609278507 |
| 761 | 2 | 83.468 | Marker196950 | 1.136256964 | 0.032674772 | -0.245440729 | 3.079168689 |
| 762 | 2 | 83.718 | Marker197129 | 1.045689579 | 0.025531915 | -0.227099664 | 2.609278507 |
| 763 | 2 | 83.718 | Marker197121 | 1.045689579 | 0.025531915 | -0.227099664 | 2.609278507 |
| 764 | 2 | 83.718 | Marker197024 | 1.045689579 | 0.025531915 | -0.227099664 | 2.609278507 |
| 765 | 2 | 83.718 | Marker197127 | 1.045689579 | 0.025531915 | -0.227099664 | 2.609278507 |
| 766 | 2 | 83.718 | Marker197128 | 1.045689579 | 0.025531915 | -0.227099664 | 2.609278507 |
| 767 | 2 | 83.718 | Marker197122 | 1.045689579 | 0.025531915 | -0.227099664 | 2.609278507 |
| 768 | 2 | 83.718 | Marker197335 | 1.045689579 | 0.025531915 | -0.227099664 | 2.609278507 |
| 769 | 2 | 83.968 | Marker197354 | 1.130114375 | 0.036643026 | -0.248079196 | 3.171809344 |
| 770 | 2 | 83.968 | Marker198465 | 1.130114375 | 0.036643026 | -0.248079196 | 3.171809344 |
| 771 | 2 | 84.218 | Marker198467 | 0.675793133 | 0.0293055 | -0.229788062 | 2.691021047 |
| 772 | 2 | 84.218 | Marker198466 | 0.675793133 | 0.0293055 | -0.229788062 | 2.691021047 |
| 773 | 2 | 84.218 | Marker198619 | 0.675793133 | 0.0293055 | -0.229788062 | 2.691021047 |
| 774 | 2 | 84.718 | Marker198748 | 0.612361291 | 0.042549095 | -0.207103338 | 2.302250306 |
| 775 | 2 | 84.968 | Marker199079 | 0.686508146 | 0.030856918 | -0.228773585 | 2.677710777 |
| 776 | 2 | 85.218 | Marker199244 | 0.572155629 | 0.019675926 | -0.207614522 | 2.16477983 |
| 777 | 2 | 85.218 | Marker199222 | 0.572155629 | 0.019675926 | -0.207614522 | 2.16477983 |
| 778 | 2 | 85.218 | Marker198749 | 0.572155629 | 0.019675926 | -0.207614522 | 2.16477983 |
| 779 | 2 | 85.218 | Marker199238 | 0.572155629 | 0.019675926 | -0.207614522 | 2.16477983 |
| 780 | 2 | 85.218 | Marker199581 | 0.572155629 | 0.019675926 | -0.207614522 | 2.16477983 |
| 781 | 2 | 85.218 | Marker199180 | 0.572155629 | 0.019675926 | -0.207614522 | 2.16477983 |
| 782 | 2 | 85.218 | Marker198754 | 0.572155629 | 0.019675926 | -0.207614522 | 2.16477983 |
| 783 | 2 | 85.718 | Marker200516 | 0.667494056 | 0.022592593 | -0.205603345 | 2.13625845 |
| 784 | 2 | 85.718 | Marker199649 | 0.667494056 | 0.022592593 | -0.205603345 | 2.13625845 |
| 785 | 2 | 85.718 | Marker199645 | 0.667494056 | 0.022592593 | -0.205603345 | 2.13625845 |
| 786 | 2 | 85.718 | Marker199588 | 0.667494056 | 0.022592593 | -0.205603345 | 2.13625845 |
| 787 | 2 | 85.718 | Marker199587 | 0.667494056 | 0.022592593 | -0.205603345 | 2.13625845 |
| 788 | 2 | 85.718 | Marker199646 | 0.667494056 | 0.022592593 | -0.205603345 | 2.13625845 |
| 789 | 2 | 85.718 | Marker199644 | 0.667494056 | 0.022592593 | -0.205603345 | 2.13625845 |
| 790 | 2 | 85.968 | Marker200517 | 0.668850512 | 0.020909091 | -0.204387352 | 2.104248744 |
| 791 | 2 | 85.968 | Marker200590 | 0.668850512 | 0.020909091 | -0.204387352 | 2.104248744 |
| 792 | 2 | 86.218 | Marker200652 | 0.451432675 | 0.013333333 | -0.185591398 | 1.716839517 |
| 793 | 2 | 86.968 | Marker200752 | 0.38378406 | 0.002727273 | -0.164466403 | 1.336436918 |
| 794 | 2 | 86.968 | Marker200817 | 0.38378406 | 0.002727273 | -0.164466403 | 1.336436918 |
| 795 | 2 | 86.968 | Marker201211 | 0.38378406 | 0.002727273 | -0.164466403 | 1.336436918 |
| 796 | 2 | 87.218 | Marker201611 | 0.397365635 | 0.004074074 | -0.16557945 | 1.355181367 |
| 797 | 2 | 87.218 | Marker201358 | 0.397365635 | 0.004074074 | -0.16557945 | 1.355181367 |
| 798 | 2 | 87.218 | Marker201375 | 0.397365635 | 0.004074074 | -0.16557945 | 1.355181367 |
| 799 | 2 | 87.968 | Marker201922 | 0.480324077 | 0.012849584 | -0.145936862 | 1.066999092 |
| 800 | 2 | 87.968 | Marker201877 | 0.480324077 | 0.012849584 | -0.145936862 | 1.066999092 |
| 801 | 2 | 88.218 | Marker202053 | 0.433770698 | 0.004813246 | -0.127262226 | 0.801619483 |
| 802 | 2 | 88.218 | Marker203419 | 0.433770698 | 0.004813246 | -0.127262226 | 0.801619483 |
| 803 | 2 | 88.718 | Marker203421 | 0.414496988 | 0.004813246 | -0.127262226 | 0.801619483 |
| 804 | 2 | 88.718 | Marker203637 | 0.414496988 | 0.004813246 | -0.127262226 | 0.801619483 |
| 805 | 2 | 88.718 | Marker203422 | 0.414496988 | 0.004813246 | -0.127262226 | 0.801619483 |
| 806 | 2 | 88.968 | Marker203719 | 0.42948253 | 0.003537736 | -0.128537736 | 0.816856847 |
| 807 | 2 | 89.218 | Marker203838 | 0.785472946 | -0.005390836 | -0.147992623 | 1.086776278 |
| 808 | 2 | 89.218 | Marker203834 | 0.785472946 | -0.005390836 | -0.147992623 | 1.086776278 |
| 809 | 2 | 89.218 | Marker204099 | 0.785472946 | -0.005390836 | -0.147992623 | 1.086776278 |
| 810 | 2 | 89.218 | Marker203839 | 0.785472946 | -0.005390836 | -0.147992623 | 1.086776278 |
| 811 | 2 | 89.218 | Marker205006 | 0.785472946 | -0.005390836 | -0.147992623 | 1.086776278 |
| 812 | 2 | 89.218 | Marker203835 | 0.785472946 | -0.005390836 | -0.147992623 | 1.086776278 |
| 813 | 2 | 90.469 | Marker205225 | 0.410365199 | -0.026077098 | -0.147826506 | 1.161562911 |
| 814 | 2 | 90.469 | Marker205224 | 0.410365199 | -0.026077098 | -0.147826506 | 1.161562911 |
| 815 | 2 | 90.469 | Marker205280 | 0.410365199 | -0.026077098 | -0.147826506 | 1.161562911 |
| 816 | 2 | 90.469 | Marker205007 | 0.410365199 | -0.026077098 | -0.147826506 | 1.161562911 |
| 817 | 2 | 90.719 | Marker207311 | 0.580907701 | -0.027777778 | -0.149415205 | 1.195512459 |
| 818 | 2 | 90.719 | Marker205711 | 0.580907701 | -0.027777778 | -0.149415205 | 1.195512459 |
| 819 | 2 | 90.719 | Marker205712 | 0.580907701 | -0.027777778 | -0.149415205 | 1.195512459 |
| 820 | 2 | 90.719 | Marker205710 | 0.580907701 | -0.027777778 | -0.149415205 | 1.195512459 |
| 821 | 2 | 91.969 | Marker207332 | 0.398040517 | -0.040660225 | -0.133252818 | 1.069110818 |
| 822 | 2 | 92.469 | Marker207333 | 0.407179395 | -0.038879599 | -0.135033445 | 1.076823522 |
| 823 | 2 | 92.469 | Marker207335 | 0.407179395 | -0.038879599 | -0.135033445 | 1.076823522 |
| 824 | 2 | 92.719 | Marker207838 | 0.596094502 | -0.040811966 | -0.136965812 | 1.120357062 |
| 825 | 2 | 92.719 | Marker207336 | 0.596094502 | -0.040811966 | -0.136965812 | 1.120357062 |
| 826 | 2 | 92.969 | Marker208322 | 0.449551356 | -0.042832168 | -0.138986014 | 1.167157069 |
| 827 | 2 | 92.969 | Marker207852 | 0.449551356 | -0.042832168 | -0.138986014 | 1.167157069 |
| 828 | 2 | 92.969 | Marker207850 | 0.449551356 | -0.042832168 | -0.138986014 | 1.167157069 |
| 829 | 2 | 92.969 | Marker207853 | 0.449551356 | -0.042832168 | -0.138986014 | 1.167157069 |
| 830 | 2 | 92.969 | Marker207851 | 0.449551356 | -0.042832168 | -0.138986014 | 1.167157069 |
| 831 | 2 | 92.969 | Marker207939 | 0.449551356 | -0.042832168 | -0.138986014 | 1.167157069 |
| 832 | 2 | 92.969 | Marker207938 | 0.449551356 | -0.042832168 | -0.138986014 | 1.167157069 |
| 833 | 2 | 92.969 | Marker207839 | 0.449551356 | -0.042832168 | -0.138986014 | 1.167157069 |
| 834 | 2 | 93.469 | Marker208324 | 0.42477146 | -0.054195804 | -0.16025064 | 1.607856242 |
| 835 | 2 | 93.469 | Marker208325 | 0.42477146 | -0.054195804 | -0.16025064 | 1.607856242 |
| 836 | 2 | 93.469 | Marker208323 | 0.42477146 | -0.054195804 | -0.16025064 | 1.607856242 |
| 837 | 2 | 93.719 | Marker208659 | 0.426414366 | -0.05657424 | -0.162532007 | 1.674159805 |
| 838 | 2 | 94.469 | Marker208917 | 0.39944446 | -0.063811189 | -0.140734266 | 1.443011348 |
| 839 | 2 | 94.719 | Marker209753 | 0.281196048 | -0.066189624 | -0.143112701 | 1.511286849 |
| 840 | 2 | 94.719 | Marker209616 | 0.281196048 | -0.066189624 | -0.143112701 | 1.511286849 |
| 841 | 2 | 94.719 | Marker209617 | 0.281196048 | -0.066189624 | -0.143112701 | 1.511286849 |
| 842 | 2 | 94.719 | Marker209569 | 0.281196048 | -0.066189624 | -0.143112701 | 1.511286849 |
| 843 | 2 | 94.719 | Marker209766 | 0.281196048 | -0.066189624 | -0.143112701 | 1.511286849 |
| 844 | 2 | 94.719 | Marker210262 | 0.281196048 | -0.066189624 | -0.143112701 | 1.511286849 |
| 845 | 2 | 94.719 | Marker210199 | 0.281196048 | -0.066189624 | -0.143112701 | 1.511286849 |
| 846 | 2 | 94.969 | Marker210866 | 0.279205964 | -0.065435477 | -0.143866849 | 1.510951243 |
| 847 | 2 | 94.969 | Marker210864 | 0.279205964 | -0.065435477 | -0.143866849 | 1.510951243 |
| 848 | 2 | 94.969 | Marker210865 | 0.279205964 | -0.065435477 | -0.143866849 | 1.510951243 |
| 849 | 2 | 94.969 | Marker210785 | 0.279205964 | -0.065435477 | -0.143866849 | 1.510951243 |
| 850 | 2 | 95.469 | Marker209558 | 0.300006683 | -0.055631555 | -0.163379508 | 1.675974912 |
| 851 | 3 | 0 | Marker211071 | 0.384378731 | 0.031960227 | -0.032470953 | 0.162578987 |
| 852 | 3 | 0 | Marker211511 | 0.384378731 | 0.031960227 | -0.032470953 | 0.162578987 |
| 853 | 3 | 0 | Marker214102 | 0.384378731 | 0.031960227 | -0.032470953 | 0.162578987 |
| 854 | 3 | 0 | Marker214103 | 0.384378731 | 0.031960227 | -0.032470953 | 0.162578987 |
| 855 | 3 | 0 | Marker214101 | 0.384378731 | 0.031960227 | -0.032470953 | 0.162578987 |
| 856 | 3 | 0.75 | Marker215529 | 0.312369444 | 0.045340502 | -0.012007168 | 0.232309405 |
| 857 | 3 | 0.75 | Marker215528 | 0.312369444 | 0.045340502 | -0.012007168 | 0.232309405 |
| 858 | 3 | 1.5 | Marker216172 | 0.482257997 | 0.049180328 | 0.027742749 | 0.306638692 |
| 859 | 3 | 2.25 | Marker216615 | 0.55880245 | 0.018410853 | -0.001092693 | 0.037315752 |
| 860 | 3 | 4.251 | Marker219000 | 0.153891331 | 0.018131868 | -0.056227106 | 0.190508202 |
| 861 | 3 | 4.251 | Marker218957 | 0.153891331 | 0.018131868 | -0.056227106 | 0.190508202 |
| 862 | 3 | 4.251 | Marker218997 | 0.153891331 | 0.018131868 | -0.056227106 | 0.190508202 |
| 863 | 3 | 4.501 | Marker219389 | 0.069103447 | 0.026785714 | -0.076236264 | 0.362358542 |
| 864 | 3 | 4.751 | Marker220243 | 0.069517816 | 0.027777778 | -0.077467219 | 0.377461248 |
| 865 | 3 | 6.802 | Marker222169 | 0.087262032 | 0.020177563 | -0.104998991 | 0.585627198 |
| 866 | 3 | 7.052 | Marker222237 | 0.09747991 | 0.010714286 | -0.084899749 | 0.367094988 |
| 867 | 3 | 8.803 | Marker223298 | 0.052620352 | 0.040556901 | -0.101568805 | 0.683136887 |
| 868 | 3 | 8.803 | Marker223644 | 0.052620352 | 0.040556901 | -0.101568805 | 0.683136887 |
| 869 | 3 | 8.803 | Marker223645 | 0.052620352 | 0.040556901 | -0.101568805 | 0.683136887 |
| 870 | 3 | 9.553 | Marker223103 | 0.081192607 | 0.022619048 | -0.062468672 | 0.246510322 |
| 871 | 3 | 10.553 | Marker227774 | 0.055058612 | 0.030952381 | -0.081328321 | 0.427599039 |
| 872 | 3 | 10.803 | Marker226664 | 0.093403183 | 0.017886179 | -0.105030488 | 0.57679716 |
| 873 | 3 | 11.053 | Marker225823 | 0.093369184 | 0.017796558 | -0.104931876 | 0.575442775 |
| 874 | 3 | 11.553 | Marker226356 | 0.196154773 | 0.008596561 | -0.085104905 | 0.364644561 |
| 875 | 3 | 11.553 | Marker227346 | 0.196154773 | 0.008596561 | -0.085104905 | 0.364644561 |
| 876 | 3 | 11.803 | Marker226675 | 0.184943564 | 0.015823821 | -0.071869444 | 0.280669751 |
| 877 | 3 | 12.553 | Marker228403 | 0.371561909 | -0.006048387 | -0.108425297 | 0.586064506 |
| 878 | 3 | 14.855 | Marker231232 | 0.115795895 | 0.041000841 | -0.103967491 | 0.711216855 |
| 879 | 3 | 18.01 | Marker234859 | 0.039644856 | 0.079991732 | 0.006254182 | 0.706854105 |
| 880 | 3 | 18.01 | Marker234861 | 0.039644856 | 0.079991732 | 0.006254182 | 0.706854105 |
| 881 | 3 | 18.862 | Marker237386 | 0.059634896 | 0.080593424 | 0.00516239 | 0.71671493 |
| 882 | 3 | 19.112 | Marker237283 | 0.005701539 | 0.090575275 | -0.01577307 | 0.912299005 |
| 883 | 3 | 23.375 | Marker238960 | 0.112412602 | 0.065199336 | 0.039689131 | 0.550269235 |
| 884 | 3 | 23.375 | Marker238923 | 0.112412602 | 0.065199336 | 0.039689131 | 0.550269235 |
| 885 | 3 | 23.375 | Marker238922 | 0.112412602 | 0.065199336 | 0.039689131 | 0.550269235 |
| 886 | 3 | 23.375 | Marker238919 | 0.112412602 | 0.065199336 | 0.039689131 | 0.550269235 |
| 887 | 3 | 24.925 | Marker239655 | 0.008209006 | 0.030315615 | -0.025806834 | 0.132589333 |
| 888 | 3 | 27.227 | Marker240752 | 0.043705516 | 0.061755335 | -0.000332615 | 0.419567568 |
| 889 | 3 | 27.477 | Marker240478 | 0.040730476 | 0.052631579 | 0.018796992 | 0.324042199 |
| 890 | 3 | 27.477 | Marker240479 | 0.040730476 | 0.052631579 | 0.018796992 | 0.324042199 |
| 891 | 3 | 27.477 | Marker240416 | 0.040730476 | 0.052631579 | 0.018796992 | 0.324042199 |
| 892 | 3 | 27.477 | Marker240477 | 0.040730476 | 0.052631579 | 0.018796992 | 0.324042199 |
| 893 | 3 | 30.029 | Marker242312 | 0.011638431 | 0.038372093 | 0.00220188 | 0.162394958 |
| 894 | 3 | 31.78 | Marker242736 | 0.209041166 | 0.06835225 | -0.021615919 | 0.534415936 |
| 895 | 3 | 32.381 | Marker243552 | 0.175058018 | 0.038676236 | -0.042464115 | 0.250665765 |
| 896 | 3 | 32.381 | Marker243548 | 0.175058018 | 0.038676236 | -0.042464115 | 0.250665765 |
| 897 | 3 | 34.933 | Marker245506 | 0.183955127 | 0.069838095 | 0.030389915 | 0.5861392 |
| 898 | 3 | 35.433 | Marker245501 | 0.22919766 | 0.075 | 0.019306931 | 0.639950096 |
| 899 | 3 | 35.433 | Marker245499 | 0.22919766 | 0.075 | 0.019306931 | 0.639950096 |
| 900 | 3 | 35.433 | Marker245500 | 0.22919766 | 0.075 | 0.019306931 | 0.639950096 |
| 901 | 3 | 37.285 | Marker247782 | 0.244524347 | 0.077586207 | -0.037586207 | 0.726763811 |
| 902 | 3 | 39.336 | Marker251249 | 0.258245487 | 0.084942085 | -0.00022275 | 0.793806089 |
| 903 | 3 | 40.337 | Marker250077 | 0.285537745 | 0.096846847 | -0.022650008 | 1.053279681 |
| 904 | 3 | 40.337 | Marker249155 | 0.285537745 | 0.096846847 | -0.022650008 | 1.053279681 |
| 905 | 3 | 40.337 | Marker248452 | 0.285537745 | 0.096846847 | -0.022650008 | 1.053279681 |
| 906 | 3 | 40.587 | Marker248813 | 0.291655912 | 0.097222222 | -0.022715472 | 1.061414295 |
| 907 | 3 | 41.337 | Marker248822 | 0.212603125 | 0.067601931 | 0.003590871 | 0.503891858 |
| 908 | 3 | 42.337 | Marker250923 | 0.237171971 | 0.072727273 | -0.02465035 | 0.608684978 |
| 909 | 3 | 42.337 | Marker251559 | 0.237171971 | 0.072727273 | -0.02465035 | 0.608684978 |
| 910 | 3 | 42.837 | Marker251352 | 0.213216458 | 0.0625 | -0.004807692 | 0.430372177 |
| 911 | 3 | 47.299 | Marker252316 | 0.216696662 | 0.034375 | 0.005141129 | 0.131637758 |
| 912 | 3 | 47.299 | Marker252323 | 0.216696662 | 0.034375 | 0.005141129 | 0.131637758 |
| 913 | 3 | 47.299 | Marker252322 | 0.216696662 | 0.034375 | 0.005141129 | 0.131637758 |
| 914 | 3 | 47.299 | Marker252324 | 0.216696662 | 0.034375 | 0.005141129 | 0.131637758 |
| 915 | 3 | 47.299 | Marker252321 | 0.216696662 | 0.034375 | 0.005141129 | 0.131637758 |
| 916 | 3 | 47.299 | Marker252317 | 0.216696662 | 0.034375 | 0.005141129 | 0.131637758 |
| 917 | 3 | 51.559 | Marker253049 | 0.448874525 | 0.089757412 | -0.006140112 | 0.887248284 |
| 918 | 3 | 52.309 | Marker253027 | 0.426020009 | 0.084131885 | 0.006966857 | 0.782237501 |
| 919 | 3 | 53.309 | Marker253734 | 0.513238493 | 0.105494505 | 0.015284715 | 1.238956999 |
| 920 | 3 | 55.911 | Marker253141 | 0.376427319 | 0.085227273 | -0.001403743 | 0.799056166 |
| 921 | 3 | 55.911 | Marker253147 | 0.376427319 | 0.085227273 | -0.001403743 | 0.799056166 |
| 922 | 3 | 55.911 | Marker253139 | 0.376427319 | 0.085227273 | -0.001403743 | 0.799056166 |
| 923 | 3 | 55.911 | Marker253148 | 0.376427319 | 0.085227273 | -0.001403743 | 0.799056166 |
| 924 | 3 | 55.911 | Marker253138 | 0.376427319 | 0.085227273 | -0.001403743 | 0.799056166 |
| 925 | 3 | 55.911 | Marker253140 | 0.376427319 | 0.085227273 | -0.001403743 | 0.799056166 |
| 926 | 3 | 58.213 | Marker253808 | 0.511710914 | 0.104043127 | 0.017319914 | 1.209122194 |
| 927 | 3 | 58.713 | Marker253678 | 0.426968841 | 0.090811966 | -0.007988316 | 0.909170165 |
| 928 | 3 | 59.713 | Marker254370 | 0.525067076 | 0.079551249 | 0.012896338 | 0.706368659 |
| 929 | 3 | 59.713 | Marker254304 | 0.525067076 | 0.079551249 | 0.012896338 | 0.706368659 |
| 930 | 3 | 59.713 | Marker254369 | 0.525067076 | 0.079551249 | 0.012896338 | 0.706368659 |
| 931 | 3 | 59.713 | Marker254368 | 0.525067076 | 0.079551249 | 0.012896338 | 0.706368659 |
| 932 | 3 | 59.713 | Marker254301 | 0.525067076 | 0.079551249 | 0.012896338 | 0.706368659 |
| 933 | 3 | 60.213 | Marker272085 | 0.563023051 | 0.093623482 | 0.033774452 | 1.02654323 |
| 934 | 3 | 60.213 | Marker268464 | 0.563023051 | 0.093623482 | 0.033774452 | 1.02654323 |
| 935 | 3 | 60.463 | Marker273108 | 0.470370306 | 0.080128205 | 0.011550556 | 0.714695682 |
| 936 | 3 | 61.213 | Marker267461 | 0.49369287 | 0.09042798 | 0.036022411 | 0.96976083 |
| 937 | 3 | 61.213 | Marker273735 | 0.49369287 | 0.09042798 | 0.036022411 | 0.96976083 |
| 938 | 3 | 61.213 | Marker273736 | 0.49369287 | 0.09042798 | 0.036022411 | 0.96976083 |
| 939 | 3 | 61.213 | Marker267460 | 0.49369287 | 0.09042798 | 0.036022411 | 0.96976083 |
| 940 | 3 | 61.713 | Marker264492 | 0.469426868 | 0.092525249 | 0.009193613 | 0.947636828 |
| 941 | 3 | 62.263 | Marker259985 | 0.255029157 | 0.062794678 | -0.044811896 | 0.527908932 |
| 942 | 3 | 62.263 | Marker259986 | 0.255029157 | 0.062794678 | -0.044811896 | 0.527908932 |
| 943 | 3 | 62.813 | Marker261877 | 0.424174826 | 0.085315018 | -0.035127825 | 0.856307397 |
| 944 | 3 | 63.063 | Marker261879 | 0.425439204 | 0.08729044 | -0.03128605 | 0.881702456 |
| 945 | 3 | 63.063 | Marker261881 | 0.425439204 | 0.08729044 | -0.03128605 | 0.881702456 |
| 946 | 3 | 63.063 | Marker261880 | 0.425439204 | 0.08729044 | -0.03128605 | 0.881702456 |
| 947 | 3 | 63.863 | Marker262625 | 0.698819193 | 0.119047619 | 0.021687883 | 1.587256884 |
| 948 | 3 | 64.113 | Marker262427 | 0.700469839 | 0.118986813 | 0.020612234 | 1.583179733 |
| 949 | 3 | 64.663 | Marker267235 | 0.729765058 | 0.125 | 0.007034632 | 1.723174196 |
| 950 | 3 | 65.664 | Marker267297 | 0.748335219 | 0.133825944 | 0.0335771 | 2.034382423 |
| 951 | 3 | 66.414 | Marker267084 | 0.702062355 | 0.120460048 | 0.013468523 | 1.608443775 |
| 952 | 3 | 66.414 | Marker267085 | 0.702062355 | 0.120460048 | 0.013468523 | 1.608443775 |
| 953 | 3 | 66.414 | Marker267083 | 0.702062355 | 0.120460048 | 0.013468523 | 1.608443775 |
| 954 | 3 | 66.414 | Marker267082 | 0.702062355 | 0.120460048 | 0.013468523 | 1.608443775 |
| 955 | 3 | 66.414 | Marker267086 | 0.702062355 | 0.120460048 | 0.013468523 | 1.608443775 |
| 956 | 3 | 67.964 | Marker273591 | 0.64057585 | 0.095650472 | 0.012694579 | 1.016792251 |
| 957 | 3 | 67.964 | Marker273592 | 0.64057585 | 0.095650472 | 0.012694579 | 1.016792251 |
| 958 | 3 | 68.714 | Marker277603 | 0.551004379 | 0.091130604 | 0.037691451 | 0.990199626 |
| 959 | 3 | 68.714 | Marker276090 | 0.551004379 | 0.091130604 | 0.037691451 | 0.990199626 |
| 960 | 3 | 68.964 | Marker274080 | 0.55173068 | 0.092353525 | 0.035749752 | 1.007598092 |
| 961 | 3 | 69.464 | Marker265025 | 0.522406783 | 0.080821206 | 0.011020636 | 0.726312822 |
| 962 | 3 | 69.464 | Marker266043 | 0.522406783 | 0.080821206 | 0.011020636 | 0.726312822 |
| 963 | 3 | 69.464 | Marker266044 | 0.522406783 | 0.080821206 | 0.011020636 | 0.726312822 |
| 964 | 3 | 69.464 | Marker271542 | 0.522406783 | 0.080821206 | 0.011020636 | 0.726312822 |
| 965 | 3 | 69.464 | Marker268168 | 0.522406783 | 0.080821206 | 0.011020636 | 0.726312822 |
| 966 | 3 | 69.464 | Marker274100 | 0.522406783 | 0.080821206 | 0.011020636 | 0.726312822 |
| 967 | 3 | 69.464 | Marker264615 | 0.522406783 | 0.080821206 | 0.011020636 | 0.726312822 |
| 968 | 3 | 69.464 | Marker275607 | 0.522406783 | 0.080821206 | 0.011020636 | 0.726312822 |
| 969 | 3 | 69.464 | Marker274099 | 0.522406783 | 0.080821206 | 0.011020636 | 0.726312822 |
| 970 | 3 | 69.464 | Marker266042 | 0.522406783 | 0.080821206 | 0.011020636 | 0.726312822 |
| 971 | 3 | 69.464 | Marker268754 | 0.522406783 | 0.080821206 | 0.011020636 | 0.726312822 |
| 972 | 3 | 69.464 | Marker274519 | 0.522406783 | 0.080821206 | 0.011020636 | 0.726312822 |
| 973 | 3 | 69.464 | Marker273055 | 0.522406783 | 0.080821206 | 0.011020636 | 0.726312822 |
| 974 | 3 | 69.464 | Marker273934 | 0.522406783 | 0.080821206 | 0.011020636 | 0.726312822 |
| 975 | 3 | 69.464 | Marker274101 | 0.522406783 | 0.080821206 | 0.011020636 | 0.726312822 |
| 976 | 3 | 69.464 | Marker265065 | 0.522406783 | 0.080821206 | 0.011020636 | 0.726312822 |
| 977 | 3 | 69.464 | Marker266510 | 0.522406783 | 0.080821206 | 0.011020636 | 0.726312822 |
| 978 | 3 | 69.464 | Marker264032 | 0.522406783 | 0.080821206 | 0.011020636 | 0.726312822 |
| 979 | 3 | 69.464 | Marker271541 | 0.522406783 | 0.080821206 | 0.011020636 | 0.726312822 |
| 980 | 3 | 69.464 | Marker271543 | 0.522406783 | 0.080821206 | 0.011020636 | 0.726312822 |
| 981 | 3 | 69.464 | Marker265066 | 0.522406783 | 0.080821206 | 0.011020636 | 0.726312822 |
| 982 | 3 | 69.964 | Marker271204 | 0.39232167 | 0.054487179 | -0.032958394 | 0.377027367 |
| 983 | 3 | 71.214 | Marker278230 | 0.376503279 | 0.054807692 | -0.032188645 | 0.378461769 |
| 984 | 3 | 71.464 | Marker278190 | 0.193238181 | 0.041666667 | -0.055212869 | 0.337421079 |
| 985 | 3 | 72.214 | Marker279042 | 0.176576623 | 0.0475 | -0.016425234 | 0.260142872 |
| 986 | 3 | 73.765 | Marker279534 | 0.239274824 | 0.072335601 | 0.027556524 | 0.616858202 |
| 987 | 3 | 74.265 | Marker280605 | 0.396389464 | 0.091681993 | 0.00787396 | 0.929198031 |
| 988 | 3 | 75.065 | Marker280725 | 0.315760957 | 0.1027063 | -0.031947562 | 1.20501759 |
| 989 | 3 | 75.315 | Marker280723 | 0.313061978 | 0.102609826 | -0.031841574 | 1.202529792 |
| 990 | 3 | 76.22 | Marker280703 | 0.163330649 | 0.071428571 | -0.012842522 | 0.567815551 |
| 991 | 3 | 77.322 | Marker281147 | 0.077571519 | 0.0625 | 0.004807692 | 0.431471886 |
| 992 | 3 | 78.572 | Marker281585 | 0.024433844 | 0.049019608 | 0.030188313 | 0.312113229 |
| 993 | 3 | 80.423 | Marker283107 | 0.015761111 | 0.045454545 | 0.04056696 | 0.312005165 |
| 994 | 3 | 80.423 | Marker282827 | 0.015761111 | 0.045454545 | 0.04056696 | 0.312005165 |
| 995 | 3 | 80.423 | Marker282836 | 0.015761111 | 0.045454545 | 0.04056696 | 0.312005165 |
| 996 | 3 | 80.423 | Marker282546 | 0.015761111 | 0.045454545 | 0.04056696 | 0.312005165 |
| 997 | 3 | 80.423 | Marker282829 | 0.015761111 | 0.045454545 | 0.04056696 | 0.312005165 |
| 998 | 3 | 80.423 | Marker282835 | 0.015761111 | 0.045454545 | 0.04056696 | 0.312005165 |
| 999 | 3 | 80.673 | Marker282273 | 0.046675908 | 0.055093515 | 0.062311724 | 0.532074339 |
| 1000 | 3 | 80.923 | Marker282763 | 0.041365773 | 0.05099129 | 0.070725203 | 0.53981335 |
| 1001 | 3 | 81.173 | Marker282654 | 0.048466443 | 0.058039377 | 0.055536489 | 0.528909106 |
| 1002 | 3 | 81.173 | Marker282714 | 0.048466443 | 0.058039377 | 0.055536489 | 0.528909106 |
| 1003 | 3 | 81.673 | Marker283404 | 0.06635978 | 0.068330606 | 0.034578643 | 0.577108122 |
| 1004 | 3 | 81.923 | Marker282522 | 0.058775118 | 0.057692308 | 0.014472641 | 0.378079816 |
| 1005 | 3 | 81.923 | Marker282521 | 0.058775118 | 0.057692308 | 0.014472641 | 0.378079816 |
| 1006 | 3 | 83.674 | Marker282717 | 0.087902197 | 0.047735849 | 0.07864713 | 0.563187364 |
| 1007 | 3 | 83.924 | Marker282719 | 0.092418825 | 0.058076923 | 0.056659919 | 0.535739583 |
| 1008 | 3 | 83.924 | Marker282718 | 0.092418825 | 0.058076923 | 0.056659919 | 0.535739583 |
| 1009 | 3 | 84.174 | Marker282725 | 0.200098943 | 0.066603774 | 0.038502609 | 0.566009169 |
| 1010 | 3 | 84.424 | Marker282720 | 0.189304976 | 0.056603774 | 0.017080437 | 0.368697952 |
| 1011 | 3 | 86.725 | Marker284456 | 0.209262538 | 0.058715221 | 0.054398109 | 0.53135398 |
| 1012 | 3 | 86.725 | Marker284454 | 0.209262538 | 0.058715221 | 0.054398109 | 0.53135398 |
| 1013 | 3 | 86.725 | Marker284455 | 0.209262538 | 0.058715221 | 0.054398109 | 0.53135398 |
| 1014 | 3 | 87.475 | Marker284099 | 0.182518711 | 0.060130719 | 0.051200414 | 0.532968293 |
| 1015 | 3 | 87.475 | Marker284093 | 0.182518711 | 0.060130719 | 0.051200414 | 0.532968293 |
| 1016 | 3 | 87.725 | Marker284098 | 0.182292363 | 0.059889173 | 0.051849957 | 0.533134366 |
| 1017 | 3 | 88.475 | Marker284085 | 0.132148522 | 0.050416667 | 0.071426768 | 0.538319852 |
| 1018 | 3 | 88.475 | Marker284090 | 0.132148522 | 0.050416667 | 0.071426768 | 0.538319852 |
| 1019 | 3 | 88.475 | Marker284092 | 0.132148522 | 0.050416667 | 0.071426768 | 0.538319852 |
| 1020 | 3 | 88.475 | Marker284083 | 0.132148522 | 0.050416667 | 0.071426768 | 0.538319852 |
| 1021 | 3 | 88.475 | Marker284091 | 0.132148522 | 0.050416667 | 0.071426768 | 0.538319852 |
| 1022 | 3 | 88.475 | Marker284084 | 0.132148522 | 0.050416667 | 0.071426768 | 0.538319852 |
| 1023 | 3 | 89.225 | Marker284998 | 0.132870378 | 0.051685892 | 0.068092454 | 0.529443964 |
| 1024 | 3 | 89.225 | Marker284999 | 0.132870378 | 0.051685892 | 0.068092454 | 0.529443964 |
| 1025 | 3 | 91.026 | Marker286195 | 0.241614695 | 0.07124183 | 0.072212515 | 0.825472005 |
| 1026 | 3 | 91.026 | Marker286197 | 0.241614695 | 0.07124183 | 0.072212515 | 0.825472005 |
| 1027 | 3 | 91.276 | Marker286129 | 0.185955333 | 0.062222222 | 0.090065359 | 0.83700728 |
| 1028 | 3 | 93.578 | Marker287073 | 0.334092061 | 0.059187621 | 0.141484272 | 1.389790039 |
| 1029 | 3 | 93.578 | Marker286744 | 0.334092061 | 0.059187621 | 0.141484272 | 1.389790039 |
| 1030 | 3 | 94.578 | Marker287629 | 0.379840195 | 0.058914132 | 0.139152463 | 1.353582154 |
| 1031 | 3 | 95.578 | Marker287788 | 0.361891505 | 0.059334845 | 0.181872276 | 2.041384592 |
| 1032 | 3 | 95.578 | Marker287792 | 0.361891505 | 0.059334845 | 0.181872276 | 2.041384592 |
| 1033 | 3 | 95.578 | Marker287793 | 0.361891505 | 0.059334845 | 0.181872276 | 2.041384592 |
| 1034 | 3 | 95.578 | Marker287790 | 0.361891505 | 0.059334845 | 0.181872276 | 2.041384592 |
| 1035 | 3 | 95.578 | Marker287789 | 0.361891505 | 0.059334845 | 0.181872276 | 2.041384592 |
| 1036 | 3 | 96.328 | Marker288430 | 0.528130121 | 0.078869048 | 0.186587942 | 2.431445291 |
| 1037 | 3 | 96.328 | Marker288437 | 0.528130121 | 0.078869048 | 0.186587942 | 2.431445291 |
| 1038 | 3 | 96.578 | Marker288413 | 0.535552065 | 0.079977204 | 0.185980243 | 2.439915439 |
| 1039 | 3 | 96.578 | Marker288631 | 0.535552065 | 0.079977204 | 0.185980243 | 2.439915439 |
| 1040 | 3 | 96.578 | Marker288630 | 0.535552065 | 0.079977204 | 0.185980243 | 2.439915439 |
| 1041 | 3 | 96.578 | Marker288634 | 0.535552065 | 0.079977204 | 0.185980243 | 2.439915439 |
| 1042 | 3 | 96.578 | Marker288412 | 0.535552065 | 0.079977204 | 0.185980243 | 2.439915439 |
| 1043 | 3 | 96.578 | Marker288361 | 0.535552065 | 0.079977204 | 0.185980243 | 2.439915439 |
| 1044 | 3 | 97.679 | Marker289104 | 0.476590608 | 0.059210526 | 0.187166285 | 2.136822915 |
| 1045 | 3 | 97.679 | Marker288953 | 0.476590608 | 0.059210526 | 0.187166285 | 2.136822915 |
| 1046 | 3 | 97.679 | Marker288906 | 0.476590608 | 0.059210526 | 0.187166285 | 2.136822915 |
| 1047 | 3 | 97.679 | Marker288954 | 0.476590608 | 0.059210526 | 0.187166285 | 2.136822915 |
| 1048 | 3 | 97.679 | Marker289123 | 0.476590608 | 0.059210526 | 0.187166285 | 2.136822915 |
| 1049 | 3 | 97.929 | Marker288917 | 0.532596636 | 0.068452381 | 0.165418587 | 1.888220672 |
| 1050 | 3 | 99.031 | Marker288633 | 0.556153217 | 0.081105408 | 0.145349503 | 1.789128837 |
| 1051 | 3 | 99.281 | Marker288635 | 0.55246095 | 0.079841897 | 0.142531291 | 1.725921829 |
| 1052 | 3 | 99.781 | Marker287591 | 0.510463243 | 0.068951931 | 0.119877857 | 1.248242549 |
| 1053 | 3 | 101.081 | Marker286887 | 0.350360487 | 0.05815595 | 0.143761862 | 1.408544765 |
| 1054 | 3 | 101.581 | Marker287126 | 0.349500694 | 0.057489043 | 0.146577959 | 1.440577051 |
| 1055 | 3 | 101.831 | Marker287127 | 0.347556816 | 0.057412752 | 0.146915343 | 1.44451993 |
| 1056 | 3 | 102.581 | Marker286561 | 0.524387691 | 0.078135583 | 0.1159073 | 1.352054054 |
| 1057 | 3 | 103.081 | Marker286562 | 0.541277483 | 0.084879752 | 0.12591799 | 1.595607836 |
| 1058 | 3 | 103.831 | Marker286897 | 0.511500853 | 0.084675976 | 0.128824809 | 1.628795819 |
| 1059 | 3 | 103.831 | Marker286896 | 0.511500853 | 0.084675976 | 0.128824809 | 1.628795819 |
| 1060 | 3 | 104.331 | Marker286899 | 0.448750938 | 0.073103448 | 0.110492057 | 1.205953978 |
| 1061 | 3 | 104.331 | Marker286898 | 0.448750938 | 0.073103448 | 0.110492057 | 1.205953978 |
| 1062 | 3 | 104.581 | Marker286900 | 0.402769981 | 0.063898305 | 0.132465331 | 1.331675495 |
| 1063 | 4 | 0 | Marker290064 | 1.117029228 | 0.024564797 | -0.251359055 | 3.176740606 |
| 1064 | 4 | 0.25 | Marker289674 | 1.199087944 | 0.032765152 | -0.2708696 | 3.726937532 |
| 1065 | 4 | 0.25 | Marker289673 | 1.199087944 | 0.032765152 | -0.2708696 | 3.726937532 |
| 1066 | 4 | 0.25 | Marker290821 | 1.199087944 | 0.032765152 | -0.2708696 | 3.726937532 |
| 1067 | 4 | 0.25 | Marker290820 | 1.199087944 | 0.032765152 | -0.2708696 | 3.726937532 |
| 1068 | 4 | 0.25 | Marker291464 | 1.199087944 | 0.032765152 | -0.2708696 | 3.726937532 |
| 1069 | 4 | 1.5 | Marker290822 | 0.751781671 | -0.005734369 | -0.287834306 | 4.099998614 |
| 1070 | 4 | 1.5 | Marker291683 | 0.751781671 | -0.005734369 | -0.287834306 | 4.099998614 |
| 1071 | 4 | 1.75 | Marker291798 | 0.512090648 | -0.013773585 | -0.26824167 | 3.582697124 |
| 1072 | 4 | 2 | Marker292916 | 0.514429117 | -0.011851852 | -0.267120669 | 3.546659731 |
| 1073 | 4 | 2.351 | Marker292960 | 0.467009446 | -0.016666667 | -0.247163121 | 3.056398901 |
| 1074 | 4 | 2.351 | Marker293002 | 0.467009446 | -0.016666667 | -0.247163121 | 3.056398901 |
| 1075 | 4 | 2.351 | Marker293000 | 0.467009446 | -0.016666667 | -0.247163121 | 3.056398901 |
| 1076 | 4 | 2.702 | Marker293033 | 0.442025352 | -0.021851852 | -0.246367981 | 3.06129797 |
| 1077 | 4 | 2.702 | Marker293030 | 0.442025352 | -0.021851852 | -0.246367981 | 3.06129797 |
| 1078 | 4 | 2.702 | Marker293034 | 0.442025352 | -0.021851852 | -0.246367981 | 3.06129797 |
| 1079 | 4 | 2.702 | Marker293032 | 0.442025352 | -0.021851852 | -0.246367981 | 3.06129797 |
| 1080 | 4 | 2.954 | Marker293028 | 0.442025352 | -0.021851852 | -0.246367981 | 3.06129797 |
| 1081 | 4 | 3.609 | Marker294045 | 0.437789382 | -0.009615385 | -0.247208437 | 3.033927546 |
| 1082 | 4 | 3.609 | Marker294809 | 0.437789382 | -0.009615385 | -0.247208437 | 3.033927546 |
| 1083 | 4 | 5.36 | Marker295222 | 0.231015385 | -0.007686932 | -0.24416492 | 2.95545182 |
| 1084 | 4 | 6.61 | Marker295144 | 0.310017041 | -0.028846154 | -0.247208437 | 3.124005448 |
| 1085 | 4 | 6.61 | Marker295143 | 0.310017041 | -0.028846154 | -0.247208437 | 3.124005448 |
| 1086 | 4 | 6.61 | Marker295346 | 0.310017041 | -0.028846154 | -0.247208437 | 3.124005448 |
| 1087 | 4 | 6.86 | Marker295949 | 0.336604769 | -0.017533937 | -0.26847261 | 3.603627158 |
| 1088 | 4 | 6.86 | Marker295223 | 0.336604769 | -0.017533937 | -0.26847261 | 3.603627158 |
| 1089 | 4 | 6.86 | Marker295293 | 0.336604769 | -0.017533937 | -0.26847261 | 3.603627158 |
| 1090 | 4 | 7.11 | Marker295951 | 0.33635386 | -0.019607843 | -0.269762642 | 3.647473079 |
| 1091 | 4 | 7.11 | Marker295802 | 0.33635386 | -0.019607843 | -0.269762642 | 3.647473079 |
| 1092 | 4 | 7.11 | Marker295806 | 0.33635386 | -0.019607843 | -0.269762642 | 3.647473079 |
| 1093 | 4 | 7.11 | Marker295350 | 0.33635386 | -0.019607843 | -0.269762642 | 3.647473079 |
| 1094 | 4 | 7.11 | Marker295349 | 0.33635386 | -0.019607843 | -0.269762642 | 3.647473079 |
| 1095 | 4 | 7.36 | Marker295950 | 0.333870576 | -0.017843137 | -0.270759804 | 3.665993236 |
| 1096 | 4 | 7.36 | Marker296525 | 0.333870576 | -0.017843137 | -0.270759804 | 3.665993236 |
| 1097 | 4 | 7.865 | Marker296593 | 0.350357554 | -0.029874214 | -0.269457547 | 3.700288255 |
| 1098 | 4 | 8.365 | Marker296592 | 0.316541175 | -0.016745469 | -0.249458235 | 3.113109621 |
| 1099 | 4 | 8.365 | Marker296539 | 0.316541175 | -0.016745469 | -0.249458235 | 3.113109621 |
| 1100 | 4 | 8.365 | Marker297387 | 0.316541175 | -0.016745469 | -0.249458235 | 3.113109621 |
| 1101 | 4 | 9.115 | Marker298234 | 0.307626501 | -0.025995807 | -0.232056413 | 2.74600588 |
| 1102 | 4 | 9.365 | Marker298365 | 0.309761632 | -0.027686628 | -0.23088095 | 2.729764881 |
| 1103 | 4 | 9.365 | Marker297559 | 0.309761632 | -0.027686628 | -0.23088095 | 2.729764881 |
| 1104 | 4 | 10.115 | Marker298366 | 0.241881249 | -0.023571131 | -0.212911341 | 2.310025964 |
| 1105 | 4 | 10.365 | Marker299026 | 0.242005571 | -0.021685761 | -0.211438258 | 2.268960186 |
| 1106 | 4 | 10.365 | Marker298535 | 0.242005571 | -0.021685761 | -0.211438258 | 2.268960186 |
| 1107 | 4 | 10.865 | Marker300063 | 0.362934612 | -0.031756181 | -0.233930094 | 2.828301154 |
| 1108 | 4 | 10.865 | Marker299726 | 0.362934612 | -0.031756181 | -0.233930094 | 2.828301154 |
| 1109 | 4 | 10.865 | Marker299238 | 0.362934612 | -0.031756181 | -0.233930094 | 2.828301154 |
| 1110 | 4 | 10.865 | Marker299025 | 0.362934612 | -0.031756181 | -0.233930094 | 2.828301154 |
| 1111 | 4 | 10.865 | Marker299227 | 0.362934612 | -0.031756181 | -0.233930094 | 2.828301154 |
| 1112 | 4 | 11.365 | Marker301192 | 0.276660629 | -0.042625746 | -0.213060529 | 2.459380348 |
| 1113 | 4 | 11.365 | Marker299728 | 0.276660629 | -0.042625746 | -0.213060529 | 2.459380348 |
| 1114 | 4 | 11.365 | Marker299727 | 0.276660629 | -0.042625746 | -0.213060529 | 2.459380348 |
| 1115 | 4 | 11.615 | Marker301105 | 0.34830494 | -0.050167224 | -0.231107057 | 2.937008172 |
| 1116 | 4 | 11.615 | Marker300225 | 0.34830494 | -0.050167224 | -0.231107057 | 2.937008172 |
| 1117 | 4 | 11.615 | Marker301160 | 0.34830494 | -0.050167224 | -0.231107057 | 2.937008172 |
| 1118 | 4 | 11.615 | Marker300520 | 0.34830494 | -0.050167224 | -0.231107057 | 2.937008172 |
| 1119 | 4 | 11.615 | Marker301197 | 0.34830494 | -0.050167224 | -0.231107057 | 2.937008172 |
| 1120 | 4 | 11.615 | Marker300282 | 0.34830494 | -0.050167224 | -0.231107057 | 2.937008172 |
| 1121 | 4 | 11.615 | Marker300064 | 0.34830494 | -0.050167224 | -0.231107057 | 2.937008172 |
| 1122 | 4 | 11.865 | Marker301972 | 0.348766873 | -0.052429668 | -0.23286445 | 3.003958327 |
| 1123 | 4 | 11.865 | Marker301193 | 0.348766873 | -0.052429668 | -0.23286445 | 3.003958327 |
| 1124 | 4 | 12.115 | Marker301507 | 0.30086463 | -0.050980392 | -0.233818676 | 3.008942869 |
| 1125 | 4 | 12.115 | Marker302201 | 0.30086463 | -0.050980392 | -0.233818676 | 3.008942869 |
| 1126 | 4 | 12.115 | Marker301768 | 0.30086463 | -0.050980392 | -0.233818676 | 3.008942869 |
| 1127 | 4 | 12.115 | Marker301701 | 0.30086463 | -0.050980392 | -0.233818676 | 3.008942869 |
| 1128 | 4 | 12.115 | Marker301514 | 0.30086463 | -0.050980392 | -0.233818676 | 3.008942869 |
| 1129 | 4 | 12.115 | Marker301509 | 0.30086463 | -0.050980392 | -0.233818676 | 3.008942869 |
| 1130 | 4 | 12.115 | Marker301508 | 0.30086463 | -0.050980392 | -0.233818676 | 3.008942869 |
| 1131 | 4 | 12.115 | Marker301198 | 0.30086463 | -0.050980392 | -0.233818676 | 3.008942869 |
| 1132 | 4 | 12.115 | Marker301517 | 0.30086463 | -0.050980392 | -0.233818676 | 3.008942869 |
| 1133 | 4 | 12.115 | Marker301199 | 0.30086463 | -0.050980392 | -0.233818676 | 3.008942869 |
| 1134 | 4 | 12.115 | Marker301515 | 0.30086463 | -0.050980392 | -0.233818676 | 3.008942869 |
| 1135 | 4 | 12.115 | Marker301203 | 0.30086463 | -0.050980392 | -0.233818676 | 3.008942869 |
| 1136 | 4 | 12.115 | Marker301204 | 0.30086463 | -0.050980392 | -0.233818676 | 3.008942869 |
| 1137 | 4 | 12.115 | Marker301766 | 0.30086463 | -0.050980392 | -0.233818676 | 3.008942869 |
| 1138 | 4 | 12.865 | Marker302882 | 0.293757507 | -0.041560102 | -0.253734015 | 3.390250774 |
| 1139 | 4 | 13.365 | Marker303690 | 0.26600867 | -0.024893617 | -0.255106383 | 3.295220326 |
| 1140 | 4 | 13.865 | Marker302947 | 0.257848547 | -0.028911565 | -0.255578231 | 3.332812152 |
| 1141 | 4 | 13.865 | Marker302984 | 0.257848547 | -0.028911565 | -0.255578231 | 3.332812152 |
| 1142 | 4 | 13.865 | Marker303673 | 0.257848547 | -0.028911565 | -0.255578231 | 3.332812152 |
| 1143 | 4 | 13.865 | Marker304518 | 0.257848547 | -0.028911565 | -0.255578231 | 3.332812152 |
| 1144 | 4 | 13.865 | Marker302980 | 0.257848547 | -0.028911565 | -0.255578231 | 3.332812152 |
| 1145 | 4 | 13.865 | Marker302979 | 0.257848547 | -0.028911565 | -0.255578231 | 3.332812152 |
| 1146 | 4 | 14.115 | Marker304163 | 0.256472081 | -0.027138515 | -0.256757222 | 3.350951532 |
| 1147 | 4 | 14.115 | Marker303691 | 0.256472081 | -0.027138515 | -0.256757222 | 3.350951532 |
| 1148 | 4 | 14.115 | Marker305189 | 0.256472081 | -0.027138515 | -0.256757222 | 3.350951532 |
| 1149 | 4 | 14.115 | Marker304497 | 0.256472081 | -0.027138515 | -0.256757222 | 3.350951532 |
| 1150 | 4 | 14.115 | Marker304164 | 0.256472081 | -0.027138515 | -0.256757222 | 3.350951532 |
| 1151 | 4 | 14.365 | Marker305190 | 0.333549217 | -0.034893617 | -0.275106383 | 3.890877869 |
| 1152 | 4 | 14.865 | Marker305264 | 0.343423623 | -0.034893617 | -0.275106383 | 3.890877869 |
| 1153 | 4 | 14.865 | Marker305191 | 0.343423623 | -0.034893617 | -0.275106383 | 3.890877869 |
| 1154 | 4 | 15.115 | Marker305643 | 0.338046042 | -0.036666667 | -0.274040404 | 3.876736494 |
| 1155 | 4 | 15.615 | Marker305644 | 0.227928899 | -0.043178974 | -0.252721205 | 3.380672279 |
| 1156 | 4 | 15.865 | Marker305645 | 0.226043983 | -0.052429668 | -0.23286445 | 3.003958327 |
| 1157 | 4 | 16.615 | Marker305715 | 0.240408462 | -0.061036789 | -0.210136482 | 2.615076526 |
| 1158 | 4 | 16.865 | Marker305717 | 0.096105822 | -0.049425759 | -0.188733656 | 2.045770115 |
| 1159 | 4 | 17.115 | Marker305719 | 0.090782078 | -0.047504026 | -0.187127513 | 1.994645543 |
| 1160 | 4 | 17.365 | Marker305718 | 0.090773436 | -0.04816282 | -0.187678119 | 2.012050169 |
| 1161 | 4 | 17.865 | Marker305720 | 0.088196685 | -0.045652174 | -0.185597826 | 1.946733312 |
| 1162 | 4 | 18.365 | Marker305721 | 0.083221027 | -0.045022796 | -0.183320669 | 1.898546315 |
| 1163 | 4 | 18.865 | Marker307220 | 0.154852225 | -0.036561265 | -0.16609025 | 1.521143757 |
| 1164 | 4 | 19.117 | Marker307682 | 0.180284032 | -0.05929866 | -0.165238377 | 1.753824766 |
| 1165 | 4 | 20.117 | Marker307766 | 0.109580242 | -0.048660362 | -0.186293341 | 1.99181132 |
| 1166 | 4 | 20.117 | Marker307393 | 0.109580242 | -0.048660362 | -0.186293341 | 1.99181132 |
| 1167 | 4 | 20.468 | Marker307875 | 0.091202012 | -0.024242424 | -0.188503593 | 1.828657306 |
| 1168 | 4 | 20.718 | Marker307767 | 0.101671063 | -0.0256917 | -0.187376482 | 1.816134469 |
| 1169 | 4 | 20.968 | Marker307876 | 0.100118568 | -0.036634461 | -0.208306357 | 2.305502674 |
| 1170 | 4 | 21.468 | Marker308161 | 0.127470487 | -0.046808511 | -0.184770437 | 1.943677982 |
| 1171 | 4 | 22.218 | Marker309197 | 0.44456459 | -0.066299392 | -0.140767477 | 1.479737327 |
| 1172 | 4 | 22.718 | Marker310299 | 0.289534849 | -0.066299392 | -0.140767477 | 1.479737327 |
| 1173 | 4 | 23.468 | Marker309200 | 0.269979973 | -0.07946027 | -0.176935726 | 2.267180477 |
| 1174 | 4 | 23.468 | Marker310305 | 0.269979973 | -0.07946027 | -0.176935726 | 2.267180477 |
| 1175 | 4 | 25.219 | Marker310308 | 0.075108549 | -0.077185245 | -0.178202686 | 2.249644237 |
| 1176 | 4 | 25.219 | Marker310306 | 0.075108549 | -0.077185245 | -0.178202686 | 2.249644237 |
| 1177 | 4 | 25.469 | Marker310849 | 0.062189254 | -0.075285771 | -0.17663216 | 2.189410162 |
| 1178 | 4 | 25.469 | Marker310309 | 0.062189254 | -0.075285771 | -0.17663216 | 2.189410162 |
| 1179 | 4 | 25.469 | Marker310307 | 0.062189254 | -0.075285771 | -0.17663216 | 2.189410162 |
| 1180 | 4 | 25.719 | Marker313056 | 0.046562414 | -0.065116279 | -0.156160317 | 1.689981367 |
| 1181 | 4 | 26.969 | Marker313273 | 0.025277143 | -0.04576144 | -0.115528882 | 0.899517079 |
| 1182 | 4 | 28.72 | Marker313746 | 0.048694342 | -0.046252927 | -0.117681499 | 0.929582174 |
| 1183 | 4 | 28.72 | Marker313278 | 0.048694342 | -0.046252927 | -0.117681499 | 0.929582174 |
| 1184 | 4 | 28.97 | Marker314833 | 0.184656432 | -0.055952381 | -0.137907268 | 1.298224431 |
| 1185 | 4 | 30.22 | Marker315741 | 0.167031122 | -0.055720339 | -0.140924421 | 1.337188114 |
| 1186 | 4 | 30.22 | Marker313747 | 0.167031122 | -0.055720339 | -0.140924421 | 1.337188114 |
| 1187 | 4 | 30.47 | Marker314839 | 0.19889577 | -0.065948276 | -0.161150296 | 1.781049754 |
| 1188 | 4 | 30.47 | Marker316176 | 0.19889577 | -0.065948276 | -0.161150296 | 1.781049754 |
| 1189 | 4 | 30.47 | Marker314834 | 0.19889577 | -0.065948276 | -0.161150296 | 1.781049754 |
| 1190 | 4 | 30.47 | Marker314837 | 0.19889577 | -0.065948276 | -0.161150296 | 1.781049754 |
| 1191 | 4 | 30.47 | Marker314838 | 0.19889577 | -0.065948276 | -0.161150296 | 1.781049754 |
| 1192 | 4 | 31.47 | Marker316258 | 0.378996349 | -0.069642857 | -0.164444837 | 1.890674755 |
| 1193 | 4 | 31.47 | Marker315848 | 0.378996349 | -0.069642857 | -0.164444837 | 1.890674755 |
| 1194 | 4 | 31.72 | Marker316419 | 0.402100869 | -0.071590909 | -0.166198752 | 1.950414195 |
| 1195 | 4 | 31.72 | Marker316177 | 0.402100869 | -0.071590909 | -0.166198752 | 1.950414195 |
| 1196 | 4 | 31.97 | Marker316309 | 0.402639906 | -0.072505543 | -0.165478255 | 1.953290319 |
| 1197 | 4 | 31.97 | Marker316418 | 0.402639906 | -0.072505543 | -0.165478255 | 1.953290319 |
| 1198 | 4 | 31.97 | Marker316417 | 0.402639906 | -0.072505543 | -0.165478255 | 1.953290319 |
| 1199 | 4 | 31.97 | Marker316412 | 0.402639906 | -0.072505543 | -0.165478255 | 1.953290319 |
| 1200 | 4 | 31.97 | Marker316420 | 0.402639906 | -0.072505543 | -0.165478255 | 1.953290319 |
| 1201 | 4 | 32.72 | Marker316873 | 0.367285506 | -0.065266486 | -0.148241139 | 1.572130517 |
| 1202 | 4 | 32.72 | Marker317750 | 0.367285506 | -0.065266486 | -0.148241139 | 1.572130517 |
| 1203 | 4 | 32.72 | Marker316759 | 0.367285506 | -0.065266486 | -0.148241139 | 1.572130517 |
| 1204 | 4 | 33.22 | Marker318269 | 0.364848794 | -0.075 | -0.125 | 1.408034421 |
| 1205 | 4 | 33.22 | Marker316880 | 0.364848794 | -0.075 | -0.125 | 1.408034421 |
| 1206 | 4 | 33.22 | Marker316882 | 0.364848794 | -0.075 | -0.125 | 1.408034421 |
| 1207 | 4 | 33.22 | Marker316881 | 0.364848794 | -0.075 | -0.125 | 1.408034421 |
| 1208 | 4 | 33.22 | Marker316876 | 0.364848794 | -0.075 | -0.125 | 1.408034421 |
| 1209 | 4 | 33.22 | Marker316877 | 0.364848794 | -0.075 | -0.125 | 1.408034421 |
| 1210 | 4 | 33.22 | Marker316879 | 0.364848794 | -0.075 | -0.125 | 1.408034421 |
| 1211 | 4 | 33.47 | Marker319242 | 0.364694897 | -0.075609756 | -0.124390244 | 1.410680284 |
| 1212 | 4 | 33.47 | Marker317836 | 0.364694897 | -0.075609756 | -0.124390244 | 1.410680284 |
| 1213 | 4 | 33.72 | Marker319332 | 0.363105965 | -0.073824042 | -0.12260453 | 1.358881315 |
| 1214 | 4 | 33.72 | Marker318271 | 0.363105965 | -0.073824042 | -0.12260453 | 1.358881315 |
| 1215 | 4 | 33.97 | Marker319347 | 0.363105812 | -0.074404762 | -0.12202381 | 1.361384422 |
| 1216 | 4 | 33.97 | Marker319355 | 0.363105812 | -0.074404762 | -0.12202381 | 1.361384422 |
| 1217 | 4 | 33.97 | Marker320199 | 0.363105812 | -0.074404762 | -0.12202381 | 1.361384422 |
| 1218 | 4 | 33.97 | Marker319350 | 0.363105812 | -0.074404762 | -0.12202381 | 1.361384422 |
| 1219 | 4 | 34.22 | Marker322327 | 0.277423051 | -0.076190476 | -0.123809524 | 1.413309109 |
| 1220 | 4 | 37.073 | Marker322499 | 0.166567498 | -0.080687831 | -0.084695374 | 1.083234272 |
| 1221 | 4 | 37.323 | Marker324811 | 0.223697226 | -0.092592593 | -0.062592593 | 1.14745246 |
| 1222 | 4 | 37.323 | Marker323352 | 0.223697226 | -0.092592593 | -0.062592593 | 1.14745246 |
| 1223 | 4 | 37.323 | Marker324030 | 0.223697226 | -0.092592593 | -0.062592593 | 1.14745246 |
| 1224 | 4 | 37.323 | Marker323207 | 0.223697226 | -0.092592593 | -0.062592593 | 1.14745246 |
| 1225 | 4 | 37.323 | Marker323205 | 0.223697226 | -0.092592593 | -0.062592593 | 1.14745246 |
| 1226 | 4 | 37.573 | Marker324440 | 0.159021579 | -0.081228956 | -0.083754209 | 1.084985172 |
| 1227 | 4 | 37.573 | Marker324298 | 0.159021579 | -0.081228956 | -0.083754209 | 1.084985172 |
| 1228 | 4 | 37.573 | Marker324510 | 0.159021579 | -0.081228956 | -0.083754209 | 1.084985172 |
| 1229 | 4 | 37.573 | Marker324447 | 0.159021579 | -0.081228956 | -0.083754209 | 1.084985172 |
| 1230 | 4 | 37.573 | Marker324559 | 0.159021579 | -0.081228956 | -0.083754209 | 1.084985172 |
| 1231 | 4 | 37.573 | Marker324441 | 0.159021579 | -0.081228956 | -0.083754209 | 1.084985172 |
| 1232 | 4 | 37.573 | Marker324446 | 0.159021579 | -0.081228956 | -0.083754209 | 1.084985172 |
| 1233 | 4 | 37.573 | Marker324444 | 0.159021579 | -0.081228956 | -0.083754209 | 1.084985172 |
| 1234 | 4 | 37.573 | Marker324445 | 0.159021579 | -0.081228956 | -0.083754209 | 1.084985172 |
| 1235 | 4 | 37.573 | Marker325125 | 0.159021579 | -0.081228956 | -0.083754209 | 1.084985172 |
| 1236 | 4 | 37.823 | Marker324032 | 0.166066262 | -0.069336779 | -0.105848407 | 1.095937239 |
| 1237 | 4 | 37.823 | Marker325674 | 0.166066262 | -0.069336779 | -0.105848407 | 1.095937239 |
| 1238 | 4 | 37.823 | Marker324812 | 0.166066262 | -0.069336779 | -0.105848407 | 1.095937239 |
| 1239 | 4 | 38.073 | Marker325318 | 0.165153797 | -0.068783069 | -0.106501126 | 1.094345863 |
| 1240 | 4 | 38.073 | Marker325319 | 0.165153797 | -0.068783069 | -0.106501126 | 1.094345863 |
| 1241 | 4 | 38.073 | Marker325317 | 0.165153797 | -0.068783069 | -0.106501126 | 1.094345863 |
| 1242 | 4 | 38.073 | Marker325681 | 0.165153797 | -0.068783069 | -0.106501126 | 1.094345863 |
| 1243 | 4 | 38.073 | Marker325673 | 0.165153797 | -0.068783069 | -0.106501126 | 1.094345863 |
| 1244 | 4 | 38.073 | Marker325320 | 0.165153797 | -0.068783069 | -0.106501126 | 1.094345863 |
| 1245 | 4 | 38.323 | Marker326177 | 0.195969081 | -0.080964686 | -0.0842205 | 1.084167671 |
| 1246 | 4 | 38.323 | Marker325675 | 0.195969081 | -0.080964686 | -0.0842205 | 1.084167671 |
| 1247 | 4 | 38.573 | Marker326739 | 0.175047632 | -0.068783069 | -0.106501126 | 1.094345863 |
| 1248 | 4 | 38.573 | Marker325682 | 0.175047632 | -0.068783069 | -0.106501126 | 1.094345863 |
| 1249 | 4 | 38.823 | Marker326988 | 0.208972946 | -0.069336779 | -0.105848407 | 1.095937239 |
| 1250 | 4 | 39.573 | Marker327147 | 0.143667954 | -0.046934461 | -0.106600893 | 0.812977577 |
| 1251 | 4 | 39.823 | Marker327146 | 0.243591503 | -0.058008658 | -0.085627706 | 0.741582239 |
| 1252 | 4 | 40.075 | Marker327853 | 0.255073957 | -0.055966899 | -0.084747387 | 0.708158086 |
| 1253 | 4 | 40.075 | Marker327027 | 0.255073957 | -0.055966899 | -0.084747387 | 0.708158086 |
| 1254 | 4 | 40.075 | Marker327028 | 0.255073957 | -0.055966899 | -0.084747387 | 0.708158086 |
| 1255 | 4 | 40.575 | Marker328551 | 0.247582043 | -0.058008658 | -0.085627706 | 0.741582239 |
| 1256 | 4 | 41.075 | Marker329359 | 0.238623832 | -0.047038328 | -0.065818815 | 0.463144333 |
| 1257 | 4 | 41.575 | Marker328553 | 0.23299577 | -0.044575273 | -0.062743516 | 0.418235848 |
| 1258 | 4 | 41.825 | Marker328835 | 0.174911547 | -0.033251232 | -0.084061246 | 0.47589142 |
| 1259 | 4 | 41.825 | Marker329642 | 0.174911547 | -0.033251232 | -0.084061246 | 0.47589142 |
| 1260 | 4 | 42.325 | Marker329508 | 0.195780796 | -0.03493179 | -0.042475164 | 0.22610774 |
| 1261 | 4 | 42.325 | Marker329502 | 0.195780796 | -0.03493179 | -0.042475164 | 0.22610774 |
| 1262 | 4 | 42.325 | Marker329363 | 0.195780796 | -0.03493179 | -0.042475164 | 0.22610774 |
| 1263 | 4 | 42.325 | Marker330378 | 0.195780796 | -0.03493179 | -0.042475164 | 0.22610774 |
| 1264 | 4 | 43.325 | Marker329643 | 0.0704956 | -0.007274283 | -0.029939964 | 0.050509624 |
| 1265 | 4 | 43.325 | Marker330379 | 0.0704956 | -0.007274283 | -0.029939964 | 0.050509624 |
| 1266 | 4 | 43.575 | Marker330381 | 0.052870453 | 0.003759398 | -0.05066237 | 0.128020061 |
| 1267 | 4 | 44.075 | Marker331120 | 0.044472667 | 0.004568106 | -0.0913028 | 0.41340546 |
| 1268 | 4 | 44.825 | Marker331121 | 0.078857461 | -0.016959064 | -0.084795322 | 0.389529141 |
| 1269 | 4 | 45.575 | Marker331981 | 0.07221468 | -0.011067194 | -0.066773715 | 0.235123975 |
| 1270 | 4 | 45.575 | Marker331982 | 0.07221468 | -0.011067194 | -0.066773715 | 0.235123975 |
| 1271 | 4 | 46.575 | Marker332412 | 0.046785064 | 0.022222222 | -0.133333333 | 0.92727029 |
| 1272 | 4 | 47.075 | Marker332505 | 0.00324802 | 0.008613618 | -0.111617083 | 0.621943709 |
| 1273 | 4 | 47.325 | Marker332516 | 0.010045793 | 0.000402576 | -0.130032206 | 0.835326384 |
| 1274 | 4 | 48.075 | Marker332558 | 0.031782303 | 0.012962963 | -0.152796674 | 1.168377168 |
| 1275 | 4 | 48.825 | Marker333286 | 0.015822936 | 0.005681818 | -0.130681818 | 0.845965354 |
| 1276 | 4 | 49.825 | Marker336600 | 0.004011538 | 0.021885522 | -0.094276094 | 0.488058061 |
| 1277 | 4 | 49.825 | Marker333721 | 0.004011538 | 0.021885522 | -0.094276094 | 0.488058061 |
| 1278 | 4 | 49.825 | Marker336314 | 0.004011538 | 0.021885522 | -0.094276094 | 0.488058061 |
| 1279 | 4 | 50.075 | Marker334403 | 0.004325803 | 0.021012007 | -0.09535163 | 0.494121405 |
| 1280 | 4 | 50.325 | Marker336809 | 0.00440542 | 0.019496855 | -0.093634458 | 0.471660238 |
| 1281 | 4 | 50.825 | Marker337874 | 0.004350937 | 0.017704918 | -0.091584422 | 0.445938781 |
| 1282 | 4 | 51.177 | Marker335097 | 0.004093865 | 0.02037037 | -0.0925548 | 0.465450368 |
| 1283 | 4 | 51.177 | Marker335098 | 0.004093865 | 0.02037037 | -0.0925548 | 0.465450368 |
| 1284 | 4 | 51.177 | Marker335317 | 0.004093865 | 0.02037037 | -0.0925548 | 0.465450368 |
| 1285 | 4 | 51.528 | Marker336810 | 0.009671758 | 0.036732236 | -0.091585377 | 0.556720309 |
| 1286 | 4 | 52.183 | Marker336811 | 0.051744417 | 0.020531401 | -0.131642512 | 0.897656899 |
| 1287 | 4 | 53.284 | Marker338194 | 0.091650045 | -0.002100088 | -0.132306813 | 0.865880528 |
| 1288 | 4 | 54.386 | Marker338193 | 0.034121379 | 0.009284333 | -0.107848926 | 0.582332184 |
| 1289 | 4 | 57.947 | Marker338256 | 0.095486351 | 0.067442794 | -0.113737289 | 1.12555604 |
| 1290 | 4 | 58.697 | Marker338255 | 0.113376275 | 0.082810047 | -0.110854919 | 1.344851726 |
| 1291 | 4 | 61.37 | Marker338253 | 0.099940859 | 0.060356057 | -0.073206279 | 0.657498683 |
| 1292 | 4 | 62.37 | Marker338245 | 0.323732547 | 0.030612245 | -0.092042878 | 0.516525092 |
| 1293 | 4 | 62.87 | Marker338244 | 0.136162106 | 0.03 | -0.048762887 | 0.213826994 |
| 1294 | 4 | 65.794 | Marker338238 | 0.14320135 | 0.062142857 | -0.06489011 | 0.625545647 |
| 1295 | 4 | 68.448 | Marker338237 | 0.101140243 | 0.01918693 | -0.087196049 | 0.413096836 |
| 1296 | 4 | 70.55 | Marker338548 | 0.302911252 | 0.022727273 | -0.0932282 | 0.482380271 |
| 1297 | 4 | 71.902 | Marker341134 | 0.418043843 | 0.035367429 | -0.079778763 | 0.446923384 |
| 1298 | 4 | 73.152 | Marker338621 | 0.061555667 | 0.053246753 | -0.03961039 | 0.385601358 |
| 1299 | 4 | 75.806 | Marker320203 | 0.031812208 | 0.031818182 | 0.000445633 | 0.111423375 |
| 1300 | 4 | 75.806 | Marker320202 | 0.031812208 | 0.031818182 | 0.000445633 | 0.111423375 |
| 1301 | 4 | 75.806 | Marker322105 | 0.031812208 | 0.031818182 | 0.000445633 | 0.111423375 |
| 1302 | 4 | 77.356 | Marker342583 | 0.074437494 | 0.051282051 | 0.036953243 | 0.360281752 |
| 1303 | 4 | 77.356 | Marker342361 | 0.074437494 | 0.051282051 | 0.036953243 | 0.360281752 |
| 1304 | 4 | 77.356 | Marker322323 | 0.074437494 | 0.051282051 | 0.036953243 | 0.360281752 |
| 1305 | 4 | 77.356 | Marker322060 | 0.074437494 | 0.051282051 | 0.036953243 | 0.360281752 |
| 1306 | 4 | 77.356 | Marker344496 | 0.074437494 | 0.051282051 | 0.036953243 | 0.360281752 |
| 1307 | 4 | 77.356 | Marker342101 | 0.074437494 | 0.051282051 | 0.036953243 | 0.360281752 |
| 1308 | 4 | 77.356 | Marker322062 | 0.074437494 | 0.051282051 | 0.036953243 | 0.360281752 |
| 1309 | 4 | 77.356 | Marker342797 | 0.074437494 | 0.051282051 | 0.036953243 | 0.360281752 |
| 1310 | 4 | 77.356 | Marker344495 | 0.074437494 | 0.051282051 | 0.036953243 | 0.360281752 |
| 1311 | 4 | 77.356 | Marker342585 | 0.074437494 | 0.051282051 | 0.036953243 | 0.360281752 |
| 1312 | 4 | 77.356 | Marker321261 | 0.074437494 | 0.051282051 | 0.036953243 | 0.360281752 |
| 1313 | 4 | 77.356 | Marker342247 | 0.074437494 | 0.051282051 | 0.036953243 | 0.360281752 |
| 1314 | 4 | 77.356 | Marker320465 | 0.074437494 | 0.051282051 | 0.036953243 | 0.360281752 |
| 1315 | 4 | 77.356 | Marker342586 | 0.074437494 | 0.051282051 | 0.036953243 | 0.360281752 |
| 1316 | 4 | 77.356 | Marker342249 | 0.074437494 | 0.051282051 | 0.036953243 | 0.360281752 |
| 1317 | 4 | 77.356 | Marker342100 | 0.074437494 | 0.051282051 | 0.036953243 | 0.360281752 |
| 1318 | 4 | 77.356 | Marker345329 | 0.074437494 | 0.051282051 | 0.036953243 | 0.360281752 |
| 1319 | 4 | 77.356 | Marker344924 | 0.074437494 | 0.051282051 | 0.036953243 | 0.360281752 |
| 1320 | 4 | 77.356 | Marker342248 | 0.074437494 | 0.051282051 | 0.036953243 | 0.360281752 |
| 1321 | 4 | 77.356 | Marker342360 | 0.074437494 | 0.051282051 | 0.036953243 | 0.360281752 |
| 1322 | 4 | 77.356 | Marker342359 | 0.074437494 | 0.051282051 | 0.036953243 | 0.360281752 |
| 1323 | 4 | 77.356 | Marker344648 | 0.074437494 | 0.051282051 | 0.036953243 | 0.360281752 |
| 1324 | 4 | 77.356 | Marker345330 | 0.074437494 | 0.051282051 | 0.036953243 | 0.360281752 |
| 1325 | 4 | 77.356 | Marker342794 | 0.074437494 | 0.051282051 | 0.036953243 | 0.360281752 |
| 1326 | 4 | 77.356 | Marker344195 | 0.074437494 | 0.051282051 | 0.036953243 | 0.360281752 |
| 1327 | 4 | 77.356 | Marker321028 | 0.074437494 | 0.051282051 | 0.036953243 | 0.360281752 |
| 1328 | 4 | 77.356 | Marker342796 | 0.074437494 | 0.051282051 | 0.036953243 | 0.360281752 |
| 1329 | 4 | 77.356 | Marker345942 | 0.074437494 | 0.051282051 | 0.036953243 | 0.360281752 |
| 1330 | 4 | 77.606 | Marker342099 | 0.155792532 | 0.051282051 | 0.03782686 | 0.363591499 |
| 1331 | 4 | 77.606 | Marker350972 | 0.155792532 | 0.051282051 | 0.03782686 | 0.363591499 |
| 1332 | 4 | 78.106 | Marker341586 | 0.072549652 | 0.051282051 | 0.03782686 | 0.363591499 |
| 1333 | 4 | 78.356 | Marker342046 | 0.055030427 | 0.042661362 | 0.020097259 | 0.221766904 |
| 1334 | 4 | 78.856 | Marker341582 | 0.060284334 | 0.045507691 | 0.010894566 | 0.234625071 |
| 1335 | 4 | 80.062 | Marker346841 | 0.124710441 | 0.055313589 | -0.041027875 | 0.415642263 |
| 1336 | 4 | 80.062 | Marker348158 | 0.124710441 | 0.055313589 | -0.041027875 | 0.415642263 |
| 1337 | 4 | 80.062 | Marker347233 | 0.124710441 | 0.055313589 | -0.041027875 | 0.415642263 |
| 1338 | 4 | 80.062 | Marker351116 | 0.124710441 | 0.055313589 | -0.041027875 | 0.415642263 |
| 1339 | 4 | 80.062 | Marker347424 | 0.124710441 | 0.055313589 | -0.041027875 | 0.415642263 |
| 1340 | 4 | 80.062 | Marker346846 | 0.124710441 | 0.055313589 | -0.041027875 | 0.415642263 |
| 1341 | 4 | 80.062 | Marker347234 | 0.124710441 | 0.055313589 | -0.041027875 | 0.415642263 |
| 1342 | 4 | 80.062 | Marker348110 | 0.124710441 | 0.055313589 | -0.041027875 | 0.415642263 |
| 1343 | 4 | 80.062 | Marker347237 | 0.124710441 | 0.055313589 | -0.041027875 | 0.415642263 |
| 1344 | 4 | 80.062 | Marker347774 | 0.124710441 | 0.055313589 | -0.041027875 | 0.415642263 |
| 1345 | 4 | 80.062 | Marker348937 | 0.124710441 | 0.055313589 | -0.041027875 | 0.415642263 |
| 1346 | 4 | 80.062 | Marker347423 | 0.124710441 | 0.055313589 | -0.041027875 | 0.415642263 |
| 1347 | 4 | 80.062 | Marker347773 | 0.124710441 | 0.055313589 | -0.041027875 | 0.415642263 |
| 1348 | 4 | 80.062 | Marker347238 | 0.124710441 | 0.055313589 | -0.041027875 | 0.415642263 |
| 1349 | 4 | 80.062 | Marker348277 | 0.124710441 | 0.055313589 | -0.041027875 | 0.415642263 |
| 1350 | 4 | 80.062 | Marker347428 | 0.124710441 | 0.055313589 | -0.041027875 | 0.415642263 |
| 1351 | 4 | 80.562 | Marker349817 | 0.082497666 | 0.04495614 | -0.02004386 | 0.240564812 |
| 1352 | 4 | 80.562 | Marker350969 | 0.082497666 | 0.04495614 | -0.02004386 | 0.240564812 |
| 1353 | 4 | 80.562 | Marker350971 | 0.082497666 | 0.04495614 | -0.02004386 | 0.240564812 |
| 1354 | 4 | 80.562 | Marker349818 | 0.082497666 | 0.04495614 | -0.02004386 | 0.240564812 |
| 1355 | 4 | 80.812 | Marker349706 | 0.100408376 | 0.044642857 | -0.020951202 | 0.239251628 |
| 1356 | 4 | 80.812 | Marker350469 | 0.100408376 | 0.044642857 | -0.020951202 | 0.239251628 |
| 1357 | 4 | 80.812 | Marker349705 | 0.100408376 | 0.044642857 | -0.020951202 | 0.239251628 |
| 1358 | 4 | 80.812 | Marker349816 | 0.100408376 | 0.044642857 | -0.020951202 | 0.239251628 |
| 1359 | 4 | 80.812 | Marker349704 | 0.100408376 | 0.044642857 | -0.020951202 | 0.239251628 |
| 1360 | 4 | 80.812 | Marker351266 | 0.100408376 | 0.044642857 | -0.020951202 | 0.239251628 |
| 1361 | 4 | 80.812 | Marker349033 | 0.100408376 | 0.044642857 | -0.020951202 | 0.239251628 |
| 1362 | 4 | 80.812 | Marker351619 | 0.100408376 | 0.044642857 | -0.020951202 | 0.239251628 |
| 1363 | 4 | 80.812 | Marker349703 | 0.100408376 | 0.044642857 | -0.020951202 | 0.239251628 |
| 1364 | 4 | 80.812 | Marker349815 | 0.100408376 | 0.044642857 | -0.020951202 | 0.239251628 |
| 1365 | 4 | 82.062 | Marker351714 | 0.512839321 | 0.051061008 | -0.062785146 | 0.475754197 |
| 1366 | 4 | 82.312 | Marker353572 | 0.49466534 | 0.053085299 | -0.065205477 | 0.513787485 |
| 1367 | 4 | 83.062 | Marker353800 | 0.450136614 | 0.066384181 | -0.051262878 | 0.608469348 |
| 1368 | 4 | 84.964 | Marker353974 | 0.185910997 | 0.007769424 | -0.020802005 | 0.027725593 |
| 1369 | 4 | 85.964 | Marker353973 | 0.087371577 | 0.032979558 | 0.027422139 | 0.158475631 |
| 1370 | 4 | 86.964 | Marker354126 | 0.056211123 | 0.012170385 | 0.039061142 | 0.092551406 |
| 1371 | 4 | 88.316 | Marker354127 | 0.113267653 | 0.014912281 | -0.030542265 | 0.069722202 |
| 1372 | 4 | 90.322 | Marker354128 | 0.05832121 | 0.022222222 | -0.015830875 | 0.066071322 |
| 1373 | 4 | 90.572 | Marker354685 | 0.02632947 | 0.006704981 | 0.007914902 | 0.008138602 |
| 1374 | 4 | 90.572 | Marker354330 | 0.02632947 | 0.006704981 | 0.007914902 | 0.008138602 |
| 1375 | 4 | 92.278 | Marker354908 | 0.0702351 | 0.000626566 | -0.008302005 | 0.003439018 |
| 1376 | 4 | 93.278 | Marker354910 | 0.043135798 | -0.010160099 | -0.025554187 | 0.044096648 |
| 1377 | 4 | 95.329 | Marker355591 | 0.009183535 | -0.012121212 | -0.02521645 | 0.048141277 |
| 1378 | 4 | 96.931 | Marker355590 | 0.002255568 | 0.004440961 | 0.019306599 | 0.02074327 |
| 1379 | 4 | 98.982 | Marker356050 | 0.052717871 | -0.003727866 | 0.067284323 | 0.22474768 |
| 1380 | 4 | 99.583 | Marker356051 | 0.042867264 | -0.00918962 | 0.053334371 | 0.148937523 |
| 1381 | 4 | 100.435 | Marker356053 | 0.120653224 | -0.004083485 | 0.068610627 | 0.233904766 |
| 1382 | 4 | 101.536 | Marker360395 | 0.087467143 | 0.010633584 | 0.047488621 | 0.124787562 |
| 1383 | 4 | 101.536 | Marker360365 | 0.087467143 | 0.010633584 | 0.047488621 | 0.124787562 |
| 1384 | 4 | 101.786 | Marker360490 | 0.087994646 | 0.01036036 | 0.046306306 | 0.118631225 |
| 1385 | 4 | 101.786 | Marker362869 | 0.087994646 | 0.01036036 | 0.046306306 | 0.118631225 |
| 1386 | 4 | 102.036 | Marker361481 | 0.097010296 | 0.016699159 | 0.059616701 | 0.20810571 |
| 1387 | 4 | 102.536 | Marker361482 | 0.116977647 | -0.003508772 | 0.07118554 | 0.251265265 |
| 1388 | 4 | 102.786 | Marker362959 | 0.090128655 | 0.008974359 | 0.049529042 | 0.130877973 |
| 1389 | 4 | 103.037 | Marker363912 | 0.044510527 | 0.029025424 | 0.045862158 | 0.199049284 |
| 1390 | 4 | 103.037 | Marker362870 | 0.044510527 | 0.029025424 | 0.045862158 | 0.199049284 |
| 1391 | 4 | 103.037 | Marker362958 | 0.044510527 | 0.029025424 | 0.045862158 | 0.199049284 |
| 1392 | 4 | 103.037 | Marker365911 | 0.044510527 | 0.029025424 | 0.045862158 | 0.199049284 |
| 1393 | 4 | 103.037 | Marker362960 | 0.044510527 | 0.029025424 | 0.045862158 | 0.199049284 |
| 1394 | 4 | 103.287 | Marker362961 | 0.043274118 | 0.029166667 | 0.046950172 | 0.205012761 |
| 1395 | 4 | 103.537 | Marker363913 | 0.038670841 | 0.029025424 | 0.045862158 | 0.199049284 |
| 1396 | 4 | 103.787 | Marker363822 | 0.025747981 | 0.020258621 | 0.026066701 | 0.079692556 |
| 1397 | 4 | 103.787 | Marker366333 | 0.025747981 | 0.020258621 | 0.026066701 | 0.079692556 |
| 1398 | 4 | 104.287 | Marker365917 | 0.059143269 | 0.018472906 | 0.029518562 | 0.081594434 |
| 1399 | 4 | 104.287 | Marker365936 | 0.059143269 | 0.018472906 | 0.029518562 | 0.081594434 |
| 1400 | 4 | 104.287 | Marker366079 | 0.059143269 | 0.018472906 | 0.029518562 | 0.081594434 |
| 1401 | 4 | 104.287 | Marker365940 | 0.059143269 | 0.018472906 | 0.029518562 | 0.081594434 |
| 1402 | 4 | 104.287 | Marker365935 | 0.059143269 | 0.018472906 | 0.029518562 | 0.081594434 |
| 1403 | 4 | 104.287 | Marker365934 | 0.059143269 | 0.018472906 | 0.029518562 | 0.081594434 |
| 1404 | 4 | 104.287 | Marker366335 | 0.059143269 | 0.018472906 | 0.029518562 | 0.081594434 |
| 1405 | 4 | 104.287 | Marker365939 | 0.059143269 | 0.018472906 | 0.029518562 | 0.081594434 |
| 1406 | 4 | 104.287 | Marker368274 | 0.059143269 | 0.018472906 | 0.029518562 | 0.081594434 |
| 1407 | 4 | 104.787 | Marker368943 | 0.027416925 | 0.008567931 | 0.010965438 | 0.014189527 |
| 1408 | 4 | 104.787 | Marker367745 | 0.027416925 | 0.008567931 | 0.010965438 | 0.014189527 |
| 1409 | 4 | 104.787 | Marker367746 | 0.027416925 | 0.008567931 | 0.010965438 | 0.014189527 |
| 1410 | 4 | 105.037 | Marker370282 | 0.024688121 | 0.009021652 | 0.012170877 | 0.016474556 |
| 1411 | 4 | 105.037 | Marker368277 | 0.024688121 | 0.009021652 | 0.012170877 | 0.016474556 |
| 1412 | 4 | 105.287 | Marker369377 | 0.024782583 | 0.009459992 | 0.013376761 | 0.018918651 |
| 1413 | 4 | 105.287 | Marker369207 | 0.024782583 | 0.009459992 | 0.013376761 | 0.018918651 |
| 1414 | 4 | 105.287 | Marker369201 | 0.024782583 | 0.009459992 | 0.013376761 | 0.018918651 |
| 1415 | 4 | 105.287 | Marker369206 | 0.024782583 | 0.009459992 | 0.013376761 | 0.018918651 |
| 1416 | 4 | 105.287 | Marker369685 | 0.024782583 | 0.009459992 | 0.013376761 | 0.018918651 |
| 1417 | 4 | 105.287 | Marker369109 | 0.024782583 | 0.009459992 | 0.013376761 | 0.018918651 |
| 1418 | 4 | 105.287 | Marker372310 | 0.024782583 | 0.009459992 | 0.013376761 | 0.018918651 |
| 1419 | 4 | 105.287 | Marker369110 | 0.024782583 | 0.009459992 | 0.013376761 | 0.018918651 |
| 1420 | 4 | 105.287 | Marker369376 | 0.024782583 | 0.009459992 | 0.013376761 | 0.018918651 |
| 1421 | 4 | 105.287 | Marker369202 | 0.024782583 | 0.009459992 | 0.013376761 | 0.018918651 |
| 1422 | 4 | 105.287 | Marker369210 | 0.024782583 | 0.009459992 | 0.013376761 | 0.018918651 |
| 1423 | 4 | 105.537 | Marker370285 | 0.02893505 | 0.001550388 | -0.004387267 | 0.001203023 |
| 1424 | 4 | 105.537 | Marker370312 | 0.02893505 | 0.001550388 | -0.004387267 | 0.001203023 |
| 1425 | 4 | 105.537 | Marker370541 | 0.02893505 | 0.001550388 | -0.004387267 | 0.001203023 |
| 1426 | 4 | 105.537 | Marker370283 | 0.02893505 | 0.001550388 | -0.004387267 | 0.001203023 |
| 1427 | 4 | 105.537 | Marker370284 | 0.02893505 | 0.001550388 | -0.004387267 | 0.001203023 |
| 1428 | 4 | 105.787 | Marker370540 | 0.04341899 | -0.006099886 | -0.022103887 | 0.028480274 |
| 1429 | 4 | 105.787 | Marker372483 | 0.04341899 | -0.006099886 | -0.022103887 | 0.028480274 |
| 1430 | 4 | 106.037 | Marker372311 | 0.052235861 | -0.017174083 | -0.000963343 | 0.032527516 |
| 1431 | 4 | 106.037 | Marker372485 | 0.052235861 | -0.017174083 | -0.000963343 | 0.032527516 |
| 1432 | 4 | 106.037 | Marker372312 | 0.052235861 | -0.017174083 | -0.000963343 | 0.032527516 |
| 1433 | 4 | 106.287 | Marker372540 | 0.053669511 | -0.016593363 | -0.002215956 | 0.030603788 |
| 1434 | 4 | 106.287 | Marker372484 | 0.053669511 | -0.016593363 | -0.002215956 | 0.030603788 |
| 1435 | 4 | 106.537 | Marker373254 | 0.068045686 | -0.029078845 | 0.021579717 | 0.114893841 |
| 1436 | 4 | 106.787 | Marker374114 | 0.096415272 | -0.040983607 | 0.045037899 | 0.2816435 |
| 1437 | 4 | 107.287 | Marker374598 | 0.073939642 | -0.029872495 | 0.024828359 | 0.12728144 |
| 1438 | 4 | 107.287 | Marker373637 | 0.073939642 | -0.029872495 | 0.024828359 | 0.12728144 |
| 1439 | 4 | 107.787 | Marker374115 | 0.062020355 | -0.017727793 | 0.000276709 | 0.03457246 |
| 1440 | 4 | 107.787 | Marker374649 | 0.062020355 | -0.017727793 | 0.000276709 | 0.03457246 |
| 1441 | 4 | 107.787 | Marker374116 | 0.062020355 | -0.017727793 | 0.000276709 | 0.03457246 |
| 1442 | 4 | 108.537 | Marker374650 | 0.073773215 | -0.017857143 | -0.002318296 | 0.03542538 |
| 1443 | 4 | 109.037 | Marker375093 | 0.090300575 | -0.017982637 | -0.004907455 | 0.036930317 |
| 1444 | 4 | 109.537 | Marker374651 | 0.090000532 | -0.018103448 | -0.007497388 | 0.039084216 |
| 1445 | 4 | 109.537 | Marker376751 | 0.090000532 | -0.018103448 | -0.007497388 | 0.039084216 |
| 1446 | 4 | 109.537 | Marker375092 | 0.090000532 | -0.018103448 | -0.007497388 | 0.039084216 |
| 1447 | 4 | 109.537 | Marker374747 | 0.090000532 | -0.018103448 | -0.007497388 | 0.039084216 |
| 1448 | 4 | 109.537 | Marker374652 | 0.090000532 | -0.018103448 | -0.007497388 | 0.039084216 |
| 1449 | 4 | 109.537 | Marker375057 | 0.090000532 | -0.018103448 | -0.007497388 | 0.039084216 |
| 1450 | 4 | 109.787 | Marker375754 | 0.09302216 | -0.030282935 | 0.014076039 | 0.109907269 |
| 1451 | 4 | 110.037 | Marker376880 | 0.140676872 | -0.018103448 | -0.007497388 | 0.039084216 |
| 1452 | 4 | 110.537 | Marker376881 | 0.154064327 | -0.010697475 | 0.011229108 | 0.018600815 |
| 1453 | 4 | 111.037 | Marker378730 | 0.146460305 | 0.010714286 | 0.005021216 | 0.013974415 |
| 1454 | 4 | 111.288 | Marker377733 | 0.122964698 | -0.001144689 | 0.026004633 | 0.03350122 |
| 1455 | 4 | 111.288 | Marker377732 | 0.122964698 | -0.001144689 | 0.026004633 | 0.03350122 |
| 1456 | 4 | 111.288 | Marker377677 | 0.122964698 | -0.001144689 | 0.026004633 | 0.03350122 |
| 1457 | 4 | 111.288 | Marker376965 | 0.122964698 | -0.001144689 | 0.026004633 | 0.03350122 |
| 1458 | 4 | 111.288 | Marker378863 | 0.122964698 | -0.001144689 | 0.026004633 | 0.03350122 |
| 1459 | 4 | 111.288 | Marker377711 | 0.122964698 | -0.001144689 | 0.026004633 | 0.03350122 |
| 1460 | 4 | 111.288 | Marker377873 | 0.122964698 | -0.001144689 | 0.026004633 | 0.03350122 |
| 1461 | 4 | 111.538 | Marker378865 | 0.121506832 | -0.001631702 | 0.02475615 | 0.030499377 |
| 1462 | 4 | 111.788 | Marker378864 | 0.139612192 | -0.01007326 | 0.00727214 | 0.013642996 |
| 1463 | 4 | 111.788 | Marker378894 | 0.139612192 | -0.01007326 | 0.00727214 | 0.013642996 |
| 1464 | 4 | 112.288 | Marker379026 | 0.056372806 | -0.001144689 | 0.026004633 | 0.03350122 |
| 1465 | 4 | 113.038 | Marker378935 | 0.026377987 | -0.014880952 | 0.052218615 | 0.157666172 |
| 1466 | 4 | 113.038 | Marker379254 | 0.026377987 | -0.014880952 | 0.052218615 | 0.157666172 |
| 1467 | 4 | 113.038 | Marker379215 | 0.026377987 | -0.014880952 | 0.052218615 | 0.157666172 |
| 1468 | 4 | 113.038 | Marker379782 | 0.026377987 | -0.014880952 | 0.052218615 | 0.157666172 |
| 1469 | 4 | 114.038 | Marker379783 | 0.031825528 | -0.054545455 | 0.016161616 | 0.338635243 |
| 1470 | 4 | 114.038 | Marker379273 | 0.031825528 | -0.054545455 | 0.016161616 | 0.338635243 |
| 1471 | 4 | 114.288 | Marker380819 | 0.042881498 | -0.054545455 | 0.015454545 | 0.337601315 |
| 1472 | 4 | 114.538 | Marker379788 | 0.076296908 | -0.042917548 | -0.005567301 | 0.204622992 |
| 1473 | 4 | 114.538 | Marker381389 | 0.076296908 | -0.042917548 | -0.005567301 | 0.204622992 |
| 1474 | 4 | 115.038 | Marker381383 | 0.059895024 | -0.054545455 | 0.016161616 | 0.338635243 |
| 1475 | 4 | 115.038 | Marker381390 | 0.059895024 | -0.054545455 | 0.016161616 | 0.338635243 |
| 1476 | 4 | 115.038 | Marker381382 | 0.059895024 | -0.054545455 | 0.016161616 | 0.338635243 |
| 1477 | 4 | 115.288 | Marker382009 | 0.045992629 | -0.054545455 | 0.016883117 | 0.339741196 |
| 1478 | 4 | 115.288 | Marker382010 | 0.045992629 | -0.054545455 | 0.016883117 | 0.339741196 |
| 1479 | 4 | 116.038 | Marker382191 | 0.015440698 | -0.054545455 | 0.016161616 | 0.338635243 |
| 1480 | 4 | 116.288 | Marker382813 | 0.008347528 | -0.046296296 | 0.033703704 | 0.289087818 |
| 1481 | 4 | 116.288 | Marker382011 | 0.008347528 | -0.046296296 | 0.033703704 | 0.289087818 |
| 1482 | 4 | 116.288 | Marker382877 | 0.008347528 | -0.046296296 | 0.033703704 | 0.289087818 |
| 1483 | 4 | 116.288 | Marker382814 | 0.008347528 | -0.046296296 | 0.033703704 | 0.289087818 |
| 1484 | 4 | 116.288 | Marker382980 | 0.008347528 | -0.046296296 | 0.033703704 | 0.289087818 |
| 1485 | 4 | 116.538 | Marker384109 | 0.00392199 | -0.037735849 | 0.051373062 | 0.283522068 |
| 1486 | 4 | 116.788 | Marker382993 | 0.008746493 | -0.037037037 | 0.052962963 | 0.285926977 |
| 1487 | 4 | 116.788 | Marker383779 | 0.008746493 | -0.037037037 | 0.052962963 | 0.285926977 |
| 1488 | 4 | 117.038 | Marker384350 | 0.011972115 | -0.025132275 | 0.030266122 | 0.113361823 |
| 1489 | 4 | 117.038 | Marker382981 | 0.011972115 | -0.025132275 | 0.030266122 | 0.113361823 |
| 1490 | 4 | 117.288 | Marker384282 | 0.012927699 | -0.025831087 | 0.028790762 | 0.113006317 |
| 1491 | 4 | 117.288 | Marker384913 | 0.012927699 | -0.025831087 | 0.028790762 | 0.113006317 |
| 1492 | 4 | 117.288 | Marker384138 | 0.012927699 | -0.025831087 | 0.028790762 | 0.113006317 |
| 1493 | 4 | 117.538 | Marker385444 | 0.013460582 | -0.026556777 | 0.027303603 | 0.113101507 |
| 1494 | 4 | 118.038 | Marker384973 | 0.044843191 | -0.026107942 | 0.029844165 | 0.117575226 |
| 1495 | 4 | 118.038 | Marker386068 | 0.044843191 | -0.026107942 | 0.029844165 | 0.117575226 |
| 1496 | 4 | 118.288 | Marker385556 | 0.074277294 | -0.02540913 | 0.031335056 | 0.11808969 |
| 1497 | 4 | 118.288 | Marker385469 | 0.074277294 | -0.02540913 | 0.031335056 | 0.11808969 |
| 1498 | 4 | 118.288 | Marker385448 | 0.074277294 | -0.02540913 | 0.031335056 | 0.11808969 |
| 1499 | 4 | 118.288 | Marker385555 | 0.074277294 | -0.02540913 | 0.031335056 | 0.11808969 |
| 1500 | 4 | 118.288 | Marker385554 | 0.074277294 | -0.02540913 | 0.031335056 | 0.11808969 |
| 1501 | 4 | 118.288 | Marker387182 | 0.074277294 | -0.02540913 | 0.031335056 | 0.11808969 |
| 1502 | 4 | 118.288 | Marker385470 | 0.074277294 | -0.02540913 | 0.031335056 | 0.11808969 |
| 1503 | 4 | 118.538 | Marker387265 | 0.227504539 | -0.013227513 | 0.00846037 | 0.02258224 |
| 1504 | 4 | 118.538 | Marker386397 | 0.227504539 | -0.013227513 | 0.00846037 | 0.02258224 |
| 1505 | 4 | 118.538 | Marker386109 | 0.227504539 | -0.013227513 | 0.00846037 | 0.02258224 |
| 1506 | 4 | 118.538 | Marker386110 | 0.227504539 | -0.013227513 | 0.00846037 | 0.02258224 |
| 1507 | 4 | 118.538 | Marker386070 | 0.227504539 | -0.013227513 | 0.00846037 | 0.02258224 |
| 1508 | 4 | 118.538 | Marker386069 | 0.227504539 | -0.013227513 | 0.00846037 | 0.02258224 |
| 1509 | 4 | 118.538 | Marker386396 | 0.227504539 | -0.013227513 | 0.00846037 | 0.02258224 |
| 1510 | 4 | 118.538 | Marker386690 | 0.227504539 | -0.013227513 | 0.00846037 | 0.02258224 |
| 1511 | 4 | 118.788 | Marker387560 | 0.228791976 | -0.013926325 | 0.007082078 | 0.023635879 |
| 1512 | 4 | 119.288 | Marker387752 | 0.195988392 | -0.035881801 | 0.007035647 | 0.143639632 |
| 1513 | 4 | 119.288 | Marker387267 | 0.195988392 | -0.035881801 | 0.007035647 | 0.143639632 |
| 1514 | 4 | 119.538 | Marker387754 | 0.203168094 | -0.027020564 | 0.024779668 | 0.109442175 |
| 1515 | 4 | 120.288 | Marker387885 | 0.152839057 | -0.04 | 0.04490566 | 0.272382542 |
| 1516 | 4 | 120.288 | Marker387753 | 0.152839057 | -0.04 | 0.04490566 | 0.272382542 |
| 1517 | 4 | 120.788 | Marker388641 | 0.140405917 | -0.057692308 | 0.009615385 | 0.369756005 |
| 1518 | 4 | 120.788 | Marker389080 | 0.140405917 | -0.057692308 | 0.009615385 | 0.369756005 |
| 1519 | 4 | 121.538 | Marker389527 | 0.138223416 | -0.049019608 | 0.027170868 | 0.298416612 |
| 1520 | 4 | 122.038 | Marker389821 | 0.184138126 | -0.045192308 | -0.013049451 | 0.234199006 |
| 1521 | 4 | 122.038 | Marker389532 | 0.184138126 | -0.045192308 | -0.013049451 | 0.234199006 |
| 1522 | 4 | 122.038 | Marker389081 | 0.184138126 | -0.045192308 | -0.013049451 | 0.234199006 |
| 1523 | 4 | 122.288 | Marker389774 | 0.183176347 | -0.044103774 | -0.011411466 | 0.221366714 |
| 1524 | 4 | 122.288 | Marker390239 | 0.183176347 | -0.044103774 | -0.011411466 | 0.221366714 |
| 1525 | 4 | 122.538 | Marker390745 | 0.188889411 | -0.052314815 | -0.028771125 | 0.344770131 |
| 1526 | 4 | 122.788 | Marker390747 | 0.196970905 | -0.060227273 | -0.046011586 | 0.508761998 |
| 1527 | 4 | 122.788 | Marker390293 | 0.196970905 | -0.060227273 | -0.046011586 | 0.508761998 |
| 1528 | 4 | 123.538 | Marker390748 | 0.252566023 | -0.058333333 | -0.088915858 | 0.77449644 |
| 1529 | 4 | 123.788 | Marker391195 | 0.250587826 | -0.05990566 | -0.090674891 | 0.811007849 |
| 1530 | 4 | 124.038 | Marker391420 | 0.25055884 | -0.060515416 | -0.089878429 | 0.811994561 |
| 1531 | 4 | 124.288 | Marker391983 | 0.247485172 | -0.062148218 | -0.091697936 | 0.850829467 |
| 1532 | 4 | 124.538 | Marker391421 | 0.247691707 | -0.062728938 | -0.09093051 | 0.851892905 |
| 1533 | 4 | 124.538 | Marker392177 | 0.247691707 | -0.062728938 | -0.09093051 | 0.851892905 |
| 1534 | 4 | 124.788 | Marker392176 | 0.316469837 | -0.070530099 | -0.108345225 | 1.141277617 |
| 1535 | 4 | 124.788 | Marker392250 | 0.316469837 | -0.070530099 | -0.108345225 | 1.141277617 |
| 1536 | 4 | 124.788 | Marker392175 | 0.316469837 | -0.070530099 | -0.108345225 | 1.141277617 |
| 1537 | 4 | 125.038 | Marker392398 | 0.245926468 | -0.082711716 | -0.086165549 | 1.132566502 |
| 1538 | 4 | 125.288 | Marker392837 | 0.213517717 | -0.071705426 | -0.06496124 | 0.782727934 |
| 1539 | 4 | 125.288 | Marker393396 | 0.213517717 | -0.071705426 | -0.06496124 | 0.782727934 |
| 1540 | 4 | 126.038 | Marker393708 | 0.428272228 | -0.060606061 | -0.085858586 | 0.777868243 |
| 1541 | 4 | 126.288 | Marker393918 | 0.152686291 | -0.071705426 | -0.06496124 | 0.782727934 |
| 1542 | 4 | 126.538 | Marker393397 | 0.151460596 | -0.071428571 | -0.065535125 | 0.782110225 |
| 1543 | 4 | 126.788 | Marker393920 | 0.168908886 | -0.079004329 | -0.082813853 | 1.037550407 |
| 1544 | 4 | 127.038 | Marker395277 | 0.21180832 | -0.086309524 | -0.100018038 | 1.329655461 |
| 1545 | 4 | 127.038 | Marker393919 | 0.21180832 | -0.086309524 | -0.100018038 | 1.329655461 |
| 1546 | 4 | 127.288 | Marker395623 | 0.174084747 | -0.075814536 | -0.079215897 | 0.953430239 |
| 1547 | 4 | 127.288 | Marker393922 | 0.174084747 | -0.075814536 | -0.079215897 | 0.953430239 |
| 1548 | 4 | 127.538 | Marker395955 | 0.156082192 | -0.076091391 | -0.078728649 | 0.954227341 |
| 1549 | 4 | 127.788 | Marker395624 | 0.142306054 | -0.076355662 | -0.078249601 | 0.954915573 |
| 1550 | 4 | 127.788 | Marker395958 | 0.142306054 | -0.076355662 | -0.078249601 | 0.954915573 |
| 1551 | 4 | 128.288 | Marker396194 | 0.100109667 | -0.089285714 | -0.058035714 | 1.052996623 |
| 1552 | 4 | 128.538 | Marker397078 | 0.208250797 | -0.096491228 | -0.075438596 | 1.318880545 |
| 1553 | 4 | 128.788 | Marker397080 | 0.135807087 | -0.098214286 | -0.077380952 | 1.371044905 |
| 1554 | 4 | 129.538 | Marker397465 | 0.103689376 | -0.118012422 | -0.07522066 | 1.828092712 |
| 1555 | 4 | 130.288 | Marker398003 | 0.038949765 | -0.111507937 | -0.014980159 | 1.382180763 |
| 1556 | 4 | 131.288 | Marker398078 | 0.070225253 | -0.117049808 | -0.030052172 | 1.558455531 |
| 1557 | 4 | 131.538 | Marker398945 | 0.070742115 | -0.116566717 | -0.030077703 | 1.54609318 |
| 1558 | 4 | 131.538 | Marker398946 | 0.070742115 | -0.116566717 | -0.030077703 | 1.54609318 |
| 1559 | 4 | 132.038 | Marker399567 | 0.090015725 | -0.107602339 | -0.05380117 | 1.427490765 |
| 1560 | 4 | 133.039 | Marker399068 | 0.003215801 | -0.081818182 | -0.040581068 | 0.823963799 |
| 1561 | 4 | 133.289 | Marker399879 | 0.003265118 | -0.083333333 | -0.042517007 | 0.859851068 |
| 1562 | 4 | 133.539 | Marker400057 | 0.071371623 | -0.072727273 | -0.021180881 | 0.606928094 |
| 1563 | 4 | 134.289 | Marker400246 | 0.063259014 | -0.061857708 | -0.041930171 | 0.512603916 |
| 1564 | 4 | 134.54 | Marker400426 | 0.019905421 | -0.063204509 | -0.043706526 | 0.538960391 |
| 1565 | 4 | 134.79 | Marker400690 | 0.020753238 | -0.064602133 | -0.045524937 | 0.56695706 |
| 1566 | 4 | 135.04 | Marker400548 | 0.010188774 | -0.072463768 | -0.063275063 | 0.783939939 |
| 1567 | 4 | 135.04 | Marker400543 | 0.010188774 | -0.072463768 | -0.063275063 | 0.783939939 |
| 1568 | 4 | 135.04 | Marker400473 | 0.010188774 | -0.072463768 | -0.063275063 | 0.783939939 |
| 1569 | 4 | 135.04 | Marker400552 | 0.010188774 | -0.072463768 | -0.063275063 | 0.783939939 |
| 1570 | 4 | 135.04 | Marker401559 | 0.010188774 | -0.072463768 | -0.063275063 | 0.783939939 |
| 1571 | 4 | 135.04 | Marker400542 | 0.010188774 | -0.072463768 | -0.063275063 | 0.783939939 |
| 1572 | 4 | 135.29 | Marker400936 | 0.008130804 | -0.080039526 | -0.080945323 | 1.040429874 |
| 1573 | 4 | 135.29 | Marker401152 | 0.008130804 | -0.080039526 | -0.080945323 | 1.040429874 |
| 1574 | 4 | 135.29 | Marker400934 | 0.008130804 | -0.080039526 | -0.080945323 | 1.040429874 |
| 1575 | 4 | 135.29 | Marker401992 | 0.008130804 | -0.080039526 | -0.080945323 | 1.040429874 |
| 1576 | 4 | 135.29 | Marker400691 | 0.008130804 | -0.080039526 | -0.080945323 | 1.040429874 |
| 1577 | 4 | 135.29 | Marker400943 | 0.008130804 | -0.080039526 | -0.080945323 | 1.040429874 |
| 1578 | 4 | 135.29 | Marker400935 | 0.008130804 | -0.080039526 | -0.080945323 | 1.040429874 |
| 1579 | 4 | 135.29 | Marker400929 | 0.008130804 | -0.080039526 | -0.080945323 | 1.040429874 |
| 1580 | 4 | 135.29 | Marker401149 | 0.008130804 | -0.080039526 | -0.080945323 | 1.040429874 |
| 1581 | 4 | 135.54 | Marker402591 | 0.011850087 | -0.08734472 | -0.098557535 | 1.335060808 |
| 1582 | 4 | 135.54 | Marker401153 | 0.011850087 | -0.08734472 | -0.098557535 | 1.335060808 |
| 1583 | 4 | 136.29 | Marker402629 | 0.032933579 | -0.085866261 | -0.139057751 | 1.788456216 |
| 1584 | 4 | 137.04 | Marker402954 | 0.023408223 | -0.118181818 | -0.118181818 | 2.25232765 |
| 1585 | 4 | 137.04 | Marker403079 | 0.023408223 | -0.118181818 | -0.118181818 | 2.25232765 |
| 1586 | 4 | 137.54 | Marker403491 | 0.046013678 | -0.109090909 | -0.098338221 | 1.80679654 |
| 1587 | 4 | 137.54 | Marker403078 | 0.046013678 | -0.109090909 | -0.098338221 | 1.80679654 |
| 1588 | 4 | 137.54 | Marker403080 | 0.046013678 | -0.109090909 | -0.098338221 | 1.80679654 |
| 1589 | 4 | 137.79 | Marker403494 | 0.0889606 | -0.107142857 | -0.096273292 | 1.739840409 |
| 1590 | 4 | 137.79 | Marker403081 | 0.0889606 | -0.107142857 | -0.096273292 | 1.739840409 |
| 1591 | 4 | 138.29 | Marker403492 | 0.026373035 | -0.101851852 | -0.080575256 | 1.477154666 |
| 1592 | 4 | 138.79 | Marker403495 | 0.017680627 | -0.111111111 | -0.100472813 | 1.877506085 |
| 1593 | 4 | 139.04 | Marker403997 | 0.019279939 | -0.111111111 | -0.100584795 | 1.878641261 |
| 1594 | 4 | 139.04 | Marker403599 | 0.019279939 | -0.111111111 | -0.100584795 | 1.878641261 |
| 1595 | 4 | 139.29 | Marker403999 | 0.010128063 | -0.1 | -0.078723404 | 1.420840764 |
| 1596 | 4 | 139.29 | Marker403601 | 0.010128063 | -0.1 | -0.078723404 | 1.420840764 |
| 1597 | 4 | 139.29 | Marker403998 | 0.010128063 | -0.1 | -0.078723404 | 1.420840764 |
| 1598 | 4 | 139.79 | Marker403994 | 0.084325999 | -0.113207547 | -0.102790881 | 1.953392428 |
| 1599 | 4 | 139.79 | Marker404001 | 0.084325999 | -0.113207547 | -0.102790881 | 1.953392428 |
| 1600 | 4 | 140.04 | Marker406254 | 0.042206053 | -0.115384615 | -0.105075337 | 2.032500569 |
| 1601 | 4 | 140.04 | Marker404000 | 0.042206053 | -0.115384615 | -0.105075337 | 2.032500569 |
| 1602 | 4 | 140.04 | Marker406048 | 0.042206053 | -0.115384615 | -0.105075337 | 2.032500569 |
| 1603 | 4 | 140.79 | Marker406132 | 0.037049725 | -0.125588697 | -0.0843472 | 2.106244097 |
| 1604 | 4 | 140.79 | Marker407305 | 0.037049725 | -0.125588697 | -0.0843472 | 2.106244097 |
| 1605 | 4 | 141.79 | Marker406255 | 0.144273541 | -0.105950653 | -0.124818578 | 2.02903175 |
| 1606 | 4 | 141.79 | Marker408357 | 0.144273541 | -0.105950653 | -0.124818578 | 2.02903175 |
| 1607 | 4 | 143.04 | Marker408038 | 0.219628932 | -0.105769231 | -0.125 | 2.027038627 |
| 1608 | 4 | 143.04 | Marker408360 | 0.219628932 | -0.105769231 | -0.125 | 2.027038627 |
| 1609 | 4 | 143.79 | Marker408619 | 0.141064225 | -0.103592163 | -0.122822932 | 1.949322986 |
| 1610 | 4 | 143.79 | Marker408963 | 0.141064225 | -0.103592163 | -0.122822932 | 1.949322986 |
| 1611 | 4 | 143.79 | Marker408615 | 0.141064225 | -0.103592163 | -0.122822932 | 1.949322986 |
| 1612 | 4 | 143.79 | Marker408358 | 0.141064225 | -0.103592163 | -0.122822932 | 1.949322986 |
| 1613 | 4 | 143.79 | Marker408620 | 0.141064225 | -0.103592163 | -0.122822932 | 1.949322986 |
| 1614 | 4 | 144.04 | Marker408621 | 0.141064225 | -0.103592163 | -0.122822932 | 1.949322986 |
| 1615 | 4 | 144.54 | Marker410164 | 0.143660024 | -0.101677149 | -0.120545073 | 1.877829594 |
| 1616 | 4 | 145.04 | Marker411791 | 0.154884438 | -0.10130719 | -0.120915033 | 1.87396785 |
| 1617 | 4 | 145.29 | Marker410927 | 0.144123531 | -0.090196078 | -0.098814911 | 1.393824942 |
| 1618 | 4 | 146.29 | Marker410165 | 0.128146783 | -0.132445431 | -0.1020849 | 2.469648411 |
| 1619 | 4 | 147.041 | Marker413317 | 0.13537846 | -0.135204082 | -0.104379252 | 2.575372061 |
| 1620 | 4 | 147.041 | Marker413200 | 0.13537846 | -0.135204082 | -0.104379252 | 2.575372061 |
| 1621 | 4 | 147.041 | Marker411813 | 0.13537846 | -0.135204082 | -0.104379252 | 2.575372061 |
| 1622 | 4 | 147.041 | Marker411792 | 0.13537846 | -0.135204082 | -0.104379252 | 2.575372061 |
| 1623 | 4 | 148.541 | Marker412336 | 0.207695766 | -0.107765152 | -0.139236783 | 2.263060545 |
| 1624 | 4 | 148.541 | Marker412730 | 0.207695766 | -0.107765152 | -0.139236783 | 2.263060545 |
| 1625 | 5 | 0 | Marker419932 | 0.614673359 | 0.00952381 | 0.22372134 | 2.48679631 |
| 1626 | 5 | 0 | Marker419931 | 0.614673359 | 0.00952381 | 0.22372134 | 2.48679631 |
| 1627 | 5 | 0 | Marker419924 | 0.614673359 | 0.00952381 | 0.22372134 | 2.48679631 |
| 1628 | 5 | 0 | Marker419930 | 0.614673359 | 0.00952381 | 0.22372134 | 2.48679631 |
| 1629 | 5 | 0 | Marker419923 | 0.614673359 | 0.00952381 | 0.22372134 | 2.48679631 |
| 1630 | 5 | 0 | Marker419922 | 0.614673359 | 0.00952381 | 0.22372134 | 2.48679631 |
| 1631 | 5 | 0 | Marker419921 | 0.614673359 | 0.00952381 | 0.22372134 | 2.48679631 |
| 1632 | 5 | 2.252 | Marker418979 | 0.564117439 | -0.010065421 | 0.147637268 | 1.085355976 |
| 1633 | 5 | 3.402 | Marker419603 | 0.463619953 | -0.02704211 | 0.139286134 | 1.032105979 |
| 1634 | 5 | 3.402 | Marker419605 | 0.463619953 | -0.02704211 | 0.139286134 | 1.032105979 |
| 1635 | 5 | 3.402 | Marker419604 | 0.463619953 | -0.02704211 | 0.139286134 | 1.032105979 |
| 1636 | 5 | 4.331 | Marker415394 | 0.536175472 | -0.018236074 | 0.150419982 | 1.149476942 |
| 1637 | 5 | 4.331 | Marker420418 | 0.536175472 | -0.018236074 | 0.150419982 | 1.149476942 |
| 1638 | 5 | 4.331 | Marker420419 | 0.536175472 | -0.018236074 | 0.150419982 | 1.149476942 |
| 1639 | 5 | 4.331 | Marker420417 | 0.536175472 | -0.018236074 | 0.150419982 | 1.149476942 |
| 1640 | 5 | 4.881 | Marker415393 | 0.236289934 | -0.00177883 | 0.142286266 | 1.000163244 |
| 1641 | 5 | 5.131 | Marker417427 | 0.216465639 | -0.010410348 | 0.12382016 | 0.767056273 |
| 1642 | 5 | 6.782 | Marker416917 | 0.171440674 | -0.017804497 | 0.099639947 | 0.522156964 |
| 1643 | 5 | 7.032 | Marker416650 | 0.114331304 | -0.006987578 | 0.07653645 | 0.293815366 |
| 1644 | 5 | 7.282 | Marker418359 | 0.116315267 | -0.007312253 | 0.075115283 | 0.283651258 |
| 1645 | 5 | 7.633 | Marker418429 | 0.118544646 | -0.007070707 | 0.073799854 | 0.273640895 |
| 1646 | 5 | 7.633 | Marker418428 | 0.118544646 | -0.007070707 | 0.073799854 | 0.273640895 |
| 1647 | 5 | 7.884 | Marker416159 | 0.118544646 | -0.007070707 | 0.073799854 | 0.273640895 |
| 1648 | 5 | 8.384 | Marker417551 | 0.115119745 | -0.006987578 | 0.07653645 | 0.293815366 |
| 1649 | 5 | 12.557 | Marker426071 | 0.282077926 | -0.029203168 | 0.119286977 | 0.790497794 |
| 1650 | 5 | 13.057 | Marker426020 | 0.166142064 | -0.024518206 | 0.080818663 | 0.385228161 |
| 1651 | 5 | 14.413 | Marker429456 | 0.278160241 | -0.037701196 | 0.09955564 | 0.639213199 |
| 1652 | 5 | 15.768 | Marker430198 | 0.118625757 | -0.018781683 | 0.056949418 | 0.197094673 |
| 1653 | 5 | 17.123 | Marker431436 | 0.172551239 | -0.008738278 | 0.069522592 | 0.246096293 |
| 1654 | 5 | 19.175 | Marker435457 | 0.085026584 | 0.019317161 | -0.017097405 | 0.054894192 |
| 1655 | 5 | 19.175 | Marker435734 | 0.085026584 | 0.019317161 | -0.017097405 | 0.054894192 |
| 1656 | 5 | 19.425 | Marker434853 | 0.104721135 | 0.029100529 | 0.003169365 | 0.093837614 |
| 1657 | 5 | 19.425 | Marker434855 | 0.104721135 | 0.029100529 | 0.003169365 | 0.093837614 |
| 1658 | 5 | 19.425 | Marker434854 | 0.104721135 | 0.029100529 | 0.003169365 | 0.093837614 |
| 1659 | 5 | 20.175 | Marker435311 | 0.041839594 | 0.051248923 | -0.03828596 | 0.357804019 |
| 1660 | 5 | 20.175 | Marker435309 | 0.041839594 | 0.051248923 | -0.03828596 | 0.357804019 |
| 1661 | 5 | 20.175 | Marker435310 | 0.041839594 | 0.051248923 | -0.03828596 | 0.357804019 |
| 1662 | 5 | 20.175 | Marker435308 | 0.041839594 | 0.051248923 | -0.03828596 | 0.357804019 |
| 1663 | 5 | 25.399 | Marker441239 | 0.169650753 | 0.015130985 | 0.064389713 | 0.231818576 |
| 1664 | 5 | 25.399 | Marker441238 | 0.169650753 | 0.015130985 | 0.064389713 | 0.231818576 |
| 1665 | 5 | 25.899 | Marker440255 | 0.248024187 | 0.003561254 | 0.083689459 | 0.347988799 |
| 1666 | 5 | 25.899 | Marker440256 | 0.248024187 | 0.003561254 | 0.083689459 | 0.347988799 |
| 1667 | 5 | 27.149 | Marker443670 | 0.19685307 | 0.005522319 | 0.044120472 | 0.099979012 |
| 1668 | 5 | 27.149 | Marker443674 | 0.19685307 | 0.005522319 | 0.044120472 | 0.099979012 |
| 1669 | 5 | 27.649 | Marker443257 | 0.272404686 | -0.006730769 | 0.063507326 | 0.203472504 |
| 1670 | 5 | 27.649 | Marker443256 | 0.272404686 | -0.006730769 | 0.063507326 | 0.203472504 |
| 1671 | 5 | 27.649 | Marker443258 | 0.272404686 | -0.006730769 | 0.063507326 | 0.203472504 |
| 1672 | 5 | 27.649 | Marker441753 | 0.272404686 | -0.006730769 | 0.063507326 | 0.203472504 |
| 1673 | 5 | 28.649 | Marker444279 | 0.711646291 | -0.042232278 | 0.086212486 | 0.556799306 |
| 1674 | 5 | 29.399 | Marker444308 | 0.698125264 | -0.041911765 | 0.08686182 | 0.559385402 |
| 1675 | 5 | 29.399 | Marker444301 | 0.698125264 | -0.041911765 | 0.08686182 | 0.559385402 |
| 1676 | 5 | 29.399 | Marker444309 | 0.698125264 | -0.041911765 | 0.08686182 | 0.559385402 |
| 1677 | 5 | 29.399 | Marker444307 | 0.698125264 | -0.041911765 | 0.08686182 | 0.559385402 |
| 1678 | 5 | 29.399 | Marker444302 | 0.698125264 | -0.041911765 | 0.08686182 | 0.559385402 |
| 1679 | 5 | 29.399 | Marker444310 | 0.698125264 | -0.041911765 | 0.08686182 | 0.559385402 |
| 1680 | 5 | 31.2 | Marker445750 | 0.821411579 | -0.044230769 | 0.129578755 | 1.034346142 |
| 1681 | 5 | 31.7 | Marker445932 | 0.782286876 | -0.044230769 | 0.129578755 | 1.034346142 |
| 1682 | 5 | 31.7 | Marker445640 | 0.782286876 | -0.044230769 | 0.129578755 | 1.034346142 |
| 1683 | 5 | 31.7 | Marker445641 | 0.782286876 | -0.044230769 | 0.129578755 | 1.034346142 |
| 1684 | 5 | 31.95 | Marker445965 | 0.792930213 | -0.034803922 | 0.14727155 | 1.195493353 |
| 1685 | 5 | 31.95 | Marker445964 | 0.792930213 | -0.034803922 | 0.14727155 | 1.195493353 |
| 1686 | 5 | 31.95 | Marker445969 | 0.792930213 | -0.034803922 | 0.14727155 | 1.195493353 |
| 1687 | 5 | 31.95 | Marker445968 | 0.792930213 | -0.034803922 | 0.14727155 | 1.195493353 |
| 1688 | 5 | 33.5 | Marker447217 | 0.334681239 | 7.36E-16 | 0.125 | 0.771995723 |
| 1689 | 5 | 33.5 | Marker447219 | 0.334681239 | 7.36E-16 | 0.125 | 0.771995723 |
| 1690 | 5 | 33.5 | Marker447214 | 0.334681239 | 7.36E-16 | 0.125 | 0.771995723 |
| 1691 | 5 | 33.5 | Marker447218 | 0.334681239 | 7.36E-16 | 0.125 | 0.771995723 |
| 1692 | 5 | 35.301 | Marker449388 | 0.067953092 | 0.012195122 | 0.101012425 | 0.522749262 |
| 1693 | 5 | 35.301 | Marker449389 | 0.067953092 | 0.012195122 | 0.101012425 | 0.522749262 |
| 1694 | 5 | 36.551 | Marker448103 | 0.270788399 | -0.009177865 | 0.100126299 | 0.502912136 |
| 1695 | 5 | 37.552 | Marker449071 | 0.058999139 | -0.008456083 | 0.055992982 | 0.161904644 |
| 1696 | 5 | 37.552 | Marker449464 | 0.058999139 | -0.008456083 | 0.055992982 | 0.161904644 |
| 1697 | 5 | 37.552 | Marker449580 | 0.058999139 | -0.008456083 | 0.055992982 | 0.161904644 |
| 1698 | 5 | 37.552 | Marker449465 | 0.058999139 | -0.008456083 | 0.055992982 | 0.161904644 |
| 1699 | 5 | 37.552 | Marker449460 | 0.058999139 | -0.008456083 | 0.055992982 | 0.161904644 |
| 1700 | 5 | 38.852 | Marker450099 | 0.012460489 | 0.036594523 | 0.016052306 | 0.161145377 |
| 1701 | 5 | 39.852 | Marker451404 | 0.002900818 | 0.025396825 | -0.006204906 | 0.072579012 |
| 1702 | 5 | 41.403 | Marker453006 | 0.019412412 | 0.058730159 | 0.054401154 | 0.531565185 |
| 1703 | 5 | 41.403 | Marker453011 | 0.019412412 | 0.058730159 | 0.054401154 | 0.531565185 |
| 1704 | 5 | 41.403 | Marker453584 | 0.019412412 | 0.058730159 | 0.054401154 | 0.531565185 |
| 1705 | 5 | 41.403 | Marker453012 | 0.019412412 | 0.058730159 | 0.054401154 | 0.531565185 |
| 1706 | 5 | 41.403 | Marker453007 | 0.019412412 | 0.058730159 | 0.054401154 | 0.531565185 |
| 1707 | 5 | 41.403 | Marker453008 | 0.019412412 | 0.058730159 | 0.054401154 | 0.531565185 |
| 1708 | 5 | 41.653 | Marker452324 | 0.020368857 | 0.059891599 | 0.052420713 | 0.536169526 |
| 1709 | 5 | 41.653 | Marker453146 | 0.020368857 | 0.059891599 | 0.052420713 | 0.536169526 |
| 1710 | 5 | 41.653 | Marker453147 | 0.020368857 | 0.059891599 | 0.052420713 | 0.536169526 |
| 1711 | 5 | 41.653 | Marker453145 | 0.020368857 | 0.059891599 | 0.052420713 | 0.536169526 |
| 1712 | 5 | 42.653 | Marker455840 | 0.05211411 | 0.07173913 | 0.07083823 | 0.823467291 |
| 1713 | 5 | 45.506 | Marker460890 | 0.416075968 | 0.099767442 | 0.056386404 | 1.262507917 |
| 1714 | 5 | 45.506 | Marker460891 | 0.416075968 | 0.099767442 | 0.056386404 | 1.262507917 |
| 1715 | 5 | 45.756 | Marker460893 | 0.416418796 | 0.098181818 | 0.05890556 | 1.242613347 |
| 1716 | 5 | 46.756 | Marker463171 | 0.359359723 | 0.110795455 | 0.037418831 | 1.427376066 |
| 1717 | 5 | 48.006 | Marker464525 | 0.251919298 | 0.125508958 | 0.050788838 | 1.872260205 |
| 1718 | 5 | 48.607 | Marker465178 | 0.387194428 | 0.13236715 | 0.039495032 | 2.014367406 |
| 1719 | 5 | 48.607 | Marker465176 | 0.387194428 | 0.13236715 | 0.039495032 | 2.014367406 |
| 1720 | 5 | 48.857 | Marker466319 | 0.189462505 | 0.122222222 | 0.0192108 | 1.666094493 |
| 1721 | 5 | 48.857 | Marker466446 | 0.189462505 | 0.122222222 | 0.0192108 | 1.666094493 |
| 1722 | 5 | 48.857 | Marker466355 | 0.189462505 | 0.122222222 | 0.0192108 | 1.666094493 |
| 1723 | 5 | 48.857 | Marker466354 | 0.189462505 | 0.122222222 | 0.0192108 | 1.666094493 |
| 1724 | 5 | 48.857 | Marker466313 | 0.189462505 | 0.122222222 | 0.0192108 | 1.666094493 |
| 1725 | 5 | 48.857 | Marker466318 | 0.189462505 | 0.122222222 | 0.0192108 | 1.666094493 |
| 1726 | 5 | 49.107 | Marker468027 | 0.188501738 | 0.120289855 | 0.02184851 | 1.620398598 |
| 1727 | 5 | 49.107 | Marker467863 | 0.188501738 | 0.120289855 | 0.02184851 | 1.620398598 |
| 1728 | 5 | 49.107 | Marker468271 | 0.188501738 | 0.120289855 | 0.02184851 | 1.620398598 |
| 1729 | 5 | 49.107 | Marker467862 | 0.188501738 | 0.120289855 | 0.02184851 | 1.620398598 |
| 1730 | 5 | 49.107 | Marker469210 | 0.188501738 | 0.120289855 | 0.02184851 | 1.620398598 |
| 1731 | 5 | 49.107 | Marker468359 | 0.188501738 | 0.120289855 | 0.02184851 | 1.620398598 |
| 1732 | 5 | 49.107 | Marker467516 | 0.188501738 | 0.120289855 | 0.02184851 | 1.620398598 |
| 1733 | 5 | 50.908 | Marker467572 | 0.146742458 | 0.060492282 | 0.093172021 | 0.841834935 |
| 1734 | 5 | 50.908 | Marker467576 | 0.146742458 | 0.060492282 | 0.093172021 | 0.841834935 |
| 1735 | 5 | 50.908 | Marker467571 | 0.146742458 | 0.060492282 | 0.093172021 | 0.841834935 |
| 1736 | 5 | 50.908 | Marker467570 | 0.146742458 | 0.060492282 | 0.093172021 | 0.841834935 |
| 1737 | 5 | 51.658 | Marker463624 | 0.16073324 | 0.071914894 | 0.111914894 | 1.202571988 |
| 1738 | 5 | 51.658 | Marker463623 | 0.16073324 | 0.071914894 | 0.111914894 | 1.202571988 |
| 1739 | 5 | 53.209 | Marker473887 | 0.182576904 | 0.106944444 | 0.087713675 | 1.655646648 |
| 1740 | 5 | 53.209 | Marker473888 | 0.182576904 | 0.106944444 | 0.087713675 | 1.655646648 |
| 1741 | 5 | 53.459 | Marker484650 | 0.18205368 | 0.105668934 | 0.090016071 | 1.64626759 |
| 1742 | 5 | 53.459 | Marker473298 | 0.18205368 | 0.105668934 | 0.090016071 | 1.64626759 |
| 1743 | 5 | 53.459 | Marker484656 | 0.18205368 | 0.105668934 | 0.090016071 | 1.64626759 |
| 1744 | 5 | 53.459 | Marker484649 | 0.18205368 | 0.105668934 | 0.090016071 | 1.64626759 |
| 1745 | 5 | 53.459 | Marker473297 | 0.18205368 | 0.105668934 | 0.090016071 | 1.64626759 |
| 1746 | 5 | 53.709 | Marker469352 | 0.140144284 | 0.117361111 | 0.067681624 | 1.756287988 |
| 1747 | 5 | 54.459 | Marker471024 | 0.139960606 | 0.111111111 | 0.080581516 | 1.695524452 |
| 1748 | 5 | 54.459 | Marker470035 | 0.139960606 | 0.111111111 | 0.080581516 | 1.695524452 |
| 1749 | 5 | 54.459 | Marker470418 | 0.139960606 | 0.111111111 | 0.080581516 | 1.695524452 |
| 1750 | 5 | 54.459 | Marker471569 | 0.139960606 | 0.111111111 | 0.080581516 | 1.695524452 |
| 1751 | 5 | 54.459 | Marker473923 | 0.139960606 | 0.111111111 | 0.080581516 | 1.695524452 |
| 1752 | 5 | 54.459 | Marker473087 | 0.139960606 | 0.111111111 | 0.080581516 | 1.695524452 |
| 1753 | 5 | 54.459 | Marker473654 | 0.139960606 | 0.111111111 | 0.080581516 | 1.695524452 |
| 1754 | 5 | 54.459 | Marker470036 | 0.139960606 | 0.111111111 | 0.080581516 | 1.695524452 |
| 1755 | 5 | 54.459 | Marker470038 | 0.139960606 | 0.111111111 | 0.080581516 | 1.695524452 |
| 1756 | 5 | 54.459 | Marker473920 | 0.139960606 | 0.111111111 | 0.080581516 | 1.695524452 |
| 1757 | 5 | 54.459 | Marker469919 | 0.139960606 | 0.111111111 | 0.080581516 | 1.695524452 |
| 1758 | 5 | 54.459 | Marker473088 | 0.139960606 | 0.111111111 | 0.080581516 | 1.695524452 |
| 1759 | 5 | 54.459 | Marker470417 | 0.139960606 | 0.111111111 | 0.080581516 | 1.695524452 |
| 1760 | 5 | 54.459 | Marker470416 | 0.139960606 | 0.111111111 | 0.080581516 | 1.695524452 |
| 1761 | 5 | 54.459 | Marker470037 | 0.139960606 | 0.111111111 | 0.080581516 | 1.695524452 |
| 1762 | 5 | 54.459 | Marker472594 | 0.139960606 | 0.111111111 | 0.080581516 | 1.695524452 |
| 1763 | 5 | 54.459 | Marker471762 | 0.139960606 | 0.111111111 | 0.080581516 | 1.695524452 |
| 1764 | 5 | 54.459 | Marker473919 | 0.139960606 | 0.111111111 | 0.080581516 | 1.695524452 |
| 1765 | 5 | 54.459 | Marker471023 | 0.139960606 | 0.111111111 | 0.080581516 | 1.695524452 |
| 1766 | 5 | 54.459 | Marker473097 | 0.139960606 | 0.111111111 | 0.080581516 | 1.695524452 |
| 1767 | 5 | 54.459 | Marker473924 | 0.139960606 | 0.111111111 | 0.080581516 | 1.695524452 |
| 1768 | 5 | 54.459 | Marker473921 | 0.139960606 | 0.111111111 | 0.080581516 | 1.695524452 |
| 1769 | 5 | 54.459 | Marker471568 | 0.139960606 | 0.111111111 | 0.080581516 | 1.695524452 |
| 1770 | 5 | 54.709 | Marker473690 | 0.155965471 | 0.123989899 | 0.057491582 | 1.867799401 |
| 1771 | 5 | 54.709 | Marker473266 | 0.155965471 | 0.123989899 | 0.057491582 | 1.867799401 |
| 1772 | 5 | 54.709 | Marker473264 | 0.155965471 | 0.123989899 | 0.057491582 | 1.867799401 |
| 1773 | 5 | 54.709 | Marker473689 | 0.155965471 | 0.123989899 | 0.057491582 | 1.867799401 |
| 1774 | 5 | 54.709 | Marker473688 | 0.155965471 | 0.123989899 | 0.057491582 | 1.867799401 |
| 1775 | 5 | 54.709 | Marker473691 | 0.155965471 | 0.123989899 | 0.057491582 | 1.867799401 |
| 1776 | 5 | 54.709 | Marker473265 | 0.155965471 | 0.123989899 | 0.057491582 | 1.867799401 |
| 1777 | 5 | 55.209 | Marker470952 | 0.134913019 | 0.098792271 | 0.103304166 | 1.619763998 |
| 1778 | 5 | 55.209 | Marker470951 | 0.134913019 | 0.098792271 | 0.103304166 | 1.619763998 |
| 1779 | 5 | 55.459 | Marker480012 | 0.134936597 | 0.1 | 0.101038422 | 1.623117798 |
| 1780 | 5 | 55.459 | Marker480932 | 0.134936597 | 0.1 | 0.101038422 | 1.623117798 |
| 1781 | 5 | 55.459 | Marker480931 | 0.134936597 | 0.1 | 0.101038422 | 1.623117798 |
| 1782 | 5 | 55.459 | Marker480933 | 0.134936597 | 0.1 | 0.101038422 | 1.623117798 |
| 1783 | 5 | 55.959 | Marker477980 | 0.152724724 | 0.111660079 | 0.078100181 | 1.689099738 |
| 1784 | 5 | 55.959 | Marker482169 | 0.152724724 | 0.111660079 | 0.078100181 | 1.689099738 |
| 1785 | 5 | 55.959 | Marker482019 | 0.152724724 | 0.111660079 | 0.078100181 | 1.689099738 |
| 1786 | 5 | 55.959 | Marker482020 | 0.152724724 | 0.111660079 | 0.078100181 | 1.689099738 |
| 1787 | 5 | 55.959 | Marker482023 | 0.152724724 | 0.111660079 | 0.078100181 | 1.689099738 |
| 1788 | 5 | 55.959 | Marker482024 | 0.152724724 | 0.111660079 | 0.078100181 | 1.689099738 |
| 1789 | 5 | 55.959 | Marker482168 | 0.152724724 | 0.111660079 | 0.078100181 | 1.689099738 |
| 1790 | 5 | 55.959 | Marker482018 | 0.152724724 | 0.111660079 | 0.078100181 | 1.689099738 |
| 1791 | 5 | 56.209 | Marker477023 | 0.152608537 | 0.110144928 | 0.080585179 | 1.67189169 |
| 1792 | 5 | 56.459 | Marker478330 | 0.14860342 | 0.109219858 | 0.08064843 | 1.649944934 |
| 1793 | 5 | 56.459 | Marker485400 | 0.14860342 | 0.109219858 | 0.08064843 | 1.649944934 |
| 1794 | 5 | 56.459 | Marker478324 | 0.14860342 | 0.109219858 | 0.08064843 | 1.649944934 |
| 1795 | 5 | 56.459 | Marker478466 | 0.14860342 | 0.109219858 | 0.08064843 | 1.649944934 |
| 1796 | 5 | 56.459 | Marker478465 | 0.14860342 | 0.109219858 | 0.08064843 | 1.649944934 |
| 1797 | 5 | 56.459 | Marker485372 | 0.14860342 | 0.109219858 | 0.08064843 | 1.649944934 |
| 1798 | 5 | 56.459 | Marker485246 | 0.14860342 | 0.109219858 | 0.08064843 | 1.649944934 |
| 1799 | 5 | 56.459 | Marker485247 | 0.14860342 | 0.109219858 | 0.08064843 | 1.649944934 |
| 1800 | 5 | 56.459 | Marker485769 | 0.14860342 | 0.109219858 | 0.08064843 | 1.649944934 |
| 1801 | 5 | 56.709 | Marker477052 | 0.157614257 | 0.111897261 | 0.091880378 | 1.813520139 |
| 1802 | 5 | 57.209 | Marker481844 | 0.179979917 | 0.119858156 | 0.100810537 | 2.104827704 |
| 1803 | 5 | 57.209 | Marker481843 | 0.179979917 | 0.119858156 | 0.100810537 | 2.104827704 |
| 1804 | 5 | 57.209 | Marker476295 | 0.179979917 | 0.119858156 | 0.100810537 | 2.104827704 |
| 1805 | 5 | 57.209 | Marker476296 | 0.179979917 | 0.119858156 | 0.100810537 | 2.104827704 |
| 1806 | 5 | 57.709 | Marker482316 | 0.23042784 | 0.106382979 | 0.126213592 | 2.056804206 |
| 1807 | 5 | 57.709 | Marker482319 | 0.23042784 | 0.106382979 | 0.126213592 | 2.056804206 |
| 1808 | 5 | 58.209 | Marker479612 | 0.194598059 | 0.097826087 | 0.103416149 | 1.599844015 |
| 1809 | 5 | 58.209 | Marker479613 | 0.194598059 | 0.097826087 | 0.103416149 | 1.599844015 |
| 1810 | 5 | 58.209 | Marker479614 | 0.194598059 | 0.097826087 | 0.103416149 | 1.599844015 |
| 1811 | 5 | 58.459 | Marker480119 | 0.178369466 | 0.098792271 | 0.103304166 | 1.619763998 |
| 1812 | 5 | 58.959 | Marker479230 | 0.140063124 | 0.112626263 | 0.078114478 | 1.713193445 |
| 1813 | 5 | 58.959 | Marker478943 | 0.140063124 | 0.112626263 | 0.078114478 | 1.713193445 |
| 1814 | 5 | 58.959 | Marker480860 | 0.140063124 | 0.112626263 | 0.078114478 | 1.713193445 |
| 1815 | 5 | 58.959 | Marker479566 | 0.140063124 | 0.112626263 | 0.078114478 | 1.713193445 |
| 1816 | 5 | 58.959 | Marker474568 | 0.140063124 | 0.112626263 | 0.078114478 | 1.713193445 |
| 1817 | 5 | 58.959 | Marker478947 | 0.140063124 | 0.112626263 | 0.078114478 | 1.713193445 |
| 1818 | 5 | 58.959 | Marker478648 | 0.140063124 | 0.112626263 | 0.078114478 | 1.713193445 |
| 1819 | 5 | 58.959 | Marker475856 | 0.140063124 | 0.112626263 | 0.078114478 | 1.713193445 |
| 1820 | 5 | 58.959 | Marker476200 | 0.140063124 | 0.112626263 | 0.078114478 | 1.713193445 |
| 1821 | 5 | 58.959 | Marker478946 | 0.140063124 | 0.112626263 | 0.078114478 | 1.713193445 |
| 1822 | 5 | 58.959 | Marker478942 | 0.140063124 | 0.112626263 | 0.078114478 | 1.713193445 |
| 1823 | 5 | 58.959 | Marker476322 | 0.140063124 | 0.112626263 | 0.078114478 | 1.713193445 |
| 1824 | 5 | 58.959 | Marker476329 | 0.140063124 | 0.112626263 | 0.078114478 | 1.713193445 |
| 1825 | 5 | 58.959 | Marker476201 | 0.140063124 | 0.112626263 | 0.078114478 | 1.713193445 |
| 1826 | 5 | 58.959 | Marker474529 | 0.140063124 | 0.112626263 | 0.078114478 | 1.713193445 |
| 1827 | 5 | 59.459 | Marker480099 | 0.159570262 | 0.121256039 | 0.060040106 | 1.809109104 |
| 1828 | 5 | 59.459 | Marker480098 | 0.159570262 | 0.121256039 | 0.060040106 | 1.809109104 |
| 1829 | 5 | 59.709 | Marker484498 | 0.202113963 | 0.136209622 | 0.054217532 | 2.200026037 |
| 1830 | 5 | 59.709 | Marker484499 | 0.202113963 | 0.136209622 | 0.054217532 | 2.200026037 |
| 1831 | 5 | 60.259 | Marker480954 | 0.218199692 | 0.135416667 | 0.067759901 | 2.261220275 |
| 1832 | 5 | 60.259 | Marker461482 | 0.218199692 | 0.135416667 | 0.067759901 | 2.261220275 |
| 1833 | 5 | 60.259 | Marker480952 | 0.218199692 | 0.135416667 | 0.067759901 | 2.261220275 |
| 1834 | 5 | 60.259 | Marker461481 | 0.218199692 | 0.135416667 | 0.067759901 | 2.261220275 |
| 1835 | 5 | 60.259 | Marker480951 | 0.218199692 | 0.135416667 | 0.067759901 | 2.261220275 |
| 1836 | 5 | 60.259 | Marker480955 | 0.218199692 | 0.135416667 | 0.067759901 | 2.261220275 |
| 1837 | 5 | 60.509 | Marker480731 | 0.190544208 | 0.12363624 | 0.078655771 | 2.005280912 |
| 1838 | 5 | 60.509 | Marker480730 | 0.190544208 | 0.12363624 | 0.078655771 | 2.005280912 |
| 1839 | 5 | 61.117 | Marker483261 | 0.111550476 | 0.096426687 | 0.061773261 | 1.222448662 |
| 1840 | 5 | 61.367 | Marker482515 | 0.110638942 | 0.096065548 | 0.062376281 | 1.218564281 |
| 1841 | 5 | 61.867 | Marker483639 | 0.110746816 | 0.095829636 | 0.063434069 | 1.220317259 |
| 1842 | 5 | 61.867 | Marker482749 | 0.110746816 | 0.095829636 | 0.063434069 | 1.220317259 |
| 1843 | 5 | 62.367 | Marker483005 | 0.159555583 | 0.104433078 | 0.083998295 | 1.564606932 |
| 1844 | 5 | 62.618 | Marker483006 | 0.15248312 | 0.105128205 | 0.081794872 | 1.562267985 |
| 1845 | 5 | 62.618 | Marker483012 | 0.15248312 | 0.105128205 | 0.081794872 | 1.562267985 |
| 1846 | 5 | 63.118 | Marker484326 | 0.211631447 | 0.114236999 | 0.103802217 | 1.989881788 |
| 1847 | 5 | 63.118 | Marker484323 | 0.211631447 | 0.114236999 | 0.103802217 | 1.989881788 |
| 1848 | 5 | 63.118 | Marker484325 | 0.211631447 | 0.114236999 | 0.103802217 | 1.989881788 |
| 1849 | 5 | 64.118 | Marker485026 | 0.188916105 | 0.107956159 | 0.100925796 | 1.805480474 |
| 1850 | 5 | 65.919 | Marker487963 | 0.122153447 | 0.094442032 | 0.065792359 | 1.206572272 |
| 1851 | 5 | 65.919 | Marker487808 | 0.122153447 | 0.094442032 | 0.065792359 | 1.206572272 |
| 1852 | 5 | 66.669 | Marker487640 | 0.196726824 | 0.117568767 | 0.022087096 | 1.549647939 |
| 1853 | 5 | 66.669 | Marker487641 | 0.196726824 | 0.117568767 | 0.022087096 | 1.549647939 |
| 1854 | 5 | 66.919 | Marker487642 | 0.123856824 | 0.10508033 | 0.045253071 | 1.324748647 |
| 1855 | 5 | 67.419 | Marker487954 | 0.189128071 | 0.096467391 | 0.063119987 | 1.2318651 |
| 1856 | 5 | 67.669 | Marker491169 | 0.188582013 | 0.095079787 | 0.065459428 | 1.217730524 |
| 1857 | 5 | 68.419 | Marker490614 | 0.208924367 | 0.0925 | 0.068510101 | 1.184888464 |
| 1858 | 5 | 68.419 | Marker490613 | 0.208924367 | 0.0925 | 0.068510101 | 1.184888464 |
| 1859 | 5 | 68.419 | Marker490612 | 0.208924367 | 0.0925 | 0.068510101 | 1.184888464 |
| 1860 | 5 | 68.419 | Marker490611 | 0.208924367 | 0.0925 | 0.068510101 | 1.184888464 |
| 1861 | 5 | 68.919 | Marker491933 | 0.212789692 | 0.093241552 | 0.066592078 | 1.187005527 |
| 1862 | 5 | 68.919 | Marker491931 | 0.212789692 | 0.093241552 | 0.066592078 | 1.187005527 |
| 1863 | 5 | 69.169 | Marker491932 | 0.216048535 | 0.093829787 | 0.066170213 | 1.196344775 |
| 1864 | 5 | 69.919 | Marker492459 | 0.195700237 | 0.096467391 | 0.063119987 | 1.2318651 |
| 1865 | 5 | 69.919 | Marker491293 | 0.195700237 | 0.096467391 | 0.063119987 | 1.2318651 |
| 1866 | 5 | 69.919 | Marker491269 | 0.195700237 | 0.096467391 | 0.063119987 | 1.2318651 |
| 1867 | 5 | 69.919 | Marker491477 | 0.195700237 | 0.096467391 | 0.063119987 | 1.2318651 |
| 1868 | 5 | 69.919 | Marker491322 | 0.195700237 | 0.096467391 | 0.063119987 | 1.2318651 |
| 1869 | 5 | 70.419 | Marker491116 | 0.140693885 | 0.104166667 | 0.047442244 | 1.314081925 |
| 1870 | 5 | 70.419 | Marker491115 | 0.140693885 | 0.104166667 | 0.047442244 | 1.314081925 |
| 1871 | 5 | 70.669 | Marker492706 | 0.144528969 | 0.10483156 | 0.047233521 | 1.328412327 |
| 1872 | 5 | 70.669 | Marker492709 | 0.144528969 | 0.10483156 | 0.047233521 | 1.328412327 |
| 1873 | 5 | 70.669 | Marker492711 | 0.144528969 | 0.10483156 | 0.047233521 | 1.328412327 |
| 1874 | 5 | 70.669 | Marker492704 | 0.144528969 | 0.10483156 | 0.047233521 | 1.328412327 |
| 1875 | 5 | 70.669 | Marker492705 | 0.144528969 | 0.10483156 | 0.047233521 | 1.328412327 |
| 1876 | 5 | 70.919 | Marker492606 | 0.210835311 | 0.095744681 | 0.065172485 | 1.229867479 |
| 1877 | 5 | 71.169 | Marker491067 | 0.199307695 | 0.097132285 | 0.062851348 | 1.244384752 |
| 1878 | 5 | 71.169 | Marker491069 | 0.199307695 | 0.097132285 | 0.062851348 | 1.244384752 |
| 1879 | 5 | 71.169 | Marker491068 | 0.199307695 | 0.097132285 | 0.062851348 | 1.244384752 |
| 1880 | 5 | 71.669 | Marker491181 | 0.151399956 | 0.097916667 | 0.060737179 | 1.248019388 |
| 1881 | 5 | 71.669 | Marker491180 | 0.151399956 | 0.097916667 | 0.060737179 | 1.248019388 |
| 1882 | 5 | 71.669 | Marker491297 | 0.151399956 | 0.097916667 | 0.060737179 | 1.248019388 |
| 1883 | 5 | 71.669 | Marker491182 | 0.151399956 | 0.097916667 | 0.060737179 | 1.248019388 |
| 1884 | 5 | 71.919 | Marker491300 | 0.161975234 | 0.085597826 | 0.08369829 | 1.165373521 |
| 1885 | 5 | 71.919 | Marker491299 | 0.161975234 | 0.085597826 | 0.08369829 | 1.165373521 |
| 1886 | 5 | 72.419 | Marker492588 | 0.204358786 | 0.080704293 | 0.079119622 | 1.037577085 |
| 1887 | 5 | 72.419 | Marker493417 | 0.204358786 | 0.080704293 | 0.079119622 | 1.037577085 |
| 1888 | 5 | 72.919 | Marker492581 | 0.385038261 | 0.094007816 | 0.106436718 | 1.550369909 |
| 1889 | 5 | 73.669 | Marker492585 | 0.359388421 | 0.082037643 | 0.087899562 | 1.135414861 |
| 1890 | 5 | 75.47 | Marker494336 | 0.312738982 | 0.081495098 | 0.089573329 | 1.140491949 |
| 1891 | 5 | 75.47 | Marker494340 | 0.312738982 | 0.081495098 | 0.089573329 | 1.140491949 |
| 1892 | 5 | 75.47 | Marker494335 | 0.312738982 | 0.081495098 | 0.089573329 | 1.140491949 |
| 1893 | 5 | 75.47 | Marker494339 | 0.312738982 | 0.081495098 | 0.089573329 | 1.140491949 |
| 1894 | 5 | 75.47 | Marker494334 | 0.312738982 | 0.081495098 | 0.089573329 | 1.140491949 |
| 1895 | 5 | 76.72 | Marker494967 | 0.216251831 | 0.095561594 | 0.107899782 | 1.598829399 |
| 1896 | 5 | 76.72 | Marker495296 | 0.216251831 | 0.095561594 | 0.107899782 | 1.598829399 |
| 1897 | 5 | 76.97 | Marker494823 | 0.211918072 | 0.094636525 | 0.108116917 | 1.581643531 |
| 1898 | 5 | 76.97 | Marker495478 | 0.211918072 | 0.094636525 | 0.108116917 | 1.581643531 |
| 1899 | 5 | 78.221 | Marker496157 | 0.26688313 | 0.095744681 | 0.146560628 | 2.095548912 |
| 1900 | 5 | 78.221 | Marker496153 | 0.26688313 | 0.095744681 | 0.146560628 | 2.095548912 |
| 1901 | 5 | 78.221 | Marker496154 | 0.26688313 | 0.095744681 | 0.146560628 | 2.095548912 |
| 1902 | 5 | 78.471 | Marker496355 | 0.300929661 | 0.084441489 | 0.169000313 | 2.22176242 |
| 1903 | 5 | 78.471 | Marker496357 | 0.300929661 | 0.084441489 | 0.169000313 | 2.22176242 |
| 1904 | 5 | 78.471 | Marker496737 | 0.300929661 | 0.084441489 | 0.169000313 | 2.22176242 |
| 1905 | 5 | 78.471 | Marker496616 | 0.300929661 | 0.084441489 | 0.169000313 | 2.22176242 |
| 1906 | 5 | 78.471 | Marker496427 | 0.300929661 | 0.084441489 | 0.169000313 | 2.22176242 |
| 1907 | 5 | 78.471 | Marker496610 | 0.300929661 | 0.084441489 | 0.169000313 | 2.22176242 |
| 1908 | 5 | 78.471 | Marker496808 | 0.300929661 | 0.084441489 | 0.169000313 | 2.22176242 |
| 1909 | 5 | 78.471 | Marker496358 | 0.300929661 | 0.084441489 | 0.169000313 | 2.22176242 |
| 1910 | 5 | 78.471 | Marker496806 | 0.300929661 | 0.084441489 | 0.169000313 | 2.22176242 |
| 1911 | 5 | 78.471 | Marker496353 | 0.300929661 | 0.084441489 | 0.169000313 | 2.22176242 |
| 1912 | 5 | 78.471 | Marker496430 | 0.300929661 | 0.084441489 | 0.169000313 | 2.22176242 |
| 1913 | 5 | 78.471 | Marker496807 | 0.300929661 | 0.084441489 | 0.169000313 | 2.22176242 |
| 1914 | 5 | 78.471 | Marker496354 | 0.300929661 | 0.084441489 | 0.169000313 | 2.22176242 |
| 1915 | 5 | 78.721 | Marker498492 | 0.34807616 | 0.074468085 | 0.187254699 | 2.368101586 |
| 1916 | 5 | 78.721 | Marker497037 | 0.34807616 | 0.074468085 | 0.187254699 | 2.368101586 |
| 1917 | 5 | 78.721 | Marker498507 | 0.34807616 | 0.074468085 | 0.187254699 | 2.368101586 |
| 1918 | 5 | 78.721 | Marker497036 | 0.34807616 | 0.074468085 | 0.187254699 | 2.368101586 |
| 1919 | 5 | 78.971 | Marker499001 | 0.325542715 | 0.084441489 | 0.169000313 | 2.22176242 |
| 1920 | 5 | 78.971 | Marker498925 | 0.325542715 | 0.084441489 | 0.169000313 | 2.22176242 |
| 1921 | 5 | 79.221 | Marker499764 | 0.357983578 | 0.073599653 | 0.191199168 | 2.427943474 |
| 1922 | 5 | 79.221 | Marker500949 | 0.357983578 | 0.073599653 | 0.191199168 | 2.427943474 |
| 1923 | 5 | 79.221 | Marker500389 | 0.357983578 | 0.073599653 | 0.191199168 | 2.427943474 |
| 1924 | 5 | 79.221 | Marker499042 | 0.357983578 | 0.073599653 | 0.191199168 | 2.427943474 |
| 1925 | 5 | 79.221 | Marker499786 | 0.357983578 | 0.073599653 | 0.191199168 | 2.427943474 |
| 1926 | 5 | 79.221 | Marker499734 | 0.357983578 | 0.073599653 | 0.191199168 | 2.427943474 |
| 1927 | 5 | 79.221 | Marker499870 | 0.357983578 | 0.073599653 | 0.191199168 | 2.427943474 |
| 1928 | 5 | 79.221 | Marker499788 | 0.357983578 | 0.073599653 | 0.191199168 | 2.427943474 |
| 1929 | 5 | 79.221 | Marker499733 | 0.357983578 | 0.073599653 | 0.191199168 | 2.427943474 |
| 1930 | 5 | 79.221 | Marker499787 | 0.357983578 | 0.073599653 | 0.191199168 | 2.427943474 |
| 1931 | 5 | 79.221 | Marker499872 | 0.357983578 | 0.073599653 | 0.191199168 | 2.427943474 |
| 1932 | 5 | 79.221 | Marker500525 | 0.357983578 | 0.073599653 | 0.191199168 | 2.427943474 |
| 1933 | 5 | 79.221 | Marker500390 | 0.357983578 | 0.073599653 | 0.191199168 | 2.427943474 |
| 1934 | 5 | 79.721 | Marker501484 | 0.326181762 | 0.063478261 | 0.171993112 | 1.924879112 |
| 1935 | 5 | 79.721 | Marker501437 | 0.326181762 | 0.063478261 | 0.171993112 | 1.924879112 |
| 1936 | 5 | 79.721 | Marker501483 | 0.326181762 | 0.063478261 | 0.171993112 | 1.924879112 |
| 1937 | 5 | 79.971 | Marker501633 | 0.345833367 | 0.062553191 | 0.172553191 | 1.921359016 |
| 1938 | 5 | 79.971 | Marker501727 | 0.345833367 | 0.062553191 | 0.172553191 | 1.921359016 |
| 1939 | 5 | 79.971 | Marker501614 | 0.345833367 | 0.062553191 | 0.172553191 | 1.921359016 |
| 1940 | 5 | 79.971 | Marker501613 | 0.345833367 | 0.062553191 | 0.172553191 | 1.921359016 |
| 1941 | 5 | 79.971 | Marker501350 | 0.345833367 | 0.062553191 | 0.172553191 | 1.921359016 |
| 1942 | 5 | 79.971 | Marker501634 | 0.345833367 | 0.062553191 | 0.172553191 | 1.921359016 |
| 1943 | 5 | 79.971 | Marker501632 | 0.345833367 | 0.062553191 | 0.172553191 | 1.921359016 |
| 1944 | 5 | 79.971 | Marker501615 | 0.345833367 | 0.062553191 | 0.172553191 | 1.921359016 |
| 1945 | 5 | 80.221 | Marker502197 | 0.346387936 | 0.062961355 | 0.17065988 | 1.894800718 |
| 1946 | 5 | 80.221 | Marker502182 | 0.346387936 | 0.062961355 | 0.17065988 | 1.894800718 |
| 1947 | 5 | 81.721 | Marker488254 | 0.363604283 | 0.06042417 | 0.175847659 | 1.948954417 |
| 1948 | 5 | 81.971 | Marker503353 | 0.363955182 | 0.060816327 | 0.173877551 | 1.920050725 |
| 1949 | 5 | 82.721 | Marker503144 | 0.220384198 | 0.059867275 | 0.174779041 | 1.92277532 |
| 1950 | 5 | 83.471 | Marker489096 | 0.234540333 | 0.059607843 | 0.176642157 | 1.951834449 |
| 1951 | 5 | 87.077 | Marker503665 | 0.410094415 | 0.050934574 | 0.232915012 | 2.987486548 |
| 1952 | 5 | 87.077 | Marker503667 | 0.410094415 | 0.050934574 | 0.232915012 | 2.987486548 |
| 1953 | 5 | 87.077 | Marker503673 | 0.410094415 | 0.050934574 | 0.232915012 | 2.987486548 |
| 1954 | 5 | 87.077 | Marker503672 | 0.410094415 | 0.050934574 | 0.232915012 | 2.987486548 |
| 1955 | 5 | 87.077 | Marker503666 | 0.410094415 | 0.050934574 | 0.232915012 | 2.987486548 |
| 1956 | 5 | 87.077 | Marker503668 | 0.410094415 | 0.050934574 | 0.232915012 | 2.987486548 |
| 1957 | 5 | 90.734 | Marker503367 | 0.1968749 | 0.081311212 | 0.166603473 | 2.123608719 |
| 1958 | 5 | 91.734 | Marker490297 | 0.158518169 | 0.080882353 | 0.130202081 | 1.576629469 |
| 1959 | 5 | 91.985 | Marker490096 | 0.162002911 | 0.080816327 | 0.131836735 | 1.596844269 |
| 1960 | 5 | 93.236 | Marker506533 | 0.254626733 | 0.081666667 | 0.132979798 | 1.627377568 |
| 1961 | 5 | 93.486 | Marker506532 | 0.257871157 | 0.082553191 | 0.132553191 | 1.637949835 |
| 1962 | 5 | 93.736 | Marker505964 | 0.176654204 | 0.081666667 | 0.132979798 | 1.627377568 |
| 1963 | 5 | 93.736 | Marker505966 | 0.176654204 | 0.081666667 | 0.132979798 | 1.627377568 |
| 1964 | 5 | 93.736 | Marker505654 | 0.176654204 | 0.081666667 | 0.132979798 | 1.627377568 |
| 1965 | 5 | 93.736 | Marker505655 | 0.176654204 | 0.081666667 | 0.132979798 | 1.627377568 |
| 1966 | 5 | 93.736 | Marker505965 | 0.176654204 | 0.081666667 | 0.132979798 | 1.627377568 |
| 1967 | 5 | 93.736 | Marker505656 | 0.176654204 | 0.081666667 | 0.132979798 | 1.627377568 |
| 1968 | 5 | 93.986 | Marker506324 | 0.204733132 | 0.072278912 | 0.151054422 | 1.722127119 |
| 1969 | 5 | 93.986 | Marker505855 | 0.204733132 | 0.072278912 | 0.151054422 | 1.722127119 |
| 1970 | 5 | 93.986 | Marker505797 | 0.204733132 | 0.072278912 | 0.151054422 | 1.722127119 |
| 1971 | 5 | 93.986 | Marker505064 | 0.204733132 | 0.072278912 | 0.151054422 | 1.722127119 |
| 1972 | 5 | 93.986 | Marker505798 | 0.204733132 | 0.072278912 | 0.151054422 | 1.722127119 |
| 1973 | 5 | 93.986 | Marker505063 | 0.204733132 | 0.072278912 | 0.151054422 | 1.722127119 |
| 1974 | 5 | 93.986 | Marker505650 | 0.204733132 | 0.072278912 | 0.151054422 | 1.722127119 |
| 1975 | 5 | 93.986 | Marker505725 | 0.204733132 | 0.072278912 | 0.151054422 | 1.722127119 |
| 1976 | 5 | 94.236 | Marker505065 | 0.26598676 | 0.0625 | 0.169348185 | 1.866103014 |
| 1977 | 5 | 94.236 | Marker505218 | 0.26598676 | 0.0625 | 0.169348185 | 1.866103014 |
| 1978 | 5 | 94.236 | Marker505217 | 0.26598676 | 0.0625 | 0.169348185 | 1.866103014 |
| 1979 | 5 | 94.236 | Marker505220 | 0.26598676 | 0.0625 | 0.169348185 | 1.866103014 |
| 1980 | 5 | 94.236 | Marker505219 | 0.26598676 | 0.0625 | 0.169348185 | 1.866103014 |
| 1981 | 5 | 94.986 | Marker503824 | 0.107123346 | 0.064523589 | 0.125693802 | 1.25349122 |
| 1982 | 5 | 94.986 | Marker504307 | 0.107123346 | 0.064523589 | 0.125693802 | 1.25349122 |
| 1983 | 5 | 94.986 | Marker504094 | 0.107123346 | 0.064523589 | 0.125693802 | 1.25349122 |
| 1984 | 5 | 95.236 | Marker508073 | 0.067754551 | 0.063858696 | 0.127572288 | 1.267672177 |
| 1985 | 5 | 95.236 | Marker508074 | 0.067754551 | 0.063858696 | 0.127572288 | 1.267672177 |
| 1986 | 5 | 95.486 | Marker508368 | 0.050637021 | 0.052526596 | 0.107673655 | 0.886723822 |
| 1987 | 5 | 95.486 | Marker508369 | 0.050637021 | 0.052526596 | 0.107673655 | 0.886723822 |
| 1988 | 5 | 95.986 | Marker508942 | 0.075721185 | 0.063164894 | 0.128115874 | 1.264743848 |
| 1989 | 5 | 95.986 | Marker509282 | 0.075721185 | 0.063164894 | 0.128115874 | 1.264743848 |
| 1990 | 5 | 95.986 | Marker509281 | 0.075721185 | 0.063164894 | 0.128115874 | 1.264743848 |
| 1991 | 5 | 95.986 | Marker508912 | 0.075721185 | 0.063164894 | 0.128115874 | 1.264743848 |
| 1992 | 5 | 95.986 | Marker509135 | 0.075721185 | 0.063164894 | 0.128115874 | 1.264743848 |
| 1993 | 5 | 95.986 | Marker508940 | 0.075721185 | 0.063164894 | 0.128115874 | 1.264743848 |
| 1994 | 5 | 95.986 | Marker509003 | 0.075721185 | 0.063164894 | 0.128115874 | 1.264743848 |
| 1995 | 5 | 95.986 | Marker508908 | 0.075721185 | 0.063164894 | 0.128115874 | 1.264743848 |
| 1996 | 5 | 95.986 | Marker509934 | 0.075721185 | 0.063164894 | 0.128115874 | 1.264743848 |
| 1997 | 5 | 96.236 | Marker509929 | 0.07762564 | 0.063858696 | 0.127572288 | 1.267672177 |
| 1998 | 5 | 96.236 | Marker509901 | 0.07762564 | 0.063858696 | 0.127572288 | 1.267672177 |
| 1999 | 5 | 96.736 | Marker510878 | 0.05717672 | 0.054137116 | 0.104593043 | 0.87332801 |
| 2000 | 5 | 96.736 | Marker510879 | 0.05717672 | 0.054137116 | 0.104593043 | 0.87332801 |
| 2001 | 5 | 97.236 | Marker510922 | 0.056790304 | 0.054347826 | 0.103416149 | 0.863671068 |
| 2002 | 5 | 97.236 | Marker511500 | 0.056790304 | 0.054347826 | 0.103416149 | 0.863671068 |
| 2003 | 5 | 97.236 | Marker511499 | 0.056790304 | 0.054347826 | 0.103416149 | 0.863671068 |
| 2004 | 5 | 97.236 | Marker510920 | 0.056790304 | 0.054347826 | 0.103416149 | 0.863671068 |
| 2005 | 5 | 97.236 | Marker510923 | 0.056790304 | 0.054347826 | 0.103416149 | 0.863671068 |
| 2006 | 5 | 97.236 | Marker510921 | 0.056790304 | 0.054347826 | 0.103416149 | 0.863671068 |
| 2007 | 5 | 97.486 | Marker512023 | 0.065815061 | 0.053654024 | 0.105208852 | 0.873950122 |
| 2008 | 5 | 97.986 | Marker512025 | 0.076273176 | 0.053654024 | 0.105208852 | 0.873950122 |
| 2009 | 5 | 97.986 | Marker512026 | 0.076273176 | 0.053654024 | 0.105208852 | 0.873950122 |
| 2010 | 5 | 97.986 | Marker512024 | 0.076273176 | 0.053654024 | 0.105208852 | 0.873950122 |
| 2011 | 5 | 98.236 | Marker511076 | 0.074937972 | 0.05298913 | 0.106993985 | 0.884909983 |
| 2012 | 5 | 98.236 | Marker511075 | 0.074937972 | 0.05298913 | 0.106993985 | 0.884909983 |
| 2013 | 5 | 98.986 | Marker512869 | 0.11771901 | 0.063683665 | 0.125342093 | 1.237046919 |
| 2014 | 5 | 98.986 | Marker512868 | 0.11771901 | 0.063683665 | 0.125342093 | 1.237046919 |
| 2015 | 5 | 99.736 | Marker512891 | 0.194331531 | 0.076570048 | 0.183069 | 2.326580183 |
| 2016 | 5 | 99.736 | Marker512890 | 0.194331531 | 0.076570048 | 0.183069 | 2.326580183 |
| 2017 | 5 | 99.986 | Marker513630 | 0.173126572 | 0.088438735 | 0.160443833 | 2.158370581 |
| 2018 | 5 | 100.236 | Marker513864 | 0.179035562 | 0.090065478 | 0.160349691 | 2.189289281 |
| 2019 | 5 | 100.486 | Marker514047 | 0.161231545 | 0.10332981 | 0.137579281 | 2.135934854 |
| 2020 | 5 | 100.486 | Marker514048 | 0.161231545 | 0.10332981 | 0.137579281 | 2.135934854 |
| 2021 | 5 | 100.736 | Marker513917 | 0.146428485 | 0.104651163 | 0.137754033 | 2.168914538 |
| 2022 | 5 | 100.736 | Marker513714 | 0.146428485 | 0.104651163 | 0.137754033 | 2.168914538 |
| 2023 | 5 | 100.736 | Marker515297 | 0.146428485 | 0.104651163 | 0.137754033 | 2.168914538 |
| 2024 | 5 | 100.736 | Marker513541 | 0.146428485 | 0.104651163 | 0.137754033 | 2.168914538 |
| 2025 | 5 | 100.736 | Marker513904 | 0.146428485 | 0.104651163 | 0.137754033 | 2.168914538 |
| 2026 | 5 | 100.736 | Marker513179 | 0.146428485 | 0.104651163 | 0.137754033 | 2.168914538 |
| 2027 | 5 | 100.736 | Marker514009 | 0.146428485 | 0.104651163 | 0.137754033 | 2.168914538 |
| 2028 | 5 | 100.736 | Marker515573 | 0.146428485 | 0.104651163 | 0.137754033 | 2.168914538 |
| 2029 | 5 | 100.986 | Marker514455 | 0.163507576 | 0.092230444 | 0.160412262 | 2.234349177 |
| 2030 | 5 | 101.236 | Marker515063 | 0.199714542 | 0.080361757 | 0.182706303 | 2.386710836 |
| 2031 | 5 | 101.236 | Marker514793 | 0.199714542 | 0.080361757 | 0.182706303 | 2.386710836 |
| 2032 | 5 | 101.236 | Marker515062 | 0.199714542 | 0.080361757 | 0.182706303 | 2.386710836 |
| 2033 | 5 | 101.486 | Marker515476 | 0.163507576 | 0.092230444 | 0.160412262 | 2.234349177 |
| 2034 | 5 | 101.486 | Marker515844 | 0.163507576 | 0.092230444 | 0.160412262 | 2.234349177 |
| 2035 | 5 | 101.486 | Marker516008 | 0.163507576 | 0.092230444 | 0.160412262 | 2.234349177 |
| 2036 | 5 | 101.486 | Marker515336 | 0.163507576 | 0.092230444 | 0.160412262 | 2.234349177 |
| 2037 | 5 | 101.486 | Marker516009 | 0.163507576 | 0.092230444 | 0.160412262 | 2.234349177 |
| 2038 | 5 | 101.486 | Marker516012 | 0.163507576 | 0.092230444 | 0.160412262 | 2.234349177 |
| 2039 | 5 | 101.736 | Marker515738 | 0.134508386 | 0.081709957 | 0.139654265 | 1.719070831 |
| 2040 | 5 | 101.736 | Marker515739 | 0.134508386 | 0.081709957 | 0.139654265 | 1.719070831 |
| 2041 | 5 | 101.986 | Marker515737 | 0.13327697 | 0.080952381 | 0.141558442 | 1.732057979 |
| 2042 | 5 | 102.486 | Marker516591 | 0.118833261 | 0.082871397 | 0.139689579 | 1.740891576 |
| 2043 | 5 | 102.736 | Marker516589 | 0.162771861 | 0.083664209 | 0.137790572 | 1.729295341 |
| 2044 | 5 | 102.986 | Marker516742 | 0.141844581 | 0.084883721 | 0.137923297 | 1.754046433 |
| 2045 | 5 | 103.736 | Marker517227 | 0.162408829 | 0.075358852 | 0.160339089 | 1.917135178 |
| 2046 | 5 | 103.736 | Marker517223 | 0.162408829 | 0.075358852 | 0.160339089 | 1.917135178 |
| 2047 | 5 | 103.986 | Marker517224 | 0.159222385 | 0.074009324 | 0.160133726 | 1.891281535 |
| 2048 | 5 | 104.986 | Marker518625 | 0.133926274 | 0.057936508 | 0.140764791 | 1.363234099 |
| 2049 | 5 | 104.986 | Marker518624 | 0.133926274 | 0.057936508 | 0.140764791 | 1.363234099 |
| 2050 | 5 | 105.236 | Marker517882 | 0.112642764 | 0.046612466 | 0.119285139 | 0.952245322 |
| 2051 | 5 | 105.486 | Marker517793 | 0.178483856 | 0.046031746 | 0.11976912 | 0.951956377 |
| 2052 | 5 | 105.486 | Marker517794 | 0.178483856 | 0.046031746 | 0.11976912 | 0.951956377 |
| 2053 | 5 | 106.236 | Marker519525 | 0.197521269 | 0.057861962 | 0.143749137 | 1.404532636 |
| 2054 | 5 | 106.236 | Marker519257 | 0.197521269 | 0.057861962 | 0.143749137 | 1.404532636 |
| 2055 | 5 | 106.236 | Marker519258 | 0.197521269 | 0.057861962 | 0.143749137 | 1.404532636 |
| 2056 | 5 | 106.236 | Marker519259 | 0.197521269 | 0.057861962 | 0.143749137 | 1.404532636 |
| 2057 | 5 | 106.236 | Marker519270 | 0.197521269 | 0.057861962 | 0.143749137 | 1.404532636 |
| 2058 | 5 | 106.236 | Marker519271 | 0.197521269 | 0.057861962 | 0.143749137 | 1.404532636 |
| 2059 | 5 | 108.037 | Marker519058 | 0.113286137 | 0.02254306 | 0.121636834 | 0.791943614 |
| 2060 | 5 | 108.037 | Marker519057 | 0.113286137 | 0.02254306 | 0.121636834 | 0.791943614 |
| 2061 | 5 | 108.037 | Marker519060 | 0.113286137 | 0.02254306 | 0.121636834 | 0.791943614 |
| 2062 | 5 | 110.639 | Marker516628 | 0.192758827 | 0.080952381 | 0.141558442 | 1.732057979 |
| 2063 | 5 | 110.639 | Marker516627 | 0.192758827 | 0.080952381 | 0.141558442 | 1.732057979 |
| 2064 | 5 | 111.139 | Marker516738 | 0.209448444 | 0.092831456 | 0.117180491 | 1.64648544 |
| 2065 | 5 | 111.689 | Marker516327 | 0.427242093 | 0.074444444 | 0.151699346 | 1.767418884 |
| 2066 | 5 | 111.689 | Marker516329 | 0.427242093 | 0.074444444 | 0.151699346 | 1.767418884 |
| 2067 | 5 | 111.689 | Marker516328 | 0.427242093 | 0.074444444 | 0.151699346 | 1.767418884 |
| 2068 | 5 | 111.689 | Marker516706 | 0.427242093 | 0.074444444 | 0.151699346 | 1.767418884 |
| 2069 | 5 | 112.789 | Marker517497 | 0.413385599 | 0.065453027 | 0.131119602 | 1.336491912 |
| 2070 | 5 | 112.789 | Marker517496 | 0.413385599 | 0.065453027 | 0.131119602 | 1.336491912 |
| 2071 | 5 | 114.791 | Marker518232 | 0.494193071 | 0.058676301 | 0.127004831 | 1.189396162 |
| 2072 | 5 | 115.041 | Marker518235 | 0.470951468 | 0.061099344 | 0.124980086 | 1.196456081 |
| 2073 | 5 | 115.891 | Marker519850 | 0.461690648 | 0.072283531 | 0.15721868 | 1.816905512 |
| 2074 | 5 | 115.891 | Marker519849 | 0.461690648 | 0.072283531 | 0.15721868 | 1.816905512 |
| 2075 | 5 | 116.391 | Marker519852 | 0.349471861 | 0.051563919 | 0.113326205 | 0.937763282 |
| 2076 | 5 | 116.891 | Marker519853 | 0.412840448 | 0.032750488 | 0.151613082 | 1.262809078 |
| 2077 | 5 | 117.991 | Marker519839 | 0.123278115 | 0.04508612 | 0.122903298 | 0.980106151 |
| 2078 | 5 | 117.991 | Marker519838 | 0.123278115 | 0.04508612 | 0.122903298 | 0.980106151 |
| 2079 | 5 | 117.991 | Marker520547 | 0.123278115 | 0.04508612 | 0.122903298 | 0.980106151 |
| 2080 | 5 | 118.241 | Marker519618 | 0.118246543 | 0.046612466 | 0.119285139 | 0.952245322 |
| 2081 | 5 | 118.241 | Marker519615 | 0.118246543 | 0.046612466 | 0.119285139 | 0.952245322 |
| 2082 | 5 | 118.491 | Marker519619 | 0.097711887 | 0.058481153 | 0.097442192 | 0.855839232 |
| 2083 | 5 | 118.491 | Marker519899 | 0.097711887 | 0.058481153 | 0.097442192 | 0.855839232 |
| 2084 | 5 | 118.491 | Marker519650 | 0.097711887 | 0.058481153 | 0.097442192 | 0.855839232 |
| 2085 | 5 | 119.491 | Marker520679 | 0.062727293 | 0.066734075 | 0.081629405 | 0.829173382 |
| 2086 | 5 | 120.791 | Marker521543 | 0.060380134 | 0.068710359 | 0.077801268 | 0.828283404 |
| 2087 | 5 | 121.041 | Marker521471 | 0.080386095 | 0.052992037 | 0.08782313 | 0.698556928 |
| 2088 | 5 | 121.291 | Marker521207 | 0.056910092 | 0.034883721 | 0.055206369 | 0.287991082 |
| 2089 | 5 | 121.791 | Marker524136 | 0.08795806 | 0.045994832 | 0.077144821 | 0.533292196 |
| 2090 | 5 | 122.041 | Marker523935 | 0.084727266 | 0.04520202 | 0.078872054 | 0.538682486 |
| 2091 | 5 | 122.041 | Marker523936 | 0.084727266 | 0.04520202 | 0.078872054 | 0.538682486 |
| 2092 | 5 | 122.041 | Marker523937 | 0.084727266 | 0.04520202 | 0.078872054 | 0.538682486 |
| 2093 | 5 | 122.791 | Marker524244 | 0.131377414 | 0.079328165 | 0.10053102 | 1.20630638 |
| 2094 | 5 | 122.791 | Marker524242 | 0.131377414 | 0.079328165 | 0.10053102 | 1.20630638 |
| 2095 | 5 | 123.591 | Marker524252 | 0.101744599 | 0.054642166 | 0.104019926 | 0.873504797 |
| 2096 | 5 | 124.642 | Marker527306 | 0.108897004 | 0.044949495 | 0.120875421 | 0.954130336 |
| 2097 | 5 | 124.642 | Marker527309 | 0.108897004 | 0.044949495 | 0.120875421 | 0.954130336 |
| 2098 | 5 | 125.192 | Marker525638 | 0.070945743 | 0.039115854 | 0.114015868 | 0.818784428 |
| 2099 | 5 | 125.192 | Marker525639 | 0.070945743 | 0.039115854 | 0.114015868 | 0.818784428 |
| 2100 | 5 | 125.492 | Marker525694 | 0.049847207 | 0.047615476 | 0.098050084 | 0.732989656 |
| 2101 | 5 | 125.792 | Marker528152 | 0.038661637 | 0.045226875 | 0.081859234 | 0.562902872 |
| 2102 | 5 | 125.792 | Marker525698 | 0.038661637 | 0.045226875 | 0.081859234 | 0.562902872 |
| 2103 | 5 | 125.792 | Marker528153 | 0.038661637 | 0.045226875 | 0.081859234 | 0.562902872 |
| 2104 | 5 | 126.092 | Marker528154 | 0.010476876 | 0.05792447 | 0.065310985 | 0.586828983 |
| 2105 | 5 | 126.092 | Marker528155 | 0.010476876 | 0.05792447 | 0.065310985 | 0.586828983 |
| 2106 | 5 | 127.592 | Marker524950 | 0.285989742 | 0.065232285 | 0.153905106 | 1.656860007 |
| 2107 | 5 | 127.592 | Marker524955 | 0.285989742 | 0.065232285 | 0.153905106 | 1.656860007 |
| 2108 | 5 | 127.592 | Marker524954 | 0.285989742 | 0.065232285 | 0.153905106 | 1.656860007 |
| 2109 | 5 | 127.592 | Marker524956 | 0.285989742 | 0.065232285 | 0.153905106 | 1.656860007 |
| 2110 | 5 | 128.192 | Marker526536 | 0.475388825 | 0.050574209 | 0.189050607 | 2.064747783 |
| 2111 | 5 | 128.192 | Marker526537 | 0.475388825 | 0.050574209 | 0.189050607 | 2.064747783 |
| 2112 | 5 | 129.542 | Marker526604 | 0.592446064 | 0.052083333 | 0.226840615 | 2.8624349 |
| 2113 | 5 | 130.643 | Marker526818 | 0.135327223 | 0.065809005 | 0.159484293 | 1.752396609 |
| 2114 | 5 | 130.643 | Marker526819 | 0.135327223 | 0.065809005 | 0.159484293 | 1.752396609 |
| 2115 | 5 | 131.143 | Marker528309 | 0.154712732 | 0.064922481 | 0.159920287 | 1.74631873 |
| 2116 | 5 | 132.143 | Marker529564 | 0.223065674 | 0.097635934 | 0.142080378 | 2.071601543 |
| 2117 | 5 | 132.143 | Marker529566 | 0.223065674 | 0.097635934 | 0.142080378 | 2.071601543 |
| 2118 | 5 | 132.643 | Marker528818 | 0.20169454 | 0.098598552 | 0.141995862 | 2.09143368 |
| 2119 | 5 | 133.643 | Marker525274 | 0.216728649 | 0.094636525 | 0.146785044 | 2.07533123 |
| 2120 | 5 | 133.643 | Marker525271 | 0.216728649 | 0.094636525 | 0.146785044 | 2.07533123 |
| 2121 | 5 | 133.643 | Marker525275 | 0.216728649 | 0.094636525 | 0.146785044 | 2.07533123 |
| 2122 | 5 | 133.643 | Marker525273 | 0.216728649 | 0.094636525 | 0.146785044 | 2.07533123 |
| 2123 | 5 | 133.893 | Marker525276 | 0.221217973 | 0.095744681 | 0.146560628 | 2.095548912 |
| 2124 | 5 | 134.893 | Marker524848 | 0.242416624 | 0.076570048 | 0.183069 | 2.326580183 |
| 2125 | 5 | 134.893 | Marker524843 | 0.242416624 | 0.076570048 | 0.183069 | 2.326580183 |
| 2126 | 5 | 135.644 | Marker524750 | 0.234037662 | 0.079545455 | 0.1808799 | 2.339000922 |
| 2127 | 5 | 135.644 | Marker525032 | 0.234037662 | 0.079545455 | 0.1808799 | 2.339000922 |
| 2128 | 5 | 135.644 | Marker525033 | 0.234037662 | 0.079545455 | 0.1808799 | 2.339000922 |
| 2129 | 5 | 136.144 | Marker525726 | 0.234842508 | 0.079756215 | 0.179034989 | 2.309688147 |
| 2130 | 5 | 136.394 | Marker526469 | 0.235911678 | 0.080073996 | 0.179016913 | 2.315058047 |
| 2131 | 5 | 136.394 | Marker526531 | 0.235911678 | 0.080073996 | 0.179016913 | 2.315058047 |
| 2132 | 5 | 136.394 | Marker527482 | 0.235911678 | 0.080073996 | 0.179016913 | 2.315058047 |
| 2133 | 5 | 137.144 | Marker528938 | 0.28273336 | 0.091056911 | 0.195429576 | 2.831825256 |
| 2134 | 5 | 137.144 | Marker529178 | 0.28273336 | 0.091056911 | 0.195429576 | 2.831825256 |
| 2135 | 5 | 137.144 | Marker529180 | 0.28273336 | 0.091056911 | 0.195429576 | 2.831825256 |
| 2136 | 5 | 137.144 | Marker529179 | 0.28273336 | 0.091056911 | 0.195429576 | 2.831825256 |
| 2137 | 5 | 137.394 | Marker529433 | 0.364127702 | 0.103935698 | 0.215869496 | 3.531984336 |
| 2138 | 5 | 137.394 | Marker529434 | 0.364127702 | 0.103935698 | 0.215869496 | 3.531984336 |
| 2139 | 5 | 138.144 | Marker529754 | 0.459323771 | 0.101084011 | 0.256873947 | 4.431865357 |
| 2140 | 5 | 139.695 | Marker530998 | 0.491665367 | 0.101086957 | 0.213249119 | 3.410557576 |
| 2141 | 5 | 142.046 | Marker531645 | 0.325845519 | 0.097383721 | 0.178559675 | 2.650533575 |
| 2142 | 5 | 142.046 | Marker531648 | 0.325845519 | 0.097383721 | 0.178559675 | 2.650533575 |
| 2143 | 5 | 142.046 | Marker531646 | 0.325845519 | 0.097383721 | 0.178559675 | 2.650533575 |
| 2144 | 5 | 143.047 | Marker531520 | 0.355055718 | 0.104037267 | 0.205486542 | 3.316216979 |
| 2145 | 5 | 143.047 | Marker531523 | 0.355055718 | 0.104037267 | 0.205486542 | 3.316216979 |
| 2146 | 5 | 143.047 | Marker531516 | 0.355055718 | 0.104037267 | 0.205486542 | 3.316216979 |
| 2147 | 5 | 143.047 | Marker531522 | 0.355055718 | 0.104037267 | 0.205486542 | 3.316216979 |
| 2148 | 5 | 143.047 | Marker531521 | 0.355055718 | 0.104037267 | 0.205486542 | 3.316216979 |
| 2149 | 5 | 143.797 | Marker531841 | 0.355376155 | 0.104761905 | 0.203143782 | 3.285388181 |
| 2150 | 5 | 145.848 | Marker536989 | 0.180910985 | 0.094090909 | 0.181248897 | 2.62835901 |
| 2151 | 5 | 145.848 | Marker536862 | 0.180910985 | 0.094090909 | 0.181248897 | 2.62835901 |
| 2152 | 5 | 145.848 | Marker536860 | 0.180910985 | 0.094090909 | 0.181248897 | 2.62835901 |
| 2153 | 5 | 146.098 | Marker537627 | 0.199010705 | 0.098461932 | 0.197738675 | 3.034151269 |
| 2154 | 5 | 146.098 | Marker537629 | 0.199010705 | 0.098461932 | 0.197738675 | 3.034151269 |
| 2155 | 5 | 146.348 | Marker537818 | 0.181973755 | 0.092156863 | 0.183906038 | 2.636472552 |
| 2156 | 5 | 146.348 | Marker537817 | 0.181973755 | 0.092156863 | 0.183906038 | 2.636472552 |
| 2157 | 5 | 146.848 | Marker538591 | 0.199375281 | 0.090301003 | 0.186699774 | 2.650209358 |
| 2158 | 5 | 146.848 | Marker538590 | 0.199375281 | 0.090301003 | 0.186699774 | 2.650209358 |
| 2159 | 5 | 146.848 | Marker538589 | 0.199375281 | 0.090301003 | 0.186699774 | 2.650209358 |
| 2160 | 5 | 147.598 | Marker531756 | 0.110450433 | 0.071824382 | 0.146606991 | 1.648803114 |
| 2161 | 5 | 147.598 | Marker531757 | 0.110450433 | 0.071824382 | 0.146606991 | 1.648803114 |
| 2162 | 5 | 147.598 | Marker531755 | 0.110450433 | 0.071824382 | 0.146606991 | 1.648803114 |
| 2163 | 5 | 148.598 | Marker537776 | 0.062716517 | 0.064703154 | 0.121521336 | 1.204627635 |
| 2164 | 5 | 149.898 | Marker539291 | 0.146231642 | 0.073165436 | 0.106870935 | 1.167590543 |
| 2165 | 5 | 151.149 | Marker539118 | 0.116773189 | 0.070251177 | 0.072377028 | 0.811115429 |
| 2166 | 5 | 151.149 | Marker539120 | 0.116773189 | 0.070251177 | 0.072377028 | 0.811115429 |
| 2167 | 5 | 151.149 | Marker539119 | 0.116773189 | 0.070251177 | 0.072377028 | 0.811115429 |
| 2168 | 5 | 151.399 | Marker539139 | 0.104639824 | 0.070628251 | 0.071680218 | 0.811960417 |
| 2169 | 5 | 151.399 | Marker539141 | 0.104639824 | 0.070628251 | 0.071680218 | 0.811960417 |
| 2170 | 5 | 151.399 | Marker539140 | 0.104639824 | 0.070628251 | 0.071680218 | 0.811960417 |
| 2171 | 5 | 152.649 | Marker538941 | 0.182101429 | 0.082553191 | 0.127446809 | 1.571581796 |
| 2172 | 5 | 154.45 | Marker540942 | 0.074730158 | 0.053093645 | 0.067147225 | 0.539440534 |
| 2173 | 5 | 154.45 | Marker541046 | 0.074730158 | 0.053093645 | 0.067147225 | 0.539440534 |
| 2174 | 5 | 154.45 | Marker540692 | 0.074730158 | 0.053093645 | 0.067147225 | 0.539440534 |
| 2175 | 5 | 154.45 | Marker540693 | 0.074730158 | 0.053093645 | 0.067147225 | 0.539440534 |
| 2176 | 5 | 155.2 | Marker541955 | 0.062525168 | 0.062450749 | 0.04981777 | 0.557416695 |
| 2177 | 5 | 155.2 | Marker541954 | 0.062525168 | 0.062450749 | 0.04981777 | 0.557416695 |
| 2178 | 5 | 155.2 | Marker541953 | 0.062525168 | 0.062450749 | 0.04981777 | 0.557416695 |
| 2179 | 5 | 155.7 | Marker541974 | 0.076681476 | 0.063781788 | 0.046922871 | 0.561849544 |
| 2180 | 5 | 155.7 | Marker541845 | 0.076681476 | 0.063781788 | 0.046922871 | 0.561849544 |
| 2181 | 5 | 155.7 | Marker542024 | 0.076681476 | 0.063781788 | 0.046922871 | 0.561849544 |
| 2182 | 5 | 155.7 | Marker542025 | 0.076681476 | 0.063781788 | 0.046922871 | 0.561849544 |
| 2183 | 5 | 155.7 | Marker541976 | 0.076681476 | 0.063781788 | 0.046922871 | 0.561849544 |
| 2184 | 5 | 155.7 | Marker541979 | 0.076681476 | 0.063781788 | 0.046922871 | 0.561849544 |
| 2185 | 5 | 156.7 | Marker542530 | 0.066871294 | 0.08408347 | 0.006575871 | 0.781019046 |
| 2186 | 5 | 156.7 | Marker542401 | 0.066871294 | 0.08408347 | 0.006575871 | 0.781019046 |
| 2187 | 5 | 157.45 | Marker543401 | 0.087151967 | 0.084272007 | 0.006042907 | 0.784099129 |
| 2188 | 5 | 157.95 | Marker543307 | 0.112474772 | 0.092323154 | 0.029788902 | 0.986670027 |
| 2189 | 5 | 158.2 | Marker543310 | 0.111609419 | 0.094468085 | 0.025531915 | 1.018496998 |
| 2190 | 5 | 158.2 | Marker543297 | 0.111609419 | 0.094468085 | 0.025531915 | 1.018496998 |
| 2191 | 5 | 158.2 | Marker543308 | 0.111609419 | 0.094468085 | 0.025531915 | 1.018496998 |
| 2192 | 5 | 158.95 | Marker544194 | 0.089737223 | 0.096086957 | 0.023120964 | 1.046293762 |
| 2193 | 5 | 158.95 | Marker544581 | 0.089737223 | 0.096086957 | 0.023120964 | 1.046293762 |
| 2194 | 5 | 158.95 | Marker544161 | 0.089737223 | 0.096086957 | 0.023120964 | 1.046293762 |
| 2195 | 5 | 158.95 | Marker544586 | 0.089737223 | 0.096086957 | 0.023120964 | 1.046293762 |
| 2196 | 5 | 158.95 | Marker544582 | 0.089737223 | 0.096086957 | 0.023120964 | 1.046293762 |
| 2197 | 5 | 158.95 | Marker544587 | 0.089737223 | 0.096086957 | 0.023120964 | 1.046293762 |
| 2198 | 5 | 158.95 | Marker544394 | 0.089737223 | 0.096086957 | 0.023120964 | 1.046293762 |
| 2199 | 5 | 158.95 | Marker544195 | 0.089737223 | 0.096086957 | 0.023120964 | 1.046293762 |
| 2200 | 5 | 158.95 | Marker544395 | 0.089737223 | 0.096086957 | 0.023120964 | 1.046293762 |
| 2201 | 5 | 159.2 | Marker541392 | 0.093353953 | 0.094468085 | 0.025531915 | 1.018496998 |
| 2202 | 5 | 159.2 | Marker543527 | 0.093353953 | 0.094468085 | 0.025531915 | 1.018496998 |
| 2203 | 5 | 159.2 | Marker543813 | 0.093353953 | 0.094468085 | 0.025531915 | 1.018496998 |
| 2204 | 5 | 159.2 | Marker544225 | 0.093353953 | 0.094468085 | 0.025531915 | 1.018496998 |
| 2205 | 5 | 159.2 | Marker541391 | 0.093353953 | 0.094468085 | 0.025531915 | 1.018496998 |
| 2206 | 5 | 159.45 | Marker544224 | 0.090611016 | 0.094075928 | 0.025947839 | 1.011473156 |
| 2207 | 5 | 159.7 | Marker543698 | 0.086775593 | 0.0956948 | 0.023520887 | 1.03899409 |
| 2208 | 5 | 159.95 | Marker536014 | 0.134133348 | 0.106086957 | 0.043021954 | 1.338055294 |
| 2209 | 5 | 159.95 | Marker536013 | 0.134133348 | 0.106086957 | 0.043021954 | 1.338055294 |
| 2210 | 5 | 159.95 | Marker534327 | 0.134133348 | 0.106086957 | 0.043021954 | 1.338055294 |
| 2211 | 5 | 159.95 | Marker536025 | 0.134133348 | 0.106086957 | 0.043021954 | 1.338055294 |
| 2212 | 5 | 159.95 | Marker534340 | 0.134133348 | 0.106086957 | 0.043021954 | 1.338055294 |
| 2213 | 5 | 159.95 | Marker534339 | 0.134133348 | 0.106086957 | 0.043021954 | 1.338055294 |
| 2214 | 5 | 161.2 | Marker534158 | 0.285973627 | 0.103829787 | 0.086170213 | 1.569363862 |
| 2215 | 5 | 161.2 | Marker534159 | 0.285973627 | 0.103829787 | 0.086170213 | 1.569363862 |
| 2216 | 5 | 161.45 | Marker534358 | 0.2406733 | 0.094361306 | 0.064466496 | 1.196123727 |
| 2217 | 5 | 161.7 | Marker534359 | 0.250905466 | 0.094892112 | 0.062828328 | 1.196651507 |
| 2218 | 5 | 162.75 | Marker548781 | 0.304482043 | 0.107834776 | 0.077196894 | 1.589064323 |
| 2219 | 5 | 163 | Marker548778 | 0.354331805 | 0.097985348 | 0.095442583 | 1.523534016 |
| 2220 | 5 | 163.5 | Marker546194 | 0.385462311 | 0.097985348 | 0.095442583 | 1.523534016 |
| 2221 | 5 | 163.5 | Marker547647 | 0.385462311 | 0.097985348 | 0.095442583 | 1.523534016 |
| 2222 | 5 | 163.5 | Marker547732 | 0.385462311 | 0.097985348 | 0.095442583 | 1.523534016 |
| 2223 | 5 | 163.5 | Marker546195 | 0.385462311 | 0.097985348 | 0.095442583 | 1.523534016 |
| 2224 | 5 | 163.75 | Marker547320 | 0.729824413 | 0.108543417 | 0.114495798 | 1.966705018 |
| 2225 | 5 | 164 | Marker548078 | 0.750654568 | 0.107159143 | 0.117093665 | 1.963797857 |
| 2226 | 5 | 164 | Marker547099 | 0.750654568 | 0.107159143 | 0.117093665 | 1.963797857 |
| 2227 | 5 | 164 | Marker547101 | 0.750654568 | 0.107159143 | 0.117093665 | 1.963797857 |
| 2228 | 5 | 164 | Marker547100 | 0.750654568 | 0.107159143 | 0.117093665 | 1.963797857 |
| 2229 | 5 | 164.5 | Marker547146 | 0.674574598 | 0.115686275 | 0.101164822 | 1.999552306 |
| 2230 | 5 | 164.5 | Marker547145 | 0.674574598 | 0.115686275 | 0.101164822 | 1.999552306 |
| 2231 | 5 | 164.5 | Marker547143 | 0.674574598 | 0.115686275 | 0.101164822 | 1.999552306 |
| 2232 | 5 | 164.5 | Marker547144 | 0.674574598 | 0.115686275 | 0.101164822 | 1.999552306 |
| 2233 | 5 | 164.75 | Marker547219 | 0.926044084 | 0.125933912 | 0.119092613 | 2.473101145 |
| 2234 | 5 | 165 | Marker547561 | 0.874308416 | 0.127985409 | 0.118041944 | 2.518305199 |
| 2235 | 5 | 166.051 | Marker547380 | 0.666480116 | 0.103421895 | 0.082817717 | 1.531373041 |
| 2236 | 5 | 166.051 | Marker547378 | 0.666480116 | 0.103421895 | 0.082817717 | 1.531373041 |
| 2237 | 5 | 166.051 | Marker547377 | 0.666480116 | 0.103421895 | 0.082817717 | 1.531373041 |
| 2238 | 5 | 166.051 | Marker547379 | 0.666480116 | 0.103421895 | 0.082817717 | 1.531373041 |
| 2239 | 5 | 167.601 | Marker549090 | 0.311487158 | 0.075785483 | 0.024786868 | 0.665707412 |
| 2240 | 5 | 167.601 | Marker549089 | 0.311487158 | 0.075785483 | 0.024786868 | 0.665707412 |
| 2241 | 5 | 167.601 | Marker549091 | 0.311487158 | 0.075785483 | 0.024786868 | 0.665707412 |
| 2242 | 5 | 169.204 | Marker549519 | 0.466098057 | 0.094968553 | 0.062645321 | 1.197090763 |
| 2243 | 5 | 171.005 | Marker550274 | 0.468847597 | 0.076328495 | 0.060960471 | 0.833124898 |
| 2244 | 5 | 172.305 | Marker549935 | 0.567346218 | 0.065224218 | 0.094031417 | 0.916144261 |
| 2245 | 5 | 174.214 | Marker553765 | 0.311465636 | 0.040532561 | 0.080094192 | 0.50365183 |
| 2246 | 5 | 174.47 | Marker553766 | 0.338846354 | 0.04206503 | 0.084967847 | 0.557924506 |
| 2247 | 5 | 175.329 | Marker555177 | 0.256117348 | 0.044743708 | 0.051163186 | 0.353789659 |
| 2248 | 5 | 176.38 | Marker557210 | 0.265491666 | 0.045141827 | 0.11263291 | 0.860303637 |
| 2249 | 5 | 176.63 | Marker557211 | 0.254340619 | 0.047271076 | 0.116205783 | 0.923097247 |
| 2250 | 5 | 177.38 | Marker560332 | 0.047574747 | 0.053703704 | 0.066855631 | 0.544724592 |
| 2251 | 5 | 177.63 | Marker560333 | 0.042489509 | 0.053878407 | 0.065999619 | 0.541110524 |
| 2252 | 5 | 177.88 | Marker557697 | 0.045259373 | 0.064685315 | 0.082687115 | 0.807956046 |
| 2253 | 5 | 177.88 | Marker557735 | 0.045259373 | 0.064685315 | 0.082687115 | 0.807956046 |
| 2254 | 5 | 177.88 | Marker557737 | 0.045259373 | 0.064685315 | 0.082687115 | 0.807956046 |
| 2255 | 5 | 177.88 | Marker557736 | 0.045259373 | 0.064685315 | 0.082687115 | 0.807956046 |
| 2256 | 5 | 177.88 | Marker557683 | 0.045259373 | 0.064685315 | 0.082687115 | 0.807956046 |
| 2257 | 5 | 178.13 | Marker557207 | 0.013045227 | 0.069105314 | 0.074355201 | 0.807986028 |
| 2258 | 5 | 178.88 | Marker559630 | 0.02310942 | 0.097632073 | 0.057933114 | 1.224922874 |
| 2259 | 5 | 178.88 | Marker559632 | 0.02310942 | 0.097632073 | 0.057933114 | 1.224922874 |
| 2260 | 5 | 179.13 | Marker562872 | 0.030366702 | 0.099206349 | 0.055359107 | 1.244308382 |
| 2261 | 5 | 179.38 | Marker560471 | 0.03897191 | 0.088753388 | 0.074645305 | 1.154093815 |
| 2262 | 5 | 179.38 | Marker561523 | 0.03897191 | 0.088753388 | 0.074645305 | 1.154093815 |
| 2263 | 5 | 179.38 | Marker562861 | 0.03897191 | 0.088753388 | 0.074645305 | 1.154093815 |
| 2264 | 5 | 179.631 | Marker560442 | 0.039121025 | 0.066486972 | 0.079092014 | 0.805056742 |
| 2265 | 5 | 179.631 | Marker560443 | 0.039121025 | 0.066486972 | 0.079092014 | 0.805056742 |
| 2266 | 5 | 180.131 | Marker557832 | 0.072809183 | 0.055816135 | 0.098030019 | 0.827587515 |
| 2267 | 5 | 180.381 | Marker557554 | 0.06143322 | 0.056730769 | 0.096016484 | 0.819563753 |
| 2268 | 5 | 180.381 | Marker557555 | 0.06143322 | 0.056730769 | 0.096016484 | 0.819563753 |
| 2269 | 5 | 180.381 | Marker557553 | 0.06143322 | 0.056730769 | 0.096016484 | 0.819563753 |
| 2270 | 5 | 181.931 | Marker557203 | 0.025796821 | 0.070149976 | 0.072347778 | 0.809325813 |
| 2271 | 5 | 183.482 | Marker555259 | 0.05824339 | 0.040540541 | 0.052917403 | 0.323107609 |
| 2272 | 5 | 183.482 | Marker557202 | 0.05824339 | 0.040540541 | 0.052917403 | 0.323107609 |
| 2273 | 5 | 183.482 | Marker555264 | 0.05824339 | 0.040540541 | 0.052917403 | 0.323107609 |
| 2274 | 5 | 183.482 | Marker555263 | 0.05824339 | 0.040540541 | 0.052917403 | 0.323107609 |
| 2275 | 5 | 183.482 | Marker557198 | 0.05824339 | 0.040540541 | 0.052917403 | 0.323107609 |
| 2276 | 5 | 183.982 | Marker553804 | 0.196147598 | 0.040540541 | 0.052917403 | 0.323107609 |
| 2277 | 5 | 183.982 | Marker555299 | 0.196147598 | 0.040540541 | 0.052917403 | 0.323107609 |
| 2278 | 5 | 183.982 | Marker554961 | 0.196147598 | 0.040540541 | 0.052917403 | 0.323107609 |
| 2279 | 5 | 183.982 | Marker555301 | 0.196147598 | 0.040540541 | 0.052917403 | 0.323107609 |
| 2280 | 5 | 183.982 | Marker555300 | 0.196147598 | 0.040540541 | 0.052917403 | 0.323107609 |
| 2281 | 6 | 0 | Marker567970 | 0.423299801 | 0.050271739 | -0.137228261 | 1.195859389 |
| 2282 | 6 | 0.604 | Marker567066 | 0.327104665 | 0.05177305 | -0.099392097 | 0.773584908 |
| 2283 | 6 | 0.956 | Marker569134 | 0.44766884 | 0.053623188 | -0.101421931 | 0.814643117 |
| 2284 | 6 | 1.206 | Marker566620 | 0.446887008 | 0.052865613 | -0.102355842 | 0.815220199 |
| 2285 | 6 | 1.206 | Marker566619 | 0.446887008 | 0.052865613 | -0.102355842 | 0.815220199 |
| 2286 | 6 | 1.706 | Marker566805 | 0.676989731 | 0.049233053 | -0.142256309 | 1.253726605 |
| 2287 | 6 | 1.706 | Marker566627 | 0.676989731 | 0.049233053 | -0.142256309 | 1.253726605 |
| 2288 | 6 | 2.206 | Marker564285 | 0.70971101 | 0.050207039 | -0.145445135 | 1.309164365 |
| 2289 | 6 | 2.456 | Marker567030 | 0.57283078 | 0.062942366 | -0.123450549 | 1.174642084 |
| 2290 | 6 | 2.706 | Marker565309 | 0.476400379 | 0.054005168 | -0.105423986 | 0.859600148 |
| 2291 | 6 | 2.706 | Marker565308 | 0.476400379 | 0.054005168 | -0.105423986 | 0.859600148 |
| 2292 | 6 | 2.956 | Marker568169 | 0.491812211 | 0.044021579 | -0.124446631 | 0.968365621 |
| 2293 | 6 | 2.956 | Marker567029 | 0.491812211 | 0.044021579 | -0.124446631 | 0.968365621 |
| 2294 | 6 | 3.206 | Marker566962 | 0.662728359 | 0.032070707 | -0.145707071 | 1.153564849 |
| 2295 | 6 | 3.456 | Marker566875 | 0.660066806 | 0.034090909 | -0.147727273 | 1.196894654 |
| 2296 | 6 | 3.706 | Marker565241 | 0.656786063 | 0.032769556 | -0.149048626 | 1.206827464 |
| 2297 | 6 | 4.206 | Marker565242 | 0.641488494 | 0.042990818 | -0.147901084 | 1.272492309 |
| 2298 | 6 | 4.706 | Marker564288 | 0.543666325 | 0.035714286 | -0.113253266 | 0.766653196 |
| 2299 | 6 | 4.956 | Marker565445 | 0.555915826 | 0.034552846 | -0.114569961 | 0.772652519 |
| 2300 | 6 | 5.707 | Marker568291 | 0.631763936 | 0.048780488 | -0.137645811 | 1.185615512 |
| 2301 | 6 | 6.058 | Marker564587 | 0.680948758 | 0.089244186 | -0.118211954 | 1.547408093 |
| 2302 | 6 | 6.058 | Marker564588 | 0.680948758 | 0.089244186 | -0.118211954 | 1.547408093 |
| 2303 | 6 | 6.058 | Marker564584 | 0.680948758 | 0.089244186 | -0.118211954 | 1.547408093 |
| 2304 | 6 | 6.058 | Marker564586 | 0.680948758 | 0.089244186 | -0.118211954 | 1.547408093 |
| 2305 | 6 | 6.058 | Marker564662 | 0.680948758 | 0.089244186 | -0.118211954 | 1.547408093 |
| 2306 | 6 | 6.058 | Marker564273 | 0.680948758 | 0.089244186 | -0.118211954 | 1.547408093 |
| 2307 | 6 | 6.058 | Marker564585 | 0.680948758 | 0.089244186 | -0.118211954 | 1.547408093 |
| 2308 | 6 | 6.058 | Marker564459 | 0.680948758 | 0.089244186 | -0.118211954 | 1.547408093 |
| 2309 | 6 | 6.058 | Marker565111 | 0.680948758 | 0.089244186 | -0.118211954 | 1.547408093 |
| 2310 | 6 | 6.308 | Marker565575 | 0.704609145 | 0.076785714 | -0.139518634 | 1.590844333 |
| 2311 | 6 | 6.909 | Marker564572 | 0.631763936 | 0.048780488 | -0.137645811 | 1.185615512 |
| 2312 | 6 | 6.909 | Marker565873 | 0.631763936 | 0.048780488 | -0.137645811 | 1.185615512 |
| 2313 | 6 | 6.909 | Marker567004 | 0.631763936 | 0.048780488 | -0.137645811 | 1.185615512 |
| 2314 | 6 | 6.909 | Marker565874 | 0.631763936 | 0.048780488 | -0.137645811 | 1.185615512 |
| 2315 | 6 | 6.909 | Marker567732 | 0.631763936 | 0.048780488 | -0.137645811 | 1.185615512 |
| 2316 | 6 | 6.909 | Marker565665 | 0.631763936 | 0.048780488 | -0.137645811 | 1.185615512 |
| 2317 | 6 | 6.909 | Marker567728 | 0.631763936 | 0.048780488 | -0.137645811 | 1.185615512 |
| 2318 | 6 | 6.909 | Marker565709 | 0.631763936 | 0.048780488 | -0.137645811 | 1.185615512 |
| 2319 | 6 | 6.909 | Marker565507 | 0.631763936 | 0.048780488 | -0.137645811 | 1.185615512 |
| 2320 | 6 | 6.909 | Marker565310 | 0.631763936 | 0.048780488 | -0.137645811 | 1.185615512 |
| 2321 | 6 | 6.909 | Marker565705 | 0.631763936 | 0.048780488 | -0.137645811 | 1.185615512 |
| 2322 | 6 | 6.909 | Marker565704 | 0.631763936 | 0.048780488 | -0.137645811 | 1.185615512 |
| 2323 | 6 | 6.909 | Marker565707 | 0.631763936 | 0.048780488 | -0.137645811 | 1.185615512 |
| 2324 | 6 | 6.909 | Marker567729 | 0.631763936 | 0.048780488 | -0.137645811 | 1.185615512 |
| 2325 | 6 | 6.909 | Marker565708 | 0.631763936 | 0.048780488 | -0.137645811 | 1.185615512 |
| 2326 | 6 | 7.409 | Marker566645 | 0.497482955 | 0.073170732 | -0.095864263 | 1.030280983 |
| 2327 | 6 | 7.409 | Marker567003 | 0.497482955 | 0.073170732 | -0.095864263 | 1.030280983 |
| 2328 | 6 | 7.409 | Marker567005 | 0.497482955 | 0.073170732 | -0.095864263 | 1.030280983 |
| 2329 | 6 | 7.659 | Marker567339 | 0.405026855 | 0.058943089 | -0.072635858 | 0.635092959 |
| 2330 | 6 | 7.909 | Marker566658 | 0.400795646 | 0.057005105 | -0.070387363 | 0.594973257 |
| 2331 | 6 | 8.159 | Marker566329 | 0.405127661 | 0.069490587 | -0.048657254 | 0.642084807 |
| 2332 | 6 | 8.409 | Marker565922 | 0.404103288 | 0.069767442 | -0.047978211 | 0.643146616 |
| 2333 | 6 | 8.409 | Marker568344 | 0.404103288 | 0.069767442 | -0.047978211 | 0.643146616 |
| 2334 | 6 | 9.159 | Marker570475 | 0.361696583 | 0.068181818 | -0.005681818 | 0.512359879 |
| 2335 | 6 | 9.409 | Marker570474 | 0.361696583 | 0.068181818 | -0.005681818 | 0.512359879 |
| 2336 | 6 | 9.659 | Marker570473 | 0.359806366 | 0.068181818 | -0.005118755 | 0.512129663 |
| 2337 | 6 | 10.409 | Marker571606 | 0.357092058 | 0.057267442 | -0.029635863 | 0.401117626 |
| 2338 | 6 | 10.409 | Marker571607 | 0.357092058 | 0.057267442 | -0.029635863 | 0.401117626 |
| 2339 | 6 | 10.409 | Marker571601 | 0.357092058 | 0.057267442 | -0.029635863 | 0.401117626 |
| 2340 | 6 | 10.909 | Marker572089 | 0.355820238 | 0.05757232 | -0.028865219 | 0.40280697 |
| 2341 | 6 | 10.909 | Marker571605 | 0.355820238 | 0.05757232 | -0.028865219 | 0.40280697 |
| 2342 | 6 | 11.909 | Marker577643 | 0.125242068 | 0.034883721 | -0.027341295 | 0.169074218 |
| 2343 | 6 | 12.659 | Marker572090 | 0.075936497 | 0.020634921 | -0.004617605 | 0.047727204 |
| 2344 | 6 | 12.909 | Marker577640 | 0.075885034 | 0.020054201 | -0.005771625 | 0.045682381 |
| 2345 | 6 | 15.261 | Marker580114 | 0.049614513 | 0.025353017 | 0.111132264 | 0.686080586 |
| 2346 | 6 | 15.261 | Marker577656 | 0.049614513 | 0.025353017 | 0.111132264 | 0.686080586 |
| 2347 | 6 | 15.261 | Marker577639 | 0.049614513 | 0.025353017 | 0.111132264 | 0.686080586 |
| 2348 | 6 | 15.512 | Marker581206 | 0.051837003 | 0.025418275 | 0.111778243 | 0.69360293 |
| 2349 | 6 | 16.012 | Marker581041 | 0.085075608 | 0.038510911 | 0.132764735 | 1.043415271 |
| 2350 | 6 | 16.762 | Marker581039 | 0.068633003 | 0.025708635 | 0.110562089 | 0.681878579 |
| 2351 | 6 | 16.762 | Marker582576 | 0.068633003 | 0.025708635 | 0.110562089 | 0.681878579 |
| 2352 | 6 | 17.012 | Marker581040 | 0.057010079 | 0.020876259 | 0.102424204 | 0.570185614 |
| 2353 | 6 | 17.512 | Marker583202 | 0.071281307 | 0.013513514 | 0.13018018 | 0.860617927 |
| 2354 | 6 | 17.512 | Marker583201 | 0.071281307 | 0.013513514 | 0.13018018 | 0.860617927 |
| 2355 | 6 | 17.762 | Marker583200 | 0.076599842 | 0.000693001 | 0.150300968 | 1.116382807 |
| 2356 | 6 | 18.263 | Marker583579 | 0.084052539 | -2.97E-17 | 0.148978247 | 1.096580667 |
| 2357 | 6 | 18.513 | Marker583578 | 0.084052539 | -2.97E-17 | 0.148978247 | 1.096580667 |
| 2358 | 6 | 18.513 | Marker582563 | 0.084052539 | -2.97E-17 | 0.148978247 | 1.096580667 |
| 2359 | 6 | 18.513 | Marker583573 | 0.084052539 | -2.97E-17 | 0.148978247 | 1.096580667 |
| 2360 | 6 | 18.513 | Marker583574 | 0.084052539 | -2.97E-17 | 0.148978247 | 1.096580667 |
| 2361 | 6 | 18.763 | Marker584444 | 0.090099627 | 0.000355619 | 0.149622228 | 1.106192715 |
| 2362 | 6 | 18.763 | Marker582562 | 0.090099627 | 0.000355619 | 0.149622228 | 1.106192715 |
| 2363 | 6 | 18.763 | Marker584220 | 0.090099627 | 0.000355619 | 0.149622228 | 1.106192715 |
| 2364 | 6 | 19.013 | Marker585063 | 0.079383052 | 0.013513514 | 0.129215993 | 0.848236891 |
| 2365 | 6 | 19.263 | Marker586316 | 0.082371648 | 0.013888889 | 0.128642987 | 0.84214259 |
| 2366 | 6 | 19.513 | Marker584631 | 0.095558838 | 0.001388889 | 0.150355831 | 1.117548711 |
| 2367 | 6 | 19.513 | Marker586311 | 0.095558838 | 0.001388889 | 0.150355831 | 1.117548711 |
| 2368 | 6 | 19.513 | Marker586309 | 0.095558838 | 0.001388889 | 0.150355831 | 1.117548711 |
| 2369 | 6 | 19.513 | Marker586310 | 0.095558838 | 0.001388889 | 0.150355831 | 1.117548711 |
| 2370 | 6 | 19.513 | Marker586315 | 0.095558838 | 0.001388889 | 0.150355831 | 1.117548711 |
| 2371 | 6 | 19.513 | Marker586050 | 0.095558838 | 0.001388889 | 0.150355831 | 1.117548711 |
| 2372 | 6 | 19.513 | Marker585236 | 0.095558838 | 0.001388889 | 0.150355831 | 1.117548711 |
| 2373 | 6 | 19.513 | Marker584632 | 0.095558838 | 0.001388889 | 0.150355831 | 1.117548711 |
| 2374 | 6 | 20.013 | Marker584630 | 0.108146868 | 0.001318392 | 0.151759056 | 1.138457368 |
| 2375 | 6 | 20.263 | Marker584627 | 0.106255709 | 0.001109606 | 0.15217743 | 1.144627113 |
| 2376 | 6 | 21.013 | Marker584626 | 0.103481488 | 0.013129431 | 0.130860604 | 0.868192614 |
| 2377 | 6 | 21.513 | Marker583490 | 0.077093826 | -3.86E-17 | 0.111111111 | 0.609971929 |
| 2378 | 6 | 21.513 | Marker580986 | 0.077093826 | -3.86E-17 | 0.111111111 | 0.609971929 |
| 2379 | 6 | 21.763 | Marker580985 | 0.082219072 | 4.83E-16 | 0.112068966 | 0.620534017 |
| 2380 | 6 | 22.263 | Marker580978 | 0.123647501 | 0.00029036 | 0.155678831 | 1.197532632 |
| 2381 | 6 | 23.013 | Marker580984 | 0.136771826 | -0.011904762 | 0.177307206 | 1.565002796 |
| 2382 | 6 | 23.763 | Marker581286 | 0.054455719 | -0.03461945 | 0.139164905 | 1.079920943 |
| 2383 | 6 | 24.013 | Marker581282 | 0.083552953 | -0.045454545 | 0.160341952 | 1.484234732 |
| 2384 | 6 | 25.115 | Marker581698 | 0.067383144 | -0.005623099 | 0.123353661 | 0.754003756 |
| 2385 | 6 | 25.615 | Marker583691 | 0.131724402 | -0.016607063 | 0.144015166 | 1.050701804 |
| 2386 | 6 | 26.469 | Marker583687 | 0.073986579 | -0.021690938 | 0.123273633 | 0.797691753 |
| 2387 | 6 | 26.969 | Marker583686 | 0.050742511 | -0.032426304 | 0.104486714 | 0.64889364 |
| 2388 | 6 | 27.97 | Marker584930 | 0.047767941 | -0.032433729 | 0.144560371 | 1.139667137 |
| 2389 | 6 | 28.72 | Marker584929 | 0.011490478 | -0.031471631 | 0.107452023 | 0.673244756 |
| 2390 | 6 | 30.02 | Marker585313 | 0.095554495 | -0.021276596 | 0.126213592 | 0.831951662 |
| 2391 | 6 | 30.27 | Marker585312 | 0.113552357 | -0.021054965 | 0.127672612 | 0.849216246 |
| 2392 | 6 | 31.32 | Marker585680 | 0.096560281 | 0.000923238 | 0.08559604 | 0.362233089 |
| 2393 | 6 | 31.82 | Marker585679 | 0.083185088 | -0.006697062 | 0.071695474 | 0.258023619 |
| 2394 | 6 | 33.12 | Marker586579 | 0.063494712 | -0.010498914 | 0.107581533 | 0.581895199 |
| 2395 | 6 | 34.921 | Marker586578 | 0.218771934 | -0.009803922 | 0.107843137 | 0.583259274 |
| 2396 | 6 | 35.522 | Marker587459 | 0.19699358 | -0.010204082 | 0.105180534 | 0.556086975 |
| 2397 | 6 | 36.124 | Marker586940 | 0.19699358 | -0.010204082 | 0.105180534 | 0.556086975 |
| 2398 | 6 | 36.874 | Marker587146 | 0.178611058 | -0.010638298 | 0.101511235 | 0.519599051 |
| 2399 | 6 | 37.124 | Marker587145 | 0.152946209 | -0.02173913 | 0.080112721 | 0.36591029 |
| 2400 | 6 | 37.374 | Marker590348 | 0.102205252 | -0.009834369 | 0.058099227 | 0.176372138 |
| 2401 | 6 | 37.624 | Marker588985 | 0.047924917 | -0.009544008 | 0.056974839 | 0.16941081 |
| 2402 | 6 | 37.874 | Marker588984 | 0.039354139 | -0.0100271 | 0.055672746 | 0.163177564 |
| 2403 | 6 | 38.475 | Marker587682 | 0.066715629 | -0.022222222 | 0.075122911 | 0.330107943 |
| 2404 | 6 | 38.475 | Marker587681 | 0.066715629 | -0.022222222 | 0.075122911 | 0.330107943 |
| 2405 | 6 | 38.475 | Marker587683 | 0.066715629 | -0.022222222 | 0.075122911 | 0.330107943 |
| 2406 | 6 | 38.975 | Marker589367 | 0.04974947 | -0.023323293 | 0.05960083 | 0.232815536 |
| 2407 | 6 | 39.326 | Marker587688 | 0.064241384 | -0.014127649 | 0.078682524 | 0.325805794 |
| 2408 | 6 | 39.826 | Marker587680 | 0.077524724 | -0.011904762 | 0.091543514 | 0.427646043 |
| 2409 | 6 | 40.076 | Marker586406 | 0.097298707 | 8.69E-17 | 0.111111111 | 0.609971929 |
| 2410 | 6 | 40.076 | Marker587074 | 0.097298707 | 8.69E-17 | 0.111111111 | 0.609971929 |
| 2411 | 6 | 40.076 | Marker587679 | 0.097298707 | 8.69E-17 | 0.111111111 | 0.609971929 |
| 2412 | 6 | 40.826 | Marker587689 | 0.049180909 | 0.0125 | 0.090948276 | 0.427951946 |
| 2413 | 6 | 40.826 | Marker586875 | 0.049180909 | 0.0125 | 0.090948276 | 0.427951946 |
| 2414 | 6 | 41.076 | Marker589669 | 0.05273555 | 0.0125 | 0.090064103 | 0.420024198 |
| 2415 | 6 | 41.576 | Marker587816 | 0.023596937 | 1.23E-17 | 0.07126193 | 0.250905412 |
| 2416 | 6 | 41.576 | Marker592932 | 0.023596937 | 1.23E-17 | 0.07126193 | 0.250905412 |
| 2417 | 6 | 42.326 | Marker589671 | 0.028142089 | 0.000872093 | 0.072363321 | 0.25892026 |
| 2418 | 6 | 42.326 | Marker589668 | 0.028142089 | 0.000872093 | 0.072363321 | 0.25892026 |
| 2419 | 6 | 42.678 | Marker594563 | 0.086974069 | 0.001709402 | 0.073413509 | 0.266836216 |
| 2420 | 6 | 43.978 | Marker594562 | 0.15062799 | -3.05E-18 | 0.114035088 | 0.642498133 |
| 2421 | 6 | 44.228 | Marker591918 | 0.122108417 | -0.010638298 | 0.095556392 | 0.461734679 |
| 2422 | 6 | 44.228 | Marker594561 | 0.122108417 | -0.010638298 | 0.095556392 | 0.461734679 |
| 2423 | 6 | 44.728 | Marker594690 | 0.085412734 | 0.002875216 | 0.073193321 | 0.265984799 |
| 2424 | 6 | 45.478 | Marker593062 | 0.032973936 | -0.01745435 | 0.038048042 | 0.103829208 |
| 2425 | 6 | 45.478 | Marker594689 | 0.032973936 | -0.01745435 | 0.038048042 | 0.103829208 |
| 2426 | 6 | 45.478 | Marker591928 | 0.032973936 | -0.01745435 | 0.038048042 | 0.103829208 |
| 2427 | 6 | 45.478 | Marker593623 | 0.032973936 | -0.01745435 | 0.038048042 | 0.103829208 |
| 2428 | 6 | 45.478 | Marker593702 | 0.032973936 | -0.01745435 | 0.038048042 | 0.103829208 |
| 2429 | 6 | 45.478 | Marker592589 | 0.032973936 | -0.01745435 | 0.038048042 | 0.103829208 |
| 2430 | 6 | 45.478 | Marker593622 | 0.032973936 | -0.01745435 | 0.038048042 | 0.103829208 |
| 2431 | 6 | 45.728 | Marker592588 | 0.019421446 | -0.028508772 | 0.017247511 | 0.103219624 |
| 2432 | 6 | 45.728 | Marker595104 | 0.019421446 | -0.028508772 | 0.017247511 | 0.103219624 |
| 2433 | 6 | 45.728 | Marker592494 | 0.019421446 | -0.028508772 | 0.017247511 | 0.103219624 |
| 2434 | 6 | 45.728 | Marker595105 | 0.019421446 | -0.028508772 | 0.017247511 | 0.103219624 |
| 2435 | 6 | 46.478 | Marker594947 | 0.030670433 | -0.018429487 | 0.037747669 | 0.10649649 |
| 2436 | 6 | 46.478 | Marker595455 | 0.030670433 | -0.018429487 | 0.037747669 | 0.10649649 |
| 2437 | 6 | 46.478 | Marker595103 | 0.030670433 | -0.018429487 | 0.037747669 | 0.10649649 |
| 2438 | 6 | 46.478 | Marker595041 | 0.030670433 | -0.018429487 | 0.037747669 | 0.10649649 |
| 2439 | 6 | 46.478 | Marker594943 | 0.030670433 | -0.018429487 | 0.037747669 | 0.10649649 |
| 2440 | 6 | 46.478 | Marker595454 | 0.030670433 | -0.018429487 | 0.037747669 | 0.10649649 |
| 2441 | 6 | 46.478 | Marker594950 | 0.030670433 | -0.018429487 | 0.037747669 | 0.10649649 |
| 2442 | 6 | 46.478 | Marker595451 | 0.030670433 | -0.018429487 | 0.037747669 | 0.10649649 |
| 2443 | 6 | 46.478 | Marker594955 | 0.030670433 | -0.018429487 | 0.037747669 | 0.10649649 |
| 2444 | 6 | 46.728 | Marker594953 | 0.037194731 | -0.03125 | 0.060493119 | 0.284789213 |
| 2445 | 6 | 47.228 | Marker594952 | 0.114443572 | -0.043154762 | 0.0834585 | 0.542450798 |
| 2446 | 6 | 47.978 | Marker595040 | 0.172595457 | -0.053339518 | 0.107499643 | 0.873500394 |
| 2447 | 6 | 48.228 | Marker596292 | 0.143354517 | -0.041723356 | 0.087294983 | 0.5613758 |
| 2448 | 6 | 48.728 | Marker596293 | 0.160042363 | -0.040522876 | 0.090610237 | 0.579599278 |
| 2449 | 6 | 49.228 | Marker594749 | 0.145233239 | -0.033448183 | 0.079746301 | 0.432418418 |
| 2450 | 6 | 50.228 | Marker594748 | 0.317729251 | -0.069391664 | 0.157648338 | 1.737698208 |
| 2451 | 6 | 50.978 | Marker594747 | 0.359059259 | -0.058354685 | 0.138720771 | 1.310622328 |
| 2452 | 6 | 51.728 | Marker594746 | 0.476905006 | -0.05682969 | 0.144758092 | 1.375614314 |
| 2453 | 6 | 52.979 | Marker597672 | 0.471275857 | -0.062865497 | 0.132224942 | 1.283427211 |
| 2454 | 6 | 52.979 | Marker597671 | 0.471275857 | -0.062865497 | 0.132224942 | 1.283427211 |
| 2455 | 6 | 53.83 | Marker598937 | 0.503710098 | -0.061111111 | 0.139022758 | 1.350263556 |
| 2456 | 6 | 54.08 | Marker598936 | 0.501618837 | -0.060856257 | 0.139657608 | 1.355578961 |
| 2457 | 6 | 55.434 | Marker598900 | 0.123613891 | -0.043332267 | 0.093182716 | 0.628209206 |
| 2458 | 6 | 56.184 | Marker598899 | 0.101814353 | -0.033898305 | 0.068374422 | 0.353170505 |
| 2459 | 6 | 56.684 | Marker599495 | 0.100103614 | -0.034238101 | 0.066992786 | 0.346521238 |
| 2460 | 6 | 57.434 | Marker598898 | 0.102453393 | -0.033898305 | 0.06732197 | 0.346179674 |
| 2461 | 6 | 57.934 | Marker598896 | 0.090336907 | -0.034482759 | 0.066268843 | 0.343620209 |
| 2462 | 6 | 58.184 | Marker598895 | 0.090804087 | -0.034482759 | 0.065517241 | 0.338773768 |
| 2463 | 6 | 59.435 | Marker600591 | 0.113861483 | -0.011755486 | 0.016474179 | 0.028259134 |
| 2464 | 6 | 60.435 | Marker599539 | 0.16213174 | 0.008143949 | 0.013194774 | 0.016095826 |
| 2465 | 6 | 61.435 | Marker600598 | 0.161822907 | 0.007610994 | 0.008550623 | 0.01010481 |
| 2466 | 6 | 62.185 | Marker600596 | 0.130304954 | 0.014940861 | 0.029517562 | 0.068415822 |
| 2467 | 6 | 63.986 | Marker598902 | 0.104695646 | 0.003496503 | 0.047150869 | 0.111490261 |
| 2468 | 6 | 64.736 | Marker598901 | 0.109800294 | 0.009239472 | 0.052894325 | 0.148520207 |
| 2469 | 6 | 65.236 | Marker596767 | 0.090091863 | 0.005 | 0.03911215 | 0.078690427 |
| 2470 | 6 | 65.236 | Marker596762 | 0.090091863 | 0.005 | 0.03911215 | 0.078690427 |
| 2471 | 6 | 65.736 | Marker596765 | 0.087343514 | -0.010935569 | 0.052631591 | 0.148967717 |
| 2472 | 6 | 66.236 | Marker594939 | 0.093456391 | -0.006842105 | 0.058585225 | 0.173995786 |
| 2473 | 6 | 66.736 | Marker596889 | 0.093680334 | -0.00689465 | 0.056168413 | 0.160397425 |
| 2474 | 6 | 66.736 | Marker596179 | 0.093680334 | -0.00689465 | 0.056168413 | 0.160397425 |
| 2475 | 6 | 66.736 | Marker596892 | 0.093680334 | -0.00689465 | 0.056168413 | 0.160397425 |
| 2476 | 6 | 66.736 | Marker596885 | 0.093680334 | -0.00689465 | 0.056168413 | 0.160397425 |
| 2477 | 6 | 66.736 | Marker596887 | 0.093680334 | -0.00689465 | 0.056168413 | 0.160397425 |
| 2478 | 6 | 66.736 | Marker596891 | 0.093680334 | -0.00689465 | 0.056168413 | 0.160397425 |
| 2479 | 6 | 66.736 | Marker596888 | 0.093680334 | -0.00689465 | 0.056168413 | 0.160397425 |
| 2480 | 6 | 66.736 | Marker596178 | 0.093680334 | -0.00689465 | 0.056168413 | 0.160397425 |
| 2481 | 6 | 66.736 | Marker596879 | 0.093680334 | -0.00689465 | 0.056168413 | 0.160397425 |
| 2482 | 6 | 66.736 | Marker596886 | 0.093680334 | -0.00689465 | 0.056168413 | 0.160397425 |
| 2483 | 6 | 66.986 | Marker597427 | 0.091056207 | -0.006486486 | 0.057395577 | 0.166709439 |
| 2484 | 6 | 66.986 | Marker596878 | 0.091056207 | -0.006486486 | 0.057395577 | 0.166709439 |
| 2485 | 6 | 66.986 | Marker594938 | 0.091056207 | -0.006486486 | 0.057395577 | 0.166709439 |
| 2486 | 6 | 66.986 | Marker597187 | 0.091056207 | -0.006486486 | 0.057395577 | 0.166709439 |
| 2487 | 6 | 66.986 | Marker596761 | 0.091056207 | -0.006486486 | 0.057395577 | 0.166709439 |
| 2488 | 6 | 66.986 | Marker598210 | 0.091056207 | -0.006486486 | 0.057395577 | 0.166709439 |
| 2489 | 6 | 66.986 | Marker597109 | 0.091056207 | -0.006486486 | 0.057395577 | 0.166709439 |
| 2490 | 6 | 66.986 | Marker595756 | 0.091056207 | -0.006486486 | 0.057395577 | 0.166709439 |
| 2491 | 6 | 67.236 | Marker598031 | 0.090108139 | 0.006315789 | 0.036253018 | 0.069743397 |
| 2492 | 6 | 67.736 | Marker598247 | 0.070125562 | -0.004934211 | 0.014506283 | 0.012944683 |
| 2493 | 6 | 67.736 | Marker598264 | 0.070125562 | -0.004934211 | 0.014506283 | 0.012944683 |
| 2494 | 6 | 68.236 | Marker599442 | 0.082060192 | -0.0137893 | -0.00478029 | 0.022170077 |
| 2495 | 6 | 68.236 | Marker599441 | 0.082060192 | -0.0137893 | -0.00478029 | 0.022170077 |
| 2496 | 6 | 68.236 | Marker598263 | 0.082060192 | -0.0137893 | -0.00478029 | 0.022170077 |
| 2497 | 6 | 68.236 | Marker598487 | 0.082060192 | -0.0137893 | -0.00478029 | 0.022170077 |
| 2498 | 6 | 68.236 | Marker598225 | 0.082060192 | -0.0137893 | -0.00478029 | 0.022170077 |
| 2499 | 6 | 68.736 | Marker599439 | 0.268528383 | 0.002040816 | -0.030576124 | 0.046535306 |
| 2500 | 6 | 68.736 | Marker601064 | 0.268528383 | 0.002040816 | -0.030576124 | 0.046535306 |
| 2501 | 6 | 68.736 | Marker599440 | 0.268528383 | 0.002040816 | -0.030576124 | 0.046535306 |
| 2502 | 6 | 68.736 | Marker600600 | 0.268528383 | 0.002040816 | -0.030576124 | 0.046535306 |
| 2503 | 6 | 69.736 | Marker600181 | 0.094890193 | -0.010513296 | -0.010249792 | 0.01754873 |
| 2504 | 6 | 69.986 | Marker600180 | 0.083345974 | -0.01969697 | -0.027671451 | 0.081515293 |
| 2505 | 6 | 69.986 | Marker600093 | 0.083345974 | -0.01969697 | -0.027671451 | 0.081515293 |
| 2506 | 6 | 69.986 | Marker600144 | 0.083345974 | -0.01969697 | -0.027671451 | 0.081515293 |
| 2507 | 6 | 69.986 | Marker600740 | 0.083345974 | -0.01969697 | -0.027671451 | 0.081515293 |
| 2508 | 6 | 70.236 | Marker600739 | 0.084707635 | -0.020717378 | -0.029149525 | 0.090309818 |
| 2509 | 6 | 70.736 | Marker601109 | 0.08452148 | -0.021608643 | -0.027800594 | 0.090658932 |
| 2510 | 6 | 71.236 | Marker601891 | 0.227221695 | -0.013095238 | -0.008834586 | 0.022935414 |
| 2511 | 6 | 71.236 | Marker601962 | 0.227221695 | -0.013095238 | -0.008834586 | 0.022935414 |
| 2512 | 6 | 71.736 | Marker601975 | 0.140260895 | -0.033928571 | -0.047211779 | 0.239712209 |
| 2513 | 6 | 71.736 | Marker601890 | 0.140260895 | -0.033928571 | -0.047211779 | 0.239712209 |
| 2514 | 6 | 71.736 | Marker601723 | 0.140260895 | -0.033928571 | -0.047211779 | 0.239712209 |
| 2515 | 6 | 71.986 | Marker602581 | 0.106816612 | -0.034722222 | -0.04602999 | 0.240255958 |
| 2516 | 6 | 71.986 | Marker601701 | 0.106816612 | -0.034722222 | -0.04602999 | 0.240255958 |
| 2517 | 6 | 71.986 | Marker601716 | 0.106816612 | -0.034722222 | -0.04602999 | 0.240255958 |
| 2518 | 6 | 71.986 | Marker601700 | 0.106816612 | -0.034722222 | -0.04602999 | 0.240255958 |
| 2519 | 6 | 72.236 | Marker601696 | 0.106586587 | -0.03484724 | -0.045839185 | 0.240345164 |
| 2520 | 6 | 72.736 | Marker601719 | 0.105507325 | -0.035472973 | -0.04488417 | 0.240896098 |
| 2521 | 6 | 72.736 | Marker603050 | 0.105507325 | -0.035472973 | -0.04488417 | 0.240896098 |
| 2522 | 6 | 72.736 | Marker601722 | 0.105507325 | -0.035472973 | -0.04488417 | 0.240896098 |
| 2523 | 6 | 72.736 | Marker601718 | 0.105507325 | -0.035472973 | -0.04488417 | 0.240896098 |
| 2524 | 6 | 72.736 | Marker601717 | 0.105507325 | -0.035472973 | -0.04488417 | 0.240896098 |
| 2525 | 6 | 72.736 | Marker603051 | 0.105507325 | -0.035472973 | -0.04488417 | 0.240896098 |
| 2526 | 6 | 73.236 | Marker603049 | 0.188638667 | -0.016451234 | -0.009101779 | 0.034144208 |
| 2527 | 6 | 73.486 | Marker603261 | 0.1886224 | -0.015700483 | -0.010386473 | 0.032749864 |
| 2528 | 6 | 73.486 | Marker603838 | 0.1886224 | -0.015700483 | -0.010386473 | 0.032749864 |
| 2529 | 6 | 73.486 | Marker603048 | 0.1886224 | -0.015700483 | -0.010386473 | 0.032749864 |
| 2530 | 6 | 73.486 | Marker603533 | 0.1886224 | -0.015700483 | -0.010386473 | 0.032749864 |
| 2531 | 6 | 73.486 | Marker603262 | 0.1886224 | -0.015700483 | -0.010386473 | 0.032749864 |
| 2532 | 6 | 73.736 | Marker602353 | 0.206320178 | -0.014906832 | -0.011704862 | 0.031537099 |
| 2533 | 6 | 73.736 | Marker602412 | 0.206320178 | -0.014906832 | -0.011704862 | 0.031537099 |
| 2534 | 6 | 73.736 | Marker602151 | 0.206320178 | -0.014906832 | -0.011704862 | 0.031537099 |
| 2535 | 6 | 73.736 | Marker602410 | 0.206320178 | -0.014906832 | -0.011704862 | 0.031537099 |
| 2536 | 6 | 73.736 | Marker603260 | 0.206320178 | -0.014906832 | -0.011704862 | 0.031537099 |
| 2537 | 6 | 73.736 | Marker603259 | 0.206320178 | -0.014906832 | -0.011704862 | 0.031537099 |
| 2538 | 6 | 73.736 | Marker602354 | 0.206320178 | -0.014906832 | -0.011704862 | 0.031537099 |
| 2539 | 6 | 73.986 | Marker605344 | 0.164336907 | -0.026984127 | -0.032844933 | 0.135035371 |
| 2540 | 6 | 74.236 | Marker602409 | 0.1686345 | -0.028246753 | -0.034542153 | 0.148522391 |
| 2541 | 6 | 74.486 | Marker605309 | 0.156906213 | -0.042929293 | -0.010878011 | 0.209466329 |
| 2542 | 6 | 74.736 | Marker605522 | 0.165169801 | -0.029791155 | -0.032121071 | 0.150375605 |
| 2543 | 6 | 74.736 | Marker603223 | 0.165169801 | -0.029791155 | -0.032121071 | 0.150375605 |
| 2544 | 6 | 74.736 | Marker605175 | 0.165169801 | -0.029791155 | -0.032121071 | 0.150375605 |
| 2545 | 6 | 75.486 | Marker603227 | 0.166304819 | -0.056818182 | 0.013978278 | 0.363390636 |
| 2546 | 6 | 75.986 | Marker603226 | 0.207434839 | -0.046511628 | 0.03313439 | 0.289441704 |
| 2547 | 6 | 76.486 | Marker603225 | 0.097176046 | -0.055555556 | 0.016516517 | 0.351378375 |
| 2548 | 6 | 76.736 | Marker603224 | 0.123496482 | -0.043478261 | 0.038339921 | 0.277560766 |
| 2549 | 6 | 76.986 | Marker606206 | 0.229554593 | -0.030978261 | 0.016093811 | 0.117469335 |
| 2550 | 6 | 77.236 | Marker606899 | 0.247203323 | -0.030657748 | 0.015129798 | 0.113872153 |
| 2551 | 6 | 78.236 | Marker607184 | 0.276703563 | -0.029957582 | -0.024192615 | 0.128985218 |
| 2552 | 6 | 78.486 | Marker607914 | 0.255065114 | -0.030538302 | -0.023111478 | 0.130288951 |
| 2553 | 6 | 79.236 | Marker607919 | 0.157979248 | -0.029471545 | 0.020212285 | 0.114658289 |
| 2554 | 6 | 79.486 | Marker607912 | 0.158618844 | -0.029166667 | 0.019227829 | 0.110836802 |
| 2555 | 6 | 79.486 | Marker607918 | 0.158618844 | -0.029166667 | 0.019227829 | 0.110836802 |
| 2556 | 6 | 79.486 | Marker607911 | 0.158618844 | -0.029166667 | 0.019227829 | 0.110836802 |
| 2557 | 6 | 79.486 | Marker607913 | 0.158618844 | -0.029166667 | 0.019227829 | 0.110836802 |
| 2558 | 6 | 79.736 | Marker607910 | 0.115056608 | -0.041666667 | 0.041666667 | 0.27361371 |
| 2559 | 6 | 79.736 | Marker608553 | 0.115056608 | -0.041666667 | 0.041666667 | 0.27361371 |
| 2560 | 6 | 79.736 | Marker608551 | 0.115056608 | -0.041666667 | 0.041666667 | 0.27361371 |
| 2561 | 6 | 80.236 | Marker610286 | 0.189121813 | -0.030053191 | 0.017674081 | 0.113834433 |
| 2562 | 6 | 80.236 | Marker610436 | 0.189121813 | -0.030053191 | 0.017674081 | 0.113834433 |
| 2563 | 6 | 80.236 | Marker608552 | 0.189121813 | -0.030053191 | 0.017674081 | 0.113834433 |
| 2564 | 6 | 80.486 | Marker610371 | 0.206577763 | -0.029166667 | 0.019227829 | 0.110836802 |
| 2565 | 6 | 80.486 | Marker610515 | 0.206577763 | -0.029166667 | 0.019227829 | 0.110836802 |
| 2566 | 6 | 80.736 | Marker610369 | 0.206577763 | -0.029166667 | 0.019227829 | 0.110836802 |
| 2567 | 6 | 80.736 | Marker610513 | 0.206577763 | -0.029166667 | 0.019227829 | 0.110836802 |
| 2568 | 6 | 80.986 | Marker610367 | 0.203153664 | -0.028846154 | 0.018240093 | 0.107025953 |
| 2569 | 6 | 80.986 | Marker607560 | 0.203153664 | -0.028846154 | 0.018240093 | 0.107025953 |
| 2570 | 6 | 80.986 | Marker610370 | 0.203153664 | -0.028846154 | 0.018240093 | 0.107025953 |
| 2571 | 6 | 80.986 | Marker610250 | 0.203153664 | -0.028846154 | 0.018240093 | 0.107025953 |
| 2572 | 6 | 80.986 | Marker607562 | 0.203153664 | -0.028846154 | 0.018240093 | 0.107025953 |
| 2573 | 6 | 81.236 | Marker608486 | 0.312850017 | -0.041666667 | 0.040902141 | 0.270553091 |
| 2574 | 6 | 81.236 | Marker608012 | 0.312850017 | -0.041666667 | 0.040902141 | 0.270553091 |
| 2575 | 6 | 81.236 | Marker612923 | 0.312850017 | -0.041666667 | 0.040902141 | 0.270553091 |
| 2576 | 6 | 81.486 | Marker612922 | 0.31704353 | -0.042553191 | 0.03926499 | 0.272344368 |
| 2577 | 6 | 82.487 | Marker612920 | 0.171488371 | -0.039262821 | -0.001267483 | 0.169779553 |
| 2578 | 6 | 82.487 | Marker612921 | 0.171488371 | -0.039262821 | -0.001267483 | 0.169779553 |
| 2579 | 6 | 82.487 | Marker611039 | 0.171488371 | -0.039262821 | -0.001267483 | 0.169779553 |
| 2580 | 6 | 82.487 | Marker613639 | 0.171488371 | -0.039262821 | -0.001267483 | 0.169779553 |
| 2581 | 6 | 82.987 | Marker610670 | 0.220169034 | -0.026020408 | -0.020464853 | 0.096159714 |
| 2582 | 6 | 82.987 | Marker611041 | 0.220169034 | -0.026020408 | -0.020464853 | 0.096159714 |
| 2583 | 6 | 82.987 | Marker610669 | 0.220169034 | -0.026020408 | -0.020464853 | 0.096159714 |
| 2584 | 6 | 83.237 | Marker611157 | 0.246622145 | -0.016666667 | -0.002446483 | 0.030932469 |
| 2585 | 6 | 83.237 | Marker611109 | 0.246622145 | -0.016666667 | -0.002446483 | 0.030932469 |
| 2586 | 6 | 83.237 | Marker611364 | 0.246622145 | -0.016666667 | -0.002446483 | 0.030932469 |
| 2587 | 6 | 83.237 | Marker611950 | 0.246622145 | -0.016666667 | -0.002446483 | 0.030932469 |
| 2588 | 6 | 83.737 | Marker611948 | 0.225808906 | -0.018478261 | -0.005415198 | 0.039199119 |
| 2589 | 6 | 83.737 | Marker614716 | 0.225808906 | -0.018478261 | -0.005415198 | 0.039199119 |
| 2590 | 6 | 84.237 | Marker612617 | 0.244395678 | -0.00625 | 0.017144495 | 0.018624316 |
| 2591 | 6 | 84.487 | Marker614714 | 0.241611635 | -0.005877726 | 0.017913458 | 0.01946293 |
| 2592 | 6 | 84.838 | Marker614721 | 0.224476539 | -0.007302804 | 0.017537949 | 0.020830111 |
| 2593 | 6 | 85.088 | Marker614160 | 0.22136975 | -0.006859756 | 0.01843383 | 0.021734953 |
| 2594 | 6 | 85.088 | Marker614715 | 0.22136975 | -0.006859756 | 0.01843383 | 0.021734953 |
| 2595 | 6 | 85.088 | Marker616114 | 0.22136975 | -0.006859756 | 0.01843383 | 0.021734953 |
| 2596 | 6 | 85.088 | Marker615071 | 0.22136975 | -0.006859756 | 0.01843383 | 0.021734953 |
| 2597 | 6 | 85.088 | Marker614713 | 0.22136975 | -0.006859756 | 0.01843383 | 0.021734953 |
| 2598 | 6 | 85.338 | Marker616116 | 0.204259598 | -0.019345238 | 0.040957388 | 0.122607009 |
| 2599 | 6 | 85.838 | Marker616065 | 0.246891894 | 0.001979218 | 0.03957974 | 0.077974307 |
| 2600 | 6 | 85.838 | Marker616115 | 0.246891894 | 0.001979218 | 0.03957974 | 0.077974307 |
| 2601 | 6 | 85.838 | Marker615790 | 0.246891894 | 0.001979218 | 0.03957974 | 0.077974307 |
| 2602 | 6 | 85.838 | Marker616064 | 0.246891894 | 0.001979218 | 0.03957974 | 0.077974307 |
| 2603 | 6 | 85.838 | Marker615637 | 0.246891894 | 0.001979218 | 0.03957974 | 0.077974307 |
| 2604 | 6 | 85.838 | Marker615791 | 0.246891894 | 0.001979218 | 0.03957974 | 0.077974307 |
| 2605 | 6 | 86.088 | Marker615639 | 0.268693478 | 0.01443769 | 0.017083193 | 0.037804356 |
| 2606 | 6 | 86.588 | Marker615635 | 0.246785811 | 0.005102041 | -0.000288795 | 0.002865431 |
| 2607 | 6 | 86.588 | Marker615636 | 0.246785811 | 0.005102041 | -0.000288795 | 0.002865431 |
| 2608 | 6 | 86.838 | Marker615638 | 0.284686694 | 0.015899383 | -0.019981016 | 0.046957181 |
| 2609 | 6 | 86.838 | Marker616326 | 0.284686694 | 0.015899383 | -0.019981016 | 0.046957181 |
| 2610 | 6 | 86.838 | Marker615748 | 0.284686694 | 0.015899383 | -0.019981016 | 0.046957181 |
| 2611 | 6 | 87.338 | Marker616324 | 0.317909866 | 0.030363365 | -0.044858883 | 0.198366226 |
| 2612 | 6 | 87.588 | Marker616164 | 0.320556885 | 0.031887755 | -0.046815949 | 0.217432014 |
| 2613 | 6 | 87.838 | Marker616389 | 0.315240875 | 0.0325 | -0.045771028 | 0.216998957 |
| 2614 | 6 | 87.838 | Marker616391 | 0.315240875 | 0.0325 | -0.045771028 | 0.216998957 |
| 2615 | 6 | 87.838 | Marker616982 | 0.315240875 | 0.0325 | -0.045771028 | 0.216998957 |
| 2616 | 6 | 87.838 | Marker616387 | 0.315240875 | 0.0325 | -0.045771028 | 0.216998957 |
| 2617 | 6 | 87.838 | Marker616390 | 0.315240875 | 0.0325 | -0.045771028 | 0.216998957 |
| 2618 | 6 | 87.838 | Marker616981 | 0.315240875 | 0.0325 | -0.045771028 | 0.216998957 |
| 2619 | 6 | 87.838 | Marker617993 | 0.315240875 | 0.0325 | -0.045771028 | 0.216998957 |
| 2620 | 6 | 87.838 | Marker616388 | 0.315240875 | 0.0325 | -0.045771028 | 0.216998957 |
| 2621 | 6 | 88.088 | Marker617218 | 0.239931786 | 0.034102564 | -0.047806268 | 0.237890899 |
| 2622 | 6 | 88.088 | Marker618254 | 0.239931786 | 0.034102564 | -0.047806268 | 0.237890899 |
| 2623 | 6 | 88.088 | Marker619400 | 0.239931786 | 0.034102564 | -0.047806268 | 0.237890899 |
| 2624 | 6 | 88.088 | Marker617610 | 0.239931786 | 0.034102564 | -0.047806268 | 0.237890899 |
| 2625 | 6 | 88.088 | Marker616977 | 0.239931786 | 0.034102564 | -0.047806268 | 0.237890899 |
| 2626 | 6 | 88.088 | Marker616980 | 0.239931786 | 0.034102564 | -0.047806268 | 0.237890899 |
| 2627 | 6 | 88.838 | Marker626117 | 0.229939768 | 0.033490319 | -0.048843249 | 0.238279582 |
| 2628 | 6 | 89.088 | Marker625784 | 0.225972046 | 0.034102564 | -0.047806268 | 0.237890899 |
| 2629 | 6 | 91.139 | Marker624295 | 0.285017523 | 0.041223404 | -0.029231141 | 0.226982694 |
| 2630 | 6 | 91.389 | Marker620647 | 0.283741311 | 0.039699014 | -0.027206334 | 0.20799303 |
| 2631 | 6 | 92.139 | Marker620648 | 0.632828477 | 0.024467054 | 0.039605273 | 0.145137309 |
| 2632 | 6 | 92.139 | Marker623507 | 0.632828477 | 0.024467054 | 0.039605273 | 0.145137309 |
| 2633 | 6 | 92.139 | Marker622241 | 0.632828477 | 0.024467054 | 0.039605273 | 0.145137309 |
| 2634 | 6 | 92.139 | Marker622242 | 0.632828477 | 0.024467054 | 0.039605273 | 0.145137309 |
| 2635 | 6 | 92.139 | Marker623653 | 0.632828477 | 0.024467054 | 0.039605273 | 0.145137309 |
| 2636 | 6 | 92.139 | Marker622596 | 0.632828477 | 0.024467054 | 0.039605273 | 0.145137309 |
| 2637 | 6 | 92.139 | Marker623652 | 0.632828477 | 0.024467054 | 0.039605273 | 0.145137309 |
| 2638 | 6 | 92.139 | Marker627199 | 0.632828477 | 0.024467054 | 0.039605273 | 0.145137309 |
| 2639 | 6 | 92.389 | Marker620997 | 0.644415188 | 0.024245423 | 0.038590131 | 0.139966411 |
| 2640 | 6 | 92.389 | Marker620996 | 0.644415188 | 0.024245423 | 0.038590131 | 0.139966411 |
| 2641 | 6 | 92.389 | Marker621508 | 0.644415188 | 0.024245423 | 0.038590131 | 0.139966411 |
| 2642 | 6 | 92.389 | Marker627086 | 0.644415188 | 0.024245423 | 0.038590131 | 0.139966411 |
| 2643 | 6 | 92.389 | Marker624182 | 0.644415188 | 0.024245423 | 0.038590131 | 0.139966411 |
| 2644 | 6 | 92.389 | Marker621555 | 0.644415188 | 0.024245423 | 0.038590131 | 0.139966411 |
| 2645 | 6 | 92.389 | Marker621510 | 0.644415188 | 0.024245423 | 0.038590131 | 0.139966411 |
| 2646 | 6 | 92.389 | Marker622539 | 0.644415188 | 0.024245423 | 0.038590131 | 0.139966411 |
| 2647 | 6 | 92.389 | Marker622252 | 0.644415188 | 0.024245423 | 0.038590131 | 0.139966411 |
| 2648 | 6 | 92.389 | Marker622534 | 0.644415188 | 0.024245423 | 0.038590131 | 0.139966411 |
| 2649 | 6 | 92.389 | Marker621446 | 0.644415188 | 0.024245423 | 0.038590131 | 0.139966411 |
| 2650 | 6 | 92.389 | Marker624130 | 0.644415188 | 0.024245423 | 0.038590131 | 0.139966411 |
| 2651 | 6 | 92.389 | Marker624131 | 0.644415188 | 0.024245423 | 0.038590131 | 0.139966411 |
| 2652 | 6 | 92.389 | Marker622538 | 0.644415188 | 0.024245423 | 0.038590131 | 0.139966411 |
| 2653 | 6 | 92.389 | Marker624129 | 0.644415188 | 0.024245423 | 0.038590131 | 0.139966411 |
| 2654 | 6 | 92.389 | Marker624135 | 0.644415188 | 0.024245423 | 0.038590131 | 0.139966411 |
| 2655 | 6 | 92.389 | Marker625140 | 0.644415188 | 0.024245423 | 0.038590131 | 0.139966411 |
| 2656 | 6 | 92.389 | Marker621297 | 0.644415188 | 0.024245423 | 0.038590131 | 0.139966411 |
| 2657 | 6 | 92.389 | Marker624134 | 0.644415188 | 0.024245423 | 0.038590131 | 0.139966411 |
| 2658 | 6 | 92.389 | Marker621296 | 0.644415188 | 0.024245423 | 0.038590131 | 0.139966411 |
| 2659 | 6 | 92.639 | Marker627924 | 0.528805692 | 0.014050388 | 0.019754644 | 0.041509227 |
| 2660 | 6 | 92.639 | Marker627923 | 0.528805692 | 0.014050388 | 0.019754644 | 0.041509227 |
| 2661 | 6 | 92.639 | Marker625144 | 0.528805692 | 0.014050388 | 0.019754644 | 0.041509227 |
| 2662 | 6 | 93.89 | Marker628048 | 0.538552817 | 0.022222222 | -0.001246106 | 0.054358731 |
| 2663 | 6 | 93.89 | Marker627970 | 0.538552817 | 0.022222222 | -0.001246106 | 0.054358731 |
| 2664 | 6 | 93.89 | Marker629247 | 0.538552817 | 0.022222222 | -0.001246106 | 0.054358731 |
| 2665 | 6 | 93.89 | Marker627975 | 0.538552817 | 0.022222222 | -0.001246106 | 0.054358731 |
| 2666 | 6 | 93.89 | Marker627971 | 0.538552817 | 0.022222222 | -0.001246106 | 0.054358731 |
| 2667 | 6 | 93.89 | Marker627973 | 0.538552817 | 0.022222222 | -0.001246106 | 0.054358731 |
| 2668 | 6 | 93.89 | Marker627974 | 0.538552817 | 0.022222222 | -0.001246106 | 0.054358731 |
| 2669 | 6 | 94.14 | Marker629246 | 0.548494348 | 0.011868687 | 0.017760943 | 0.031470032 |
| 2670 | 6 | 96.191 | Marker629185 | 0.205725907 | -0.013858093 | 0.012115933 | 0.028075289 |
| 2671 | 6 | 96.191 | Marker629186 | 0.205725907 | -0.013858093 | 0.012115933 | 0.028075289 |
| 2672 | 6 | 96.441 | Marker630447 | 0.232454244 | -0.012987013 | 0.013630514 | 0.027412446 |
| 2673 | 6 | 96.441 | Marker630122 | 0.232454244 | -0.012987013 | 0.013630514 | 0.027412446 |
| 2674 | 6 | 97.191 | Marker630627 | 0.275335183 | 0.000528541 | 0.035835095 | 0.063512526 |
| 2675 | 6 | 97.191 | Marker630446 | 0.275335183 | 0.000528541 | 0.035835095 | 0.063512526 |
| 2676 | 6 | 97.441 | Marker632813 | 0.165941169 | 2.39E-17 | 0.034569453 | 0.05904456 |
| 2677 | 6 | 97.441 | Marker631714 | 0.165941169 | 2.39E-17 | 0.034569453 | 0.05904456 |
| 2678 | 6 | 97.441 | Marker630631 | 0.165941169 | 2.39E-17 | 0.034569453 | 0.05904456 |
| 2679 | 6 | 97.441 | Marker632816 | 0.165941169 | 2.39E-17 | 0.034569453 | 0.05904456 |
| 2680 | 6 | 97.441 | Marker630605 | 0.165941169 | 2.39E-17 | 0.034569453 | 0.05904456 |
| 2681 | 6 | 97.441 | Marker633315 | 0.165941169 | 2.39E-17 | 0.034569453 | 0.05904456 |
| 2682 | 6 | 97.441 | Marker631223 | 0.165941169 | 2.39E-17 | 0.034569453 | 0.05904456 |
| 2683 | 6 | 97.691 | Marker634821 | 0.154809392 | -0.00055371 | 0.033291805 | 0.054760771 |
| 2684 | 6 | 97.691 | Marker633671 | 0.154809392 | -0.00055371 | 0.033291805 | 0.054760771 |
| 2685 | 6 | 97.941 | Marker633672 | 0.127488061 | -0.013329552 | 0.01095528 | 0.025211278 |
| 2686 | 6 | 98.191 | Marker630920 | 0.124637383 | -0.012458472 | 0.012458472 | 0.024461883 |
| 2687 | 6 | 98.441 | Marker630919 | 0.095334021 | -0.012987013 | 0.013630514 | 0.027412446 |
| 2688 | 6 | 98.441 | Marker634813 | 0.095334021 | -0.012987013 | 0.013630514 | 0.027412446 |
| 2689 | 6 | 99.191 | Marker638907 | 0.109394338 | -0.013492063 | 0.014790765 | 0.030471839 |
| 2690 | 6 | 99.191 | Marker638906 | 0.109394338 | -0.013492063 | 0.014790765 | 0.030471839 |
| 2691 | 6 | 99.441 | Marker639847 | 0.077630415 | -0.024289406 | -0.004513667 | 0.066118464 |
| 2692 | 6 | 99.441 | Marker640355 | 0.077630415 | -0.024289406 | -0.004513667 | 0.066118464 |
| 2693 | 6 | 99.691 | Marker641012 | 0.077513456 | -0.024772497 | -0.003435944 | 0.068258015 |
| 2694 | 6 | 99.691 | Marker638598 | 0.077513456 | -0.024772497 | -0.003435944 | 0.068258015 |
| 2695 | 6 | 100.191 | Marker638597 | 0.027674719 | -0.036507937 | 0.013997114 | 0.155387632 |
| 2696 | 6 | 100.691 | Marker650185 | 0.00924715 | -0.03698075 | 0.015816729 | 0.16175567 |
| 2697 | 6 | 100.941 | Marker638758 | 0.013360001 | -0.03587333 | 0.017616429 | 0.155765711 |
| 2698 | 6 | 100.941 | Marker638110 | 0.013360001 | -0.03587333 | 0.017616429 | 0.155765711 |
| 2699 | 6 | 100.941 | Marker639526 | 0.013360001 | -0.03587333 | 0.017616429 | 0.155765711 |
| 2700 | 6 | 100.941 | Marker639525 | 0.013360001 | -0.03587333 | 0.017616429 | 0.155765711 |
| 2701 | 6 | 101.191 | Marker637037 | 0.015702325 | -0.025678295 | -0.001307225 | 0.072692703 |
| 2702 | 6 | 101.691 | Marker639669 | 0.058905814 | -0.025046382 | 0.001444984 | 0.069057132 |
| 2703 | 6 | 101.691 | Marker637034 | 0.058905814 | -0.025046382 | 0.001444984 | 0.069057132 |
| 2704 | 6 | 101.691 | Marker648757 | 0.058905814 | -0.025046382 | 0.001444984 | 0.069057132 |
| 2705 | 6 | 101.691 | Marker643597 | 0.058905814 | -0.025046382 | 0.001444984 | 0.069057132 |
| 2706 | 6 | 101.691 | Marker667602 | 0.058905814 | -0.025046382 | 0.001444984 | 0.069057132 |
| 2707 | 6 | 101.691 | Marker667101 | 0.058905814 | -0.025046382 | 0.001444984 | 0.069057132 |
| 2708 | 6 | 101.691 | Marker668713 | 0.058905814 | -0.025046382 | 0.001444984 | 0.069057132 |
| 2709 | 6 | 101.691 | Marker643598 | 0.058905814 | -0.025046382 | 0.001444984 | 0.069057132 |
| 2710 | 6 | 101.691 | Marker648752 | 0.058905814 | -0.025046382 | 0.001444984 | 0.069057132 |
| 2711 | 6 | 101.691 | Marker680699 | 0.058905814 | -0.025046382 | 0.001444984 | 0.069057132 |
| 2712 | 6 | 101.691 | Marker680700 | 0.058905814 | -0.025046382 | 0.001444984 | 0.069057132 |
| 2713 | 6 | 101.691 | Marker671856 | 0.058905814 | -0.025046382 | 0.001444984 | 0.069057132 |
| 2714 | 6 | 101.691 | Marker667171 | 0.058905814 | -0.025046382 | 0.001444984 | 0.069057132 |
| 2715 | 6 | 101.691 | Marker667197 | 0.058905814 | -0.025046382 | 0.001444984 | 0.069057132 |
| 2716 | 6 | 101.941 | Marker643412 | 0.078464529 | -0.024621212 | 0.000378788 | 0.066686831 |
| 2717 | 6 | 101.941 | Marker643411 | 0.078464529 | -0.024621212 | 0.000378788 | 0.066686831 |
| 2718 | 6 | 101.941 | Marker643416 | 0.078464529 | -0.024621212 | 0.000378788 | 0.066686831 |
| 2719 | 6 | 101.941 | Marker646272 | 0.078464529 | -0.024621212 | 0.000378788 | 0.066686831 |
| 2720 | 6 | 101.941 | Marker645231 | 0.078464529 | -0.024621212 | 0.000378788 | 0.066686831 |
| 2721 | 6 | 101.941 | Marker643417 | 0.078464529 | -0.024621212 | 0.000378788 | 0.066686831 |
| 2722 | 6 | 101.941 | Marker667351 | 0.078464529 | -0.024621212 | 0.000378788 | 0.066686831 |
| 2723 | 6 | 101.941 | Marker645232 | 0.078464529 | -0.024621212 | 0.000378788 | 0.066686831 |
| 2724 | 6 | 101.941 | Marker643419 | 0.078464529 | -0.024621212 | 0.000378788 | 0.066686831 |
| 2725 | 6 | 101.941 | Marker641083 | 0.078464529 | -0.024621212 | 0.000378788 | 0.066686831 |
| 2726 | 6 | 101.941 | Marker667198 | 0.078464529 | -0.024621212 | 0.000378788 | 0.066686831 |
| 2727 | 6 | 101.941 | Marker640652 | 0.078464529 | -0.024621212 | 0.000378788 | 0.066686831 |
| 2728 | 6 | 101.941 | Marker643410 | 0.078464529 | -0.024621212 | 0.000378788 | 0.066686831 |
| 2729 | 6 | 101.941 | Marker643415 | 0.078464529 | -0.024621212 | 0.000378788 | 0.066686831 |
| 2730 | 6 | 102.441 | Marker641109 | 0.013008218 | -0.037731372 | -0.021404841 | 0.180750829 |
| 2731 | 6 | 102.441 | Marker642354 | 0.013008218 | -0.037731372 | -0.021404841 | 0.180750829 |
| 2732 | 6 | 102.441 | Marker668726 | 0.013008218 | -0.037731372 | -0.021404841 | 0.180750829 |
| 2733 | 6 | 102.441 | Marker642334 | 0.013008218 | -0.037731372 | -0.021404841 | 0.180750829 |
| 2734 | 6 | 102.441 | Marker670332 | 0.013008218 | -0.037731372 | -0.021404841 | 0.180750829 |
| 2735 | 6 | 102.441 | Marker671854 | 0.013008218 | -0.037731372 | -0.021404841 | 0.180750829 |
| 2736 | 6 | 102.441 | Marker642336 | 0.013008218 | -0.037731372 | -0.021404841 | 0.180750829 |
| 2737 | 6 | 102.441 | Marker671855 | 0.013008218 | -0.037731372 | -0.021404841 | 0.180750829 |
| 2738 | 6 | 102.691 | Marker641952 | 0.013008218 | -0.037731372 | -0.021404841 | 0.180750829 |
| 2739 | 6 | 102.691 | Marker667200 | 0.013008218 | -0.037731372 | -0.021404841 | 0.180750829 |
| 2740 | 6 | 102.691 | Marker639914 | 0.013008218 | -0.037731372 | -0.021404841 | 0.180750829 |
| 2741 | 6 | 102.691 | Marker641112 | 0.013008218 | -0.037731372 | -0.021404841 | 0.180750829 |
| 2742 | 6 | 102.691 | Marker667199 | 0.013008218 | -0.037731372 | -0.021404841 | 0.180750829 |
| 2743 | 6 | 102.691 | Marker667242 | 0.013008218 | -0.037731372 | -0.021404841 | 0.180750829 |
| 2744 | 6 | 102.691 | Marker641953 | 0.013008218 | -0.037731372 | -0.021404841 | 0.180750829 |
| 2745 | 6 | 102.691 | Marker667196 | 0.013008218 | -0.037731372 | -0.021404841 | 0.180750829 |
| 2746 | 6 | 102.941 | Marker648753 | 0.058905814 | -0.025046382 | 0.001444984 | 0.069057132 |
| 2747 | 6 | 102.941 | Marker650184 | 0.058905814 | -0.025046382 | 0.001444984 | 0.069057132 |
| 2748 | 6 | 102.941 | Marker649352 | 0.058905814 | -0.025046382 | 0.001444984 | 0.069057132 |
| 2749 | 6 | 102.941 | Marker668676 | 0.058905814 | -0.025046382 | 0.001444984 | 0.069057132 |
| 2750 | 6 | 102.941 | Marker650207 | 0.058905814 | -0.025046382 | 0.001444984 | 0.069057132 |
| 2751 | 6 | 102.941 | Marker648751 | 0.058905814 | -0.025046382 | 0.001444984 | 0.069057132 |
| 2752 | 6 | 102.941 | Marker649893 | 0.058905814 | -0.025046382 | 0.001444984 | 0.069057132 |
| 2753 | 6 | 103.441 | Marker650824 | 0.003337968 | -0.047722868 | -0.002518466 | 0.251109194 |
| 2754 | 6 | 103.441 | Marker649218 | 0.003337968 | -0.047722868 | -0.002518466 | 0.251109194 |
| 2755 | 6 | 103.691 | Marker649313 | 0.003065184 | -0.049107143 | -0.004519915 | 0.266738934 |
| 2756 | 6 | 103.691 | Marker648464 | 0.003065184 | -0.049107143 | -0.004519915 | 0.266738934 |
| 2757 | 6 | 103.691 | Marker649873 | 0.003065184 | -0.049107143 | -0.004519915 | 0.266738934 |
| 2758 | 6 | 103.691 | Marker649872 | 0.003065184 | -0.049107143 | -0.004519915 | 0.266738934 |
| 2759 | 6 | 103.691 | Marker650256 | 0.003065184 | -0.049107143 | -0.004519915 | 0.266738934 |
| 2760 | 6 | 103.691 | Marker649314 | 0.003065184 | -0.049107143 | -0.004519915 | 0.266738934 |
| 2761 | 6 | 103.691 | Marker649315 | 0.003065184 | -0.049107143 | -0.004519915 | 0.266738934 |
| 2762 | 6 | 103.691 | Marker650983 | 0.003065184 | -0.049107143 | -0.004519915 | 0.266738934 |
| 2763 | 6 | 103.941 | Marker652776 | 0.000449063 | -0.038363821 | 0.01487692 | 0.171821622 |
| 2764 | 6 | 103.941 | Marker650605 | 0.000449063 | -0.038363821 | 0.01487692 | 0.171821622 |
| 2765 | 6 | 103.941 | Marker652051 | 0.000449063 | -0.038363821 | 0.01487692 | 0.171821622 |
| 2766 | 6 | 103.941 | Marker650255 | 0.000449063 | -0.038363821 | 0.01487692 | 0.171821622 |
| 2767 | 6 | 103.941 | Marker650907 | 0.000449063 | -0.038363821 | 0.01487692 | 0.171821622 |
| 2768 | 6 | 103.941 | Marker648576 | 0.000449063 | -0.038363821 | 0.01487692 | 0.171821622 |
| 2769 | 6 | 104.941 | Marker655472 | 0.005092892 | -0.05 | 0.032568807 | 0.324487428 |
| 2770 | 6 | 104.941 | Marker652718 | 0.005092892 | -0.05 | 0.032568807 | 0.324487428 |
| 2771 | 6 | 104.941 | Marker655426 | 0.005092892 | -0.05 | 0.032568807 | 0.324487428 |
| 2772 | 6 | 105.191 | Marker652715 | 0.0051734 | -0.048780488 | 0.034552846 | 0.317708832 |
| 2773 | 6 | 105.191 | Marker657286 | 0.0051734 | -0.048780488 | 0.034552846 | 0.317708832 |
| 2774 | 6 | 105.191 | Marker655712 | 0.0051734 | -0.048780488 | 0.034552846 | 0.317708832 |
| 2775 | 6 | 105.441 | Marker654076 | 0.005575671 | -0.048780488 | 0.03378832 | 0.315195593 |
| 2776 | 6 | 105.441 | Marker657914 | 0.005575671 | -0.048780488 | 0.03378832 | 0.315195593 |
| 2777 | 6 | 105.691 | Marker658613 | 0.013170897 | -0.059197154 | 0.054228771 | 0.524978219 |
| 2778 | 6 | 105.941 | Marker658612 | 0.013149948 | -0.060416667 | 0.052159786 | 0.530259705 |
| 2779 | 6 | 106.441 | Marker656621 | 0.017204746 | -0.047002033 | 0.075683153 | 0.519558327 |
| 2780 | 6 | 106.691 | Marker659373 | 0.016924624 | -0.046130952 | 0.077506119 | 0.524396814 |
| 2781 | 6 | 106.692 | Marker659369 | 0.016924624 | -0.046130952 | 0.077506119 | 0.524396814 |
| 2782 | 6 | 106.692 | Marker659372 | 0.016924624 | -0.046130952 | 0.077506119 | 0.524396814 |
| 2783 | 6 | 106.692 | Marker658689 | 0.016924624 | -0.046130952 | 0.077506119 | 0.524396814 |
| 2784 | 6 | 106.942 | Marker660300 | 0.035571131 | -0.056990881 | 0.096673421 | 0.809022944 |
| 2785 | 6 | 106.942 | Marker660299 | 0.035571131 | -0.056990881 | 0.096673421 | 0.809022944 |
| 2786 | 6 | 106.942 | Marker660301 | 0.035571131 | -0.056990881 | 0.096673421 | 0.809022944 |
| 2787 | 6 | 107.442 | Marker660295 | 0.035571131 | -0.056990881 | 0.096673421 | 0.809022944 |
| 2788 | 6 | 108.192 | Marker660781 | 0.028988609 | -0.055871212 | 0.060200216 | 0.516351115 |
| 2789 | 6 | 108.442 | Marker660782 | 0.032629608 | -0.055658627 | 0.060903382 | 0.517901661 |
| 2790 | 6 | 108.442 | Marker661817 | 0.032629608 | -0.055658627 | 0.060903382 | 0.517901661 |
| 2791 | 6 | 108.442 | Marker662011 | 0.032629608 | -0.055658627 | 0.060903382 | 0.517901661 |
| 2792 | 6 | 108.442 | Marker661911 | 0.032629608 | -0.055658627 | 0.060903382 | 0.517901661 |
| 2793 | 6 | 108.692 | Marker663315 | 0.027738942 | -0.045087803 | 0.080082265 | 0.533921019 |
| 2794 | 6 | 109.192 | Marker663314 | 0.031956099 | -0.045714286 | 0.079047619 | 0.532039924 |
| 2795 | 6 | 109.942 | Marker663313 | 0.006963823 | -0.026969981 | 0.040337711 | 0.158431078 |
| 2796 | 6 | 109.942 | Marker663312 | 0.006963823 | -0.026969981 | 0.040337711 | 0.158431078 |
| 2797 | 6 | 109.942 | Marker662009 | 0.006963823 | -0.026969981 | 0.040337711 | 0.158431078 |
| 2798 | 6 | 109.942 | Marker663662 | 0.006963823 | -0.026969981 | 0.040337711 | 0.158431078 |
| 2799 | 6 | 109.942 | Marker663657 | 0.006963823 | -0.026969981 | 0.040337711 | 0.158431078 |
| 2800 | 6 | 109.942 | Marker663661 | 0.006963823 | -0.026969981 | 0.040337711 | 0.158431078 |
| 2801 | 6 | 110.192 | Marker664683 | 0.004649116 | -0.026781444 | 0.039324998 | 0.153393641 |
| 2802 | 6 | 110.192 | Marker662158 | 0.004649116 | -0.026781444 | 0.039324998 | 0.153393641 |
| 2803 | 6 | 110.192 | Marker662317 | 0.004649116 | -0.026781444 | 0.039324998 | 0.153393641 |
| 2804 | 6 | 110.192 | Marker663246 | 0.004649116 | -0.026781444 | 0.039324998 | 0.153393641 |
| 2805 | 6 | 110.442 | Marker672527 | 0.003886646 | -0.027696078 | 0.037601739 | 0.152347589 |
| 2806 | 6 | 110.442 | Marker672519 | 0.003886646 | -0.027696078 | 0.037601739 | 0.152347589 |
| 2807 | 6 | 112.243 | Marker672518 | 0.003647936 | -0.026098901 | 0.04204897 | 0.160293304 |
| 2808 | 6 | 112.743 | Marker672516 | 0.001158432 | -0.036585366 | 0.058652729 | 0.31330843 |
| 2809 | 6 | 112.743 | Marker672517 | 0.001158432 | -0.036585366 | 0.058652729 | 0.31330843 |
| 2810 | 6 | 112.993 | Marker673655 | 0.000728306 | -0.036585366 | 0.05956848 | 0.318596061 |
| 2811 | 6 | 112.993 | Marker674374 | 0.000728306 | -0.036585366 | 0.05956848 | 0.318596061 |
| 2812 | 6 | 112.993 | Marker672708 | 0.000728306 | -0.036585366 | 0.05956848 | 0.318596061 |
| 2813 | 6 | 112.993 | Marker673653 | 0.000728306 | -0.036585366 | 0.05956848 | 0.318596061 |
| 2814 | 6 | 113.243 | Marker674937 | 0.089929884 | -0.023809524 | 0.082986593 | 0.399015899 |
| 2815 | 6 | 113.493 | Marker675268 | 0.108508245 | -0.023255814 | 0.084587323 | 0.40941775 |
| 2816 | 6 | 113.993 | Marker675267 | 0.152454321 | -0.021167558 | 0.125891266 | 0.827465262 |
| 2817 | 6 | 114.243 | Marker675064 | 0.182314 | -0.009803922 | 0.148417783 | 1.096257871 |
| 2818 | 6 | 114.243 | Marker675909 | 0.182314 | -0.009803922 | 0.148417783 | 1.096257871 |
| 2819 | 6 | 114.243 | Marker675183 | 0.182314 | -0.009803922 | 0.148417783 | 1.096257871 |
| 2820 | 6 | 114.243 | Marker675908 | 0.182314 | -0.009803922 | 0.148417783 | 1.096257871 |
| 2821 | 6 | 114.243 | Marker675075 | 0.182314 | -0.009803922 | 0.148417783 | 1.096257871 |
| 2822 | 6 | 114.243 | Marker675620 | 0.182314 | -0.009803922 | 0.148417783 | 1.096257871 |
| 2823 | 6 | 114.243 | Marker675285 | 0.182314 | -0.009803922 | 0.148417783 | 1.096257871 |
| 2824 | 6 | 114.243 | Marker674838 | 0.182314 | -0.009803922 | 0.148417783 | 1.096257871 |
| 2825 | 6 | 114.243 | Marker675079 | 0.182314 | -0.009803922 | 0.148417783 | 1.096257871 |
| 2826 | 6 | 114.243 | Marker674840 | 0.182314 | -0.009803922 | 0.148417783 | 1.096257871 |
| 2827 | 6 | 114.243 | Marker675076 | 0.182314 | -0.009803922 | 0.148417783 | 1.096257871 |
| 2828 | 6 | 114.243 | Marker675286 | 0.182314 | -0.009803922 | 0.148417783 | 1.096257871 |
| 2829 | 6 | 114.243 | Marker675065 | 0.182314 | -0.009803922 | 0.148417783 | 1.096257871 |
| 2830 | 6 | 114.993 | Marker674839 | 0.252497061 | -0.030612245 | 0.18747499 | 1.829129329 |
| 2831 | 6 | 115.243 | Marker676661 | 0.185318736 | -0.041250543 | 0.168488799 | 1.577108739 |
| 2832 | 6 | 115.243 | Marker676812 | 0.185318736 | -0.041250543 | 0.168488799 | 1.577108739 |
| 2833 | 6 | 115.243 | Marker676809 | 0.185318736 | -0.041250543 | 0.168488799 | 1.577108739 |
| 2834 | 6 | 115.493 | Marker677415 | 0.225106953 | -0.030612245 | 0.190612245 | 1.887558859 |
| 2835 | 6 | 115.493 | Marker677416 | 0.225106953 | -0.030612245 | 0.190612245 | 1.887558859 |
| 2836 | 6 | 115.493 | Marker684622 | 0.225106953 | -0.030612245 | 0.190612245 | 1.887558859 |
| 2837 | 6 | 115.493 | Marker677444 | 0.225106953 | -0.030612245 | 0.190612245 | 1.887558859 |
| 2838 | 6 | 115.743 | Marker682730 | 0.24703782 | -0.030612245 | 0.192228407 | 1.918038484 |
| 2839 | 6 | 115.743 | Marker683662 | 0.24703782 | -0.030612245 | 0.192228407 | 1.918038484 |
| 2840 | 6 | 115.743 | Marker684584 | 0.24703782 | -0.030612245 | 0.192228407 | 1.918038484 |
| 2841 | 6 | 118.295 | Marker683655 | 0.095836378 | -0.04913522 | 0.117531447 | 0.937559818 |
| 2842 | 6 | 118.545 | Marker683375 | 0.082325632 | -0.049679487 | 0.11569852 | 0.92240516 |
| 2843 | 6 | 118.795 | Marker683376 | 0.0634644 | -0.039050235 | 0.095941261 | 0.615706148 |
| 2844 | 6 | 118.795 | Marker683553 | 0.0634644 | -0.039050235 | 0.095941261 | 0.615706148 |
| 2845 | 6 | 118.795 | Marker683528 | 0.0634644 | -0.039050235 | 0.095941261 | 0.615706148 |
| 2846 | 6 | 118.795 | Marker683552 | 0.0634644 | -0.039050235 | 0.095941261 | 0.615706148 |
| 2847 | 6 | 118.795 | Marker683230 | 0.0634644 | -0.039050235 | 0.095941261 | 0.615706148 |
| 2848 | 6 | 119.045 | Marker681798 | 0.038639536 | -0.028846154 | 0.076417004 | 0.376036333 |
| 2849 | 6 | 119.295 | Marker681914 | 0.01057329 | -0.037735849 | 0.058008832 | 0.318925607 |
| 2850 | 6 | 119.545 | Marker681913 | 0.00756531 | -0.037735849 | 0.057000993 | 0.313268285 |
| 2851 | 6 | 119.545 | Marker681676 | 0.00756531 | -0.037735849 | 0.057000993 | 0.313268285 |
| 2852 | 6 | 119.545 | Marker681912 | 0.00756531 | -0.037735849 | 0.057000993 | 0.313268285 |
| 2853 | 6 | 119.795 | Marker681318 | 0.008879883 | -0.037037037 | 0.058707644 | 0.317233283 |
| 2854 | 6 | 119.795 | Marker681053 | 0.008879883 | -0.037037037 | 0.058707644 | 0.317233283 |
| 2855 | 6 | 120.045 | Marker681059 | 0.008368617 | -0.037037037 | 0.059737157 | 0.323188307 |
| 2856 | 6 | 120.295 | Marker681058 | 0.00788617 | -0.037037037 | 0.06078905 | 0.329380963 |
| 2857 | 6 | 120.545 | Marker681174 | 0.032818467 | -0.037037037 | 0.061864062 | 0.335822689 |
| 2858 | 6 | 120.545 | Marker680583 | 0.032818467 | -0.037037037 | 0.061864062 | 0.335822689 |
| 2859 | 6 | 120.795 | Marker681456 | 0.00751257 | -0.027603075 | 0.04241789 | 0.170585939 |
| 2860 | 6 | 120.795 | Marker681178 | 0.00751257 | -0.027603075 | 0.04241789 | 0.170585939 |
| 2861 | 6 | 120.795 | Marker682098 | 0.00751257 | -0.027603075 | 0.04241789 | 0.170585939 |
| 2862 | 6 | 120.795 | Marker682099 | 0.00751257 | -0.027603075 | 0.04241789 | 0.170585939 |
| 2863 | 6 | 121.045 | Marker685057 | 0.007802174 | -0.017838765 | 0.064416906 | 0.237928344 |
| 2864 | 6 | 121.045 | Marker681451 | 0.007802174 | -0.017838765 | 0.064416906 | 0.237928344 |
| 2865 | 6 | 121.295 | Marker685059 | 0.004399171 | -0.018013468 | 0.065740741 | 0.247066553 |
| 2866 | 6 | 123.597 | Marker685058 | 0.004601801 | -0.027931927 | 0.038481735 | 0.157037874 |
| 2867 | 6 | 124.347 | Marker686545 | 0.011328223 | -0.028120464 | 0.039605288 | 0.16246429 |
| 2868 | 6 | 124.347 | Marker686546 | 0.011328223 | -0.028120464 | 0.039605288 | 0.16246429 |
| 2869 | 6 | 124.347 | Marker688780 | 0.011328223 | -0.028120464 | 0.039605288 | 0.16246429 |
| 2870 | 6 | 124.347 | Marker685627 | 0.011328223 | -0.028120464 | 0.039605288 | 0.16246429 |
| 2871 | 6 | 124.597 | Marker688779 | 0.01212716 | -0.037735849 | 0.06116525 | 0.337293015 |
| 2872 | 6 | 126.449 | Marker688776 | 0.002280291 | -0.039411765 | 0.095171569 | 0.611551369 |
| 2873 | 6 | 126.449 | Marker688777 | 0.002280291 | -0.039411765 | 0.095171569 | 0.611551369 |
| 2874 | 6 | 126.449 | Marker688775 | 0.002280291 | -0.039411765 | 0.095171569 | 0.611551369 |
| 2875 | 6 | 126.449 | Marker690435 | 0.002280291 | -0.039411765 | 0.095171569 | 0.611551369 |
| 2876 | 6 | 126.449 | Marker689073 | 0.002280291 | -0.039411765 | 0.095171569 | 0.611551369 |
| 2877 | 6 | 126.949 | Marker691509 | 0.012176833 | -0.049607843 | 0.114607843 | 0.909325987 |
| 2878 | 6 | 127.949 | Marker691508 | 0.054106944 | -0.039607843 | 0.13502451 | 1.063599455 |
| 2879 | 6 | 128.449 | Marker692170 | 0.024689106 | -0.039411765 | 0.095171569 | 0.611551369 |
| 2880 | 6 | 128.699 | Marker692171 | 0.02439046 | -0.039215686 | 0.096181631 | 0.619366238 |
| 2881 | 6 | 128.949 | Marker692335 | 0.039214937 | -0.048642534 | 0.117478579 | 0.931756372 |
| 2882 | 6 | 129.699 | Marker692338 | 0.039851796 | -0.049019608 | 0.116511868 | 0.924637656 |
| 2883 | 6 | 129.699 | Marker690303 | 0.039851796 | -0.049019608 | 0.116511868 | 0.924637656 |
| 2884 | 6 | 129.949 | Marker692931 | 0.008647279 | -0.058069382 | 0.098167581 | 0.836710254 |
| 2885 | 6 | 129.949 | Marker692337 | 0.008647279 | -0.058069382 | 0.098167581 | 0.836710254 |
| 2886 | 6 | 129.949 | Marker692877 | 0.008647279 | -0.058069382 | 0.098167581 | 0.836710254 |
| 2887 | 6 | 130.199 | Marker692929 | 0.001373119 | -0.048461538 | 0.076801619 | 0.543013016 |
| 2888 | 6 | 130.199 | Marker692928 | 0.001373119 | -0.048461538 | 0.076801619 | 0.543013016 |
| 2889 | 6 | 130.199 | Marker692926 | 0.001373119 | -0.048461538 | 0.076801619 | 0.543013016 |
| 2890 | 6 | 130.199 | Marker692208 | 0.001373119 | -0.048461538 | 0.076801619 | 0.543013016 |
| 2891 | 6 | 130.199 | Marker692927 | 0.001373119 | -0.048461538 | 0.076801619 | 0.543013016 |
| 2892 | 6 | 130.449 | Marker692364 | 0.001297276 | -0.049215686 | 0.07495098 | 0.537302748 |
| 2893 | 6 | 130.449 | Marker684652 | 0.001297276 | -0.049215686 | 0.07495098 | 0.537302748 |
| 2894 | 6 | 130.449 | Marker692971 | 0.001297276 | -0.049215686 | 0.07495098 | 0.537302748 |
| 2895 | 6 | 130.449 | Marker692363 | 0.001297276 | -0.049215686 | 0.07495098 | 0.537302748 |
| 2896 | 6 | 130.449 | Marker684721 | 0.001297276 | -0.049215686 | 0.07495098 | 0.537302748 |
| 2897 | 6 | 130.699 | Marker693699 | 0.003538402 | -0.058076923 | 0.056659919 | 0.523696418 |
| 2898 | 6 | 130.699 | Marker693466 | 0.003538402 | -0.058076923 | 0.056659919 | 0.523696418 |
| 2899 | 6 | 130.949 | Marker693356 | 0.004036379 | -0.048076923 | 0.03702946 | 0.318796908 |
| 2900 | 6 | 130.949 | Marker694237 | 0.004036379 | -0.048076923 | 0.03702946 | 0.318796908 |
| 2901 | 6 | 130.949 | Marker693353 | 0.004036379 | -0.048076923 | 0.03702946 | 0.318796908 |
| 2902 | 6 | 130.949 | Marker693354 | 0.004036379 | -0.048076923 | 0.03702946 | 0.318796908 |
| 2903 | 6 | 130.949 | Marker694419 | 0.004036379 | -0.048076923 | 0.03702946 | 0.318796908 |
| 2904 | 6 | 131.449 | Marker694738 | 0.008392534 | -0.065724667 | 0.076694329 | 0.756665999 |
| 2905 | 6 | 131.949 | Marker694737 | 0.009108585 | -0.066944848 | 0.079486654 | 0.795510499 |
| 2906 | 6 | 132.95 | Marker694937 | 0.023475214 | -0.049433962 | 0.075816941 | 0.546014227 |
| 2907 | 6 | 133.2 | Marker694882 | 0.022413613 | -0.049259259 | 0.076786141 | 0.551361619 |
| 2908 | 6 | 133.7 | Marker695125 | 0.012540378 | -0.040816327 | 0.055957867 | 0.333826487 |
| 2909 | 6 | 133.95 | Marker697399 | 0.014362807 | -0.052083333 | 0.03302305 | 0.34919119 |
| 2910 | 6 | 134.45 | Marker695873 | 0.008933227 | -0.044262918 | 0.012348024 | 0.222092278 |
| 2911 | 6 | 134.45 | Marker695943 | 0.008933227 | -0.044262918 | 0.012348024 | 0.222092278 |
| 2912 | 6 | 134.45 | Marker697398 | 0.008933227 | -0.044262918 | 0.012348024 | 0.222092278 |
| 2913 | 6 | 134.45 | Marker695871 | 0.008933227 | -0.044262918 | 0.012348024 | 0.222092278 |
| 2914 | 6 | 135.45 | Marker701316 | 0.075940854 | -0.05994898 | 0.05573425 | 0.542773357 |
| 2915 | 6 | 135.95 | Marker698898 | 0.1014209 | -0.068877551 | 0.075532387 | 0.794323203 |
| 2916 | 6 | 136.2 | Marker701536 | 0.136283881 | -0.057857143 | 0.098736264 | 0.839513606 |
| 2917 | 6 | 136.551 | Marker701003 | 0.158452372 | -0.05754386 | 0.099766082 | 0.845584053 |
| 2918 | 6 | 136.551 | Marker701385 | 0.158452372 | -0.05754386 | 0.099766082 | 0.845584053 |
| 2919 | 6 | 136.801 | Marker701002 | 0.076050909 | -0.057857143 | 0.098736264 | 0.839513606 |
| 2920 | 6 | 137.301 | Marker698574 | 0.076748746 | -0.057857143 | 0.098736264 | 0.839513606 |
| 2921 | 6 | 137.301 | Marker698573 | 0.076748746 | -0.057857143 | 0.098736264 | 0.839513606 |
| 2922 | 6 | 137.551 | Marker698567 | 0.06532539 | -0.068877551 | 0.075532387 | 0.794323203 |
| 2923 | 6 | 137.551 | Marker698102 | 0.06532539 | -0.068877551 | 0.075532387 | 0.794323203 |
| 2924 | 6 | 137.551 | Marker698106 | 0.06532539 | -0.068877551 | 0.075532387 | 0.794323203 |
| 2925 | 6 | 138.801 | Marker698100 | 0.103062071 | -0.089526377 | 0.072340555 | 1.128543869 |
| 2926 | 6 | 138.801 | Marker698104 | 0.103062071 | -0.089526377 | 0.072340555 | 1.128543869 |
| 2927 | 6 | 138.801 | Marker698105 | 0.103062071 | -0.089526377 | 0.072340555 | 1.128543869 |
| 2928 | 6 | 138.801 | Marker698101 | 0.103062071 | -0.089526377 | 0.072340555 | 1.128543869 |
| 2929 | 6 | 139.051 | Marker698118 | 0.198695027 | -0.089002268 | 0.072936267 | 1.122497378 |
| 2930 | 6 | 139.051 | Marker698117 | 0.198695027 | -0.089002268 | 0.072936267 | 1.122497378 |
| 2931 | 6 | 139.051 | Marker701973 | 0.198695027 | -0.089002268 | 0.072936267 | 1.122497378 |
| 2932 | 6 | 139.051 | Marker697976 | 0.198695027 | -0.089002268 | 0.072936267 | 1.122497378 |
| 2933 | 6 | 139.051 | Marker698116 | 0.198695027 | -0.089002268 | 0.072936267 | 1.122497378 |
| 2934 | 6 | 139.051 | Marker701975 | 0.198695027 | -0.089002268 | 0.072936267 | 1.122497378 |
| 2935 | 6 | 139.051 | Marker698015 | 0.198695027 | -0.089002268 | 0.072936267 | 1.122497378 |
| 2936 | 6 | 139.051 | Marker698005 | 0.198695027 | -0.089002268 | 0.072936267 | 1.122497378 |
| 2937 | 6 | 139.051 | Marker698114 | 0.198695027 | -0.089002268 | 0.072936267 | 1.122497378 |
| 2938 | 6 | 139.051 | Marker697975 | 0.198695027 | -0.089002268 | 0.072936267 | 1.122497378 |
| 2939 | 6 | 139.051 | Marker698119 | 0.198695027 | -0.089002268 | 0.072936267 | 1.122497378 |
| 2940 | 6 | 139.051 | Marker698014 | 0.198695027 | -0.089002268 | 0.072936267 | 1.122497378 |
| 2941 | 6 | 140.051 | Marker701971 | 0.206216142 | -0.071780148 | 0.107272606 | 1.121349525 |
| 2942 | 6 | 140.051 | Marker701974 | 0.206216142 | -0.071780148 | 0.107272606 | 1.121349525 |
| 2943 | 6 | 140.051 | Marker701972 | 0.206216142 | -0.071780148 | 0.107272606 | 1.121349525 |
| 2944 | 6 | 140.301 | Marker701965 | 0.207101293 | -0.072746331 | 0.105069564 | 1.113703871 |
| 2945 | 6 | 140.301 | Marker704994 | 0.207101293 | -0.072746331 | 0.105069564 | 1.113703871 |
| 2946 | 6 | 140.301 | Marker704995 | 0.207101293 | -0.072746331 | 0.105069564 | 1.113703871 |
| 2947 | 6 | 141.051 | Marker705047 | 0.31463862 | -0.071399345 | 0.108741942 | 1.130915385 |
| 2948 | 6 | 141.551 | Marker705166 | 0.276018111 | -0.084401709 | 0.083290598 | 1.113663402 |
| 2949 | 6 | 141.801 | Marker704152 | 0.239999391 | -0.075163399 | 0.063062189 | 0.809395695 |
| 2950 | 6 | 142.051 | Marker705165 | 0.224837666 | -0.087789661 | 0.039661319 | 0.919303118 |
| 2951 | 6 | 142.301 | Marker705171 | 0.332303973 | -0.088181818 | 0.039196823 | 0.925132399 |
| 2952 | 6 | 142.551 | Marker705489 | 0.52141087 | -0.098794063 | 0.058584273 | 1.232842499 |
| 2953 | 6 | 142.551 | Marker705170 | 0.52141087 | -0.098794063 | 0.058584273 | 1.232842499 |
| 2954 | 6 | 142.551 | Marker706375 | 0.52141087 | -0.098794063 | 0.058584273 | 1.232842499 |
| 2955 | 6 | 142.801 | Marker706373 | 0.563065579 | -0.0861678 | 0.081852806 | 1.135033145 |
| 2956 | 6 | 143.051 | Marker706609 | 0.575381505 | -0.086805556 | 0.081463675 | 1.143996723 |
| 2957 | 6 | 143.051 | Marker709383 | 0.575381505 | -0.086805556 | 0.081463675 | 1.143996723 |
| 2958 | 6 | 143.051 | Marker709381 | 0.575381505 | -0.086805556 | 0.081463675 | 1.143996723 |
| 2959 | 6 | 143.301 | Marker710017 | 0.563065579 | -0.0861678 | 0.081852806 | 1.135033145 |
| 2960 | 6 | 143.301 | Marker709157 | 0.563065579 | -0.0861678 | 0.081852806 | 1.135033145 |
| 2961 | 6 | 143.551 | Marker708291 | 0.608796017 | -0.07606679 | 0.100542315 | 1.122069913 |
| 2962 | 6 | 143.551 | Marker707855 | 0.608796017 | -0.07606679 | 0.100542315 | 1.122069913 |
| 2963 | 6 | 143.551 | Marker705838 | 0.608796017 | -0.07606679 | 0.100542315 | 1.122069913 |
| 2964 | 6 | 143.551 | Marker708065 | 0.608796017 | -0.07606679 | 0.100542315 | 1.122069913 |
| 2965 | 6 | 143.551 | Marker706469 | 0.608796017 | -0.07606679 | 0.100542315 | 1.122069913 |
| 2966 | 6 | 143.551 | Marker706470 | 0.608796017 | -0.07606679 | 0.100542315 | 1.122069913 |
| 2967 | 6 | 143.551 | Marker707224 | 0.608796017 | -0.07606679 | 0.100542315 | 1.122069913 |
| 2968 | 6 | 143.551 | Marker708292 | 0.608796017 | -0.07606679 | 0.100542315 | 1.122069913 |
| 2969 | 6 | 143.551 | Marker707227 | 0.608796017 | -0.07606679 | 0.100542315 | 1.122069913 |
| 2970 | 6 | 143.551 | Marker708063 | 0.608796017 | -0.07606679 | 0.100542315 | 1.122069913 |
| 2971 | 6 | 143.551 | Marker706983 | 0.608796017 | -0.07606679 | 0.100542315 | 1.122069913 |
| 2972 | 6 | 143.551 | Marker709147 | 0.608796017 | -0.07606679 | 0.100542315 | 1.122069913 |
| 2973 | 6 | 143.551 | Marker709064 | 0.608796017 | -0.07606679 | 0.100542315 | 1.122069913 |
| 2974 | 6 | 143.551 | Marker707854 | 0.608796017 | -0.07606679 | 0.100542315 | 1.122069913 |
| 2975 | 6 | 143.551 | Marker708293 | 0.608796017 | -0.07606679 | 0.100542315 | 1.122069913 |
| 2976 | 6 | 143.551 | Marker706926 | 0.608796017 | -0.07606679 | 0.100542315 | 1.122069913 |
| 2977 | 6 | 143.551 | Marker708413 | 0.608796017 | -0.07606679 | 0.100542315 | 1.122069913 |
| 2978 | 6 | 143.551 | Marker707539 | 0.608796017 | -0.07606679 | 0.100542315 | 1.122069913 |
| 2979 | 6 | 143.551 | Marker708290 | 0.608796017 | -0.07606679 | 0.100542315 | 1.122069913 |
| 2980 | 6 | 143.551 | Marker708837 | 0.608796017 | -0.07606679 | 0.100542315 | 1.122069913 |
| 2981 | 6 | 143.551 | Marker708570 | 0.608796017 | -0.07606679 | 0.100542315 | 1.122069913 |
| 2982 | 6 | 143.551 | Marker709636 | 0.608796017 | -0.07606679 | 0.100542315 | 1.122069913 |
| 2983 | 6 | 143.551 | Marker707538 | 0.608796017 | -0.07606679 | 0.100542315 | 1.122069913 |
| 2984 | 6 | 143.551 | Marker708064 | 0.608796017 | -0.07606679 | 0.100542315 | 1.122069913 |
| 2985 | 6 | 143.551 | Marker708294 | 0.608796017 | -0.07606679 | 0.100542315 | 1.122069913 |
| 2986 | 6 | 143.551 | Marker705837 | 0.608796017 | -0.07606679 | 0.100542315 | 1.122069913 |
| 2987 | 6 | 144.052 | Marker709156 | 0.421706897 | -0.07606679 | 0.100542315 | 1.122069913 |
| 2988 | 6 | 144.052 | Marker709158 | 0.421706897 | -0.07606679 | 0.100542315 | 1.122069913 |
| 2989 | 6 | 144.052 | Marker709029 | 0.421706897 | -0.07606679 | 0.100542315 | 1.122069913 |
| 2990 | 6 | 144.302 | Marker711699 | 0.420812301 | -0.075056689 | 0.102672655 | 1.126557488 |
| 2991 | 6 | 144.302 | Marker709340 | 0.420812301 | -0.075056689 | 0.102672655 | 1.126557488 |
| 2992 | 6 | 144.552 | Marker709646 | 0.467224881 | -0.084444444 | 0.123006536 | 1.513125166 |
| 2993 | 6 | 144.802 | Marker711698 | 0.459160627 | -0.083660131 | 0.123484113 | 1.504538527 |
| 2994 | 6 | 145.302 | Marker711602 | 0.579375772 | -0.074099407 | 0.140254295 | 1.557004212 |
| 2995 | 6 | 145.302 | Marker711600 | 0.579375772 | -0.074099407 | 0.140254295 | 1.557004212 |
| 2996 | 6 | 145.302 | Marker711601 | 0.579375772 | -0.074099407 | 0.140254295 | 1.557004212 |
| 2997 | 6 | 145.302 | Marker714691 | 0.579375772 | -0.074099407 | 0.140254295 | 1.557004212 |
| 2998 | 6 | 145.302 | Marker711435 | 0.579375772 | -0.074099407 | 0.140254295 | 1.557004212 |
| 2999 | 6 | 145.302 | Marker711436 | 0.579375772 | -0.074099407 | 0.140254295 | 1.557004212 |
| 3000 | 6 | 145.302 | Marker714690 | 0.579375772 | -0.074099407 | 0.140254295 | 1.557004212 |
| 3001 | 6 | 146.052 | Marker714688 | 0.646000921 | -0.060954817 | 0.167476556 | 1.775918681 |
| 3002 | 6 | 146.302 | Marker714458 | 0.701271371 | -0.069816054 | 0.187953954 | 2.257685251 |
| 3003 | 6 | 146.302 | Marker714466 | 0.701271371 | -0.069816054 | 0.187953954 | 2.257685251 |
| 3004 | 6 | 146.302 | Marker714687 | 0.701271371 | -0.069816054 | 0.187953954 | 2.257685251 |
| 3005 | 6 | 147.052 | Marker716410 | 0.535448693 | -0.091025641 | 0.184358974 | 2.560192363 |
| 3006 | 6 | 147.552 | Marker712081 | 0.194772972 | -0.073333333 | 0.143921569 | 1.595772434 |
| 3007 | 6 | 148.302 | Marker721690 | 0.40601106 | -0.07273122 | 0.147515294 | 1.637523309 |
| 3008 | 6 | 148.552 | Marker721694 | 0.411468154 | -0.07358156 | 0.147006675 | 1.643649663 |
| 3009 | 6 | 149.302 | Marker721691 | 0.346774249 | -0.085106383 | 0.126213592 | 1.56431468 |
| 3010 | 6 | 149.802 | Marker721029 | 0.311360056 | -0.086956522 | 0.123809524 | 1.569597248 |
| 3011 | 6 | 149.802 | Marker721293 | 0.311360056 | -0.086956522 | 0.123809524 | 1.569597248 |
| 3012 | 6 | 149.802 | Marker721747 | 0.311360056 | -0.086956522 | 0.123809524 | 1.569597248 |
| 3013 | 6 | 149.802 | Marker721294 | 0.311360056 | -0.086956522 | 0.123809524 | 1.569597248 |
| 3014 | 6 | 149.802 | Marker721808 | 0.311360056 | -0.086956522 | 0.123809524 | 1.569597248 |
| 3015 | 6 | 150.302 | Marker721290 | 0.351539898 | -0.075886525 | 0.142553191 | 1.617839515 |
| 3016 | 6 | 150.302 | Marker721142 | 0.351539898 | -0.075886525 | 0.142553191 | 1.617839515 |
| 3017 | 6 | 150.802 | Marker720157 | 0.663171565 | -0.065217391 | 0.164596273 | 1.786872156 |
| 3018 | 6 | 151.552 | Marker719625 | 0.590178258 | -0.055112738 | 0.145058027 | 1.35918717 |
| 3019 | 6 | 152.052 | Marker719735 | 0.599405914 | -0.054004678 | 0.145855648 | 1.357564177 |
| 3020 | 6 | 152.552 | Marker719476 | 0.580986066 | -0.055367505 | 0.144889785 | 1.359823212 |
| 3021 | 6 | 153.052 | Marker716992 | 0.539867043 | -0.04508612 | 0.122903298 | 0.959826172 |
| 3022 | 6 | 153.302 | Marker716638 | 0.464415289 | -0.032904503 | 0.102500844 | 0.632049982 |
| 3023 | 6 | 153.802 | Marker716637 | 0.915817043 | -0.056160317 | 0.144448247 | 1.363074716 |
| 3024 | 6 | 153.802 | Marker722882 | 0.915817043 | -0.056160317 | 0.144448247 | 1.363074716 |
| 3025 | 6 | 154.302 | Marker723606 | 0.945683651 | -0.054924242 | 0.146590909 | 1.378889469 |
| 3026 | 6 | 154.802 | Marker725152 | 1.061345792 | -0.034090909 | 0.186471861 | 1.83422903 |
| 3027 | 6 | 154.802 | Marker725352 | 1.061345792 | -0.034090909 | 0.186471861 | 1.83422903 |
| 3028 | 6 | 154.802 | Marker726061 | 1.061345792 | -0.034090909 | 0.186471861 | 1.83422903 |
| 3029 | 6 | 154.802 | Marker726060 | 1.061345792 | -0.034090909 | 0.186471861 | 1.83422903 |
| 3030 | 6 | 154.802 | Marker726059 | 1.061345792 | -0.034090909 | 0.186471861 | 1.83422903 |
| 3031 | 6 | 154.802 | Marker725354 | 1.061345792 | -0.034090909 | 0.186471861 | 1.83422903 |
| 3032 | 6 | 155.052 | Marker726959 | 1.018056479 | -0.034090909 | 0.186968097 | 1.84335402 |
| 3033 | 6 | 155.552 | Marker725151 | 1.61100997 | -0.044444444 | 0.207905983 | 2.336065745 |
| 3034 | 6 | 155.552 | Marker716636 | 1.61100997 | -0.044444444 | 0.207905983 | 2.336065745 |
| 3035 | 6 | 156.052 | Marker726889 | 1.000725555 | -0.044729207 | 0.164962045 | 1.551128932 |
| 3036 | 6 | 156.052 | Marker724457 | 1.000725555 | -0.044729207 | 0.164962045 | 1.551128932 |
| 3037 | 6 | 156.052 | Marker727077 | 1.000725555 | -0.044729207 | 0.164962045 | 1.551128932 |
| 3038 | 6 | 156.052 | Marker727241 | 1.000725555 | -0.044729207 | 0.164962045 | 1.551128932 |
| 3039 | 6 | 156.052 | Marker724362 | 1.000725555 | -0.044729207 | 0.164962045 | 1.551128932 |
| 3040 | 6 | 156.052 | Marker723819 | 1.000725555 | -0.044729207 | 0.164962045 | 1.551128932 |
| 3041 | 6 | 156.052 | Marker727066 | 1.000725555 | -0.044729207 | 0.164962045 | 1.551128932 |
| 3042 | 6 | 156.052 | Marker724512 | 1.000725555 | -0.044729207 | 0.164962045 | 1.551128932 |
| 3043 | 6 | 156.052 | Marker726499 | 1.000725555 | -0.044729207 | 0.164962045 | 1.551128932 |
| 3044 | 6 | 156.052 | Marker724456 | 1.000725555 | -0.044729207 | 0.164962045 | 1.551128932 |
| 3045 | 6 | 156.052 | Marker727081 | 1.000725555 | -0.044729207 | 0.164962045 | 1.551128932 |
| 3046 | 6 | 156.052 | Marker726957 | 1.000725555 | -0.044729207 | 0.164962045 | 1.551128932 |
| 3047 | 6 | 156.052 | Marker724513 | 1.000725555 | -0.044729207 | 0.164962045 | 1.551128932 |
| 3048 | 6 | 156.052 | Marker727067 | 1.000725555 | -0.044729207 | 0.164962045 | 1.551128932 |
| 3049 | 6 | 156.052 | Marker727078 | 1.000725555 | -0.044729207 | 0.164962045 | 1.551128932 |
| 3050 | 6 | 156.052 | Marker725117 | 1.000725555 | -0.044729207 | 0.164962045 | 1.551128932 |
| 3051 | 6 | 156.052 | Marker726498 | 1.000725555 | -0.044729207 | 0.164962045 | 1.551128932 |
| 3052 | 6 | 156.052 | Marker723822 | 1.000725555 | -0.044729207 | 0.164962045 | 1.551128932 |
| 3053 | 6 | 156.052 | Marker724517 | 1.000725555 | -0.044729207 | 0.164962045 | 1.551128932 |
| 3054 | 6 | 156.052 | Marker725298 | 1.000725555 | -0.044729207 | 0.164962045 | 1.551128932 |
| 3055 | 6 | 156.052 | Marker726264 | 1.000725555 | -0.044729207 | 0.164962045 | 1.551128932 |
| 3056 | 6 | 156.052 | Marker725686 | 1.000725555 | -0.044729207 | 0.164962045 | 1.551128932 |
| 3057 | 6 | 156.052 | Marker724732 | 1.000725555 | -0.044729207 | 0.164962045 | 1.551128932 |
| 3058 | 6 | 156.052 | Marker726958 | 1.000725555 | -0.044729207 | 0.164962045 | 1.551128932 |
| 3059 | 6 | 156.052 | Marker725683 | 1.000725555 | -0.044729207 | 0.164962045 | 1.551128932 |
| 3060 | 6 | 156.052 | Marker726887 | 1.000725555 | -0.044729207 | 0.164962045 | 1.551128932 |
| 3061 | 6 | 156.052 | Marker723834 | 1.000725555 | -0.044729207 | 0.164962045 | 1.551128932 |
| 3062 | 6 | 156.052 | Marker726156 | 1.000725555 | -0.044729207 | 0.164962045 | 1.551128932 |
| 3063 | 6 | 156.052 | Marker724412 | 1.000725555 | -0.044729207 | 0.164962045 | 1.551128932 |
| 3064 | 6 | 156.052 | Marker726888 | 1.000725555 | -0.044729207 | 0.164962045 | 1.551128932 |
| 3065 | 6 | 156.052 | Marker725960 | 1.000725555 | -0.044729207 | 0.164962045 | 1.551128932 |
| 3066 | 6 | 156.052 | Marker723818 | 1.000725555 | -0.044729207 | 0.164962045 | 1.551128932 |
| 3067 | 6 | 156.052 | Marker725963 | 1.000725555 | -0.044729207 | 0.164962045 | 1.551128932 |
| 3068 | 6 | 156.052 | Marker724355 | 1.000725555 | -0.044729207 | 0.164962045 | 1.551128932 |
| 3069 | 6 | 156.052 | Marker723836 | 1.000725555 | -0.044729207 | 0.164962045 | 1.551128932 |
| 3070 | 6 | 156.052 | Marker724455 | 1.000725555 | -0.044729207 | 0.164962045 | 1.551128932 |
| 3071 | 6 | 156.052 | Marker726265 | 1.000725555 | -0.044729207 | 0.164962045 | 1.551128932 |
| 3072 | 6 | 156.052 | Marker724585 | 1.000725555 | -0.044729207 | 0.164962045 | 1.551128932 |
| 3073 | 6 | 156.052 | Marker724354 | 1.000725555 | -0.044729207 | 0.164962045 | 1.551128932 |
| 3074 | 6 | 156.052 | Marker725877 | 1.000725555 | -0.044729207 | 0.164962045 | 1.551128932 |
| 3075 | 6 | 156.552 | Marker723662 | 0.950905055 | -0.055367505 | 0.144889785 | 1.359823212 |
| 3076 | 6 | 156.552 | Marker725961 | 0.950905055 | -0.055367505 | 0.144889785 | 1.359823212 |
| 3077 | 6 | 156.552 | Marker723661 | 0.950905055 | -0.055367505 | 0.144889785 | 1.359823212 |
| 3078 | 6 | 157.052 | Marker724223 | 1.04827799 | -0.068895643 | 0.165231341 | 1.850308146 |
| 3079 | 6 | 157.052 | Marker722883 | 1.04827799 | -0.068895643 | 0.165231341 | 1.850308146 |
| 3080 | 6 | 157.052 | Marker722881 | 1.04827799 | -0.068895643 | 0.165231341 | 1.850308146 |
| 3081 | 6 | 157.052 | Marker723428 | 1.04827799 | -0.068895643 | 0.165231341 | 1.850308146 |
| 3082 | 6 | 157.052 | Marker723113 | 1.04827799 | -0.068895643 | 0.165231341 | 1.850308146 |
| 3083 | 6 | 157.302 | Marker717440 | 1.10848172 | -0.058035714 | 0.186735091 | 2.073591414 |
| 3084 | 6 | 157.302 | Marker719595 | 1.10848172 | -0.058035714 | 0.186735091 | 2.073591414 |
| 3085 | 6 | 157.302 | Marker722754 | 1.10848172 | -0.058035714 | 0.186735091 | 2.073591414 |
| 3086 | 6 | 157.302 | Marker722753 | 1.10848172 | -0.058035714 | 0.186735091 | 2.073591414 |
| 3087 | 6 | 157.302 | Marker719598 | 1.10848172 | -0.058035714 | 0.186735091 | 2.073591414 |
| 3088 | 6 | 157.302 | Marker717616 | 1.10848172 | -0.058035714 | 0.186735091 | 2.073591414 |
| 3089 | 6 | 157.302 | Marker722755 | 1.10848172 | -0.058035714 | 0.186735091 | 2.073591414 |
| 3090 | 6 | 157.552 | Marker717571 | 1.145404526 | -0.056928295 | 0.187038357 | 2.065531254 |
| 3091 | 6 | 157.552 | Marker716676 | 1.145404526 | -0.056928295 | 0.187038357 | 2.065531254 |
| 3092 | 6 | 157.552 | Marker719594 | 1.145404526 | -0.056928295 | 0.187038357 | 2.065531254 |
| 3093 | 6 | 157.552 | Marker717572 | 1.145404526 | -0.056928295 | 0.187038357 | 2.065531254 |
| 3094 | 6 | 157.802 | Marker723112 | 1.085258794 | -0.067788224 | 0.165421948 | 1.837079369 |
| 3095 | 6 | 158.052 | Marker717119 | 1.072638766 | -0.066731141 | 0.165687384 | 1.826064146 |
| 3096 | 6 | 158.302 | Marker717118 | 1.143662345 | -0.076832151 | 0.186659912 | 2.344706049 |
| 3097 | 6 | 158.302 | Marker719302 | 1.143662345 | -0.076832151 | 0.186659912 | 2.344706049 |
| 3098 | 6 | 158.802 | Marker720394 | 1.094705418 | -0.076832151 | 0.186659912 | 2.344706049 |
| 3099 | 6 | 158.802 | Marker716927 | 1.094705418 | -0.076832151 | 0.186659912 | 2.344706049 |
| 3100 | 6 | 158.802 | Marker716926 | 1.094705418 | -0.076832151 | 0.186659912 | 2.344706049 |
| 3101 | 6 | 158.802 | Marker719301 | 1.094705418 | -0.076832151 | 0.186659912 | 2.344706049 |
| 3102 | 6 | 158.802 | Marker719303 | 1.094705418 | -0.076832151 | 0.186659912 | 2.344706049 |
| 3103 | 6 | 159.552 | Marker720915 | 0.66887572 | -0.066183575 | 0.164214748 | 1.794397492 |
| 3104 | 6 | 159.552 | Marker721292 | 0.66887572 | -0.066183575 | 0.164214748 | 1.794397492 |
| 3105 | 6 | 159.802 | Marker720917 | 0.663171565 | -0.065217391 | 0.164596273 | 1.786872156 |
| 3106 | 6 | 159.802 | Marker720916 | 0.663171565 | -0.065217391 | 0.164596273 | 1.786872156 |
| 3107 | 6 | 159.802 | Marker720393 | 0.663171565 | -0.065217391 | 0.164596273 | 1.786872156 |
| 3108 | 6 | 160.302 | Marker721978 | 0.35903199 | -0.076750455 | 0.142387667 | 1.629815667 |
| 3109 | 6 | 160.552 | Marker721140 | 0.35754334 | -0.076811594 | 0.142220399 | 1.62850413 |
| 3110 | 6 | 160.552 | Marker721827 | 0.35754334 | -0.076811594 | 0.142220399 | 1.62850413 |
| 3111 | 6 | 160.552 | Marker721141 | 0.35754334 | -0.076811594 | 0.142220399 | 1.62850413 |
| 3112 | 6 | 160.552 | Marker721809 | 0.35754334 | -0.076811594 | 0.142220399 | 1.62850413 |
| 3113 | 6 | 161.302 | Marker722492 | 0.21713532 | -0.085144928 | 0.124401998 | 1.542878256 |
| 3114 | 6 | 161.552 | Marker722436 | 0.213001884 | -0.084294587 | 0.124789046 | 1.531925102 |
| 3115 | 6 | 162.052 | Marker716409 | 0.240688524 | -0.082482993 | 0.12914966 | 1.553149379 |
| 3116 | 6 | 162.052 | Marker722023 | 0.240688524 | -0.082482993 | 0.12914966 | 1.553149379 |
| 3117 | 6 | 162.802 | Marker712322 | 0.307195286 | -0.07113058 | 0.148714934 | 1.630021977 |
| 3118 | 6 | 162.802 | Marker716341 | 0.307195286 | -0.07113058 | 0.148714934 | 1.630021977 |
| 3119 | 6 | 162.802 | Marker722440 | 0.307195286 | -0.07113058 | 0.148714934 | 1.630021977 |
| 3120 | 6 | 162.802 | Marker716305 | 0.307195286 | -0.07113058 | 0.148714934 | 1.630021977 |
| 3121 | 6 | 163.802 | Marker714377 | 0.439199826 | -0.091555184 | 0.146012804 | 1.951154325 |
| 3122 | 6 | 163.802 | Marker712313 | 0.439199826 | -0.091555184 | 0.146012804 | 1.951154325 |
| 3123 | 6 | 163.802 | Marker712240 | 0.439199826 | -0.091555184 | 0.146012804 | 1.951154325 |
| 3124 | 6 | 163.802 | Marker712080 | 0.439199826 | -0.091555184 | 0.146012804 | 1.951154325 |
| 3125 | 6 | 163.802 | Marker711798 | 0.439199826 | -0.091555184 | 0.146012804 | 1.951154325 |
| 3126 | 6 | 164.052 | Marker714798 | 0.457645528 | -0.090648072 | 0.146548693 | 1.940869408 |
| 3127 | 6 | 164.052 | Marker714108 | 0.457645528 | -0.090648072 | 0.146548693 | 1.940869408 |
| 3128 | 6 | 164.302 | Marker715071 | 0.493542845 | -0.080503145 | 0.16535163 | 2.039542712 |
| 3129 | 6 | 164.302 | Marker715937 | 0.493542845 | -0.080503145 | 0.16535163 | 2.039542712 |
| 3130 | 6 | 164.802 | Marker714381 | 0.762283691 | -0.068636238 | 0.189961052 | 2.277343139 |
| 3131 | 6 | 165.302 | Marker714376 | 0.706087023 | -0.060200669 | 0.16823756 | 1.77863587 |
| 3132 | 6 | 165.802 | Marker714375 | 0.700033733 | -0.059474979 | 0.169057943 | 1.782886425 |
| 3133 | 6 | 166.302 | Marker712612 | 0.67962612 | -0.059958071 | 0.167028778 | 1.755612811 |
| 3134 | 6 | 166.302 | Marker714374 | 0.67962612 | -0.059958071 | 0.167028778 | 1.755612811 |
| 3135 | 6 | 166.803 | Marker710962 | 0.672483653 | -0.059259259 | 0.167876039 | 1.760587927 |
| 3136 | 6 | 166.803 | Marker712135 | 0.672483653 | -0.059259259 | 0.167876039 | 1.760587927 |
| 3137 | 6 | 167.154 | Marker710961 | 0.617938143 | -0.050505051 | 0.146464646 | 1.326997013 |
| 3138 | 6 | 167.404 | Marker710957 | 0.620337339 | -0.051033592 | 0.144521964 | 1.305011534 |
| 3139 | 6 | 167.904 | Marker710956 | 0.516113937 | -0.053896104 | 0.102467532 | 0.828250938 |
| 3140 | 6 | 168.154 | Marker710955 | 0.544280524 | -0.053065539 | 0.104510218 | 0.839323092 |
| 3141 | 6 | 168.654 | Marker718153 | 0.500061936 | -0.048827873 | 0.097612913 | 0.724363084 |
| 3142 | 6 | 168.904 | Marker711950 | 0.539984696 | -0.052740864 | 0.105422402 | 0.844978964 |
| 3143 | 6 | 169.154 | Marker711955 | 0.536182874 | -0.052427581 | 0.106371479 | 0.851254729 |
| 3144 | 6 | 169.404 | Marker718814 | 0.378664034 | -0.043504411 | 0.088320302 | 0.586607186 |
| 3145 | 6 | 169.404 | Marker718815 | 0.378664034 | -0.043504411 | 0.088320302 | 0.586607186 |
| 3146 | 6 | 169.904 | Marker691845 | 0.291113176 | -0.030822939 | 0.053819599 | 0.244604697 |
| 3147 | 6 | 170.155 | Marker691844 | 0.285925055 | -0.026550388 | 0.052527627 | 0.211329588 |
| 3148 | 6 | 171.06 | Marker702646 | 0.203389837 | -0.03968254 | 0.031400966 | 0.219691571 |
| 3149 | 6 | 171.912 | Marker702645 | 0.108658373 | -0.03968254 | 0.031400966 | 0.219691571 |
| 3150 | 7 | 0 | Marker728254 | 0.520084094 | -0.297395833 | 0.014725379 | 9.733665665 |
| 3151 | 7 | 0 | Marker728251 | 0.520084094 | -0.297395833 | 0.014725379 | 9.733665665 |
| 3152 | 7 | 0 | Marker728250 | 0.520084094 | -0.297395833 | 0.014725379 | 9.733665665 |
| 3153 | 7 | 3.104 | Marker728249 | 0.779916293 | -0.380482456 | 0.054517544 | 16.0366779 |
| 3154 | 7 | 3.604 | Marker731194 | 0.41169494 | -0.373342175 | 0.08127321 | 15.60641327 |
| 3155 | 7 | 3.604 | Marker731193 | 0.41169494 | -0.373342175 | 0.08127321 | 15.60641327 |
| 3156 | 7 | 4.605 | Marker727473 | 0.808741987 | -0.412644788 | 0.087095337 | 19.04338355 |
| 3157 | 7 | 4.605 | Marker727389 | 0.808741987 | -0.412644788 | 0.087095337 | 19.04338355 |
| 3158 | 7 | 4.605 | Marker727474 | 0.808741987 | -0.412644788 | 0.087095337 | 19.04338355 |
| 3159 | 7 | 5.605 | Marker731643 | 0.55216661 | -0.414377289 | 0.099951519 | 19.30981391 |
| 3160 | 7 | 6.105 | Marker736157 | 0.513854691 | -0.420932538 | 0.118304636 | 20.09484234 |
| 3161 | 7 | 6.355 | Marker735654 | 0.516852358 | -0.421428571 | 0.117680339 | 20.13390963 |
| 3162 | 7 | 6.355 | Marker735527 | 0.516852358 | -0.421428571 | 0.117680339 | 20.13390963 |
| 3163 | 7 | 6.355 | Marker735617 | 0.516852358 | -0.421428571 | 0.117680339 | 20.13390963 |
| 3164 | 7 | 6.355 | Marker735564 | 0.516852358 | -0.421428571 | 0.117680339 | 20.13390963 |
| 3165 | 7 | 6.355 | Marker735618 | 0.516852358 | -0.421428571 | 0.117680339 | 20.13390963 |
| 3166 | 7 | 6.355 | Marker735567 | 0.516852358 | -0.421428571 | 0.117680339 | 20.13390963 |
| 3167 | 7 | 6.355 | Marker736650 | 0.516852358 | -0.421428571 | 0.117680339 | 20.13390963 |
| 3168 | 7 | 6.355 | Marker735667 | 0.516852358 | -0.421428571 | 0.117680339 | 20.13390963 |
| 3169 | 7 | 6.355 | Marker736085 | 0.516852358 | -0.421428571 | 0.117680339 | 20.13390963 |
| 3170 | 7 | 6.855 | Marker736649 | 0.539792208 | -0.430769231 | 0.118147872 | 21.01277557 |
| 3171 | 7 | 7.605 | Marker737987 | 0.489742036 | -0.4375 | 0.135539216 | 21.85837356 |
| 3172 | 7 | 7.605 | Marker737988 | 0.489742036 | -0.4375 | 0.135539216 | 21.85837356 |
| 3173 | 7 | 8.105 | Marker738415 | 0.480601526 | -0.447606143 | 0.148041873 | 23.00496509 |
| 3174 | 7 | 8.605 | Marker735089 | 0.538496098 | -0.459090909 | 0.138948307 | 24.02624986 |
| 3175 | 7 | 9.605 | Marker737406 | 0.642891131 | -0.457875458 | 0.107735402 | 23.54966149 |
| 3176 | 7 | 9.605 | Marker737407 | 0.642891131 | -0.457875458 | 0.107735402 | 23.54966149 |
| 3177 | 7 | 9.855 | Marker739308 | 0.685022986 | -0.462887989 | 0.093598429 | 23.92781961 |
| 3178 | 7 | 9.855 | Marker739311 | 0.685022986 | -0.462887989 | 0.093598429 | 23.92781961 |
| 3179 | 7 | 9.855 | Marker739324 | 0.685022986 | -0.462887989 | 0.093598429 | 23.92781961 |
| 3180 | 7 | 9.855 | Marker737170 | 0.685022986 | -0.462887989 | 0.093598429 | 23.92781961 |
| 3181 | 7 | 10.105 | Marker739332 | 0.737905577 | -0.466804029 | 0.089002909 | 24.2901785 |
| 3182 | 7 | 10.355 | Marker739569 | 0.685048755 | -0.473214286 | 0.105993635 | 25.10109201 |
| 3183 | 7 | 10.605 | Marker739613 | 0.842169375 | -0.478070175 | 0.091929825 | 25.48316029 |
| 3184 | 7 | 10.855 | Marker739696 | 0.895898217 | -0.471659919 | 0.074925509 | 24.68890675 |
| 3185 | 7 | 11.355 | Marker739480 | 0.842169375 | -0.478070175 | 0.091929825 | 25.48316029 |
| 3186 | 7 | 11.605 | Marker739479 | 0.842169375 | -0.478070175 | 0.091929825 | 25.48316029 |
| 3187 | 7 | 12.605 | Marker740098 | 0.766925406 | -0.463580388 | 0.097723045 | 24.03376968 |
| 3188 | 7 | 13.105 | Marker740239 | 0.359275481 | -0.45530303 | 0.107991202 | 23.29422693 |
| 3189 | 7 | 13.105 | Marker740238 | 0.359275481 | -0.45530303 | 0.107991202 | 23.29422693 |
| 3190 | 7 | 13.355 | Marker740202 | 0.797539163 | -0.467449923 | 0.084290889 | 24.32015083 |
| 3191 | 7 | 13.355 | Marker740229 | 0.797539163 | -0.467449923 | 0.084290889 | 24.32015083 |
| 3192 | 7 | 13.355 | Marker740225 | 0.797539163 | -0.467449923 | 0.084290889 | 24.32015083 |
| 3193 | 7 | 13.855 | Marker740265 | 0.838073995 | -0.467449923 | 0.084290889 | 24.32015083 |
| 3194 | 7 | 15.356 | Marker741596 | 0.4034585 | -0.471072797 | 0.157059591 | 25.49869676 |
| 3195 | 7 | 16.106 | Marker741478 | 0.725145927 | -0.487780844 | 0.152366035 | 27.18897139 |
| 3196 | 7 | 16.606 | Marker742307 | 0.664478324 | -0.496865204 | 0.164378815 | 28.34766057 |
| 3197 | 7 | 17.106 | Marker742487 | 0.681321027 | -0.50487013 | 0.153986477 | 29.07364529 |
| 3198 | 7 | 17.106 | Marker742488 | 0.681321027 | -0.50487013 | 0.153986477 | 29.07364529 |
| 3199 | 7 | 17.106 | Marker742492 | 0.681321027 | -0.50487013 | 0.153986477 | 29.07364529 |
| 3200 | 7 | 17.106 | Marker742489 | 0.681321027 | -0.50487013 | 0.153986477 | 29.07364529 |
| 3201 | 7 | 18.656 | Marker743228 | 0.315932135 | -0.510833801 | 0.181156816 | 30.16292018 |
| 3202 | 7 | 18.906 | Marker743205 | 0.312470717 | -0.505847953 | 0.176023392 | 29.52102062 |
| 3203 | 7 | 19.156 | Marker743204 | 0.511932052 | -0.499809306 | 0.171328529 | 28.77853417 |
| 3204 | 7 | 20.156 | Marker743792 | 0.569187885 | -0.520454545 | 0.170176252 | 31.07121062 |
| 3205 | 7 | 20.156 | Marker743793 | 0.569187885 | -0.520454545 | 0.170176252 | 31.07121062 |
| 3206 | 7 | 20.906 | Marker744578 | 0.457204915 | -0.52483165 | 0.16456229 | 31.48578603 |
| 3207 | 7 | 20.906 | Marker744581 | 0.457204915 | -0.52483165 | 0.16456229 | 31.48578603 |
| 3208 | 7 | 20.906 | Marker744582 | 0.457204915 | -0.52483165 | 0.16456229 | 31.48578603 |
| 3209 | 7 | 21.406 | Marker744268 | 0.466097677 | -0.52483165 | 0.16456229 | 31.48578603 |
| 3210 | 7 | 21.406 | Marker743944 | 0.466097677 | -0.52483165 | 0.16456229 | 31.48578603 |
| 3211 | 7 | 21.406 | Marker743945 | 0.466097677 | -0.52483165 | 0.16456229 | 31.48578603 |
| 3212 | 7 | 21.406 | Marker744269 | 0.466097677 | -0.52483165 | 0.16456229 | 31.48578603 |
| 3213 | 7 | 21.906 | Marker745138 | 0.55616806 | -0.533210044 | 0.146302603 | 32.19595254 |
| 3214 | 7 | 22.657 | Marker744990 | 0.422101787 | -0.520933014 | 0.161832137 | 30.99696241 |
| 3215 | 7 | 23.407 | Marker745469 | 0.459365866 | -0.53615222 | 0.15635424 | 32.68177335 |
| 3216 | 7 | 23.407 | Marker745468 | 0.459365866 | -0.53615222 | 0.15635424 | 32.68177335 |
| 3217 | 7 | 23.657 | Marker745540 | 0.418759392 | -0.529545455 | 0.150881262 | 31.83123197 |
| 3218 | 7 | 23.657 | Marker745700 | 0.418759392 | -0.529545455 | 0.150881262 | 31.83123197 |
| 3219 | 7 | 24.407 | Marker746040 | 0.297737973 | -0.536525974 | 0.178094457 | 33.06367535 |
| 3220 | 7 | 24.407 | Marker746065 | 0.297737973 | -0.536525974 | 0.178094457 | 33.06367535 |
| 3221 | 7 | 26.458 | Marker747106 | 0.80156018 | -0.556725146 | 0.202339181 | 35.91773302 |
| 3222 | 7 | 26.958 | Marker747450 | 0.673410049 | -0.565217391 | 0.212585812 | 37.16233651 |
| 3223 | 7 | 26.958 | Marker747434 | 0.673410049 | -0.565217391 | 0.212585812 | 37.16233651 |
| 3224 | 7 | 26.958 | Marker747854 | 0.673410049 | -0.565217391 | 0.212585812 | 37.16233651 |
| 3225 | 7 | 26.958 | Marker747449 | 0.673410049 | -0.565217391 | 0.212585812 | 37.16233651 |
| 3226 | 7 | 26.958 | Marker747451 | 0.673410049 | -0.565217391 | 0.212585812 | 37.16233651 |
| 3227 | 7 | 26.958 | Marker747453 | 0.673410049 | -0.565217391 | 0.212585812 | 37.16233651 |
| 3228 | 7 | 26.958 | Marker747903 | 0.673410049 | -0.565217391 | 0.212585812 | 37.16233651 |
| 3229 | 7 | 26.958 | Marker747855 | 0.673410049 | -0.565217391 | 0.212585812 | 37.16233651 |
| 3230 | 7 | 27.208 | Marker748049 | 0.620719541 | -0.569148936 | 0.228723404 | 37.98655027 |
| 3231 | 7 | 27.208 | Marker748206 | 0.620719541 | -0.569148936 | 0.228723404 | 37.98655027 |
| 3232 | 7 | 27.208 | Marker748210 | 0.620719541 | -0.569148936 | 0.228723404 | 37.98655027 |
| 3233 | 7 | 27.208 | Marker748026 | 0.620719541 | -0.569148936 | 0.228723404 | 37.98655027 |
| 3234 | 7 | 27.208 | Marker748050 | 0.620719541 | -0.569148936 | 0.228723404 | 37.98655027 |
| 3235 | 7 | 27.208 | Marker748025 | 0.620719541 | -0.569148936 | 0.228723404 | 37.98655027 |
| 3236 | 7 | 27.208 | Marker748048 | 0.620719541 | -0.569148936 | 0.228723404 | 37.98655027 |
| 3237 | 7 | 27.458 | Marker748511 | 7.600039609 | -0.586956522 | 0.255377574 | 40.85309827 |
| 3238 | 7 | 27.458 | Marker748285 | 7.600039609 | -0.586956522 | 0.255377574 | 40.85309827 |
| 3239 | 7 | 27.708 | Marker748522 | 7.598983069 | -0.582570557 | 0.261555253 | 40.44197599 |
| 3240 | 7 | 27.708 | Marker748574 | 7.598983069 | -0.582570557 | 0.261555253 | 40.44197599 |
| 3241 | 7 | 27.708 | Marker748573 | 7.598983069 | -0.582570557 | 0.261555253 | 40.44197599 |
| 3242 | 7 | 27.958 | Marker748804 | 7.590263113 | -0.586956522 | 0.255377574 | 40.85309827 |
| 3243 | 7 | 27.958 | Marker748801 | 7.590263113 | -0.586956522 | 0.255377574 | 40.85309827 |
| 3244 | 7 | 27.958 | Marker749475 | 7.590263113 | -0.586956522 | 0.255377574 | 40.85309827 |
| 3245 | 7 | 27.958 | Marker748922 | 7.590263113 | -0.586956522 | 0.255377574 | 40.85309827 |
| 3246 | 7 | 27.958 | Marker748921 | 7.590263113 | -0.586956522 | 0.255377574 | 40.85309827 |
| 3247 | 7 | 29.759 | Marker748839 | 0.360204277 | -0.5625 | 0.216346154 | 36.90205221 |
| 3248 | 7 | 29.759 | Marker748828 | 0.360204277 | -0.5625 | 0.216346154 | 36.90205221 |
| 3249 | 7 | 29.759 | Marker748829 | 0.360204277 | -0.5625 | 0.216346154 | 36.90205221 |
| 3250 | 7 | 29.759 | Marker748836 | 0.360204277 | -0.5625 | 0.216346154 | 36.90205221 |
| 3251 | 7 | 29.759 | Marker748840 | 0.360204277 | -0.5625 | 0.216346154 | 36.90205221 |
| 3252 | 7 | 29.759 | Marker748837 | 0.360204277 | -0.5625 | 0.216346154 | 36.90205221 |
| 3253 | 7 | 29.759 | Marker748838 | 0.360204277 | -0.5625 | 0.216346154 | 36.90205221 |
| 3254 | 7 | 31.509 | Marker748870 | 1.053318393 | -0.591501976 | 0.249077734 | 41.290132 |
| 3255 | 7 | 32.009 | Marker749635 | 0.567974159 | -0.583333333 | 0.239583333 | 40.01888425 |
| 3256 | 7 | 32.009 | Marker749611 | 0.567974159 | -0.583333333 | 0.239583333 | 40.01888425 |
| 3257 | 7 | 33.009 | Marker750075 | 0.40703182 | -0.583931419 | 0.210576156 | 39.48126882 |
| 3258 | 7 | 33.009 | Marker750076 | 0.40703182 | -0.583931419 | 0.210576156 | 39.48126882 |
| 3259 | 7 | 33.259 | Marker750074 | 0.42724106 | -0.57996736 | 0.194906771 | 38.6778825 |
| 3260 | 7 | 33.259 | Marker750701 | 0.42724106 | -0.57996736 | 0.194906771 | 38.6778825 |
| 3261 | 7 | 33.509 | Marker750986 | 0.437769265 | -0.584202085 | 0.181544039 | 38.98452612 |
| 3262 | 7 | 33.759 | Marker749968 | 0.233147709 | -0.571344107 | 0.206134473 | 37.79937044 |
| 3263 | 7 | 34.009 | Marker750941 | 0.193303352 | -0.575308166 | 0.222080615 | 38.61856367 |
| 3264 | 7 | 34.259 | Marker750946 | 0.242034457 | -0.579545455 | 0.208577713 | 38.88224419 |
| 3265 | 7 | 34.509 | Marker750945 | 0.240773204 | -0.572222222 | 0.202657005 | 37.8429284 |
| 3266 | 7 | 34.509 | Marker750940 | 0.240773204 | -0.572222222 | 0.202657005 | 37.8429284 |
| 3267 | 7 | 35.51 | Marker750332 | 0.142853244 | -0.559103737 | 0.192967845 | 36.03542053 |
| 3268 | 7 | 35.51 | Marker750331 | 0.142853244 | -0.559103737 | 0.192967845 | 36.03542053 |
| 3269 | 7 | 35.51 | Marker750330 | 0.142853244 | -0.559103737 | 0.192967845 | 36.03542053 |
| 3270 | 7 | 35.51 | Marker750333 | 0.142853244 | -0.559103737 | 0.192967845 | 36.03542053 |
| 3271 | 7 | 36.81 | Marker752807 | 0.220746955 | -0.56705969 | 0.150331614 | 36.33945254 |
| 3272 | 7 | 37.06 | Marker752806 | 0.216563711 | -0.560584205 | 0.145050586 | 35.46618155 |
| 3273 | 7 | 37.06 | Marker752808 | 0.216563711 | -0.560584205 | 0.145050586 | 35.46618155 |
| 3274 | 7 | 37.31 | Marker752805 | 0.215106914 | -0.554378531 | 0.140065913 | 34.6414023 |
| 3275 | 7 | 38.56 | Marker753192 | 0.057719195 | -0.562968516 | 0.164006706 | 36.03021923 |
| 3276 | 7 | 38.56 | Marker753193 | 0.057719195 | -0.562968516 | 0.164006706 | 36.03021923 |
| 3277 | 7 | 38.81 | Marker753141 | 0.053797167 | -0.55649303 | 0.158816868 | 35.15710183 |
| 3278 | 7 | 38.81 | Marker753191 | 0.053797167 | -0.55649303 | 0.158816868 | 35.15710183 |
| 3279 | 7 | 39.81 | Marker752539 | 0.111595975 | -0.562853107 | 0.120480226 | 35.44898792 |
| 3280 | 7 | 40.31 | Marker752458 | 0.19062311 | -0.571327684 | 0.100894539 | 36.31088998 |
| 3281 | 7 | 40.81 | Marker751490 | 0.013599352 | -0.552380952 | 0.121320346 | 34.1755282 |
| 3282 | 7 | 46.333 | Marker758880 | 0.615666553 | -0.590401786 | -0.040951236 | 38.47852619 |
| 3283 | 7 | 46.583 | Marker758850 | 0.601855949 | -0.595238095 | -0.045548654 | 39.13443374 |
| 3284 | 7 | 46.583 | Marker759184 | 0.601855949 | -0.595238095 | -0.045548654 | 39.13443374 |
| 3285 | 7 | 46.583 | Marker758849 | 0.601855949 | -0.595238095 | -0.045548654 | 39.13443374 |
| 3286 | 7 | 47.833 | Marker758188 | 0.380287515 | -0.580261137 | -0.054147465 | 37.24766017 |
| 3287 | 7 | 47.833 | Marker758187 | 0.380287515 | -0.580261137 | -0.054147465 | 37.24766017 |
| 3288 | 7 | 47.833 | Marker758190 | 0.380287515 | -0.580261137 | -0.054147465 | 37.24766017 |
| 3289 | 7 | 47.833 | Marker758181 | 0.380287515 | -0.580261137 | -0.054147465 | 37.24766017 |
| 3290 | 7 | 47.833 | Marker758189 | 0.380287515 | -0.580261137 | -0.054147465 | 37.24766017 |
| 3291 | 7 | 48.333 | Marker758271 | 0.076901667 | -0.572196621 | -0.035330261 | 36.12140762 |
| 3292 | 7 | 48.333 | Marker758269 | 0.076901667 | -0.572196621 | -0.035330261 | 36.12140762 |
| 3293 | 7 | 48.333 | Marker758265 | 0.076901667 | -0.572196621 | -0.035330261 | 36.12140762 |
| 3294 | 7 | 48.333 | Marker758270 | 0.076901667 | -0.572196621 | -0.035330261 | 36.12140762 |
| 3295 | 7 | 50.635 | Marker757802 | 0.01968497 | -0.571428571 | -0.091097308 | 36.43138003 |
| 3296 | 7 | 50.885 | Marker757804 | 0.008317921 | -0.56562137 | -0.107189689 | 35.87822889 |
| 3297 | 7 | 50.885 | Marker757799 | 0.008317921 | -0.56562137 | -0.107189689 | 35.87822889 |
| 3298 | 7 | 50.885 | Marker757803 | 0.008317921 | -0.56562137 | -0.107189689 | 35.87822889 |
| 3299 | 7 | 50.885 | Marker757801 | 0.008317921 | -0.56562137 | -0.107189689 | 35.87822889 |
| 3300 | 7 | 50.885 | Marker757806 | 0.008317921 | -0.56562137 | -0.107189689 | 35.87822889 |
| 3301 | 7 | 50.885 | Marker757805 | 0.008317921 | -0.56562137 | -0.107189689 | 35.87822889 |
| 3302 | 7 | 52.635 | Marker756677 | 0.047804263 | -0.59587889 | -0.089905769 | 39.56369111 |
| 3303 | 7 | 53.886 | Marker756106 | 0.059337094 | -0.598111658 | -0.115539248 | 40.14562124 |
| 3304 | 7 | 53.886 | Marker756107 | 0.059337094 | -0.598111658 | -0.115539248 | 40.14562124 |
| 3305 | 7 | 53.886 | Marker756108 | 0.059337094 | -0.598111658 | -0.115539248 | 40.14562124 |
| 3306 | 7 | 55.136 | Marker753970 | 0.012289409 | -0.588983051 | -0.151483051 | 39.4643623 |
| 3307 | 7 | 55.636 | Marker754368 | 5.93702622 | -0.589842464 | -0.162768238 | 39.76346499 |
| 3308 | 7 | 55.636 | Marker754365 | 5.93702622 | -0.589842464 | -0.162768238 | 39.76346499 |
| 3309 | 7 | 56.386 | Marker754989 | 0.008597953 | -0.595765289 | -0.139156305 | 40.15986105 |
| 3310 | 7 | 56.636 | Marker754339 | 0.004315204 | -0.592105263 | -0.143125671 | 39.74024724 |
| 3311 | 7 | 56.636 | Marker754668 | 0.004315204 | -0.592105263 | -0.143125671 | 39.74024724 |
| 3312 | 7 | 56.886 | Marker754334 | 0.139604379 | -0.586007702 | -0.158808885 | 39.19906078 |
| 3313 | 7 | 57.136 | Marker753832 | 0.167993374 | -0.592105263 | -0.143125671 | 39.74024724 |
| 3314 | 7 | 57.136 | Marker753825 | 0.167993374 | -0.592105263 | -0.143125671 | 39.74024724 |
| 3315 | 7 | 57.386 | Marker753801 | 0.880208801 | -0.597919217 | -0.127528423 | 40.27735771 |
| 3316 | 7 | 57.386 | Marker753831 | 0.880208801 | -0.597919217 | -0.127528423 | 40.27735771 |
| 3317 | 7 | 58.136 | Marker761267 | 0.442553989 | -0.586363636 | -0.116363636 | 38.62246141 |
| 3318 | 7 | 58.636 | Marker760473 | 0.305917175 | -0.586363636 | -0.116363636 | 38.62246141 |
| 3319 | 7 | 58.886 | Marker759956 | 0.090967907 | -0.580266075 | -0.132065158 | 38.04789667 |
| 3320 | 7 | 58.886 | Marker760106 | 0.090967907 | -0.580266075 | -0.132065158 | 38.04789667 |
| 3321 | 7 | 58.886 | Marker760105 | 0.090967907 | -0.580266075 | -0.132065158 | 38.04789667 |
| 3322 | 7 | 58.886 | Marker759955 | 0.090967907 | -0.580266075 | -0.132065158 | 38.04789667 |
| 3323 | 7 | 58.886 | Marker759948 | 0.090967907 | -0.580266075 | -0.132065158 | 38.04789667 |
| 3324 | 7 | 59.386 | Marker760457 | 0.293940367 | -0.580549683 | -0.12248062 | 37.95346436 |
| 3325 | 7 | 60.936 | Marker762128 | 0.241281468 | -0.544372294 | -0.121991342 | 33.46135336 |
| 3326 | 7 | 61.436 | Marker761986 | 0.351801062 | -0.546522132 | -0.102715341 | 33.48658122 |
| 3327 | 7 | 61.687 | Marker761987 | 0.339762537 | -0.552314815 | -0.096732291 | 34.12298882 |
| 3328 | 7 | 61.687 | Marker762134 | 0.339762537 | -0.552314815 | -0.096732291 | 34.12298882 |
| 3329 | 7 | 61.687 | Marker761988 | 0.339762537 | -0.552314815 | -0.096732291 | 34.12298882 |
| 3330 | 7 | 62.187 | Marker763838 | 0.351482089 | -0.52842987 | -0.130942704 | 31.69657198 |
| 3331 | 7 | 62.937 | Marker764682 | 0.472248907 | -0.522431866 | -0.110310654 | 30.73599358 |
| 3332 | 7 | 63.437 | Marker764681 | 0.666229328 | -0.512205387 | -0.080387205 | 29.25981792 |
| 3333 | 7 | 63.937 | Marker763049 | 0.687149823 | -0.513319089 | -0.084701975 | 29.42489007 |
| 3334 | 7 | 64.187 | Marker763305 | 0.681846445 | -0.505598622 | -0.096993971 | 28.67990996 |
| 3335 | 7 | 65.187 | Marker763889 | 0.808044093 | -0.497763864 | -0.069355291 | 27.56127618 |
| 3336 | 7 | 65.187 | Marker763829 | 0.808044093 | -0.497763864 | -0.069355291 | 27.56127618 |
| 3337 | 7 | 66.187 | Marker765613 | 1.087730098 | -0.478846154 | -0.058846154 | 25.45038787 |
| 3338 | 7 | 66.187 | Marker765616 | 1.087730098 | -0.478846154 | -0.058846154 | 25.45038787 |
| 3339 | 7 | 66.187 | Marker765617 | 1.087730098 | -0.478846154 | -0.058846154 | 25.45038787 |
| 3340 | 7 | 66.437 | Marker765502 | 1.343221047 | -0.474509804 | -0.044806834 | 24.91098413 |
| 3341 | 7 | 66.437 | Marker765503 | 1.343221047 | -0.474509804 | -0.044806834 | 24.91098413 |
| 3342 | 7 | 66.437 | Marker765501 | 1.343221047 | -0.474509804 | -0.044806834 | 24.91098413 |
| 3343 | 7 | 66.437 | Marker765260 | 1.343221047 | -0.474509804 | -0.044806834 | 24.91098413 |
| 3344 | 7 | 66.437 | Marker765338 | 1.343221047 | -0.474509804 | -0.044806834 | 24.91098413 |
| 3345 | 7 | 66.437 | Marker765658 | 1.343221047 | -0.474509804 | -0.044806834 | 24.91098413 |
| 3346 | 7 | 66.437 | Marker765259 | 1.343221047 | -0.474509804 | -0.044806834 | 24.91098413 |
| 3347 | 7 | 66.437 | Marker764800 | 1.343221047 | -0.474509804 | -0.044806834 | 24.91098413 |
| 3348 | 7 | 66.937 | Marker767793 | 1.606773721 | -0.474545455 | -0.026619594 | 24.83473315 |
| 3349 | 7 | 67.187 | Marker767601 | 1.807778266 | -0.469851577 | -0.012683745 | 24.30773851 |
| 3350 | 7 | 67.187 | Marker767605 | 1.807778266 | -0.469851577 | -0.012683745 | 24.30773851 |
| 3351 | 7 | 67.437 | Marker767604 | 1.817269606 | -0.462980541 | -0.029536466 | 23.65181241 |
| 3352 | 7 | 67.687 | Marker767646 | 1.822172815 | -0.467687075 | -0.025189321 | 24.11852004 |
| 3353 | 7 | 67.687 | Marker767647 | 1.822172815 | -0.467687075 | -0.025189321 | 24.11852004 |
| 3354 | 7 | 67.937 | Marker768337 | 2.170074732 | -0.462797619 | -0.011306742 | 23.58094511 |
| 3355 | 7 | 67.937 | Marker768487 | 2.170074732 | -0.462797619 | -0.011306742 | 23.58094511 |
| 3356 | 7 | 68.437 | Marker767657 | 1.963315057 | -0.462130823 | -0.027109715 | 23.55643452 |
| 3357 | 7 | 68.937 | Marker768943 | 2.161594949 | -0.453598485 | -0.019615801 | 22.67284111 |
| 3358 | 7 | 69.687 | Marker770890 | 3.508631649 | -0.427224371 | 0.034610963 | 20.11372443 |
| 3359 | 7 | 69.687 | Marker770972 | 3.508631649 | -0.427224371 | 0.034610963 | 20.11372443 |
| 3360 | 7 | 69.687 | Marker770891 | 3.508631649 | -0.427224371 | 0.034610963 | 20.11372443 |
| 3361 | 7 | 69.687 | Marker770889 | 3.508631649 | -0.427224371 | 0.034610963 | 20.11372443 |
| 3362 | 7 | 69.937 | Marker769741 | 3.505681001 | -0.424146571 | 0.032048488 | 19.81916819 |
| 3363 | 7 | 70.687 | Marker770827 | 2.651226306 | -0.417361111 | -0.004113248 | 19.16903603 |
| 3364 | 7 | 70.937 | Marker771361 | 3.340289205 | -0.420625981 | -0.018679669 | 19.4976923 |
| 3365 | 7 | 71.437 | Marker771827 | 3.380359471 | -0.424418605 | -0.037845546 | 19.91885215 |
| 3366 | 7 | 71.437 | Marker771828 | 3.380359471 | -0.424418605 | -0.037845546 | 19.91885215 |
| 3367 | 7 | 71.937 | Marker771825 | 2.637019908 | -0.425773694 | -0.047128754 | 20.09191017 |
| 3368 | 7 | 71.937 | Marker771826 | 2.637019908 | -0.425773694 | -0.047128754 | 20.09191017 |
| 3369 | 7 | 72.687 | Marker771763 | 2.65726308 | -0.441365661 | -0.022543665 | 21.47633286 |
| 3370 | 7 | 72.687 | Marker771764 | 2.65726308 | -0.441365661 | -0.022543665 | 21.47633286 |
| 3371 | 7 | 72.687 | Marker771765 | 2.65726308 | -0.441365661 | -0.022543665 | 21.47633286 |
| 3372 | 7 | 73.687 | Marker772493 | 1.405955402 | -0.450892857 | -0.032557299 | 22.44753015 |
| 3373 | 7 | 73.687 | Marker772487 | 1.405955402 | -0.450892857 | -0.032557299 | 22.44753015 |
| 3374 | 7 | 74.187 | Marker772357 | 1.36160787 | -0.458316366 | -0.031205772 | 23.18518844 |
| 3375 | 7 | 74.937 | Marker772573 | 1.537572199 | -0.449230546 | -0.034087264 | 22.28909984 |
| 3376 | 7 | 75.437 | Marker772850 | 1.41332571 | -0.455538618 | -0.028257678 | 22.89462034 |
| 3377 | 7 | 75.437 | Marker772049 | 1.41332571 | -0.455538618 | -0.028257678 | 22.89462034 |
| 3378 | 7 | 75.437 | Marker772486 | 1.41332571 | -0.455538618 | -0.028257678 | 22.89462034 |
| 3379 | 7 | 75.437 | Marker772849 | 1.41332571 | -0.455538618 | -0.028257678 | 22.89462034 |
| 3380 | 7 | 76.988 | Marker776392 | 0.761931311 | -0.446463178 | -0.036634306 | 22.02719072 |
| 3381 | 7 | 77.238 | Marker776220 | 0.754420132 | -0.438378876 | -0.054378271 | 21.33363683 |
| 3382 | 7 | 77.738 | Marker775006 | 0.80354513 | -0.431148374 | -0.071166441 | 20.75856564 |
| 3383 | 7 | 77.738 | Marker776219 | 0.80354513 | -0.431148374 | -0.071166441 | 20.75856564 |
| 3384 | 7 | 77.738 | Marker775005 | 0.80354513 | -0.431148374 | -0.071166441 | 20.75856564 |
| 3385 | 7 | 78.238 | Marker776303 | 0.731238779 | -0.419323068 | -0.092258933 | 19.83700366 |
| 3386 | 7 | 79.238 | Marker776749 | 1.541595768 | -0.418055556 | -0.066269841 | 19.49656933 |
| 3387 | 7 | 79.238 | Marker776750 | 1.541595768 | -0.418055556 | -0.066269841 | 19.49656933 |
| 3388 | 7 | 79.238 | Marker776751 | 1.541595768 | -0.418055556 | -0.066269841 | 19.49656933 |
| 3389 | 7 | 80.193 | Marker777195 | 1.893083588 | -0.363636364 | -0.086113428 | 14.97227271 |
| 3390 | 7 | 80.943 | Marker777409 | 3.356178171 | -0.348412698 | -0.058513709 | 13.56240148 |
| 3391 | 7 | 81.443 | Marker777581 | 3.297746121 | -0.342049884 | -0.074565689 | 13.19394621 |
| 3392 | 7 | 81.943 | Marker777480 | 3.162493473 | -0.357955091 | -0.050539064 | 14.25683976 |
| 3393 | 7 | 82.443 | Marker777580 | 3.287841164 | -0.377057136 | -0.056703918 | 15.84028393 |
| 3394 | 7 | 84.149 | Marker779303 | 5.080715817 | -0.341036782 | -0.078526238 | 13.15009652 |
| 3395 | 7 | 85.104 | Marker779114 | 5.22856746 | -0.347308489 | -0.090864627 | 13.73708522 |
| 3396 | 7 | 85.604 | Marker778747 | 4.871603192 | -0.333160353 | -0.104473846 | 12.81513713 |
| 3397 | 7 | 85.604 | Marker780647 | 4.871603192 | -0.333160353 | -0.104473846 | 12.81513713 |
| 3398 | 7 | 85.604 | Marker780553 | 4.871603192 | -0.333160353 | -0.104473846 | 12.81513713 |
| 3399 | 7 | 85.604 | Marker778744 | 4.871603192 | -0.333160353 | -0.104473846 | 12.81513713 |
| 3400 | 7 | 86.259 | Marker780171 | 4.466521697 | -0.288888889 | -0.123987539 | 10.00730931 |
| 3401 | 7 | 86.259 | Marker780325 | 4.466521697 | -0.288888889 | -0.123987539 | 10.00730931 |
| 3402 | 7 | 86.259 | Marker780326 | 4.466521697 | -0.288888889 | -0.123987539 | 10.00730931 |
| 3403 | 7 | 87.465 | Marker781155 | 2.392000153 | -0.339130435 | -0.148139444 | 13.82996341 |
| 3404 | 7 | 87.965 | Marker781433 | 0.604735561 | -0.353116531 | -0.176012598 | 15.36340235 |
| 3405 | 7 | 87.965 | Marker781511 | 0.604735561 | -0.353116531 | -0.176012598 | 15.36340235 |
| 3406 | 7 | 89.265 | Marker782145 | 0.090903479 | -0.344849951 | -0.170938333 | 14.63574016 |
| 3407 | 7 | 89.765 | Marker782955 | 1.208773554 | -0.338888889 | -0.199494949 | 14.72579973 |
| 3408 | 7 | 89.765 | Marker782642 | 1.208773554 | -0.338888889 | -0.199494949 | 14.72579973 |
| 3409 | 7 | 90.265 | Marker783248 | 0.067272363 | -0.335365854 | -0.182491289 | 14.1318145 |
| 3410 | 7 | 91.265 | Marker784052 | 0.361100117 | -0.333934917 | -0.1699166 | 13.79933361 |
| 3411 | 7 | 92.015 | Marker784427 | 0.57059403 | -0.308928571 | -0.166071429 | 11.95684904 |
| 3412 | 7 | 92.015 | Marker785589 | 0.57059403 | -0.308928571 | -0.166071429 | 11.95684904 |
| 3413 | 7 | 92.515 | Marker786094 | 0.554725435 | -0.287231976 | -0.135547818 | 10.05622101 |
| 3414 | 7 | 93.815 | Marker787349 | 0.168736773 | -0.282539683 | -0.13968254 | 9.81926864 |
| 3415 | 7 | 93.815 | Marker787348 | 0.168736773 | -0.282539683 | -0.13968254 | 9.81926864 |
| 3416 | 7 | 95.416 | Marker788593 | 0.29814771 | -0.250642393 | -0.133045285 | 7.84745536 |
| 3417 | 7 | 96.766 | Marker791008 | 0.325311543 | -0.267684109 | -0.128149225 | 8.757880558 |
| 3418 | 7 | 97.766 | Marker791009 | 0.343940874 | -0.238139535 | -0.121860465 | 7.026298652 |
| 3419 | 7 | 98.266 | Marker791005 | 0.079183637 | -0.255102041 | -0.102810936 | 7.730254849 |
| 3420 | 7 | 98.516 | Marker791004 | 0.195620272 | -0.258928571 | -0.106725634 | 7.989768923 |
| 3421 | 7 | 100.067 | Marker793448 | 0.203158404 | -0.281146978 | -0.118523403 | 9.451717921 |
| 3422 | 7 | 100.567 | Marker793562 | 0.157233958 | -0.279304029 | -0.108216496 | 9.216943562 |
| 3423 | 7 | 100.567 | Marker793560 | 0.157233958 | -0.279304029 | -0.108216496 | 9.216943562 |
| 3424 | 7 | 100.817 | Marker802395 | 0.097070939 | -0.272045028 | -0.092557849 | 8.612038612 |
| 3425 | 7 | 100.817 | Marker794407 | 0.097070939 | -0.272045028 | -0.092557849 | 8.612038612 |
| 3426 | 7 | 100.817 | Marker793460 | 0.097070939 | -0.272045028 | -0.092557849 | 8.612038612 |
| 3427 | 7 | 100.817 | Marker798378 | 0.097070939 | -0.272045028 | -0.092557849 | 8.612038612 |
| 3428 | 7 | 100.817 | Marker793463 | 0.097070939 | -0.272045028 | -0.092557849 | 8.612038612 |
| 3429 | 7 | 100.817 | Marker800323 | 0.097070939 | -0.272045028 | -0.092557849 | 8.612038612 |
| 3430 | 7 | 100.817 | Marker797034 | 0.097070939 | -0.272045028 | -0.092557849 | 8.612038612 |
| 3431 | 7 | 101.067 | Marker793458 | 0.123601137 | -0.266134716 | -0.101910182 | 8.355487001 |
| 3432 | 7 | 101.567 | Marker802025 | 0.184751889 | -0.270121951 | -0.094259882 | 8.513550523 |
| 3433 | 7 | 101.567 | Marker797535 | 0.184751889 | -0.270121951 | -0.094259882 | 8.513550523 |
| 3434 | 7 | 101.567 | Marker797533 | 0.184751889 | -0.270121951 | -0.094259882 | 8.513550523 |
| 3435 | 7 | 101.567 | Marker802595 | 0.184751889 | -0.270121951 | -0.094259882 | 8.513550523 |
| 3436 | 7 | 101.567 | Marker802597 | 0.184751889 | -0.270121951 | -0.094259882 | 8.513550523 |
| 3437 | 7 | 101.567 | Marker797534 | 0.184751889 | -0.270121951 | -0.094259882 | 8.513550523 |
| 3438 | 7 | 102.067 | Marker797567 | 0.224090255 | -0.267399267 | -0.087691045 | 8.289794525 |
| 3439 | 7 | 102.067 | Marker797568 | 0.224090255 | -0.267399267 | -0.087691045 | 8.289794525 |
| 3440 | 7 | 102.567 | Marker793462 | 0.096683356 | -0.270121951 | -0.094259882 | 8.513550523 |
| 3441 | 7 | 103.317 | Marker793956 | 0.113131082 | -0.238095238 | -0.092498946 | 6.700206935 |
| 3442 | 7 | 103.817 | Marker793949 | 0.088345985 | -0.220930233 | -0.068615127 | 5.630627909 |
| 3443 | 7 | 103.817 | Marker793950 | 0.088345985 | -0.220930233 | -0.068615127 | 5.630627909 |
| 3444 | 7 | 103.817 | Marker793954 | 0.088345985 | -0.220930233 | -0.068615127 | 5.630627909 |
| 3445 | 7 | 104.067 | Marker793955 | 0.110643464 | -0.236710963 | -0.093646179 | 6.638706264 |
| 3446 | 7 | 104.817 | Marker798939 | 0.213071323 | -0.229978355 | -0.083903709 | 6.202289839 |
| 3447 | 7 | 106.221 | Marker796813 | 0.280350825 | -0.200680272 | -0.086574252 | 4.833045199 |
| 3448 | 7 | 106.221 | Marker796814 | 0.280350825 | -0.200680272 | -0.086574252 | 4.833045199 |
| 3449 | 7 | 106.471 | Marker799959 | 0.174054442 | -0.197687661 | -0.080796684 | 4.651538076 |
| 3450 | 7 | 106.471 | Marker799960 | 0.174054442 | -0.197687661 | -0.080796684 | 4.651538076 |
| 3451 | 7 | 106.721 | Marker796812 | 0.235635755 | -0.186511628 | -0.064642218 | 4.055866949 |
| 3452 | 7 | 106.971 | Marker796807 | 0.229611213 | -0.185454545 | -0.065419241 | 4.017737587 |
| 3453 | 7 | 108.631 | Marker799955 | 0.069456363 | -0.218690683 | -0.051009633 | 5.410921814 |
| 3454 | 7 | 110.181 | Marker802214 | 0.205940192 | -0.246352967 | -0.008714954 | 6.684995119 |
| 3455 | 7 | 110.431 | Marker802213 | 0.238636302 | -0.248937111 | -0.006105398 | 6.822756167 |
| 3456 | 7 | 111.166 | Marker798206 | 0.188166465 | -0.233534072 | -0.010102148 | 6.009849081 |
| 3457 | 7 | 111.416 | Marker798207 | 0.221706691 | -0.23787453 | -0.00827429 | 6.232596795 |
| 3458 | 7 | 112.401 | Marker796912 | 0.071905188 | -0.244616155 | -0.045747509 | 6.707374312 |
| 3459 | 7 | 113.151 | Marker796911 | 0.055184329 | -0.236584763 | -0.068843348 | 6.422251994 |
| 3460 | 7 | 114.201 | Marker794750 | 0.037018261 | -0.238636364 | -0.06557548 | 6.506649547 |
| 3461 | 7 | 115.304 | Marker794179 | 0.323470297 | -0.212249141 | -0.10088183 | 5.498545667 |
| 3462 | 7 | 116.604 | Marker795628 | 0.057997311 | -0.236358656 | -0.056443258 | 6.328336646 |
| 3463 | 7 | 117.711 | Marker797279 | 0.087626146 | -0.261182866 | -0.049695058 | 7.651198339 |
| 3464 | 7 | 118.211 | Marker797277 | 0.067672093 | -0.269448547 | -0.033899284 | 8.061488943 |
| 3465 | 7 | 119.315 | Marker796930 | 0.014859873 | -0.243083004 | 0.004155739 | 6.5002325 |
| 3466 | 7 | 119.315 | Marker796933 | 0.014859873 | -0.243083004 | 0.004155739 | 6.5002325 |
| 3467 | 8 | 0 | Marker803196 | 0.464636745 | 0.086841978 | 0.0375498 | 0.905378287 |
| 3468 | 8 | 0.359 | Marker803195 | 0.561939501 | 0.095251077 | 0.058238978 | 1.175950544 |
| 3469 | 8 | 1.109 | Marker803193 | 0.56260132 | 0.098690072 | 0.049925895 | 1.203770839 |
| 3470 | 8 | 2.96 | Marker804199 | 0.448828754 | 0.072920696 | 0.029471648 | 0.631890153 |
| 3471 | 8 | 4.01 | Marker804476 | 0.383296497 | 0.08025252 | 0.014040787 | 0.720406314 |
| 3472 | 8 | 4.01 | Marker804475 | 0.383296497 | 0.08025252 | 0.014040787 | 0.720406314 |
| 3473 | 8 | 4.01 | Marker804478 | 0.383296497 | 0.08025252 | 0.014040787 | 0.720406314 |
| 3474 | 8 | 4.81 | Marker804477 | 0.318105516 | 0.082007576 | 0.010642328 | 0.74712891 |
| 3475 | 8 | 6.661 | Marker803462 | 0.316043968 | 0.075217391 | 0.023990529 | 0.654214653 |
| 3476 | 8 | 6.911 | Marker803461 | 0.397771239 | 0.085625555 | 0.043426066 | 0.906643142 |
| 3477 | 8 | 6.911 | Marker803207 | 0.397771239 | 0.085625555 | 0.043426066 | 0.906643142 |
| 3478 | 8 | 7.161 | Marker804153 | 0.305536929 | 0.086050725 | 0.042994583 | 0.912797423 |
| 3479 | 8 | 7.161 | Marker804601 | 0.305536929 | 0.086050725 | 0.042994583 | 0.912797423 |
| 3480 | 8 | 7.411 | Marker804603 | 0.316743893 | 0.088126687 | 0.047333676 | 0.972808499 |
| 3481 | 8 | 7.911 | Marker805129 | 0.298236309 | 0.085625555 | 0.043426066 | 0.906643142 |
| 3482 | 8 | 7.911 | Marker805175 | 0.298236309 | 0.085625555 | 0.043426066 | 0.906643142 |
| 3483 | 8 | 7.911 | Marker805174 | 0.298236309 | 0.085625555 | 0.043426066 | 0.906643142 |
| 3484 | 8 | 8.161 | Marker805176 | 0.298764283 | 0.08707483 | 0.041120137 | 0.92429635 |
| 3485 | 8 | 8.161 | Marker805180 | 0.298764283 | 0.08707483 | 0.041120137 | 0.92429635 |
| 3486 | 8 | 8.411 | Marker805190 | 0.287317368 | 0.074755989 | 0.064099553 | 0.826635511 |
| 3487 | 8 | 8.411 | Marker805200 | 0.287317368 | 0.074755989 | 0.064099553 | 0.826635511 |
| 3488 | 8 | 8.411 | Marker805202 | 0.287317368 | 0.074755989 | 0.064099553 | 0.826635511 |
| 3489 | 8 | 8.411 | Marker805201 | 0.287317368 | 0.074755989 | 0.064099553 | 0.826635511 |
| 3490 | 8 | 8.411 | Marker805183 | 0.287317368 | 0.074755989 | 0.064099553 | 0.826635511 |
| 3491 | 8 | 8.411 | Marker805182 | 0.287317368 | 0.074755989 | 0.064099553 | 0.826635511 |
| 3492 | 8 | 8.411 | Marker805184 | 0.287317368 | 0.074755989 | 0.064099553 | 0.826635511 |
| 3493 | 8 | 8.411 | Marker805204 | 0.287317368 | 0.074755989 | 0.064099553 | 0.826635511 |
| 3494 | 8 | 8.911 | Marker805607 | 0.284635768 | 0.077226345 | 0.059743828 | 0.840966277 |
| 3495 | 8 | 8.911 | Marker805608 | 0.284635768 | 0.077226345 | 0.059743828 | 0.840966277 |
| 3496 | 8 | 8.911 | Marker805606 | 0.284635768 | 0.077226345 | 0.059743828 | 0.840966277 |
| 3497 | 8 | 9.911 | Marker805886 | 0.30711928 | 0.103741497 | 0.012546528 | 1.194267062 |
| 3498 | 8 | 11.462 | Marker806234 | 0.429628068 | 0.091036415 | 0.034717733 | 0.977171234 |
| 3499 | 8 | 12.063 | Marker806850 | 0.411033063 | 0.099153298 | 0.018493761 | 1.101938221 |
| 3500 | 8 | 12.063 | Marker806895 | 0.411033063 | 0.099153298 | 0.018493761 | 1.101938221 |
| 3501 | 8 | 12.063 | Marker806896 | 0.411033063 | 0.099153298 | 0.018493761 | 1.101938221 |
| 3502 | 8 | 12.313 | Marker806893 | 0.455933524 | 0.109545455 | 0.037833186 | 1.398610722 |
| 3503 | 8 | 12.313 | Marker806892 | 0.455933524 | 0.109545455 | 0.037833186 | 1.398610722 |
| 3504 | 8 | 12.313 | Marker806894 | 0.455933524 | 0.109545455 | 0.037833186 | 1.398610722 |
| 3505 | 8 | 13.063 | Marker807727 | 0.44118882 | 0.096856913 | 0.021639057 | 1.05913141 |
| 3506 | 8 | 13.063 | Marker807725 | 0.44118882 | 0.096856913 | 0.021639057 | 1.05913141 |
| 3507 | 8 | 13.063 | Marker807726 | 0.44118882 | 0.096856913 | 0.021639057 | 1.05913141 |
| 3508 | 8 | 14.063 | Marker807858 | 0.431218199 | 0.08721174 | 0.002363255 | 0.837480888 |
| 3509 | 8 | 14.063 | Marker807991 | 0.431218199 | 0.08721174 | 0.002363255 | 0.837480888 |
| 3510 | 8 | 14.063 | Marker807857 | 0.431218199 | 0.08721174 | 0.002363255 | 0.837480888 |
| 3511 | 8 | 14.313 | Marker808003 | 0.362885668 | 0.077777778 | -0.017777778 | 0.678660416 |
| 3512 | 8 | 14.313 | Marker808002 | 0.362885668 | 0.077777778 | -0.017777778 | 0.678660416 |
| 3513 | 8 | 14.563 | Marker808625 | 0.332793819 | 0.068343816 | -0.03670669 | 0.575887491 |
| 3514 | 8 | 14.563 | Marker808446 | 0.332793819 | 0.068343816 | -0.03670669 | 0.575887491 |
| 3515 | 8 | 14.563 | Marker808626 | 0.332793819 | 0.068343816 | -0.03670669 | 0.575887491 |
| 3516 | 8 | 14.563 | Marker808445 | 0.332793819 | 0.068343816 | -0.03670669 | 0.575887491 |
| 3517 | 8 | 14.563 | Marker808447 | 0.332793819 | 0.068343816 | -0.03670669 | 0.575887491 |
| 3518 | 8 | 14.813 | Marker808627 | 0.279632528 | 0.059259259 | -0.05547997 | 0.532427859 |
| 3519 | 8 | 14.813 | Marker808968 | 0.279632528 | 0.059259259 | -0.05547997 | 0.532427859 |
| 3520 | 8 | 15.416 | Marker809590 | 0.736789217 | 0.093181818 | -0.080751391 | 1.263727261 |
| 3521 | 8 | 16.166 | Marker811301 | 0.802577129 | 0.107142857 | -0.066738817 | 1.470007498 |
| 3522 | 8 | 17.216 | Marker814974 | 0.999316037 | 0.125051 | -0.066270027 | 1.922343149 |
| 3523 | 8 | 17.716 | Marker814973 | 0.803704552 | 0.115982815 | -0.077012547 | 1.756728927 |
| 3524 | 8 | 18.516 | Marker813226 | 0.757625352 | 0.104842489 | -0.077324318 | 1.489950973 |
| 3525 | 8 | 19.616 | Marker816826 | 0.668396335 | 0.09137931 | -0.076705796 | 1.196595087 |
| 3526 | 8 | 19.866 | Marker816828 | 0.663321272 | 0.089205397 | -0.074188712 | 1.135352762 |
| 3527 | 8 | 20.116 | Marker816827 | 0.667597104 | 0.087123991 | -0.071756675 | 1.078105933 |
| 3528 | 8 | 20.616 | Marker816829 | 0.728468196 | 0.097826087 | -0.055272895 | 1.193971045 |
| 3529 | 8 | 21.666 | Marker815774 | 0.819051955 | 0.090621618 | -0.078285832 | 1.193366656 |
| 3530 | 8 | 23.517 | Marker814221 | 0.631380696 | 0.103432701 | -0.130665816 | 1.995903041 |
| 3531 | 8 | 24.267 | Marker812556 | 0.85714494 | 0.106011015 | -0.134404153 | 2.102934569 |
| 3532 | 8 | 24.517 | Marker814258 | 0.856269054 | 0.106132075 | -0.13425254 | 2.103747403 |
| 3533 | 8 | 24.517 | Marker812554 | 0.856269054 | 0.106132075 | -0.13425254 | 2.103747403 |
| 3534 | 8 | 24.517 | Marker812553 | 0.856269054 | 0.106132075 | -0.13425254 | 2.103747403 |
| 3535 | 8 | 24.517 | Marker814100 | 0.856269054 | 0.106132075 | -0.13425254 | 2.103747403 |
| 3536 | 8 | 25.267 | Marker817281 | 1.036255543 | 0.099998147 | -0.128340795 | 1.890523661 |
| 3537 | 8 | 25.517 | Marker815775 | 1.014056583 | 0.100179695 | -0.128111622 | 1.891617716 |
| 3538 | 8 | 27.318 | Marker817964 | 1.205264529 | 0.09154334 | -0.184472633 | 2.572468875 |
| 3539 | 8 | 28.318 | Marker818127 | 1.318700482 | 0.091405772 | -0.220406091 | 3.282553646 |
| 3540 | 8 | 29.569 | Marker818501 | 1.204962308 | 0.075925926 | -0.199130763 | 2.565759373 |
| 3541 | 8 | 30.319 | Marker818794 | 1.325058348 | 0.080489763 | -0.216066499 | 2.987561813 |
| 3542 | 8 | 30.319 | Marker818792 | 1.325058348 | 0.080489763 | -0.216066499 | 2.987561813 |
| 3543 | 8 | 31.319 | Marker818847 | 1.22131336 | 0.073001112 | -0.216162302 | 2.86609023 |
| 3544 | 8 | 33.37 | Marker820594 | 1.153999305 | 0.060351882 | -0.256191757 | 3.615288599 |
| 3545 | 8 | 34.62 | Marker819724 | 0.870919957 | 0.050983541 | -0.234605404 | 2.983485495 |
| 3546 | 8 | 34.62 | Marker819723 | 0.870919957 | 0.050983541 | -0.234605404 | 2.983485495 |
| 3547 | 8 | 35.37 | Marker819725 | 1.065073273 | 0.041133845 | -0.25799659 | 3.4554285 |
| 3548 | 8 | 39.278 | Marker822594 | 1.040945608 | 0.036643026 | -0.223674434 | 2.604612889 |
| 3549 | 8 | 40.028 | Marker823387 | 0.813879338 | 0.03115942 | -0.183360678 | 1.757511973 |
| 3550 | 8 | 40.028 | Marker823389 | 0.813879338 | 0.03115942 | -0.183360678 | 1.757511973 |
| 3551 | 8 | 40.028 | Marker823388 | 0.813879338 | 0.03115942 | -0.183360678 | 1.757511973 |
| 3552 | 8 | 40.778 | Marker824308 | 0.962223849 | 0.048418972 | -0.206891138 | 2.354453587 |
| 3553 | 8 | 40.778 | Marker824307 | 0.962223849 | 0.048418972 | -0.206891138 | 2.354453587 |
| 3554 | 8 | 40.778 | Marker824306 | 0.962223849 | 0.048418972 | -0.206891138 | 2.354453587 |
| 3555 | 8 | 40.778 | Marker824309 | 0.962223849 | 0.048418972 | -0.206891138 | 2.354453587 |
| 3556 | 8 | 41.528 | Marker824169 | 1.148720139 | 0.027083333 | -0.201762821 | 2.082005097 |
| 3557 | 8 | 41.528 | Marker824170 | 1.148720139 | 0.027083333 | -0.201762821 | 2.082005097 |
| 3558 | 8 | 41.778 | Marker824174 | 1.260535218 | 0.018367347 | -0.220467605 | 2.431217654 |
| 3559 | 8 | 41.778 | Marker824175 | 1.260535218 | 0.018367347 | -0.220467605 | 2.431217654 |
| 3560 | 8 | 42.528 | Marker824173 | 0.756043354 | 0.030106885 | -0.174754467 | 1.59896907 |
| 3561 | 8 | 43.579 | Marker825407 | 0.602095382 | 0.026356589 | -0.168864234 | 1.477153473 |
| 3562 | 8 | 44.329 | Marker825777 | 0.667618136 | 0.041029437 | -0.201559972 | 2.17734144 |
| 3563 | 8 | 45.329 | Marker825822 | 0.600005353 | 0.039670655 | -0.191239733 | 1.966238885 |
| 3564 | 8 | 45.829 | Marker825046 | 0.721145051 | 0.04047619 | -0.191991342 | 1.987235967 |
| 3565 | 8 | 45.829 | Marker825843 | 0.721145051 | 0.04047619 | -0.191991342 | 1.987235967 |
| 3566 | 8 | 46.579 | Marker825841 | 0.811410202 | 0.047313773 | -0.198423146 | 2.174387142 |
| 3567 | 8 | 46.829 | Marker825842 | 0.790606044 | 0.045833333 | -0.19702381 | 2.132529504 |
| 3568 | 8 | 48.379 | Marker824904 | 0.531721444 | 0.058313521 | -0.184260937 | 2.031965325 |
| 3569 | 8 | 51.032 | Marker824220 | 0.201159877 | 0.055555556 | -0.185358255 | 2.018270381 |
| 3570 | 8 | 51.282 | Marker824219 | 0.231287609 | 0.063903263 | -0.20350677 | 2.47171935 |
| 3571 | 8 | 51.782 | Marker824227 | 0.219979211 | 0.062267762 | -0.200528662 | 2.390513664 |
| 3572 | 8 | 52.832 | Marker824229 | 0.313333383 | 0.056198076 | -0.226242656 | 2.853184126 |
| 3573 | 8 | 56.589 | Marker828182 | 0.018475775 | -0.01275615 | -0.12633365 | 0.809408701 |
| 3574 | 8 | 56.589 | Marker828183 | 0.018475775 | -0.01275615 | -0.12633365 | 0.809408701 |
| 3575 | 8 | 56.589 | Marker828185 | 0.018475775 | -0.01275615 | -0.12633365 | 0.809408701 |
| 3576 | 8 | 56.589 | Marker828184 | 0.018475775 | -0.01275615 | -0.12633365 | 0.809408701 |
| 3577 | 8 | 56.589 | Marker828181 | 0.018475775 | -0.01275615 | -0.12633365 | 0.809408701 |
| 3578 | 8 | 59.342 | Marker829897 | 0.229299173 | -0.016355814 | -0.170507201 | 1.470952239 |
| 3579 | 8 | 61.309 | Marker828751 | 0.796739561 | 0.036805556 | -0.240438034 | 2.989132514 |
| 3580 | 8 | 61.309 | Marker828752 | 0.796739561 | 0.036805556 | -0.240438034 | 2.989132514 |
| 3581 | 8 | 61.913 | Marker828585 | 0.836178259 | 0.025876123 | -0.239919709 | 2.906289684 |
| 3582 | 8 | 61.913 | Marker828584 | 0.836178259 | 0.025876123 | -0.239919709 | 2.906289684 |
| 3583 | 8 | 61.913 | Marker828586 | 0.836178259 | 0.025876123 | -0.239919709 | 2.906289684 |
| 3584 | 8 | 62.516 | Marker829094 | 0.815863145 | 0.04093432 | -0.241185156 | 3.040352679 |
| 3585 | 8 | 62.516 | Marker828750 | 0.815863145 | 0.04093432 | -0.241185156 | 3.040352679 |
| 3586 | 8 | 62.516 | Marker828480 | 0.815863145 | 0.04093432 | -0.241185156 | 3.040352679 |
| 3587 | 8 | 62.516 | Marker828479 | 0.815863145 | 0.04093432 | -0.241185156 | 3.040352679 |
| 3588 | 8 | 62.516 | Marker828402 | 0.815863145 | 0.04093432 | -0.241185156 | 3.040352679 |
| 3589 | 8 | 63.618 | Marker826567 | 0.606386071 | 0.05377907 | -0.207080957 | 2.416558082 |
| 3590 | 8 | 64.368 | Marker827442 | 0.388283212 | 0.065258662 | -0.186347097 | 2.161999104 |
| 3591 | 8 | 64.368 | Marker827359 | 0.388283212 | 0.065258662 | -0.186347097 | 2.161999104 |
| 3592 | 8 | 64.368 | Marker827450 | 0.388283212 | 0.065258662 | -0.186347097 | 2.161999104 |
| 3593 | 8 | 64.368 | Marker827358 | 0.388283212 | 0.065258662 | -0.186347097 | 2.161999104 |
| 3594 | 8 | 64.618 | Marker827439 | 0.395817249 | 0.06627907 | -0.185509839 | 2.1611377 |
| 3595 | 8 | 64.618 | Marker827438 | 0.395817249 | 0.06627907 | -0.185509839 | 2.1611377 |
| 3596 | 8 | 64.618 | Marker827437 | 0.395817249 | 0.06627907 | -0.185509839 | 2.1611377 |
| 3597 | 8 | 65.118 | Marker827843 | 0.292100944 | 0.068027211 | -0.188935952 | 2.249334675 |
| 3598 | 8 | 67.42 | Marker831004 | 0.42398684 | 0.076923077 | -0.202779893 | 2.654114024 |
| 3599 | 8 | 67.42 | Marker831003 | 0.42398684 | 0.076923077 | -0.202779893 | 2.654114024 |
| 3600 | 8 | 67.67 | Marker830856 | 0.400530545 | 0.087264151 | -0.182735849 | 2.458495625 |
| 3601 | 8 | 69.47 | Marker830884 | 0.293483823 | 0.076023392 | -0.156725146 | 1.827675719 |
| 3602 | 8 | 69.47 | Marker830885 | 0.293483823 | 0.076023392 | -0.156725146 | 1.827675719 |
| 3603 | 8 | 69.47 | Marker830887 | 0.293483823 | 0.076023392 | -0.156725146 | 1.827675719 |
| 3604 | 8 | 69.97 | Marker830539 | 0.309415052 | 0.083878858 | -0.138343365 | 1.698463926 |
| 3605 | 8 | 70.22 | Marker830536 | 0.309106725 | 0.081988071 | -0.135403233 | 1.625112335 |
| 3606 | 8 | 70.22 | Marker830535 | 0.309106725 | 0.081988071 | -0.135403233 | 1.625112335 |
| 3607 | 8 | 71.821 | Marker830515 | 0.580351873 | 0.078081092 | -0.134684866 | 1.547789448 |
| 3608 | 8 | 72.071 | Marker830514 | 0.665830127 | 0.091263331 | -0.158071186 | 2.124514381 |
| 3609 | 8 | 72.321 | Marker830513 | 0.673717708 | 0.090719064 | -0.158512381 | 2.12060209 |
| 3610 | 8 | 72.821 | Marker830306 | 0.508778489 | 0.092389246 | -0.161129462 | 2.194654058 |
| 3611 | 8 | 74.071 | Marker830344 | 0.504524537 | 0.092219862 | -0.162028604 | 2.205467995 |
| 3612 | 8 | 75.071 | Marker831152 | 0.535317591 | 0.092222222 | -0.162026144 | 2.205476225 |
| 3613 | 8 | 75.321 | Marker831151 | 0.425880018 | 0.095 | -0.164708738 | 2.304711117 |
| 3614 | 8 | 76.923 | Marker832304 | 0.249419142 | 0.095 | -0.164708738 | 2.304711117 |
| 3615 | 8 | 76.923 | Marker832189 | 0.249419142 | 0.095 | -0.164708738 | 2.304711117 |
| 3616 | 8 | 76.923 | Marker832190 | 0.249419142 | 0.095 | -0.164708738 | 2.304711117 |
| 3617 | 8 | 76.923 | Marker832303 | 0.249419142 | 0.095 | -0.164708738 | 2.304711117 |
| 3618 | 8 | 76.923 | Marker832191 | 0.249419142 | 0.095 | -0.164708738 | 2.304711117 |
| 3619 | 8 | 77.173 | Marker832307 | 0.270758002 | 0.095539057 | -0.164256907 | 2.308584517 |
| 3620 | 8 | 78.974 | Marker833196 | 0.202898008 | 0.086867305 | -0.145690834 | 1.855791368 |
| 3621 | 8 | 78.974 | Marker833199 | 0.202898008 | 0.086867305 | -0.145690834 | 1.855791368 |
| 3622 | 8 | 78.974 | Marker833200 | 0.202898008 | 0.086867305 | -0.145690834 | 1.855791368 |
| 3623 | 8 | 78.974 | Marker833195 | 0.202898008 | 0.086867305 | -0.145690834 | 1.855791368 |
| 3624 | 8 | 78.974 | Marker833551 | 0.202898008 | 0.086867305 | -0.145690834 | 1.855791368 |
| 3625 | 8 | 79.474 | Marker833685 | 0.202898008 | 0.086867305 | -0.145690834 | 1.855791368 |
| 3626 | 8 | 81.024 | Marker834563 | 0.212462715 | 0.073636364 | -0.163345102 | 1.892850786 |
| 3627 | 8 | 81.024 | Marker834641 | 0.212462715 | 0.073636364 | -0.163345102 | 1.892850786 |
| 3628 | 8 | 81.024 | Marker834640 | 0.212462715 | 0.073636364 | -0.163345102 | 1.892850786 |
| 3629 | 8 | 81.024 | Marker834642 | 0.212462715 | 0.073636364 | -0.163345102 | 1.892850786 |
| 3630 | 8 | 81.024 | Marker834643 | 0.212462715 | 0.073636364 | -0.163345102 | 1.892850786 |
| 3631 | 8 | 81.274 | Marker834541 | 0.244788745 | 0.074420677 | -0.162655971 | 1.894386698 |
| 3632 | 8 | 81.274 | Marker834539 | 0.244788745 | 0.074420677 | -0.162655971 | 1.894386698 |
| 3633 | 8 | 82.825 | Marker835940 | 0.31897952 | 0.075 | -0.204126214 | 2.649565186 |
| 3634 | 8 | 82.825 | Marker835941 | 0.31897952 | 0.075 | -0.204126214 | 2.649565186 |
| 3635 | 8 | 83.575 | Marker835450 | 0.097034772 | 0.070358534 | -0.193232033 | 2.364590407 |
| 3636 | 8 | 84.575 | Marker837043 | 0.291355113 | 0.065524614 | -0.221940892 | 2.879486139 |
| 3637 | 8 | 85.325 | Marker837198 | 0.084115601 | 0.04138796 | -0.216407385 | 2.485946951 |
| 3638 | 8 | 86.325 | Marker837546 | 0.03187239 | 0.021477318 | -0.253144309 | 3.206950155 |
| 3639 | 8 | 89.481 | Marker839911 | 0.629788197 | 0.067230274 | -0.297852649 | 4.843919329 |
| 3640 | 8 | 90.731 | Marker840148 | 0.255706762 | 0.066789216 | -0.275472689 | 4.206441 |
| 3641 | 8 | 91.231 | Marker840421 | 0.246902528 | 0.076177905 | -0.256623839 | 3.856487441 |
| 3642 | 8 | 91.481 | Marker840415 | 0.246768099 | 0.075416667 | -0.256022727 | 3.829005809 |
| 3643 | 8 | 92.231 | Marker842164 | 0.221223443 | 0.075576241 | -0.218958594 | 2.966896223 |
| 3644 | 8 | 92.481 | Marker842165 | 0.237772043 | 0.078351449 | -0.221353067 | 3.064532307 |
| 3645 | 8 | 92.731 | Marker843715 | 0.240620019 | 0.077243293 | -0.22208781 | 3.06200446 |
| 3646 | 8 | 92.731 | Marker843716 | 0.240620019 | 0.077243293 | -0.22208781 | 3.06200446 |
| 3647 | 8 | 92.981 | Marker843776 | 0.291089818 | 0.085106383 | -0.240033051 | 3.606198936 |
| 3648 | 8 | 94.334 | Marker844839 | 0.396560981 | 0.080441552 | -0.274577982 | 4.396527893 |
| 3649 | 8 | 95.084 | Marker841205 | 0.290495987 | 0.064999891 | -0.243089606 | 3.355563232 |
| 3650 | 8 | 95.584 | Marker841197 | 0.289773093 | 0.067050354 | -0.240745713 | 3.328697958 |
| 3651 | 8 | 98.738 | Marker854309 | 0.272694304 | 0.072977636 | -0.180188513 | 2.16605926 |
| 3652 | 8 | 98.738 | Marker854308 | 0.272694304 | 0.072977636 | -0.180188513 | 2.16605926 |
| 3653 | 8 | 98.988 | Marker854310 | 0.260676321 | 0.070459333 | -0.178000026 | 2.088699014 |
| 3654 | 8 | 100.288 | Marker855292 | 0.255898185 | 0.072576832 | -0.18051334 | 2.165518523 |
| 3655 | 8 | 100.538 | Marker855229 | 0.255898185 | 0.072576832 | -0.18051334 | 2.165518523 |
| 3656 | 8 | 100.538 | Marker857391 | 0.255898185 | 0.072576832 | -0.18051334 | 2.165518523 |
| 3657 | 8 | 100.538 | Marker856713 | 0.255898185 | 0.072576832 | -0.18051334 | 2.165518523 |
| 3658 | 8 | 100.538 | Marker855286 | 0.255898185 | 0.072576832 | -0.18051334 | 2.165518523 |
| 3659 | 8 | 100.538 | Marker855233 | 0.255898185 | 0.072576832 | -0.18051334 | 2.165518523 |
| 3660 | 8 | 100.538 | Marker857351 | 0.255898185 | 0.072576832 | -0.18051334 | 2.165518523 |
| 3661 | 8 | 100.538 | Marker856011 | 0.255898185 | 0.072576832 | -0.18051334 | 2.165518523 |
| 3662 | 8 | 100.538 | Marker857669 | 0.255898185 | 0.072576832 | -0.18051334 | 2.165518523 |
| 3663 | 8 | 100.788 | Marker861728 | 0.212519118 | 0.062673451 | -0.200215257 | 2.389772154 |
| 3664 | 8 | 100.788 | Marker857694 | 0.212519118 | 0.062673451 | -0.200215257 | 2.389772154 |
| 3665 | 8 | 100.788 | Marker857695 | 0.212519118 | 0.062673451 | -0.200215257 | 2.389772154 |
| 3666 | 8 | 100.788 | Marker857693 | 0.212519118 | 0.062673451 | -0.200215257 | 2.389772154 |
| 3667 | 8 | 100.788 | Marker857692 | 0.212519118 | 0.062673451 | -0.200215257 | 2.389772154 |
| 3668 | 8 | 100.788 | Marker857691 | 0.212519118 | 0.062673451 | -0.200215257 | 2.389772154 |
| 3669 | 8 | 101.288 | Marker863262 | 0.256780394 | 0.075120773 | -0.182877586 | 2.248141267 |
| 3670 | 8 | 101.538 | Marker863404 | 0.201399083 | 0.072576832 | -0.18051334 | 2.165518523 |
| 3671 | 8 | 101.538 | Marker862093 | 0.201399083 | 0.072576832 | -0.18051334 | 2.165518523 |
| 3672 | 8 | 102.039 | Marker864210 | 0.235795746 | 0.067800454 | -0.176106818 | 2.016230654 |
| 3673 | 8 | 102.039 | Marker864211 | 0.235795746 | 0.067800454 | -0.176106818 | 2.016230654 |
| 3674 | 8 | 102.289 | Marker864031 | 0.293358276 | 0.078153989 | -0.155951192 | 1.851360497 |
| 3675 | 8 | 102.289 | Marker857944 | 0.293358276 | 0.078153989 | -0.155951192 | 1.851360497 |
| 3676 | 8 | 102.789 | Marker858476 | 0.560263196 | 0.104206963 | -0.200690668 | 3.146469908 |
| 3677 | 8 | 102.789 | Marker858583 | 0.560263196 | 0.104206963 | -0.200690668 | 3.146469908 |
| 3678 | 8 | 103.539 | Marker845668 | 0.691341569 | 0.105437352 | -0.159405606 | 2.447838852 |
| 3679 | 8 | 103.539 | Marker845670 | 0.691341569 | 0.105437352 | -0.159405606 | 2.447838852 |
| 3680 | 8 | 103.539 | Marker845669 | 0.691341569 | 0.105437352 | -0.159405606 | 2.447838852 |
| 3681 | 8 | 103.539 | Marker845671 | 0.691341569 | 0.105437352 | -0.159405606 | 2.447838852 |
| 3682 | 8 | 103.789 | Marker845667 | 0.672282418 | 0.093568665 | -0.180618408 | 2.544170575 |
| 3683 | 8 | 103.789 | Marker858417 | 0.672282418 | 0.093568665 | -0.180618408 | 2.544170575 |
| 3684 | 8 | 105.339 | Marker872982 | 0.891306057 | 0.100532946 | -0.198602274 | 3.024241416 |
| 3685 | 8 | 106.089 | Marker868624 | 0.866347988 | 0.094285714 | -0.194285714 | 2.80955747 |
| 3686 | 8 | 106.089 | Marker868620 | 0.866347988 | 0.094285714 | -0.194285714 | 2.80955747 |
| 3687 | 8 | 106.339 | Marker868623 | 0.856466615 | 0.092107857 | -0.198458437 | 2.845935582 |
| 3688 | 8 | 107.339 | Marker865807 | 0.791135534 | 0.095153061 | -0.197930839 | 2.897329458 |
| 3689 | 8 | 107.339 | Marker865808 | 0.791135534 | 0.095153061 | -0.197930839 | 2.897329458 |
| 3690 | 8 | 107.589 | Marker867444 | 0.459497045 | 0.0825 | -0.176191589 | 2.256036979 |
| 3691 | 8 | 107.589 | Marker867443 | 0.459497045 | 0.0825 | -0.176191589 | 2.256036979 |
| 3692 | 8 | 107.589 | Marker867403 | 0.459497045 | 0.0825 | -0.176191589 | 2.256036979 |
| 3693 | 8 | 108.339 | Marker868541 | 0.441381803 | 0.081932773 | -0.172592114 | 2.184469198 |
| 3694 | 8 | 108.339 | Marker868542 | 0.441381803 | 0.081932773 | -0.172592114 | 2.184469198 |
| 3695 | 8 | 108.339 | Marker868536 | 0.441381803 | 0.081932773 | -0.172592114 | 2.184469198 |
| 3696 | 8 | 108.589 | Marker868537 | 0.409874081 | 0.071135431 | -0.1932849 | 2.377412905 |
| 3697 | 8 | 108.589 | Marker868540 | 0.409874081 | 0.071135431 | -0.1932849 | 2.377412905 |
| 3698 | 8 | 109.089 | Marker868602 | 0.441381803 | 0.081932773 | -0.172592114 | 2.184469198 |
| 3699 | 8 | 109.839 | Marker869890 | 0.506822815 | 0.0625 | -0.235119048 | 3.134196386 |
| 3700 | 8 | 110.941 | Marker871017 | 0.607527564 | 0.08079342 | -0.230976571 | 3.319950196 |
| 3701 | 8 | 110.941 | Marker874021 | 0.607527564 | 0.08079342 | -0.230976571 | 3.319950196 |
| 3702 | 8 | 111.191 | Marker875343 | 0.541364866 | 0.073717949 | -0.2140179 | 2.832089946 |
| 3703 | 8 | 111.191 | Marker875346 | 0.541364866 | 0.073717949 | -0.2140179 | 2.832089946 |
| 3704 | 8 | 111.191 | Marker875345 | 0.541364866 | 0.073717949 | -0.2140179 | 2.832089946 |
| 3705 | 8 | 111.191 | Marker875344 | 0.541364866 | 0.073717949 | -0.2140179 | 2.832089946 |
| 3706 | 8 | 111.941 | Marker875927 | 0.629119664 | 0.083294984 | -0.229399649 | 3.328426592 |
| 3707 | 8 | 112.691 | Marker876968 | 0.614741452 | 0.07965368 | -0.224891775 | 3.164148622 |
| 3708 | 8 | 112.691 | Marker877983 | 0.614741452 | 0.07965368 | -0.224891775 | 3.164148622 |
| 3709 | 8 | 112.941 | Marker877406 | 0.616599549 | 0.07983724 | -0.224791929 | 3.165090386 |
| 3710 | 8 | 113.441 | Marker878283 | 0.578478292 | 0.073541325 | -0.237166201 | 3.34220377 |
| 3711 | 8 | 113.941 | Marker877654 | 0.58785899 | 0.069133192 | -0.246018323 | 3.485127134 |
| 3712 | 8 | 113.941 | Marker877653 | 0.58785899 | 0.069133192 | -0.246018323 | 3.485127134 |
| 3713 | 8 | 113.941 | Marker877262 | 0.58785899 | 0.069133192 | -0.246018323 | 3.485127134 |
| 3714 | 8 | 113.941 | Marker877261 | 0.58785899 | 0.069133192 | -0.246018323 | 3.485127134 |
| 3715 | 8 | 113.941 | Marker876905 | 0.58785899 | 0.069133192 | -0.246018323 | 3.485127134 |
| 3716 | 8 | 113.941 | Marker876904 | 0.58785899 | 0.069133192 | -0.246018323 | 3.485127134 |
| 3717 | 8 | 114.441 | Marker878281 | 0.625762897 | 0.080761099 | -0.224289406 | 3.169958834 |
| 3718 | 8 | 115.691 | Marker877428 | 0.344319639 | 0.050915332 | -0.202164175 | 2.285697679 |
| 3719 | 8 | 115.691 | Marker877429 | 0.344319639 | 0.050915332 | -0.202164175 | 2.285697679 |
| 3720 | 8 | 115.941 | Marker877427 | 0.354659665 | 0.052071669 | -0.2014654 | 2.284503672 |
| 3721 | 9 | 0 | Marker880130 | 0.157137744 | -0.028468324 | -0.022896619 | 0.116263244 |
| 3722 | 9 | 0 | Marker881298 | 0.157137744 | -0.028468324 | -0.022896619 | 0.116263244 |
| 3723 | 9 | 0 | Marker881301 | 0.157137744 | -0.028468324 | -0.022896619 | 0.116263244 |
| 3724 | 9 | 0.75 | Marker879760 | 0.130504085 | -0.017747858 | -0.046278186 | 0.141974025 |
| 3725 | 9 | 0.75 | Marker881583 | 0.130504085 | -0.017747858 | -0.046278186 | 0.141974025 |
| 3726 | 9 | 0.75 | Marker881396 | 0.130504085 | -0.017747858 | -0.046278186 | 0.141974025 |
| 3727 | 9 | 0.75 | Marker879761 | 0.130504085 | -0.017747858 | -0.046278186 | 0.141974025 |
| 3728 | 9 | 0.75 | Marker879766 | 0.130504085 | -0.017747858 | -0.046278186 | 0.141974025 |
| 3729 | 9 | 0.75 | Marker879762 | 0.130504085 | -0.017747858 | -0.046278186 | 0.141974025 |
| 3730 | 9 | 0.75 | Marker879738 | 0.130504085 | -0.017747858 | -0.046278186 | 0.141974025 |
| 3731 | 9 | 1.25 | Marker882059 | 0.158853316 | -0.019661734 | -0.049025135 | 0.163046947 |
| 3732 | 9 | 1.25 | Marker882009 | 0.158853316 | -0.019661734 | -0.049025135 | 0.163046947 |
| 3733 | 9 | 1.25 | Marker881861 | 0.158853316 | -0.019661734 | -0.049025135 | 0.163046947 |
| 3734 | 9 | 1.25 | Marker882735 | 0.158853316 | -0.019661734 | -0.049025135 | 0.163046947 |
| 3735 | 9 | 1.25 | Marker882732 | 0.158853316 | -0.019661734 | -0.049025135 | 0.163046947 |
| 3736 | 9 | 1.25 | Marker881863 | 0.158853316 | -0.019661734 | -0.049025135 | 0.163046947 |
| 3737 | 9 | 1.25 | Marker881584 | 0.158853316 | -0.019661734 | -0.049025135 | 0.163046947 |
| 3738 | 9 | 2 | Marker882157 | 0.234855315 | -0.03654485 | -0.085904129 | 0.517289532 |
| 3739 | 9 | 2.75 | Marker886317 | 0.251556214 | -0.058547532 | -0.040566232 | 0.462793123 |
| 3740 | 9 | 2.75 | Marker882156 | 0.251556214 | -0.058547532 | -0.040566232 | 0.462793123 |
| 3741 | 9 | 6.658 | Marker888449 | 0.182621611 | -0.029545455 | -0.030525847 | 0.143733274 |
| 3742 | 9 | 7.908 | Marker888465 | 0.308020926 | -0.064814815 | -0.006562388 | 0.465111208 |
| 3743 | 9 | 7.908 | Marker889275 | 0.308020926 | -0.064814815 | -0.006562388 | 0.465111208 |
| 3744 | 9 | 8.908 | Marker889276 | 0.317367677 | -0.070192308 | 0.030998168 | 0.585575934 |
| 3745 | 9 | 9.158 | Marker889272 | 0.28700408 | -0.059669811 | 0.05186865 | 0.518998679 |
| 3746 | 9 | 9.158 | Marker889347 | 0.28700408 | -0.059669811 | 0.05186865 | 0.518998679 |
| 3747 | 9 | 9.158 | Marker889369 | 0.28700408 | -0.059669811 | 0.05186865 | 0.518998679 |
| 3748 | 9 | 9.158 | Marker889348 | 0.28700408 | -0.059669811 | 0.05186865 | 0.518998679 |
| 3749 | 9 | 9.158 | Marker889271 | 0.28700408 | -0.059669811 | 0.05186865 | 0.518998679 |
| 3750 | 9 | 9.158 | Marker890439 | 0.28700408 | -0.059669811 | 0.05186865 | 0.518998679 |
| 3751 | 9 | 9.158 | Marker889345 | 0.28700408 | -0.059669811 | 0.05186865 | 0.518998679 |
| 3752 | 9 | 9.158 | Marker889346 | 0.28700408 | -0.059669811 | 0.05186865 | 0.518998679 |
| 3753 | 9 | 9.658 | Marker890438 | 0.24114012 | -0.048076923 | 0.027394775 | 0.288976804 |
| 3754 | 9 | 10.159 | Marker890178 | 0.203324843 | -0.058823529 | 0.005991285 | 0.381833399 |
| 3755 | 9 | 10.409 | Marker890540 | 0.204683349 | -0.06 | 0.004220183 | 0.396501866 |
| 3756 | 9 | 10.409 | Marker891115 | 0.204683349 | -0.06 | 0.004220183 | 0.396501866 |
| 3757 | 9 | 10.409 | Marker890539 | 0.204683349 | -0.06 | 0.004220183 | 0.396501866 |
| 3758 | 9 | 10.909 | Marker892023 | 0.176713847 | -0.058823529 | 0.005396654 | 0.381562836 |
| 3759 | 9 | 10.909 | Marker892764 | 0.176713847 | -0.058823529 | 0.005396654 | 0.381562836 |
| 3760 | 9 | 10.909 | Marker891206 | 0.176713847 | -0.058823529 | 0.005396654 | 0.381562836 |
| 3761 | 9 | 11.409 | Marker892776 | 0.139553937 | -0.063717018 | -0.00507444 | 0.448544491 |
| 3762 | 9 | 12.159 | Marker891061 | 0.166331322 | -0.057692308 | 0.007122507 | 0.367957621 |
| 3763 | 9 | 12.159 | Marker891062 | 0.166331322 | -0.057692308 | 0.007122507 | 0.367957621 |
| 3764 | 9 | 12.159 | Marker891063 | 0.166331322 | -0.057692308 | 0.007122507 | 0.367957621 |
| 3765 | 9 | 13.459 | Marker891026 | 0.066433929 | -0.031265246 | -0.030854471 | 0.15635122 |
| 3766 | 9 | 14.259 | Marker891053 | 0.064426791 | -0.013697142 | -0.060753267 | 0.204526403 |
| 3767 | 9 | 15.559 | Marker888902 | 0.163409221 | -0.018803325 | -0.052694229 | 0.177903191 |
| 3768 | 9 | 16.166 | Marker888901 | 0.169692745 | -0.009098454 | -0.074016965 | 0.281021081 |
| 3769 | 9 | 16.666 | Marker888897 | 0.102948547 | 0.01030829 | -0.035330754 | 0.072698553 |
| 3770 | 9 | 18.216 | Marker891041 | 0.171383164 | -0.001097928 | -0.11777433 | 0.68569358 |
| 3771 | 9 | 19.266 | Marker891040 | 0.113864063 | 0.019985932 | -0.074580498 | 0.316038142 |
| 3772 | 9 | 20.982 | Marker893354 | 0.141460574 | 0.029988183 | -0.077681194 | 0.3928249 |
| 3773 | 9 | 22.783 | Marker893053 | 0.131195401 | 0.041396104 | -0.053730419 | 0.32710858 |
| 3774 | 9 | 24.284 | Marker893757 | 0.089612673 | 0.030648537 | -0.034603811 | 0.160570114 |
| 3775 | 9 | 25.834 | Marker894670 | 0.048260547 | 0.007148011 | 0.001860094 | 0.005816836 |
| 3776 | 9 | 27.44 | Marker894946 | 0.090732378 | -0.002932551 | -0.060874609 | 0.184363964 |
| 3777 | 9 | 27.44 | Marker897389 | 0.090732378 | -0.002932551 | -0.060874609 | 0.184363964 |
| 3778 | 9 | 29.992 | Marker897484 | 0.364466693 | -0.008261494 | -0.10143915 | 0.517443528 |
| 3779 | 9 | 30.243 | Marker897483 | 0.364466693 | -0.008261494 | -0.10143915 | 0.517443528 |
| 3780 | 9 | 31.744 | Marker897376 | 0.245355933 | -0.011992263 | -0.065519699 | 0.229359821 |
| 3781 | 9 | 32.244 | Marker897375 | 0.279982561 | -0.015381692 | -0.076619205 | 0.318236014 |
| 3782 | 9 | 33.244 | Marker897888 | 0.555567662 | -0.033444816 | -0.110300328 | 0.730920552 |
| 3783 | 9 | 33.244 | Marker897887 | 0.555567662 | -0.033444816 | -0.110300328 | 0.730920552 |
| 3784 | 9 | 33.244 | Marker897884 | 0.555567662 | -0.033444816 | -0.110300328 | 0.730920552 |
| 3785 | 9 | 33.244 | Marker897883 | 0.555567662 | -0.033444816 | -0.110300328 | 0.730920552 |
| 3786 | 9 | 33.744 | Marker897890 | 0.552086332 | -0.034307334 | -0.112621587 | 0.763236377 |
| 3787 | 9 | 34.599 | Marker897881 | 0.522108413 | -0.042447447 | -0.092350431 | 0.6267913 |
| 3788 | 9 | 35.599 | Marker900212 | 0.482408051 | -0.009259259 | -0.110166289 | 0.610941741 |
| 3789 | 9 | 36.349 | Marker900210 | 0.402175744 | 0.001010101 | -0.089482453 | 0.395559931 |
| 3790 | 9 | 37.099 | Marker900211 | 0.365554316 | -0.009090909 | -0.069387755 | 0.248128604 |
| 3791 | 9 | 37.349 | Marker900215 | 0.366458414 | -0.01010101 | -0.070707071 | 0.259546137 |
| 3792 | 9 | 37.849 | Marker900702 | 0.440523907 | -0.019393202 | -0.08891873 | 0.435179235 |
| 3793 | 9 | 38.099 | Marker900826 | 0.448015119 | -0.02058319 | -0.091492281 | 0.463643638 |
| 3794 | 9 | 38.099 | Marker900703 | 0.448015119 | -0.02058319 | -0.091492281 | 0.463643638 |
| 3795 | 9 | 38.099 | Marker900827 | 0.448015119 | -0.02058319 | -0.091492281 | 0.463643638 |
| 3796 | 9 | 38.099 | Marker900704 | 0.448015119 | -0.02058319 | -0.091492281 | 0.463643638 |
| 3797 | 9 | 38.849 | Marker900319 | 0.50428529 | -0.023667407 | -0.090779835 | 0.472728971 |
| 3798 | 9 | 39.355 | Marker900318 | 0.496008533 | -0.00563204 | -0.091812999 | 0.420924866 |
| 3799 | 9 | 39.355 | Marker900098 | 0.496008533 | -0.00563204 | -0.091812999 | 0.420924866 |
| 3800 | 9 | 39.355 | Marker900094 | 0.496008533 | -0.00563204 | -0.091812999 | 0.420924866 |
| 3801 | 9 | 40.355 | Marker902490 | 0.349434535 | 0.005836925 | -0.033302386 | 0.058188353 |
| 3802 | 9 | 42.656 | Marker902872 | 0.186992849 | 0.011972027 | -0.009361098 | 0.019894158 |
| 3803 | 9 | 42.906 | Marker904340 | 0.213451868 | 0.011404562 | -0.008372421 | 0.017598752 |
| 3804 | 9 | 45.009 | Marker904339 | 0.024252649 | 0.022634508 | 0.016270672 | 0.070121304 |
| 3805 | 9 | 45.259 | Marker904336 | 0.023594724 | 0.022297808 | 0.01513324 | 0.066635515 |
| 3806 | 9 | 46.967 | Marker904332 | 0.043346126 | 0.000287075 | 0.033336699 | 0.054935139 |
| 3807 | 9 | 46.967 | Marker904330 | 0.043346126 | 0.000287075 | 0.033336699 | 0.054935139 |
| 3808 | 9 | 46.967 | Marker904329 | 0.043346126 | 0.000287075 | 0.033336699 | 0.054935139 |
| 3809 | 9 | 47.967 | Marker905714 | 0.178835276 | -0.012743628 | 0.019046928 | 0.035348089 |
| 3810 | 9 | 49.468 | Marker904338 | 0.010567964 | 0.003220612 | 0.052525856 | 0.137764977 |
| 3811 | 9 | 50.218 | Marker904331 | 0.167674217 | -0.006825754 | 0.029471041 | 0.047670694 |
| 3812 | 9 | 50.468 | Marker904337 | 0.165159248 | -0.007107023 | 0.030222797 | 0.050294096 |
| 3813 | 9 | 51.968 | Marker905713 | 0.16008927 | 0.003472222 | 0.014656433 | 0.012032931 |
| 3814 | 9 | 52.718 | Marker905712 | 0.153224948 | 0.001885446 | 0.010875482 | 0.0062724 |
| 3815 | 9 | 54.519 | Marker905635 | 0.146769901 | -0.006259984 | 0.027299666 | 0.040821001 |
| 3816 | 9 | 55.37 | Marker905636 | 0.235404727 | 0.001682836 | 0.042709276 | 0.09056686 |
| 3817 | 9 | 55.87 | Marker906094 | 0.232855391 | 0.00619207 | 0.034292891 | 0.062710675 |
| 3818 | 9 | 56.12 | Marker905638 | 0.232060082 | 0.001893939 | 0.04215368 | 0.088334955 |
| 3819 | 9 | 56.12 | Marker906091 | 0.232060082 | 0.001893939 | 0.04215368 | 0.088334955 |
| 3820 | 9 | 56.12 | Marker906090 | 0.232060082 | 0.001893939 | 0.04215368 | 0.088334955 |
| 3821 | 9 | 56.62 | Marker906101 | 0.303610271 | -0.013353532 | 0.012894501 | 0.027518919 |
| 3822 | 9 | 57.37 | Marker906280 | 0.265738249 | -0.018707483 | 0.042388654 | 0.125829586 |
| 3823 | 9 | 58.371 | Marker907093 | 0.182448823 | -0.009250156 | 0.060608433 | 0.189881761 |
| 3824 | 9 | 59.371 | Marker907471 | 0.199919881 | -0.017507003 | 0.045221935 | 0.133312937 |
| 3825 | 9 | 59.621 | Marker907688 | 0.207628786 | -0.029411765 | 0.067675614 | 0.317820656 |
| 3826 | 9 | 59.621 | Marker907685 | 0.207628786 | -0.029411765 | 0.067675614 | 0.317820656 |
| 3827 | 9 | 59.621 | Marker907689 | 0.207628786 | -0.029411765 | 0.067675614 | 0.317820656 |
| 3828 | 9 | 59.621 | Marker910470 | 0.207628786 | -0.029411765 | 0.067675614 | 0.317820656 |
| 3829 | 9 | 59.621 | Marker907470 | 0.207628786 | -0.029411765 | 0.067675614 | 0.317820656 |
| 3830 | 9 | 59.621 | Marker907092 | 0.207628786 | -0.029411765 | 0.067675614 | 0.317820656 |
| 3831 | 9 | 59.871 | Marker908538 | 0.207628786 | -0.029411765 | 0.067675614 | 0.317820656 |
| 3832 | 9 | 59.871 | Marker908466 | 0.207628786 | -0.029411765 | 0.067675614 | 0.317820656 |
| 3833 | 9 | 60.121 | Marker908671 | 0.207628786 | -0.029411765 | 0.067675614 | 0.317820656 |
| 3834 | 9 | 60.621 | Marker908780 | 0.22082803 | -0.03 | 0.066153846 | 0.311614221 |
| 3835 | 9 | 60.621 | Marker908779 | 0.22082803 | -0.03 | 0.066153846 | 0.311614221 |
| 3836 | 9 | 60.621 | Marker908778 | 0.22082803 | -0.03 | 0.066153846 | 0.311614221 |
| 3837 | 9 | 61.121 | Marker911820 | 0.370580913 | -0.027310924 | 0.025802629 | 0.113670124 |
| 3838 | 9 | 61.121 | Marker908777 | 0.370580913 | -0.027310924 | 0.025802629 | 0.113670124 |
| 3839 | 9 | 61.371 | Marker911281 | 0.444128492 | -0.036172161 | 0.00797948 | 0.146575485 |
| 3840 | 9 | 61.371 | Marker911282 | 0.444128492 | -0.036172161 | 0.00797948 | 0.146575485 |
| 3841 | 9 | 61.621 | Marker916290 | 0.44442665 | -0.035881801 | 0.007035647 | 0.143639632 |
| 3842 | 9 | 61.621 | Marker911724 | 0.44442665 | -0.035881801 | 0.007035647 | 0.143639632 |
| 3843 | 9 | 61.621 | Marker912542 | 0.44442665 | -0.035881801 | 0.007035647 | 0.143639632 |
| 3844 | 9 | 61.621 | Marker911278 | 0.44442665 | -0.035881801 | 0.007035647 | 0.143639632 |
| 3845 | 9 | 61.621 | Marker911725 | 0.44442665 | -0.035881801 | 0.007035647 | 0.143639632 |
| 3846 | 9 | 62.871 | Marker916286 | 0.332140167 | -0.034974689 | 0.008596232 | 0.137684982 |
| 3847 | 9 | 62.871 | Marker916288 | 0.332140167 | -0.034974689 | 0.008596232 | 0.137684982 |
| 3848 | 9 | 62.871 | Marker916287 | 0.332140167 | -0.034974689 | 0.008596232 | 0.137684982 |
| 3849 | 9 | 63.121 | Marker920151 | 0.331218361 | -0.034538825 | 0.009364558 | 0.134991544 |
| 3850 | 9 | 63.621 | Marker919696 | 0.330294415 | -0.034101174 | 0.010136033 | 0.132389052 |
| 3851 | 9 | 63.871 | Marker922191 | 0.332140167 | -0.034974689 | 0.008596232 | 0.137684982 |
| 3852 | 9 | 64.121 | Marker914952 | 0.330294415 | -0.034101174 | 0.010136033 | 0.132389052 |
| 3853 | 9 | 65.371 | Marker924351 | 0.292479123 | -0.012037037 | 0.005924128 | 0.01754485 |
| 3854 | 9 | 65.371 | Marker922192 | 0.292479123 | -0.012037037 | 0.005924128 | 0.01754485 |
| 3855 | 9 | 65.371 | Marker924352 | 0.292479123 | -0.012037037 | 0.005924128 | 0.01754485 |
| 3856 | 9 | 65.621 | Marker922707 | 0.310573861 | -0.012735849 | 0.004571843 | 0.018772204 |
| 3857 | 9 | 66.121 | Marker920468 | 0.34662541 | -0.013340724 | 0.003432663 | 0.020079836 |
| 3858 | 9 | 66.371 | Marker924284 | 0.346875293 | -0.013461538 | 0.003205128 | 0.020366329 |
| 3859 | 9 | 66.871 | Marker929244 | 0.445332054 | -0.035 | -0.038271028 | 0.209596256 |
| 3860 | 9 | 67.871 | Marker929170 | 0.643692362 | -0.045787546 | -0.060372349 | 0.415805324 |
| 3861 | 9 | 67.871 | Marker929169 | 0.643692362 | -0.045787546 | -0.060372349 | 0.415805324 |
| 3862 | 9 | 68.371 | Marker928522 | 0.642548432 | -0.045787546 | -0.060372349 | 0.415805324 |
| 3863 | 9 | 68.371 | Marker929109 | 0.642548432 | -0.045787546 | -0.060372349 | 0.415805324 |
| 3864 | 9 | 68.621 | Marker929108 | 0.642593817 | -0.045894275 | -0.060209046 | 0.415907092 |
| 3865 | 9 | 69.372 | Marker928328 | 0.308325726 | -0.035583464 | -0.040993956 | 0.225009326 |
| 3866 | 9 | 69.622 | Marker928316 | 0.379335124 | -0.044358974 | -0.058603989 | 0.390939981 |
| 3867 | 9 | 69.872 | Marker928327 | 0.384093733 | -0.044519088 | -0.058357417 | 0.391077879 |
| 3868 | 9 | 70.372 | Marker928128 | 0.377438037 | -0.042986425 | -0.056885299 | 0.36766036 |
| 3869 | 9 | 70.372 | Marker928129 | 0.377438037 | -0.042986425 | -0.056885299 | 0.36766036 |
| 3870 | 9 | 70.622 | Marker928126 | 0.349884863 | -0.041666667 | -0.055212869 | 0.345840654 |
| 3871 | 9 | 71.122 | Marker932701 | 0.35218514 | -0.043564322 | -0.057608934 | 0.377374835 |
| 3872 | 9 | 71.622 | Marker928740 | 0.286237696 | -0.034358974 | -0.039344729 | 0.20884457 |
| 3873 | 9 | 73.172 | Marker934045 | 0.259853945 | -0.040691489 | -0.002055126 | 0.182538628 |
| 3874 | 9 | 73.425 | Marker932699 | 0.259853945 | -0.040691489 | -0.002055126 | 0.182538628 |
| 3875 | 9 | 73.425 | Marker932700 | 0.259853945 | -0.040691489 | -0.002055126 | 0.182538628 |
| 3876 | 9 | 74.975 | Marker934044 | 0.285177248 | -0.0268757 | -0.02593585 | 0.113981011 |
| 3877 | 9 | 75.225 | Marker933587 | 0.258633637 | -0.015350877 | -0.004919393 | 0.027260897 |
| 3878 | 9 | 75.225 | Marker933008 | 0.258633637 | -0.015350877 | -0.004919393 | 0.027260897 |
| 3879 | 9 | 75.225 | Marker933071 | 0.258633637 | -0.015350877 | -0.004919393 | 0.027260897 |
| 3880 | 9 | 75.225 | Marker933016 | 0.258633637 | -0.015350877 | -0.004919393 | 0.027260897 |
| 3881 | 9 | 75.225 | Marker933588 | 0.258633637 | -0.015350877 | -0.004919393 | 0.027260897 |
| 3882 | 9 | 75.475 | Marker933280 | 0.246942423 | -0.014500537 | -0.003495752 | 0.023830729 |
| 3883 | 9 | 75.475 | Marker933284 | 0.246942423 | -0.014500537 | -0.003495752 | 0.023830729 |
| 3884 | 9 | 75.475 | Marker933283 | 0.246942423 | -0.014500537 | -0.003495752 | 0.023830729 |
| 3885 | 9 | 75.475 | Marker933279 | 0.246942423 | -0.014500537 | -0.003495752 | 0.023830729 |
| 3886 | 9 | 75.475 | Marker933278 | 0.246942423 | -0.014500537 | -0.003495752 | 0.023830729 |
| 3887 | 9 | 75.475 | Marker933586 | 0.246942423 | -0.014500537 | -0.003495752 | 0.023830729 |
| 3888 | 9 | 76.475 | Marker933277 | 0.246273372 | -0.027658432 | 0.018753051 | 0.100593311 |
| 3889 | 9 | 76.475 | Marker935235 | 0.246273372 | -0.027658432 | 0.018753051 | 0.100593311 |
| 3890 | 9 | 77.475 | Marker935239 | 0.14844081 | -0.014358974 | -0.000826211 | 0.022740136 |
| 3891 | 9 | 77.725 | Marker935237 | 0.357167919 | -0.026842105 | 0.020236601 | 0.098511369 |
| 3892 | 9 | 77.725 | Marker937993 | 0.357167919 | -0.026842105 | 0.020236601 | 0.098511369 |
| 3893 | 9 | 77.725 | Marker935238 | 0.357167919 | -0.026842105 | 0.020236601 | 0.098511369 |
| 3894 | 9 | 77.725 | Marker935236 | 0.357167919 | -0.026842105 | 0.020236601 | 0.098511369 |
| 3895 | 9 | 78.475 | Marker937992 | 0.232540873 | -0.023993381 | -0.023993381 | 0.092835299 |
| 3896 | 9 | 78.725 | Marker937985 | 0.23310332 | -0.025056306 | -0.025538932 | 0.102471437 |
| 3897 | 9 | 78.975 | Marker937150 | 0.127923626 | -0.012609649 | -0.046511972 | 0.125454245 |
| 3898 | 9 | 78.975 | Marker937984 | 0.127923626 | -0.012609649 | -0.046511972 | 0.125454245 |
| 3899 | 9 | 78.975 | Marker938060 | 0.127923626 | -0.012609649 | -0.046511972 | 0.125454245 |
| 3900 | 9 | 78.975 | Marker938226 | 0.127923626 | -0.012609649 | -0.046511972 | 0.125454245 |
| 3901 | 9 | 78.975 | Marker938225 | 0.127923626 | -0.012609649 | -0.046511972 | 0.125454245 |
| 3902 | 9 | 78.975 | Marker937149 | 0.127923626 | -0.012609649 | -0.046511972 | 0.125454245 |
| 3903 | 9 | 78.975 | Marker937983 | 0.127923626 | -0.012609649 | -0.046511972 | 0.125454245 |
| 3904 | 9 | 78.975 | Marker938476 | 0.127923626 | -0.012609649 | -0.046511972 | 0.125454245 |
| 3905 | 9 | 78.975 | Marker937986 | 0.127923626 | -0.012609649 | -0.046511972 | 0.125454245 |
| 3906 | 9 | 78.975 | Marker937148 | 0.127923626 | -0.012609649 | -0.046511972 | 0.125454245 |
| 3907 | 9 | 79.725 | Marker937473 | 0.141906973 | -0.013621795 | -0.045090326 | 0.121991915 |
| 3908 | 9 | 79.725 | Marker937435 | 0.141906973 | -0.013621795 | -0.045090326 | 0.121991915 |
| 3909 | 9 | 80.226 | Marker938180 | 0.139624223 | -0.001020408 | -0.063983371 | 0.20250293 |
| 3910 | 9 | 80.726 | Marker939213 | 0.248545294 | -0.013520408 | -0.042224112 | 0.109244994 |
| 3911 | 9 | 81.976 | Marker939212 | 0.154096321 | 0.009955202 | -0.083958635 | 0.357652171 |
| 3912 | 9 | 82.226 | Marker940086 | 0.156127469 | 0.008503401 | -0.082242331 | 0.34085987 |
| 3913 | 9 | 82.976 | Marker941838 | 0.144070265 | -0.003540935 | -0.061273186 | 0.18727306 |
| 3914 | 9 | 83.976 | Marker941704 | 0.468790755 | 0.028525046 | -0.120950478 | 0.805996982 |
| 3915 | 9 | 83.976 | Marker943119 | 0.468790755 | 0.028525046 | -0.120950478 | 0.805996982 |
| 3916 | 9 | 83.976 | Marker941834 | 0.468790755 | 0.028525046 | -0.120950478 | 0.805996982 |
| 3917 | 9 | 83.976 | Marker941833 | 0.468790755 | 0.028525046 | -0.120950478 | 0.805996982 |
| 3918 | 9 | 83.976 | Marker941837 | 0.468790755 | 0.028525046 | -0.120950478 | 0.805996982 |
| 3919 | 9 | 83.976 | Marker941836 | 0.468790755 | 0.028525046 | -0.120950478 | 0.805996982 |
| 3920 | 9 | 83.976 | Marker941832 | 0.468790755 | 0.028525046 | -0.120950478 | 0.805996982 |
| 3921 | 9 | 83.976 | Marker941441 | 0.468790755 | 0.028525046 | -0.120950478 | 0.805996982 |
| 3922 | 9 | 84.976 | Marker940847 | 0.348275781 | 0.027959937 | -0.120610251 | 0.798566889 |
| 3923 | 9 | 85.976 | Marker941686 | 0.270664203 | 0.016197783 | -0.094236999 | 0.464844323 |
| 3924 | 9 | 87.333 | Marker941684 | 0.335087229 | -0.001064992 | -0.104498052 | 0.539853049 |
| 3925 | 9 | 87.833 | Marker941685 | 0.31532748 | -0.007803121 | -0.089225381 | 0.401316492 |
| 3926 | 9 | 89.884 | Marker940751 | 0.177961785 | 0.00240096 | -0.109738741 | 0.595149152 |
| 3927 | 9 | 91.685 | Marker941343 | 0.515953851 | 0.020721925 | -0.138368984 | 0.987956233 |
| 3928 | 9 | 91.935 | Marker941344 | 0.507876042 | 0.019070647 | -0.135448231 | 0.941732823 |
| 3929 | 9 | 92.685 | Marker941439 | 0.460500744 | 0.0278646 | -0.116863895 | 0.754237184 |
| 3930 | 9 | 92.935 | Marker941440 | 0.471838455 | 0.029545455 | -0.119836717 | 0.799100735 |
| 3931 | 9 | 94.986 | Marker942069 | 0.186605887 | -0.000980392 | -0.067769608 | 0.227143172 |
| 3932 | 9 | 95.486 | Marker942068 | 0.208325484 | 0.006684771 | -0.08522728 | 0.362756355 |
| 3933 | 9 | 97.698 | Marker942295 | 0.015047762 | -0.010341074 | -0.042334511 | 0.101115864 |
| 3934 | 9 | 98.448 | Marker942296 | 0.006123957 | -0.029331046 | -0.038932681 | 0.171634096 |
| 3935 | 9 | 99.964 | Marker945125 | 0.061818183 | -0.004563233 | -0.014220309 | 0.012400869 |
| 3936 | 9 | 102.516 | Marker945940 | 0.063531252 | -0.013836478 | -0.032884097 | 0.075324169 |
| 3937 | 9 | 103.367 | Marker945936 | 0.072007321 | -0.019290376 | -0.047288825 | 0.153098334 |
| 3938 | 9 | 103.968 | Marker945939 | 0.063601912 | -0.012962963 | -0.031436859 | 0.068062397 |
| 3939 | 9 | 105.071 | Marker945764 | 0.01818975 | -0.006410256 | -0.054449472 | 0.151641041 |
| 3940 | 9 | 107.373 | Marker947727 | 0.000916967 | 0.000983607 | -0.034471979 | 0.058756456 |
| 3941 | 9 | 109.225 | Marker947726 | 0.013847458 | -0.002004043 | -0.007362641 | 0.003147194 |
| 3942 | 9 | 110.236 | Marker951951 | 0.043651563 | -0.011695564 | -0.048317984 | 0.13143246 |
| 3943 | 9 | 113.142 | Marker951947 | 0.063668907 | 0.003302642 | -0.018811631 | 0.018570668 |
| 3944 | 9 | 113.892 | Marker951945 | 0.038409696 | -0.005663295 | 0.000109101 | 0.003528237 |
| 3945 | 9 | 114.892 | Marker951944 | 0.024922586 | -0.006501182 | 0.03427896 | 0.062298793 |
| 3946 | 9 | 115.142 | Marker961559 | 0.04608007 | 0.004951609 | 0.011535137 | 0.00937629 |
| 3947 | 9 | 115.893 | Marker961293 | 0.04701146 | 0.00105844 | 0.019665129 | 0.01926817 |
| 3948 | 9 | 116.643 | Marker961754 | 0.038451584 | 0.016483516 | 0.022724848 | 0.056094665 |
| 3949 | 9 | 116.643 | Marker961487 | 0.038451584 | 0.016483516 | 0.022724848 | 0.056094665 |
| 3950 | 9 | 116.643 | Marker961488 | 0.038451584 | 0.016483516 | 0.022724848 | 0.056094665 |
| 3951 | 9 | 116.893 | Marker961208 | 0.037824099 | 0.016864813 | 0.024265559 | 0.061134138 |
| 3952 | 9 | 117.144 | Marker961815 | 0.035218293 | 0.017195767 | 0.024975117 | 0.064137664 |
| 3953 | 9 | 117.144 | Marker961645 | 0.035218293 | 0.017195767 | 0.024975117 | 0.064137664 |
| 3954 | 9 | 117.394 | Marker961753 | 0.067986422 | 0.027993109 | 0.004969854 | 0.087691045 |
| 3955 | 9 | 117.644 | Marker961194 | 0.067548766 | 0.029100529 | 0.003169365 | 0.093837614 |
| 3956 | 9 | 117.894 | Marker960714 | 0.058734643 | 0.038185085 | 0.021378363 | 0.184500473 |
| 3957 | 9 | 117.894 | Marker960726 | 0.058734643 | 0.038185085 | 0.021378363 | 0.184500473 |
| 3958 | 9 | 117.894 | Marker960722 | 0.058734643 | 0.038185085 | 0.021378363 | 0.184500473 |
| 3959 | 9 | 117.894 | Marker960723 | 0.058734643 | 0.038185085 | 0.021378363 | 0.184500473 |
| 3960 | 9 | 117.894 | Marker960724 | 0.058734643 | 0.038185085 | 0.021378363 | 0.184500473 |
| 3961 | 9 | 117.894 | Marker960725 | 0.058734643 | 0.038185085 | 0.021378363 | 0.184500473 |
| 3962 | 9 | 117.894 | Marker960713 | 0.058734643 | 0.038185085 | 0.021378363 | 0.184500473 |
| 3963 | 9 | 118.644 | Marker960720 | 0.06126899 | 0.038359788 | 0.022329614 | 0.188099624 |
| 3964 | 9 | 119.144 | Marker955446 | 0.057875349 | 0.038387988 | 0.022490492 | 0.188706455 |
| 3965 | 9 | 120.144 | Marker955030 | 0.052979419 | 0.046511628 | 0.044322276 | 0.33884979 |
| 3966 | 9 | 121.144 | Marker962443 | 0.053407687 | 0.046511628 | 0.044397463 | 0.339185768 |
| 3967 | 9 | 121.144 | Marker957683 | 0.053407687 | 0.046511628 | 0.044397463 | 0.339185768 |
| 3968 | 9 | 121.144 | Marker957539 | 0.053407687 | 0.046511628 | 0.044397463 | 0.339185768 |
| 3969 | 9 | 121.395 | Marker957515 | 0.053407687 | 0.046511628 | 0.044397463 | 0.339185768 |
| 3970 | 9 | 122.497 | Marker962442 | 0.012890862 | 0.035010482 | 0.026929674 | 0.172415887 |
| 3971 | 9 | 122.497 | Marker962407 | 0.012890862 | 0.035010482 | 0.026929674 | 0.172415887 |
| 3972 | 9 | 123.247 | Marker962406 | 0.036411808 | 0.021816038 | 0.053066038 | 0.193615468 |
| 3973 | 9 | 123.247 | Marker962100 | 0.036411808 | 0.021816038 | 0.053066038 | 0.193615468 |
| 3974 | 9 | 123.247 | Marker962379 | 0.036411808 | 0.021816038 | 0.053066038 | 0.193615468 |
| 3975 | 9 | 123.497 | Marker962931 | 0.037027768 | 0.021634615 | 0.051918121 | 0.186730818 |
| 3976 | 9 | 124.247 | Marker963003 | 0.052632252 | 0.030816327 | 0.030816327 | 0.153141173 |
| 3977 | 9 | 124.497 | Marker962978 | 0.128073222 | 0.040816327 | 0.050092764 | 0.311015957 |
| 3978 | 9 | 124.497 | Marker963219 | 0.128073222 | 0.040816327 | 0.050092764 | 0.311015957 |
| 3979 | 9 | 124.497 | Marker963076 | 0.128073222 | 0.040816327 | 0.050092764 | 0.311015957 |
| 3980 | 9 | 124.997 | Marker963644 | 0.051224239 | 0.031012405 | 0.031853979 | 0.157757948 |
| 3981 | 9 | 124.997 | Marker962977 | 0.051224239 | 0.031012405 | 0.031853979 | 0.157757948 |
| 3982 | 9 | 124.997 | Marker964332 | 0.051224239 | 0.031012405 | 0.031853979 | 0.157757948 |
| 3983 | 9 | 125.247 | Marker963643 | 0.052567056 | 0.030816327 | 0.030816327 | 0.153141173 |
| 3984 | 9 | 125.747 | Marker964354 | 0.03963935 | 0.041879252 | 0.007712585 | 0.196497458 |
| 3985 | 9 | 125.747 | Marker921453 | 0.03963935 | 0.041879252 | 0.007712585 | 0.196497458 |
| 3986 | 9 | 125.747 | Marker921454 | 0.03963935 | 0.041879252 | 0.007712585 | 0.196497458 |
| 3987 | 9 | 126.098 | Marker921451 | 0.05355132 | 0.07 | -0.009393939 | 0.542272796 |
| 3988 | 9 | 126.098 | Marker964640 | 0.05355132 | 0.07 | -0.009393939 | 0.542272796 |
| 3989 | 9 | 126.598 | Marker964600 | 0.047720982 | 0.06122449 | 0.009482581 | 0.417922082 |
| 3990 | 9 | 126.95 | Marker964685 | 0.117086615 | 0.072916667 | -0.012916667 | 0.591498079 |
| 3991 | 9 | 128.301 | Marker965403 | 0.175996617 | 0.071028411 | 0.031053659 | 0.606754944 |
| 3992 | 9 | 129.152 | Marker966148 | 0.076227443 | 0.052083333 | 0.02954932 | 0.344415605 |
| 3993 | 9 | 129.903 | Marker966287 | 0.037211569 | 0.052083333 | 0.030462941 | 0.347211632 |
| 3994 | 9 | 130.903 | Marker968044 | 0.055360026 | 0.041853232 | 0.012770406 | 0.201763358 |
| 3995 | 9 | 132.403 | Marker968329 | 0.045460685 | 0.031789639 | -0.007751177 | 0.113705412 |
| 3996 | 9 | 132.653 | Marker968327 | 0.031308263 | 0.022435897 | 0.011267513 | 0.062117813 |
| 3997 | 9 | 132.653 | Marker968330 | 0.031308263 | 0.022435897 | 0.011267513 | 0.062117813 |
| 3998 | 9 | 132.653 | Marker968336 | 0.031308263 | 0.022435897 | 0.011267513 | 0.062117813 |
| 3999 | 9 | 132.903 | Marker968331 | 0.031147397 | 0.031789639 | -0.007751177 | 0.113705412 |
| 4000 | 9 | 132.903 | Marker968328 | 0.031147397 | 0.031789639 | -0.007751177 | 0.113705412 |
| 4001 | 9 | 132.903 | Marker968337 | 0.031147397 | 0.031789639 | -0.007751177 | 0.113705412 |
| 4002 | 9 | 133.403 | Marker968326 | 0.036244183 | 0.01835822 | 0.019304084 | 0.056140826 |
| 4003 | 9 | 133.903 | Marker969316 | 0.033671789 | 0.021727463 | 0.012602217 | 0.060288064 |
| 4004 | 9 | 135.153 | Marker969315 | 0.005325089 | 0.011804722 | -0.048998981 | 0.13289652 |
| 4005 | 9 | 136.154 | Marker968857 | 0.009071159 | 0.003142085 | -0.028688826 | 0.041586243 |
| 4006 | 9 | 137.154 | Marker970682 | 0.012713588 | -0.007211538 | -0.007855868 | 0.008874775 |
| 4007 | 9 | 138.404 | Marker970681 | 0.01292872 | -0.015919811 | -0.026336478 | 0.062921375 |
| 4008 | 9 | 138.654 | Marker970699 | 0.014650044 | -0.015254918 | -0.027538313 | 0.063841405 |
| 4009 | 9 | 138.904 | Marker970889 | 0.006964848 | -0.00369155 | -0.049831746 | 0.124525385 |
| 4010 | 9 | 138.904 | Marker970698 | 0.006964848 | -0.00369155 | -0.049831746 | 0.124525385 |
| 4011 | 9 | 139.154 | Marker970888 | 0.008136422 | -0.012077295 | -0.068105981 | 0.24672736 |
| 4012 | 9 | 139.404 | Marker971819 | 0.004448924 | -0.002364066 | -0.087913712 | 0.382858677 |
| 4013 | 9 | 140.154 | Marker971817 | 0.020102637 | -0.015435962 | -0.11171793 | 0.646023556 |
| 4014 | 9 | 140.404 | Marker972465 | 0.011387855 | -0.006808511 | -0.093191489 | 0.435350303 |
| 4015 | 9 | 140.654 | Marker974501 | 0.022369901 | -0.012278076 | -0.104936741 | 0.563007927 |
| 4016 | 9 | 141.154 | Marker976316 | 0.013855685 | -0.016521739 | -0.073775291 | 0.301179459 |
| 4017 | 9 | 142.955 | Marker976296 | 0.023635041 | 0.003170732 | -0.143170732 | 1.013028004 |
| 4018 | 9 | 143.705 | Marker979152 | 0.039943449 | 0.026405833 | -0.147439774 | 1.143640857 |
| 4019 | 9 | 144.455 | Marker979388 | 0.052853656 | 0.016071429 | -0.168187831 | 1.421075397 |
| 4020 | 9 | 144.455 | Marker978587 | 0.052853656 | 0.016071429 | -0.168187831 | 1.421075397 |
| 4021 | 9 | 144.705 | Marker976864 | 0.076547001 | 0.0075 | -0.186191589 | 1.716466321 |
| 4022 | 9 | 144.955 | Marker978128 | 0.076971824 | 0.005365854 | -0.184233778 | 1.678360088 |
| 4023 | 9 | 144.955 | Marker977900 | 0.076971824 | 0.005365854 | -0.184233778 | 1.678360088 |
| 4024 | 9 | 144.955 | Marker978127 | 0.076971824 | 0.005365854 | -0.184233778 | 1.678360088 |
| 4025 | 9 | 145.205 | Marker977600 | 0.077681125 | 0.006934481 | -0.182844845 | 1.654781335 |
| 4026 | 9 | 145.205 | Marker977601 | 0.077681125 | 0.006934481 | -0.182844845 | 1.654781335 |
| 4027 | 9 | 145.205 | Marker977295 | 0.077681125 | 0.006934481 | -0.182844845 | 1.654781335 |
| 4028 | 9 | 145.705 | Marker974673 | 0.063724734 | 0.016025641 | -0.160349764 | 1.293927826 |
| 4029 | 9 | 145.705 | Marker977035 | 0.063724734 | 0.016025641 | -0.160349764 | 1.293927826 |
| 4030 | 9 | 145.705 | Marker974672 | 0.063724734 | 0.016025641 | -0.160349764 | 1.293927826 |
| 4031 | 9 | 145.955 | Marker975728 | 0.052771825 | 0.014087657 | -0.158506963 | 1.259091647 |
| 4032 | 9 | 146.705 | Marker974671 | 0.017319845 | 0.015357613 | -0.157334075 | 1.2445676 |

Table S3. QTL analysis of QAC2

| Number | Group | Position | Locus | LOD | Additive | Dominance | expl% |
| --- | --- | --- | --- | --- | --- | --- | --- |
| 1 | 1 | 0 | Marker809 | 0.15232923 | -0.14846568 | -0.213870921 | 1.716808365 |
| 2 | 1 | 0 | Marker803 | 0.15232923 | -0.14846568 | -0.213870921 | 1.716808365 |
| 3 | 1 | 0 | Marker808 | 0.15232923 | -0.14846568 | -0.213870921 | 1.716808365 |
| 4 | 1 | 0 | Marker810 | 0.15232923 | -0.14846568 | -0.213870921 | 1.716808365 |
| 5 | 1 | 0 | Marker806 | 0.15232923 | -0.14846568 | -0.213870921 | 1.716808365 |
| 6 | 1 | 0 | Marker802 | 0.15232923 | -0.14846568 | -0.213870921 | 1.716808365 |
| 7 | 1 | 0 | Marker807 | 0.15232923 | -0.14846568 | -0.213870921 | 1.716808365 |
| 8 | 1 | 3.154 | Marker362 | 0.255657917 | -0.235833333 | -0.151792929 | 2.650622111 |
| 9 | 1 | 3.904 | Marker622 | 0.504327474 | -0.25297619 | -0.118690476 | 2.8203814 |
| 10 | 1 | 4.404 | Marker642 | 0.368814241 | -0.24146383 | -0.141868316 | 2.704502564 |
| 11 | 1 | 4.905 | Marker638 | 0.286900208 | -0.225620567 | -0.173889237 | 2.593915745 |
| 12 | 1 | 4.905 | Marker635 | 0.286900208 | -0.225620567 | -0.173889237 | 2.593915745 |
| 13 | 1 | 4.905 | Marker637 | 0.286900208 | -0.225620567 | -0.173889237 | 2.593915745 |
| 14 | 1 | 5.405 | Marker882 | 0.220141746 | -0.198196115 | -0.173530563 | 2.125618547 |
| 15 | 1 | 5.655 | Marker1245 | 0.170855401 | -0.20212766 | -0.165771535 | 2.140628808 |
| 16 | 1 | 5.655 | Marker1456 | 0.170855401 | -0.20212766 | -0.165771535 | 2.140628808 |
| 17 | 1 | 5.655 | Marker1505 | 0.170855401 | -0.20212766 | -0.165771535 | 2.140628808 |
| 18 | 1 | 5.655 | Marker1508 | 0.170855401 | -0.20212766 | -0.165771535 | 2.140628808 |
| 19 | 1 | 5.655 | Marker860 | 0.170855401 | -0.20212766 | -0.165771535 | 2.140628808 |
| 20 | 1 | 5.655 | Marker1506 | 0.170855401 | -0.20212766 | -0.165771535 | 2.140628808 |
| 21 | 1 | 5.655 | Marker1458 | 0.170855401 | -0.20212766 | -0.165771535 | 2.140628808 |
| 22 | 1 | 5.655 | Marker1457 | 0.170855401 | -0.20212766 | -0.165771535 | 2.140628808 |
| 23 | 1 | 5.655 | Marker1507 | 0.170855401 | -0.20212766 | -0.165771535 | 2.140628808 |
| 24 | 1 | 5.655 | Marker1248 | 0.170855401 | -0.20212766 | -0.165771535 | 2.140628808 |
| 25 | 1 | 6.405 | Marker1898 | 0.190004724 | -0.189838435 | -0.215671769 | 2.29411771 |
| 26 | 1 | 6.405 | Marker1907 | 0.190004724 | -0.189838435 | -0.215671769 | 2.29411771 |
| 27 | 1 | 6.405 | Marker1702 | 0.190004724 | -0.189838435 | -0.215671769 | 2.29411771 |
| 28 | 1 | 6.405 | Marker1539 | 0.190004724 | -0.189838435 | -0.215671769 | 2.29411771 |
| 29 | 1 | 6.405 | Marker1906 | 0.190004724 | -0.189838435 | -0.215671769 | 2.29411771 |
| 30 | 1 | 6.655 | Marker2327 | 0.324880895 | -0.206904038 | -0.182467552 | 2.325220149 |
| 31 | 1 | 6.905 | Marker2331 | 0.400345917 | -0.213842059 | -0.169511283 | 2.358920032 |
| 32 | 1 | 6.905 | Marker2334 | 0.400345917 | -0.213842059 | -0.169511283 | 2.358920032 |
| 33 | 1 | 7.655 | Marker2871 | 0.42029671 | -0.21875 | -0.227413366 | 2.863415849 |
| 34 | 1 | 7.655 | Marker2879 | 0.42029671 | -0.21875 | -0.227413366 | 2.863415849 |
| 35 | 1 | 7.655 | Marker2872 | 0.42029671 | -0.21875 | -0.227413366 | 2.863415849 |
| 36 | 1 | 7.655 | Marker2880 | 0.42029671 | -0.21875 | -0.227413366 | 2.863415849 |
| 37 | 1 | 7.655 | Marker2878 | 0.42029671 | -0.21875 | -0.227413366 | 2.863415849 |
| 38 | 1 | 7.905 | Marker2660 | 0.311637674 | -0.214760638 | -0.234994264 | 2.857710691 |
| 39 | 1 | 8.655 | Marker2513 | 0.27701596 | -0.209977324 | -0.177183365 | 2.341891672 |
| 40 | 1 | 8.655 | Marker2608 | 0.27701596 | -0.209977324 | -0.177183365 | 2.341891672 |
| 41 | 1 | 8.655 | Marker3595 | 0.27701596 | -0.209977324 | -0.177183365 | 2.341891672 |
| 42 | 1 | 8.655 | Marker2610 | 0.27701596 | -0.209977324 | -0.177183365 | 2.341891672 |
| 43 | 1 | 8.655 | Marker2609 | 0.27701596 | -0.209977324 | -0.177183365 | 2.341891672 |
| 44 | 1 | 9.155 | Marker2769 | 0.479512645 | -0.228180634 | -0.141388976 | 2.452321398 |
| 45 | 1 | 9.155 | Marker2768 | 0.479512645 | -0.228180634 | -0.141388976 | 2.452321398 |
| 46 | 1 | 9.405 | Marker2520 | 0.239475062 | -0.213842059 | -0.169511283 | 2.358920032 |
| 47 | 1 | 9.405 | Marker2563 | 0.239475062 | -0.213842059 | -0.169511283 | 2.358920032 |
| 48 | 1 | 9.405 | Marker2519 | 0.239475062 | -0.213842059 | -0.169511283 | 2.358920032 |
| 49 | 1 | 9.405 | Marker2517 | 0.239475062 | -0.213842059 | -0.169511283 | 2.358920032 |
| 50 | 1 | 9.405 | Marker2518 | 0.239475062 | -0.213842059 | -0.169511283 | 2.358920032 |
| 51 | 1 | 9.655 | Marker2585 | 0.195650562 | -0.198866213 | -0.198003214 | 2.302116335 |
| 52 | 1 | 9.655 | Marker2586 | 0.195650562 | -0.198866213 | -0.198003214 | 2.302116335 |
| 53 | 1 | 10.155 | Marker3445 | 0.365193776 | -0.221088435 | -0.156363516 | 2.406696987 |
[truncated: 389,792 more chars]
